# Iridium-Catalyzed Asymmetric Hydrogenation of Carbocation Precursors via Wagner–Meerwein Rearrangement

**DOI:** 10.1021/jacs.6c01858

**Published:** 2026-05-05

**Authors:** Rajendra K. Mallick, Lars Eriksson, Fan Gong, Pher G. Andersson

**Affiliations:** † Department of Chemistry, Arrhenius Laboratory, Stockholm University,106 91 Stockholm, Sweden; ‡ SILKROAD Research Center of Sustainable Energy Conversion and Utilization & College of Chemistry and Chemical Engineering, 74602 Southwest Petroleum University,610500 Chengdu, Sichuan, China

## Abstract

Carbocation rearrangement is a powerful tool for converting a simple precursor into a complex molecular scaffold. However, controlling the stereoselectivity of a reaction that involves carbocation rearrangement is challenging and remains elusive. In this study, we demonstrate a novel iridium-catalyzed Wagner–Meerwein rearrangement and asymmetric hydrogenation of carbocation precursors (1-(aryl)-1-(1-methylcyclobutyl/cyclopentyl) ethan-1-ol) for the synthesis of various optically active *gem*-dimethyl cycloalkanes. Hence, enantiopure *gem*-dimethyl-containing compounds are important motifs found in many natural products, and some are FDA-approved drugs. Our methodology starts with an iridium-catalyzed formal deoxygenation of tertiary alcohols to generate a tertiary carbocation that triggers the Wagner–Meerwein rearrangement via the ring expansion and alkyl migration cascade to furnish a stable tertiary-benzyl carbocation. Sequential olefination and in situ asymmetric hydrogenation provide access to various *gem*-dimethyl chiral cycloalkanes in excellent yield (> 99%) and enantioselectivity (ee up to > 99%). Otherwise, establishing a high yield and enantioselectivity in a saturated cyclic hydrocarbon next to a sterically hindered *gem*-dimethyl group would not be possible by conventional methods.

## Graphical abstract

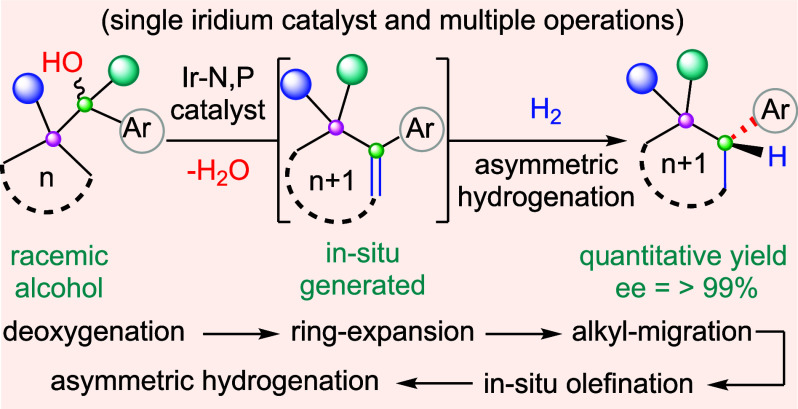

## Introduction

Carbocations are key intermediates in classical organic chemistry and have promoted many transformations via skeletal rearrangement.^1^ However, the use of carbocation intermediates in asymmetric catalysis is a formidable task because of the difficulty to control the facial selectivity in a planar *sp* ^2^-hybridized carbon.^2^ Stereocontrol at a secondary carbocation could be possible via an ion-pair approach^3–6^ or diastereoselective substrate control^7^ but elusive in the case of tertiary carbocations.^8,9^ In 2019, Carreira et al. reported iridium-catalyzed enantioselective reductive deoxygenation of tertiary allenic alcohols **A** by using Hantzsch ester as the hydride donor (Scheme 1a).^10^ The stereoselectivity is controlled by blocking one of the faces of a planar tertiary-benzyl-allenic carbocation **B** by its coordination with the metal center of the chiral iridium complex. Hence, the reductant Hantzsch ester could easily deliver hydride ion stereoselectively, leading to high enantio- and regioselectivities (Scheme 1a). In the same year, our group also developed an iridium-catalyzed enantioselective deoxygenation of racemic tertiary-benzyl alcohols **D** for the construction of chiral alkanes **F** via asymmetric hydrogenation (Scheme 1b).^11^ The reaction involves a tertiary-benzyl carbocation **E** which is stabilized by its complexation with the metal center of the Ir-hydride complex due to the π-stacking interaction (Scheme 1b). Later in 2023, we found that the secondary hydroxy group of a tetrasubstituted olefin tethered allylic alcohol could undergo iridium-catalyzed double convergent 1,3-rearrangement/hydrogenation that provides enantioenriched-single diastereomer of chiral alcohols **I** (Scheme 1c).^12^ Since tetrasubstituted olefins **G** are unreactive^13^ under iridium-catalyzed asymmetric hydrogenation, it rearranges to a reactive trisubstituted^14^ olefin **H** which is prone to undergo enantioconvergent^15^ hydrogenation rapidly (Scheme 1c).

**Scheme 1:**
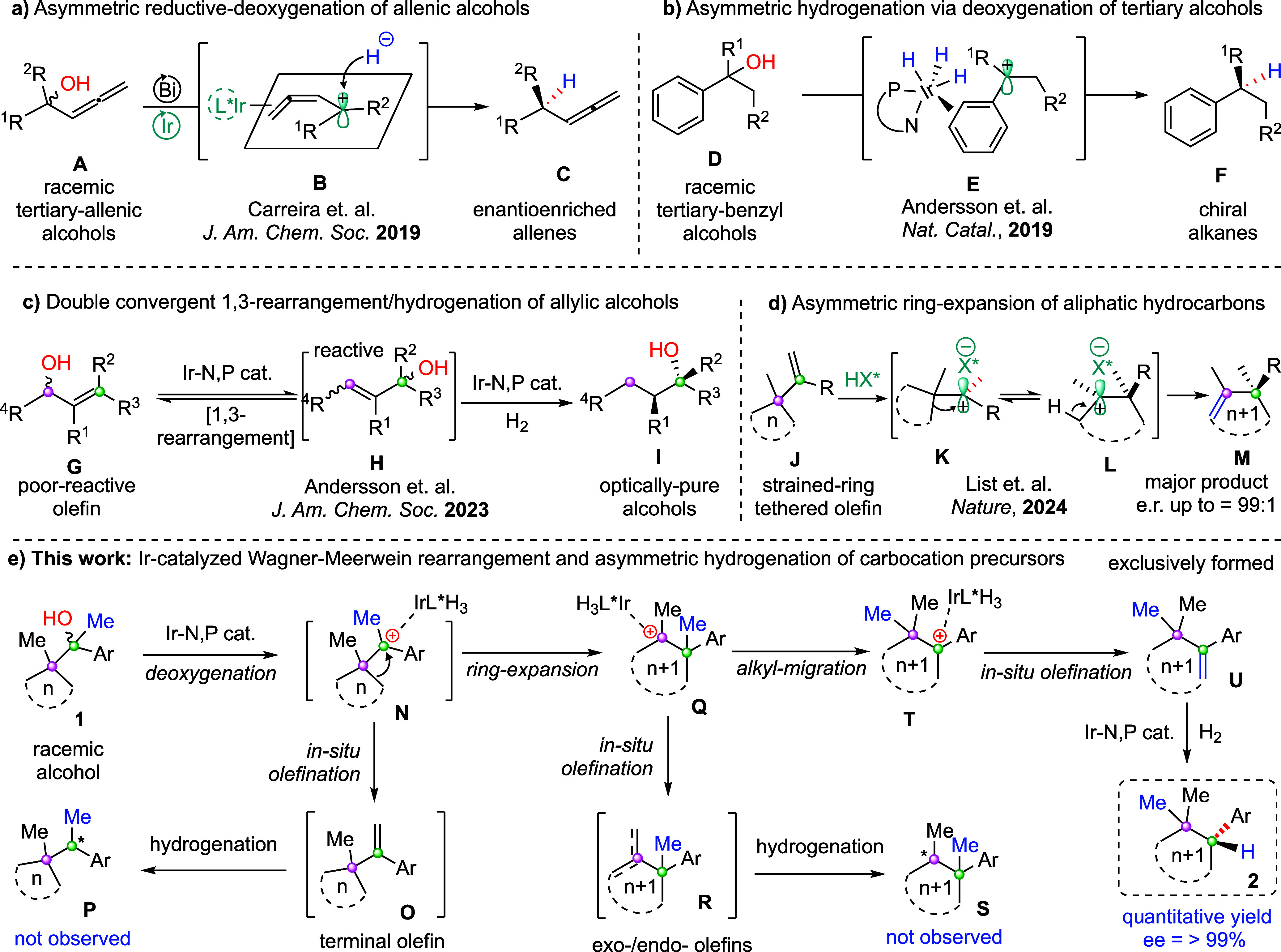
Asymmetric Deoxygenation and Rearrangement of Carbocation Precursors

Wagner–Meerwein rearrangement^16^ is a fundamental acid-catalyzed 1,2-shift of a carbocation precursor to generate a stable carbocation intermediate that could be either trapped by a nucleophile or lead to olefin in situ by deprotonation. However, controlling the stereoselectivity by such a rearrangement is challenging and less explored. In this context, an enantioselective Wagner–Meerwein rearrangement of aliphatic hydrocarbons was reported by List and co-workers in 2024 (Scheme 1d).^17^ The chiral imidodiphosphorimidate (HX*) triggers the asymmetric ring expansion of (1-(1-methylcyclobutyl/cyclopentyl)­vinyl) alkanes/arenes **J** via cationic rearrangement and provides access to various chiral cycloalkenes **M**. The carbocation intermediate **K** generated via protonation of substrate **J** with HX* could perfectly fit inside the pocket of the ligand anion (*X^–^) due to its higher buried volume and hence is responsible for asymmetric induction and origin of enantioselectivity (Scheme 1d).

Nevertheless, the use of Wagner–Meerwein rearrangement in asymmetric hydrogenation is not known so far. Hence, as our ongoing research on iridium-catalyzed asymmetric hydrogenation of olefins,^18^ we were interested to use a carbocation precursor for the cationic rearrangement and enantioconvergent hydrogenation. In general, asymmetric hydrogenation of olefins is well explored and has been extensively used for many decades toward the synthesis of various value-added enantioenriched products.^19^ However, the vast majority of these reactions are enantiodivergent^20^ and require an isomerically pure olefin. The major disadvantages of classic olefination reactions are as follows: It (i) proceeds under harsh reaction conditions, (ii) produces substantial wastes, and (iii) requires tedious purification procedures.^21^ A potential alternative is to choose an alcohol precursor which could be easily obtained from the corresponding ketone by the addition of an organometallic reagent.^22^ Hence, under slightly acidic conditions, this could lead to olefin in situ by removal of a simple water molecule as a sole byproduct.^23^ Considering the Bronsted acidic properties of chiral iridium N, P-complexes,^24^ we envisioned a Wagner–Meerwein rearrangement and asymmetric hydrogenation of tertiary alcohol precursor **1** (Scheme 1e). Iridium-catalyzed deoxygenation of tertiary alcohol **1** could form the tertiary benzyl carbocation **N**. The carbocation intermediate **N** could either undergo terminal olefination followed by asymmetric hydrogenation leading to chiral alkane **P** (via int-**O**), or it could undergo a strain release ring expansion of int-**N** to form a new tertiary carbocation intermediate **Q**. From the intermediate **Q**, we could expect *exo*/endo olefination that forms the olefin **R** in situ followed by asymmetric hydrogenation to provide **S**. However, we have not observed the hydrogenation products **P** and **S** during the course of the reaction, hence suggesting the absence of olefins **O** and **R** during the reaction sequence. Rather, it undergoes unprecedented further methyl migration from a tertiary-benzyl carbon of int-**Q** to the tertiary-alkyl enabled carbocation center and generates a new stable tertiary-benzyl carbocation **T**. In situ olefination via deprotonation could lead to trisubstituted olefin **U** and finally undergo iridium-catalyzed enantioselective hydrogenation to form enantioenriched *gem*-dimethyl cycloalkanes **2**. Surprisingly, we have exclusively obtained chiral cycloalkanes **2** in an excellent yield (up to > 99%) and enantioselectivity (up to > 99%) via a cascade cationic rearrangement. This concept has not been exploited in the literature and could open up a straightforward route to construct a chiral center next to a sterically hindered bulky *gem*-dimethyl group. Moreover, *gem*-dimethyl-containing compounds are found in numerous natural products, bioactive molecules, and FDA-approved drugs, and hence a convenient and novel synthetic route is highly desirable.^25^

## Results and Discussion

At the onset, we were interested to study the acid-mediated olefination of 1-(1-methylcyclopentyl)-1-phenylethan-1-ol **1a′** (R = H) without iridium catalyst and hydrogen pressure (Table 1, entries 1–6). Hence, the tertiary alcohol **1a′** could possibly form a series of olefins (**1aa**–**ad**) on treatment with acid via deoxygenation and rearrangement. Various solvents (toluene, chloroform, dichloromethane, fluorobenzene, trifluorotoluene, and hexafluorobenzene) were investigated in the presence of 1.0 equiv of methanesulfonic acid (MsOH) at room temperature overnights (Table 1, entries 1–6). Although a mixture of four different olefins **1aa**–**ad** formed in each case, the major product obtained is the six-membered endocyclic olefin **1ac** which is generated by deoxygenation and ring expansion of **1a′** (Table 1, entries 1–6). The formation of **1ac** is higher when fluorinated solvents such as fluorobenzene, trifluorotoluene, and hexafluorobenzene were used (Table 1, entries 4–6). This result is in agreement with List’s Bronsted acid-catalyzed ring expansion of cyclopentane derivatives.^17^ Hence, utilizing the Bronsted^24^ acidic properties of chiral Ir–N, P catalysts and inspired by our preliminary studies, we were motivated to carry out the iridium-catalyzed ring expansion and asymmetric hydrogenation of carbocation precursor **1a** (R = OMe) without MsOH (Table 1, entries 7–14). In this regard, the reaction was investigated by employing different chiral ligands under 50 bar hydrogen pressure in toluene at room temperature for 24 h (Table 1, entries 7–9). To our delight, the reaction in the presence of thiazole-based chiral ligand **L1** undergoes complete conversion and delivered the hydrogenation product **2a** in 40% yield along with 60% unreacted olefin **1ad** (Table 1, entry 7). Surprisingly, this finding is in contrast with our preliminary result with MsOH (Table 1, entries 1–6) and the List et al. ring expansion work. In other words, further methyl migration takes place from the tertiary benzyl carbon to the tertiary carbocation center of **int-Q** (Scheme 1e) and hence forms a stable tertiary benzyl carbocation (**int-T;** Scheme 1e). In situ olefination provides olefin **1ad,** and asymmetric hydrogenation in the presence of chiral Ir–N, P catalysts delivers fully saturated optically active *gem*-dimethyl cycloalkanes **2a**. Chiral imidazole ligand **L2** was not beneficial and gave unreacted olefin **1ad** in 70% yield (Table 1, entry 8). Surprisingly, to our delight, the reaction in the presence of oxazole ligands **L3** provides the desired product **2a** in 99% yield and ee > 99% (Table 1, entry 9). Lowering the catalyst to 1 mol % decreased the product formation (**2a**) to 74% (Table 1, entry 10). Reaction in dichloromethane (DCM) did not deliver the desired product **2a** (< 5%), leaving the unreacted olefins **1ac** and **1ad** in 23 and 70%, respectively (Table 1, entry 11). A similar result was obtained in fluorobenzene (**2a** = 38%), but in this case, olefin **1ac** (42%) is major (Table 1, entry 12). Interestingly, lowering the hydrogen pressure to 20 and 10 bar did not affect the reaction outcome and provides the desired product **2a** in excellent yield (> 99%) and enantioselectivity (ee ≥ 99%) (Table 1, entries 13 and 14). Hence, the optimized reaction condition was chosen by using 2.0 mol % [Ir-L3*­(cod)]^+^[B­(Ar_F_)_4_]^−^ and 10 bar H_2_ in toluene at room temperature for 24 h (Table 1, entry 14).

**Table 1:**
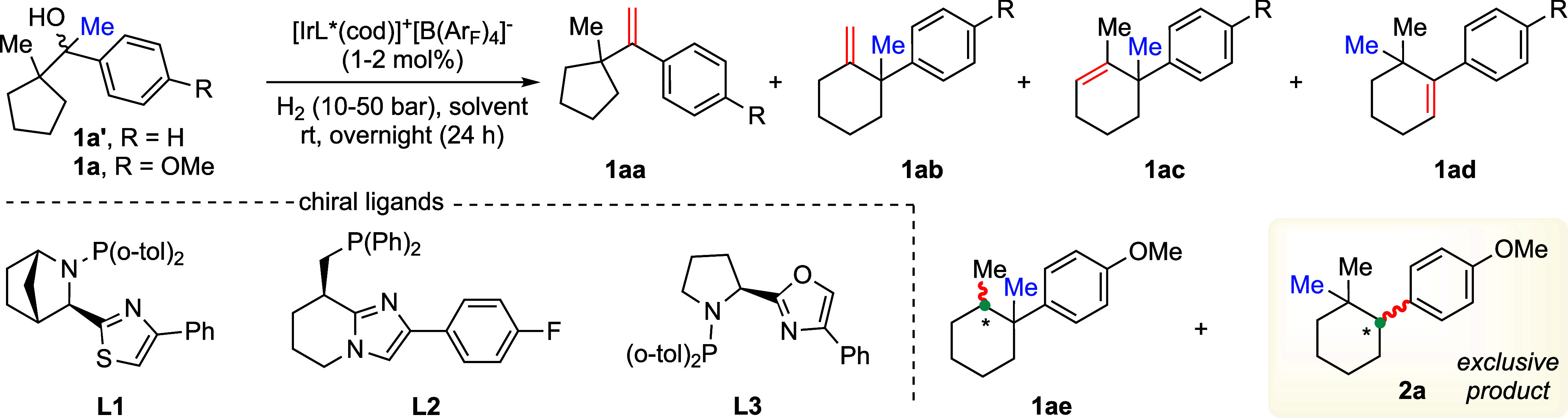
Reaction Development and Optimization^a^

SL	ligand	MsOH (equiv)	H_2_ (bar)	solvent	conv. (%)	**1aa** (%)	**1ab** (%)	**1ac** (%)	**1ad** (%)	**1ae** (%)	**2a** (%)	ee **2a**
1		1.0		toluene	100	2	7	76	15			
2		1.0		CHCl_3_	100	19	25	54	2			
3		1.0		CH_2_Cl_2_	100	5	27	56	12			
4		1.0		PhF	100	<1	3	87	9			
5		1.0		PhCF_3_	100	<1	2	84	13			
6		1.0		C_6_F_6_	100	<1	2	86	11			
7	L1		50	toluene	100				60		40	
8	L2		50	toluene	100				70		25	
9	L3		50	toluene	100				<1		99	99%
10^b^	L3		50	toluene	100				26		74	
11	L3		50	CH_2_Cl_2_	100			23	70	2	5	
12	L3		50	PhF	100			42	8	7	38	
13	L3		20	toluene	100				<1		99	99%
14	L3		10	toluene	100			0	<1		99	99%

a Reaction conditions: 0.025 mmol of substrate, 2 mol % Ir–N, P catalyst, 1 mL of solvent, room temperature, 24 h.

b 1 mol % catalyst used. NB: R = H for entries 1–6 and R = OMe for entries 7–14.

With the optimized condition in hand, we scrutinized the scope of the reaction by varying the ring sizes, migrating groups, and substituents on the aromatic ring (Scheme 2). First, we evaluated the ring expansion of cyclopentanes by changing the substituents on the *para* position of the aromatic ring. Electron-donating groups such as methoxy, methyl, isopropyl, and *tert*-butyl underwent the Wagner–Meerwein rearrangement and asymmetric hydrogenation very smoothly and provides desired products **2a**–**d** in excellent yield (up to 99%) and enantioselectivity (ee ≥ 99%). Aromatic substituents (Ph and OPh) on the *para* position of the aromatic ring worked well and provide the desired products **2e** and **2f** in 99% yield and ee up to > 99%, although the reaction time was longer. Not only methyl is migrated but also other groups such as hydride, ethyl, and ^*n*^butyl successfully migrated during the course of the reaction leading to the formation of **2g**–**i** in excellent yield (up to 96%) and enantioselectivity (ee up to 99%) but an inseparable mixture of diastereomers. Next, we were interested in studying the ring expansion of cyclobutanes by varying the substituents on the aromatic ring. In this case, the chiral thiazole ligand **L1** is found to be suitable, leading to full conversion and excellent enantioselectivity. As such, various electron-donating substituents (Me, Et, ^*i*^Pr, ^*t*^Bu) on the *para* position of the benzene ring were scrutinized and delivered the desired products **2j**–**n** in both excellent yield (99%) and enantioselectivity (up to ee ≥ 99%). Also, *p*-bromo biphenyl-tethered *gem*-dimethyl cyclopentane has no exception and delivered **2o** in 64% yield with 99% enantioselectivity. To our delight, long-chain aliphatic groups such as Et and ^*n*^Bu successfully migrated over the reaction forging **2p** and **2q** in quantitative yield and ee up to 99%. Moreover, hydride ions are migrated during the course of the reaction and delivered **2r**–**s** in very good yield (up to 84%) and ee up to > 99%, although there is no control over diastereoselectivity. Next, we focused on screening various heterocycles. Thiophene-bearing *gem*-dimethyl cyclopentane **2t** was obtained in satisfactory yield but with poor enantioselectivity (ee = 50%). The reason for poor enantioselectivity is unclear. Pleasingly, benzothiophene is well tolerated and furnished enantioenriched **2u** in good yield and excellent enantioselectivity (ee ≥ 99%). Disappointingly, a complex reaction mixture (**2v**) was observed from the benzofuran-tethered starting material **1v**. Moreover, sterically hindered *o*-OMe substituent could not deliver the desired product (**2w**). A complex reaction mixture was noticed when 3-membered cyclopropane was employed (**2x**). Similarly, the cyclohexane ring did not undergo expansion and led to unreacted starting material (**2y**). As expected, thiazole- containing product **2z** could not be achieved probably due to catalyst poisoning. Aliphatic hydrocarbon leads to a complex reaction mixture under standard reaction conditions (**2aa**). Similarly, a complex mixture was observed with an electron-withdrawing group such as *p-*F-aryl-substituted alcohol precursor **1″b** and provides the desired product in 48% yield (by NMR) along with an inseparable mixture of other byproducts (see SI). This result could be due to the destabilization of the carbocation intermediate by the electron-withdrawing group.

**Scheme 2:**
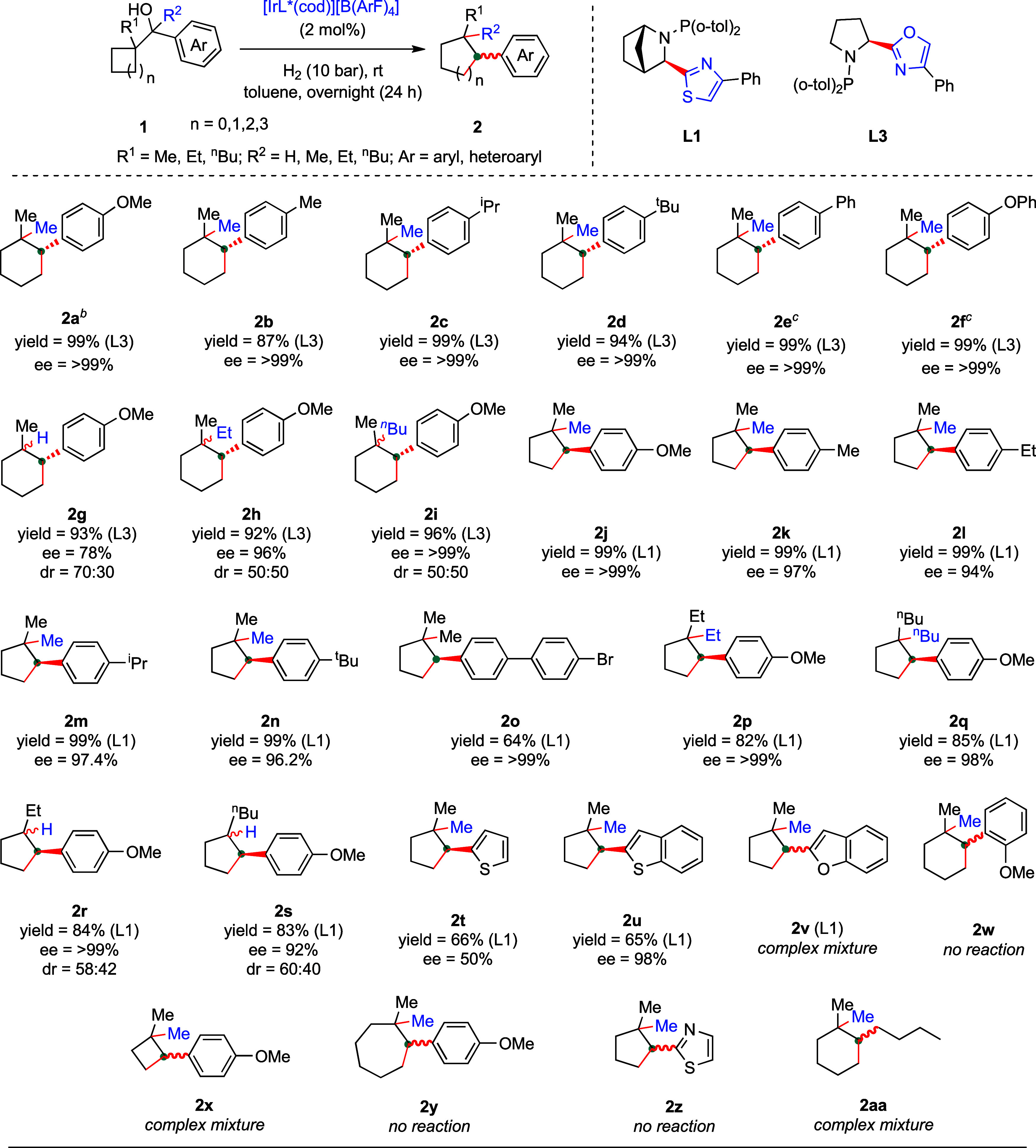
Scope of the Reaction^a^

a Reaction conditions: 0.1 mmol of substrate, 2.0 mol % Ir–N,P catalyst, 10 bar H_2_, 3 mL dry toluene, room temperature, 24 h.
b Reaction was carried out in 1.0 mmol scale.
c Stirred for 60 h.

To gain insight into the reaction mechanism, a series of control experiments were performed. We were interested to study the reaction of the alcohol precursor **1a** in the presence of an active iridium complex Ir-L3** under an argon atmosphere (Scheme 3b). The active iridium complex Ir-L3** was generated in situ upon treatment of precatalyst Ir-L3* under H_2_-ballon pressure at room temperature in toluene (Scheme 3a). Reaction of **1a** with active iridium complex Ir-L3** under an argon atmosphere exclusively delivered the desired olefin **1ad** in quantitative yield (90%) (Scheme 3b). This result supports that the iridium catalyst is responsible for the Wagner–Meerwein rearrangement. To further investigate the reaction mechanism, the preformed olefin intermediate **1ad** was subjected to standard asymmetric hydrogenation conditions and provided the desired product **2a** (Scheme 3c). This result also supports that the reaction proceeds through a *gem*-dimethyl tethered olefin **1ad** intermediate. Finally, a deuterium labeling study was performed with the carbocation precursor **1g** under optimized reaction conditions by using D_2_-gas (Scheme 3d). Complete deuterium incorporation (D = 100%) was obtained in the tertiary-benzyl carbon (labeled in green), and no deuterium incorporation (D = 0%) was observed in the tertiary-alkyl carbon (labeled in blue). These experiments support the existence of olefin intermediate **1ad** (Scheme 3d).

**Scheme 3:**
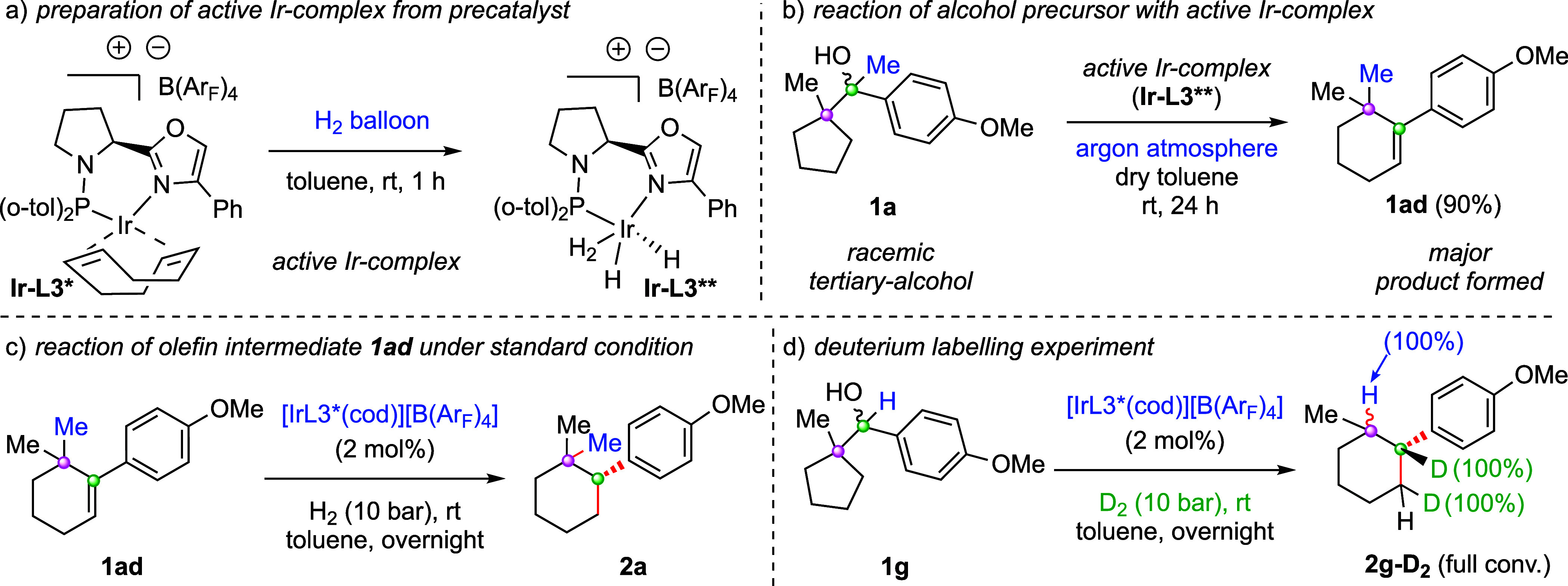
Mechanistic Investigations

The synthetic potential of enantiopure *gem*-dimethyl cycloalkanes was examined by converting the methyl ether of **(** *R* **)-2a** (ee ≥ 99%) and **(** *S* **)-2j** (ee ≥ 99%) to corresponding phenol derivatives **(** *R* **)-3** (ee ≥ 99%) and **(** *S* **)-5** (ee ≥ 99%) in 65 and 43% yields, respectively. The ee values of **(** *R* **)-3** and **(** *S* **)-5** were determined by reconverting back to starting materials **(** *R* **)-2a** and **(** *S* **)-2j** using the methylation procedure (Scheme 4). To our delight, further modification of **(** *R* **)-3** and **(** *S* **)-5** with ferrocene carboxylic acid leads to the formation of enantioenriched ferrocene ester complexes **(** *R* **)-4** and **(** *S* **)-6**.^26^ The absolute configurations of **(** *R* **)-4** and **(** *S* **)-6** were determined by using single-crystal X-ray crystallographic analysis (Scheme 4). Based on the elution order in SFC and optical rotation values, the stereochemistry of the remaining compounds was assigned. Thus, the ***R***-configuration of products **2a**–**i** is formed when oxazole ligand **L3** was used, while opposite stereochemistry (***S***-configuration) was observed for compounds **2j**–**u** with thiazole ligand **L1.**

**Scheme 4:**
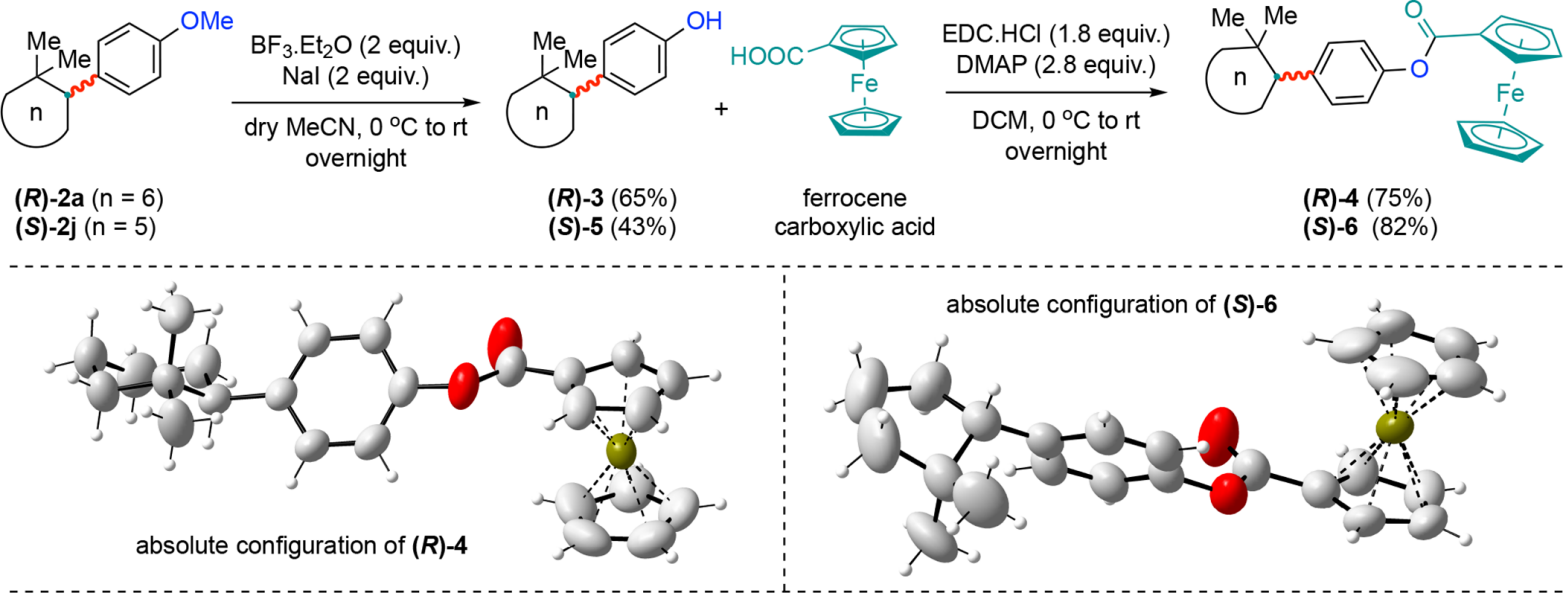
Synthetic Modifications and Determination of Absolute Stereochemistry^a^

a Reaction conditions: (i) starting material **(** *R* **)-2a/(** *S* **)-2j** (1.0 mmol), BF_3_.Et_2_O (2.0 equiv), NaI (2.0 equiv), dry MeCN (5 mL), rt, overnight; (ii) alcohol precursor **(** *R* **)-3/(** *S* **)-5** (0.1 mmol), ferrocene carboxylic acid (1.3 equiv), DMAP (2.8 equiv), EDC.HCl (1.8 equiv), DCM (5 mL), rt, overnight. NB: ee of compounds **(** *R* **)-3** and **(** *S* **)-5** were determined after reconverting back to **(** *R* **)-2a** and **(** *S* **)-2j** (see the SI).

## Conclusions

In summary, we have discovered a novel catalytic route to synthesize enantiopure *gem*-dimethyl cycloalkanes in excellent yield (> 99%) and enantioselectivity (ee ≥ 99%). This methodology represents the first example for the iridium-catalyzed asymmetric hydrogenation of carbocation precursors via Wagner–Meerwein rearrangement. Hence, the concept would open up a new era in asymmetric hydrogenation via rearrangement.

## Supplementary Material



## Notes

Experimental procedures, spectroscopic and crystallographic data, NMR spectra (^1^H and ^13^C), and SFC chromatograms (PDF)
