# Supplementary material for: Iridium-Catalyzed Asymmetric Hydrogenation of Carbocation Precursors via Wagner–Meerwein Rearrangement
Source: J Am Chem Soc. 2026 May 5;148(19):19781–8. doi: 10.1021/jacs.6c01858 (PMC13195667; doi:10.1021/jacs.6c01858)
Supplement: Supplementary file 1 [file ja6c01858_si_001.pdf]

Supporting Information

Iridium-Catalyzed Asymmetric Hydrogenation of Carbocation Precursors via Wagner-Meerwein Rearrangement

Rajendra K. Mallick,[a] Lars Eriksson,[a] Fan Gong,[b] and Pher G. Andersson*[a]

[a]Department of Chemistry, Stockholm University, Svante Arrhenius väg 16C, SE-10691 Stockholm, Sweden.

[b]SILKROAD Research Center of Sustainable Energy Conversion and Utilization & College of Chemistry and Chemical Engineering, Southwest Petroleum University, 610500 Chengdu, Sichuan, China

Table of Contents

General information	S2
List of starting materials	S3
General procedures	S3
Experimental data of starting materials	S7
Experimental data of final products	S16
General procedure for methoxy cleavage	S23
General procedure for synthesis of ferrocene complex	S24
General procedure for O-methylation	S25
X-ray crystallography	S26
References	S28
NMR spectra of starting materials	S29
NMR spectra of final products	S57
SFC chromatograms	S83

General Information

All reactions were performed under anhydrous conditions (unless otherwise stated) under a dry nitrogen or argon atmospheres in glassware that were dried in a vacuum oven (160 °C) and cooled down to room temperature under a flow of nitrogen prior to use. Reaction mixtures were stirred magnetically. Air- and moisture-sensitive liquids and solutions were transferred via syringe into the reaction vessels through rubber septa. The commercially available chemicals were purchased (unless specified) at highest commercial quality and used as received. Non-anhydrous solvents were purchased (unless specified) at the highest commercial quality and used as received. Toluene, tetrahydrofuran (THF) and diethyl ether (Et_2O) were dried using sodium-benzophenone and freshly distilled under nitrogen. Anhydrous CH_2Cl_2 was obtained by freshly distillation over CaH_2 under nitrogen atmosphere. Reactions at 0 °C was conducted in an ice bath and acetone/dry ice bath used for reactions at -78°C. All heating reactions were done either in a heating block (dry syn) or in an oil bath. All hydrogenation reactions were performed in a high-pressure hydrogenation apparatus (Parr MS5000).

TLC: TLC was performed on aluminium backed silica plates coated with Kiesel gel 60 (0.20 mm, UV 254 nm) and visualized under ultraviolet (UV) light followed by staining with potassium permanganate/Δ, 2,4-dinitrophenylhydrazine (2,4-DNP)/Δ, or bromocresol green/Δ.

Chromatography: Chromatographic separations were performed on Kiesel gel 60 H silica gel (particle size: 0.063-0.100 mm) using manual glass columns.

^{1}H NMR: Spectra were recorded on Bruker at 400 MHz in $CDCl_3$ and referenced internally to the residual $CHCl_3$ signal (7.26 ppm). Chemical shifts (δ H) are quoted in parts per million (ppm). Spin-spin coupling constants (*J*) are reported in Hertz (Hz). 2D NMR experiments COSY, NOESY, HSQC and HMBC were obtained where necessary for structure elucidation.

^{13}C NMR: Spectra were recorded on Bruker NMR (101 MHz) instruments. Chemical shifts (δ C) are quoted in parts per million (ppm) and referenced to the central peak of $CDCl_3$ (77.00 ppm).

HRMS: High resolution mass spectrometric (HRMS) data were obtained from a Bruker microTOF-Q II instrument operated at ambient temperatures and GC-APCI-TOF.

Optical rotation: Optical rotation was recorded on a thermostated polarimeter using a sodium lamp (589 nm) and a 10 cm cell.

SFC: Enantiomeric excesses were determined using SFC (250 mm Chiralcel or Chiralpak columns, CO_2/MeOH) using chiral stationary phases. Racemic compounds were in all cases used for comparison.

Table S1: List of Starting Materials

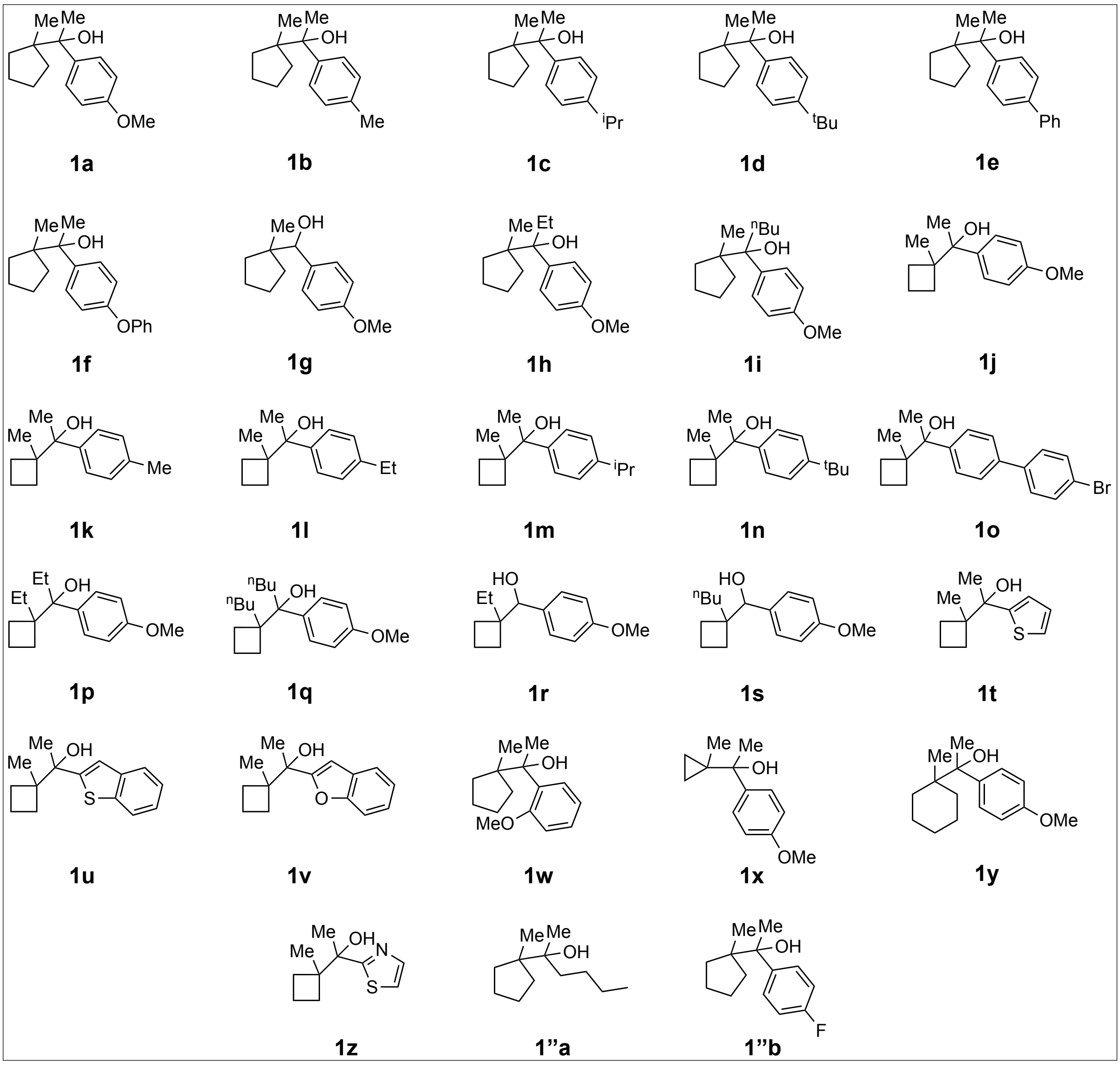

General Procedures:

General Procedure 1 (GP-1): Alkylation of cyclobutane/cyclopentane carboxylic acid

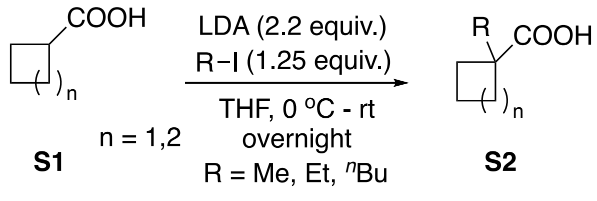

The compound **S2** was synthesized following the known literature procedure.[1,2,3] An oven dried 250 mL round-bottom (RB) flask equipped with a magnetic stir bar, capped with a Suba seal was cooled under vacuum and back-filled with nitrogen three times. Freshly distilled diisopropylamine (15.52 mL, 110 mmol, 2.2 equiv.) and dry THF (50 mL) was added under N_2 atmosphere. The solution was cooled to 0 °C, and ^{n}BuLi (2.5 M in hexane, 44 mL, 110 mmol, 2.2 equiv.) was added dropwise. After 5 minutes,

the mixture was warmed up to rt slowly and was stirred for another 15 minutes at same temperature. Again, the reaction mixture was cooled down to 0 °C. A solution of cyclobutane/cyclopentane carboxylic acid (**S1**, 50 mmol, 1.0 equiv.) in THF (10 mL) was slowly added to the reaction mixture, and the resulting mixture was slowly warmed to room temperature (NB: in case of cyclopentane carboxylic acid, the reaction was heated at 45 °C and stirred for 1 h). Then, the reaction mixture was cooled to 0 °C, and alkyl iodide (3.89 mL, 62.5 mmol, 1.25 equiv.) was slowly added. The reaction mixture was allowed to warm to rt and stirred overnight. Then, the mixture was cooled down to 0 °C in an ice/water bath, and 10% HCl (50 mL) was slowly added. The layers were separated and the aq. layer was extracted with Et_2O (3 x 40 mL), the combined organic layers were washed with brine (50 mL), dried over Na_2SO_4, filtered and the solvents removed under reduced pressure. The crude product was purified by silica-gel column chromatography and provides **S2** in quantitative yield as dark-yellow liquid.

General Procedure 2 (GP-2): Preparation of Weinreb Amides

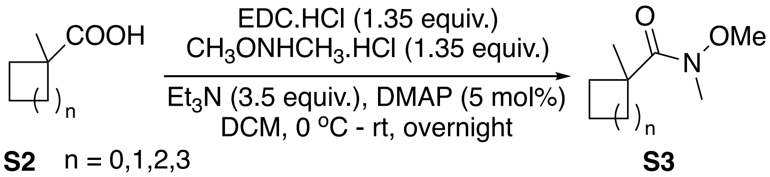

The Weinreb Amides **S3** was synthesized following the known literature procedure.[1,2,3] The 1-methylcycloalkane-1-carboxylic acid (**S2**, 40 mmol, 1.0 equiv.) was taken in an oven dried 250 mL RB flask. Dry CH_2Cl_2 (120 mL) was added under nitrogen atmosphere. The solution was cooled down to 0 °C in an ice bath. Then, N, O-dimethylhydroxylamine hydrochloride (5.27 g, 54 mmol, 1.35 equiv.), N-(3-Dimethylaminopropyl)-N′-ethylcarbodiimide (EDC) hydrochloride (10.35 g, 54 mmol, 1.35 equiv.) and 4-(Dimethylamino)pyridine (0.244 g, 2.0 mmol, 5 mol%) was added sequentially under nitrogen atmosphere. Then triethylamine (19.52 mL, 140 mmol, 3.5 equiv.) was slowly added. The reaction was warmed up slowly to rt and allowed to stir overnight. Upon completion, the reaction mixture cooled to 0 °C, and 1M HCl solution (50 mL) was slowly added. The organic phase was separated and then washed with 1M HCl solution (2x30 mL), water (2x30 mL), sat. aq. $NaHCO_3$ (2x30 mL), and finally washed with brine (40 mL). The organic layer was dried with Na_2SO_4, filtered and concentrated in vacuo. The crude product was purified by silica gel column chromatography (EtOAc/^{n}pentane) and provides corresponding Weinreb amide **S3**.

General Procedure 3 (GP-3): Synthesis of aryl-ketones via Grignard reaction

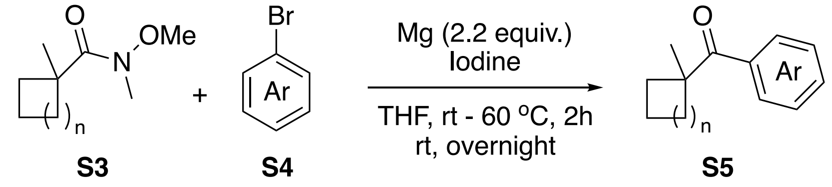

The ketone **S5** was synthesized following the known literature procedure.[2] An oven dried 100 mL 2-neck round bottom flask equipped with a magnetic stir bar was cooled under a nitrogen flow desiccator. The flask was capped with a Suba seal and fitted with a vertical reflux condenser. The upper end of the condenser is connected to the Schlenk-line. An activated magnesium turnings (2.2 equiv., 6.6 mmol) and a small crystal of iodine was added in to the flask under nitrogen atmosphere. The flask was again evacuated and refilled with nitrogen three times. A solution of aryl bromide (**S4**, 2.2 equiv., 6.6 mmol) in dry THF (10 mL) was added slowly at room temperature. Then the mixture was heated to 60 °C and stirred for 2 hours. The reaction was cooled down to room temperature and then to 0 °C in an ice bath. A solution of Weinreb amide (**S3**, 3.0 mmol, 1.0 equiv.) in dry THF (3 mL) was slowly added under nitrogen atmosphere. The reaction was stirred further overnight warming up slowly to rt. Then the reaction mixture was cooled in an ice/water bath, and sat. aq. NH_4Cl solution (10 mL) was slowly added. The layers were separated and the aqueous layer was extracted with diethyl ether (3x15 mL). The combined organic layers were collected and dried over Na_2SO_4, filtered and concentrated under reduced pressure. The crude product was purified by silica gel flash column chromatography (EtOAc/^{n}pentane). Upon evaporation of the solvent under reduced pressure, the corresponding ketone **S4** was obtained.

General Procedure 4 (GP-4): Synthesis of heteroaryl-ketones via lithiation

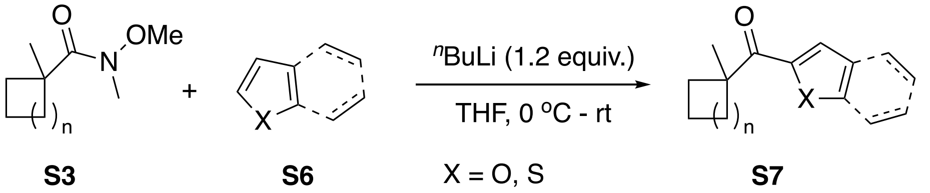

An oven dried 100 mL round-bottom (RB) flask equipped with a magnetic stir bar, capped with a Suba seal was cooled under vacuum and back-filled with nitrogen three times. Heteroarenes (**S6**, 2.2 mmol, 1.1 equiv.) and dry THF (8 mL) were added in to the flask under nitrogen atmosphere. The flask containing heteroarenes **S6** and dry THF was cooled down to 0 °C in an ice bath. Then, ^{n}BuLi (1.2 equiv., 2.4 mmol) was added dropwise manner. After stirring the reaction mixture for 30 minutes at same temperature, Weinreb amide (**S3**, 1.0 equiv., 2.0 mmol) in dry THF (2 mL) as slowly added. The progress of the reaction was monitored by TLC. Upon completion, the reaction was quenched with saturated aqueous NH_4Cl. The organic layers separated and the aqueous layer was extracted three times with diethyl ether (3x10 mL). Dried over anhydrous sodium sulphate and evaporated under reduced

pressure. The crude residue was purified by silica gel flash column chromatography and provides the desired compound **S7**.

Procedure for the synthesis of (4'-bromo-[1,1'-biphenyl]-4-yl)(1-methylcyclobutyl)methanone S8

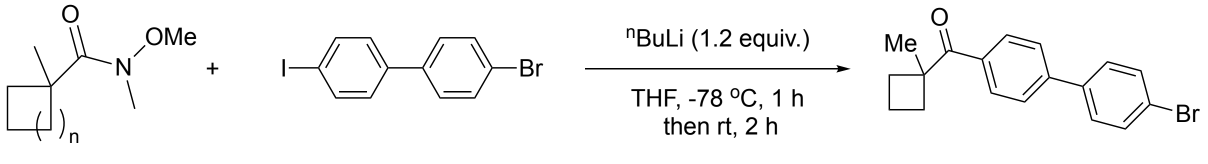

S3 **S8**

An oven dried 100 mL round-bottom (RB) flask equipped with a magnetic stir bar, capped with a Suba seal was cooled under vacuum and back-filled with nitrogen three times. Then, 4-bromo-4'-iodo-1,1'-biphenyl (3.3 mmol, 1.1 equiv.) and dry THF (25 mL) were added in to the flask under nitrogen atmosphere. The flask containing 4-bromo-4'-iodo-1,1'-biphenyl and dry THF was cooled down to -78 °C (dry ice-acetone). Then, ^{n}BuLi (1.2 equiv., 3.6 mmol) was added dropwise manner. After stirring the reaction mixture for 1 hour at same temperature, Weinreb amide (**S3**, 1.0 equiv., 3.0 mmol) in dry THF (2 mL) as slowly added. The reaction mixture was warmed slowly to room temperature and stirred for additional 2 hours at rt. The progress of the reaction was monitored by TLC. Upon completion, the reaction was quenched with saturated aqueous NH_4Cl. The organic layers separated and the aqueous layer was extracted three times with diethyl ether (3x10 mL). Dried over anhydrous sodium sulphate and evaporated under reduced pressure. The crude residue was purified by silica gel flash column chromatography and provides the desired compound **S8**.

General Procedure 5 (GP-5): Synthesis of tertiary alcohols

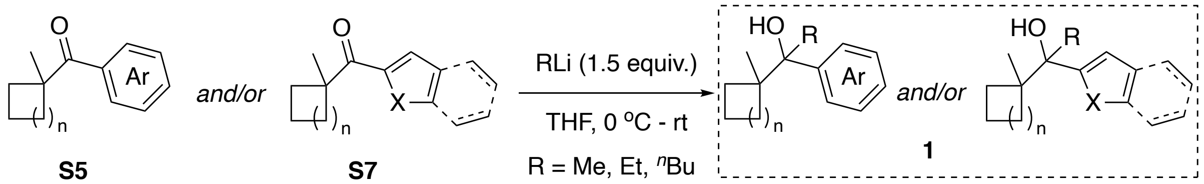

S5 **S7**

To an oven dried 50 mL round-bottom (RB) flask equipped with a magnetic stir bar, capped with a Suba seal was added the corresponding ketones **S5** and **S7** (1.0 mmol). The flask containing the starting materials (**S5** and **S7**) was evacuated under vacuum and back-filled with nitrogen three times. Dry THF (5 mL) was added in to the flask under nitrogen atmosphere and was cooled down to 0 °C in an ice bath. Then, alkyl lithium (1.5 equiv., 1.5 mmol) was added dropwise manner at 0 °C. The progress of the reaction was monitored by TLC. Upon completion, the reaction was quenched with saturated aqueous NH_4Cl. The organic layers separated and the aqueous layer was extracted three times with diethyl ether (3x10 mL). Dried over anhydrous sodium sulphate and evaporated under reduced pressure. The crude residue was purified by silica gel flash column chromatography and provides the desired compound **1**.

General Procedure 6 (GP-6): Synthesis of secondary alcohols

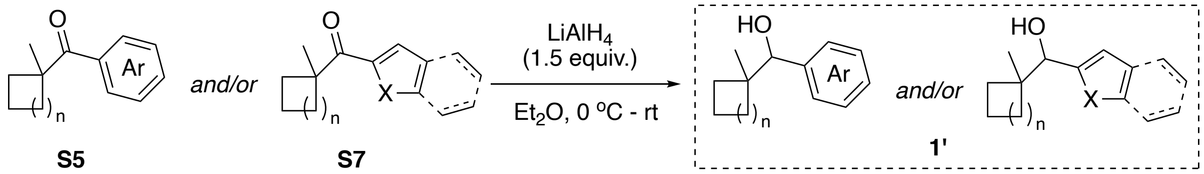

To an oven dried 50 mL round-bottom (RB) flask equipped with a magnetic stir bar, capped with a Suba seal was added $LiAlH_4$ (1.0 equiv., 1.0 mmol) under nitrogen atmosphere. Freshly prepared dry diethyl ether (5 mL) was added subsequently and cooled down to 0 ºC in an ice bath. Then, the corresponding ketones **S5** and **S7** (1.0 mmol, 1.0 equiv.) in dry diethyl ether (1 mL) was added slowly in dropwise manner at 0 ºC. Upon completion, the reaction was quenched very slowly with required amount of saturated aqueous Na_2SO_4 (~ 1 – 2 mL) at 0 ºC. The reaction mixture was dried with anhydrous Na_2SO_4, filtered and the solvent was removed under reduced pressure. The crude product was purified by silica gel flash column chromatography and provides the desired compound **1'**.

Experimental data of starting materials:

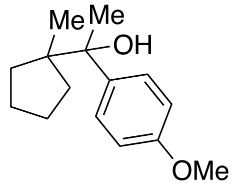

1-(4-Methoxyphenyl)-1-(1-methylcyclopentyl)ethan-1-ol (1a): (Synthesized according to GP-5, 3.2 mmol scale, yield = 55%, colorless oil): **^{1}H NMR (400 MHz, CDCl$_3$)** δ 7.42 – 7.35 (m, 2H), 6.88 – 6.80 (m, 2H), 3.81 (s, 3H), 2.01 – 1.82 (m, 2H), 1.67 – 1.60 (m, 1H), 1.60 (s, 3H), 1.59 – 1.55 (m, 3H), 1.55 – 1.53 (m, 1H), 1.31 – 1.23 (m, 1H), 1.03 – 0.93 (m, 1H), 0.87 (s, 3H); **^{13}C NMR (101 MHz, CDCl$_3$)** δ 158.02, 139.11, 127.84, 112.51, 77.84, 55.17, 50.72, 34.71, 34.22, 26.09, 24.85, 24.64, 23.82; **HRMS-ESI:** Found $[M+Na]^+$ = 257.1521; $C_{15}H_{22}O_2Na$ requires 257.1512.

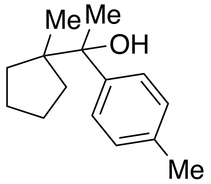

1-(1-Methylcyclopentyl)-1-(*p*-tolyl)ethan-1-ol (1b): (Synthesized according to GP-5, 0.7 mmol scale, yield = 74%, colorless oil): **^{1}H NMR (400 MHz, CDCl$_3$)** δ 7.36 (d, *J* = 8.2 Hz, 2H), 7.11 (d, *J* = 8.0 Hz, 2H), 2.33 (s, 3H), 2.04 – 1.82 (m, 2H), 1.62 (s, 1H), 1.60 (s, 3H), 1.60 – 1.56 (m, 2H), 1.56 – 1.54 (m, 2H), 1.31 – 1.24 (m, 1H), 1.01 – 0.94 (m, 1H), 0.87 (s, 3H); **^{13}C NMR (101 MHz, CDCl$_3$)** δ 143.95, 135.80, 127.94, 126.65, 78.00, 50.57, 34.71, 34.21, 26.02, 24.83, 24.63, 23.81, 20.88; **HRMS-ESI:** Found $[M+Na]^+$ = 241.1577; $C_{15}H_{22}ONa$ requires 241.1563.

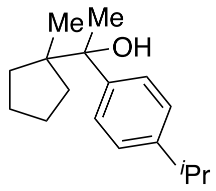

1-(4-Isopropylphenyl)-1-(1-methylcyclopentyl)ethan-1-ol (1c): (Synthesized according to GP-5, 0.65 mmol scale, yield = 79%, colorless oil): **^{1}H NMR (400 MHz, $CDCl_3$)** δ 7.39 (d, J = 8.3 Hz, 2H), 7.16 (d, J = 8.4 Hz, 2H), 2.89 (hept, J = 6.9 Hz, 1H), 2.06 – 1.83 (m, 2H), 1.67 – 1.63 (m, 1H), 1.61 (s, 3H), 1.61 – 1.50 (m, 4H), 1.33 – 1.26 (m, 1H), 1.25 (d, J = 6.8 Hz, 6H), 1.04 – 0.94 (m, 1H), 0.87 (s, 3H); **^{13}C NMR (101 MHz, $CDCl_3$)** δ 146.75, 144.27, 126.66, 125.25, 78.01, 50.61, 34.70, 34.20, 33.53, 26.00, 24.81, 24.62, 23.98, 23.81; **HRMS-ESI:** Found $[M+Na]^+$ = 269.1893; $C_{17}H_{26}ONa$ requires 269.1876.

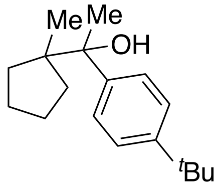

1-(4-(Tert-butyl)phenyl)-1-(1-methylcyclopentyl)ethan-1-ol (1d): (Synthesized according to GP-5, 0.53 mmol scale, yield = 73%, colorless solid): **^{1}H NMR (400 MHz, $CDCl_3$)** δ 7.39 (d, J = 8.6 Hz, 2H), 7.31 (d, J = 8.6 Hz, 2H), 2.01 – 1.87 (m, 2H), 1.63 (s, 1H), 1.61 – 1.55 (m, 4H), 1.55 (s, 3H), 1.32 (s, 9H), 1.30 – 1.25 (m, 1H), 1.04 – 0.94 (m, 1H), 0.87 (s, 3H); **^{13}C NMR (101 MHz, $CDCl_3$)** δ 149.02, 143.87, 126.40, 124.10, 77.98, 50.61, 34.70, 34.29, 34.20, 31.36, 25.97, 24.82, 24.62, 23.82; **HRMS-ESI:** Found $[M+Na]^+$ = 283.2041; $C_{18}H_{28}ONa$ requires 283.2032.

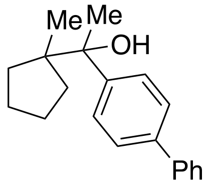

1-([1,1'-Biphenyl]-4-yl)-1-(1-methylcyclopentyl)ethan-1-ol (1e): (Synthesized according to GP-5, 0.57 mmol scale, yield = 84%, colorless oil): **^{1}H NMR (400 MHz, $CDCl_3$)** δ 7.65 – 7.57 (m, 2H), 7.57 – 7.51 (m, 4H), 7.44 (t, J = 7.6 Hz, 2H), 7.37 – 7.30 (m, 1H), 2.07 – 1.88 (m, 2H), 1.69 (s, 1H), 1.66 (s, 3H), 1.66 – 1.55 (m, 4H), 1.38 – 1.28 (m, 1H), 1.08 – 0.99 (m, 1H), 0.92 (s, 3H); **^{13}C NMR (101 MHz, $CDCl_3$)** δ 146.00, 140.82, 139.06, 128.71, 127.21, 127.12, 126.99, 125.91, 78.07, 50.67, 34.75, 34.22, 26.01, 24.83, 24.64, 23.82; **HRMS-ESI:** Found $[M+Na]^+$ = 303.1728; $C_{20}H_{24}ONa$ requires 303.1719.

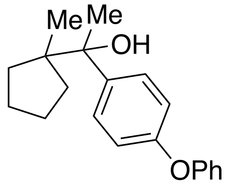

1-(1-Methylcyclopentyl)-1-(4-phenoxyphenyl)ethan-1-ol (1f): (Synthesized according to GP-5, 0.54 mmol scale, yield = 71%, colorless oil): **^{1}H NMR (400 MHz, CDCl$_3$)** δ 7.44 (d, *J* = 8.6 Hz, 2H), 7.33 (dd, *J* = 8.4, 7.0 Hz, 2H), 7.10 (td, *J* = 7.4, 1.2 Hz, 1H), 7.01 (d, *J* = 8.4 Hz, 2H), 6.94 (d, *J* = 8.6 Hz, 2H), 2.05 – 1.80 (m, 2H), 1.65 (s, 1H), 1.62 (s, 3H), 1.62 – 1.44 (m, 4H), 1.33 – 1.26 (m, 1H), 1.06 – 0.96 (m, 1H), 0.89 (s, 3H); **^{13}C NMR (101 MHz, CDCl$_3$)** δ 157.31, 155.61, 141.76, 129.68, 128.13, 123.10, 118.78, 117.51, 77.93, 50.69, 34.72, 34.21, 26.06, 24.85, 24.63, 23.82; **HRMS-ESI:** Found [M+Na]$^+$ = 319.1694; $C_{20}H_{24}O_2Na$ requires 319.1669.

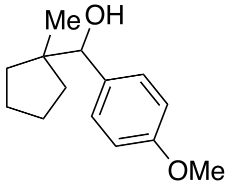

(4-Methoxyphenyl)(1-methylcyclopentyl)methanol (1g): (Synthesized according to GP-6, 1.37 mmol scale, yield = 61%, colorless oil): **^{1}H NMR (400 MHz, CDCl$_3$)** δ 7.28 – 7.25 (m, 2H), 6.89 – 6.81 (m, 2H), 4.48 (d, *J* = 2.6 Hz, 1H), 3.80 (s, 3H), 1.81 (d, *J* = 2.7 Hz, 1H), 1.80 – 1.72 (m, 1H), 1.72 – 1.59 (m, 5H), 1.46 – 1.34 (m, 1H), 1.18 – 1.05 (m, 1H), 0.87 (s, 3H); **^{13}C NMR (101 MHz, CDCl$_3$)** δ 158.77, 135.04, 128.34, 113.04, 81.10, 55.21, 47.89, 36.68, 36.09, 24.46, 24.34, 21.66; **HRMS-ESI:** Found [M+Na]$^+$ = 243.1364; $C_{14}H_{20}O_2Na$ requires 243.1356.

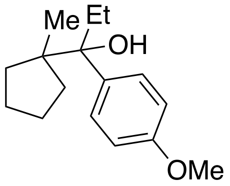

1-(4-Methoxyphenyl)-1-(1-methylcyclopentyl)propan-1-ol (1h): (Synthesized according to GP-5, 1.15 mmol scale, yield = 52%, colorless oil): **^{1}H NMR (400 MHz, CDCl$_3$)** δ 7.37 – 7.28 (m, 2H), 6.89 – 6.79 (m, 2H), 3.81 (s, 3H), 2.30 – 2.18 (m, 1H), 1.98 – 1.84 (m, 2H), 1.84 – 1.75 (m, 1H), 1.66 (s, 1H), 1.64 – 1.55 (m, 3H), 1.54 – 1.50 (m, 1H), 1.31 – 1.23 (m, 1H), 0.90 (dd, *J* = 7.1, 4.0 Hz, 1H), 0.87 (s, 3H), 0.71 (t, *J* = 7.3 Hz, 3H); **^{13}C NMR (101 MHz, CDCl$_3$)** δ 157.86, 135.79, 128.46, 112.53, 80.55, 55.12, 51.17, 34.59, 34.50, 28.00, 24.94, 24.52, 23.85, 7.91; **HRMS-ESI:** Found [M+Na]$^+$ = 271.1682; $C_{16}H_{24}O_2Na$ requires 271.1669.

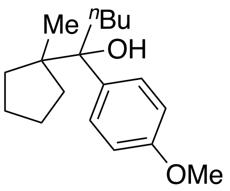

1-(4-Methoxyphenyl)-1-(1-methylcyclopentyl)pentan-1-ol (1i): (Synthesized according to GP-5, 1.0 mmol scale, yield = 50%, colorless oil): **^{1}H NMR (400 MHz, CDCl$_3$)** δ 7.35 – 7.28 (m, 2H), 6.88 – 6.80 (m, 2H), 3.81 (s, 3H), 2.17 (ddd, *J* = 14.0, 12.1, 4.2 Hz, 1H), 1.99 – 1.82 (m, 2H), 1.82 – 1.70 (m, 1H), 1.67 (s, 1H), 1.65 – 1.55 (m, 3H), 1.54 – 1.47 (m, 1H), 1.35 – 1.24 (m, 3H), 1.22 – 1.11 (m, 1H),

1.04 – 0.87 (m, 2H), 0.87 (s, 3H), 0.84 (t, *J* = 7.3 Hz, 3H); **^{13}C NMR (101 MHz, CDCl$_3$)** δ 157.83, 136.32, 128.29, 112.49, 80.31, 55.10, 51.20, 35.53, 34.61, 34.43, 25.91, 24.94, 24.53, 23.84, 23.29, 14.10; **HRMS-ESI:** Found [M+Na]$^+$ = 299.1992; $C_{18}H_{28}O_2Na$ requires 299.1982.

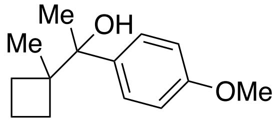

1-(4-Methoxyphenyl)-1-(1-methylcyclobutyl)ethan-1-ol (1j): (Synthesized according to GP-5, 0.74 mmol scale, yield = 55%, colorless oil): **^{1}H NMR (400 MHz, CDCl$_3$)** δ 7.37 – 7.28 (m, 2H), 6.89 – 6.76 (m, 2H), 3.80 (s, 3H), 2.65 – 2.50 (m, 1H), 2.47 – 2.34 (m, 1H), 1.95 – 1.79 (m, 1H), 1.72 – 1.62 (m, 2H), 1.56 – 1.50 (m, 1H), 1.49 (s, 3H), 1.32 – 1.21 (m, 1H), 1.00 (s, 3H); **^{13}C NMR (101 MHz, CDCl$_3$)** δ 158.18, 138.08, 127.18, 112.89, 76.47, 55.17, 46.47, 28.66, 27.60, 23.67, 23.39, 13.73; **HRMS-ESI:** Found [M+Na]$^+$ = 243.1377; $C_{14}H_{20}O_2Na$ requires 243.1356.

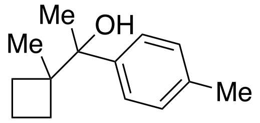

1-(1-Methylcyclobutyl)-1-(*p*-tolyl)ethan-1-ol (1k): (Synthesized according to GP-5, 1.38 mmol scale, yield = 68%, colorless oil): **^{1}H NMR (400 MHz, CDCl$_3$)** δ 7.30 (d, *J* = 8.3 Hz, 2H), 7.12 (d, *J* = 8.0 Hz, 2H), 2.58 (q, *J* = 9.9 Hz, 1H), 2.42 (q, *J* = 9.9 Hz, 1H), 2.33 (s, 3H), 1.95 – 1.80 (m, 1H), 1.74 – 1.61 (m, 2H), 1.57 – 1.51 (m, 1H), 1.50 (s, 3H), 1.32 – 1.21 (m, 1H), 1.01 (s, 3H); **^{13}C NMR (101 MHz, CDCl$_3$)** δ 142.91, 136.01, 128.30, 125.97, 76.66, 46.34, 28.70, 27.59, 23.69, 23.39, 20.91, 13.76; **HRMS-ESI:** Found [M+Na]$^+$ = 227.1417; $C_{14}H_{20}ONa$ requires 227.1406.

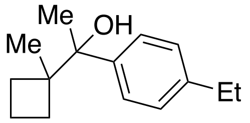

1-(4-Ethylphenyl)-1-(1-methylcyclobutyl)ethan-1-ol (1l): (Synthesized according to GP-5, 1.28 mmol scale, yield = 70%, colorless oil): **^{1}H NMR (400 MHz, CDCl$_3$)** δ 7.32 (d, *J* = 8.3 Hz, 2H), 7.14 (d, *J* = 8.0 Hz, 2H), 2.69 – 2.53 (m, 3H), 2.43 (q, *J* = 9.9 Hz, 1H), 1.96 – 1.80 (m, 1H), 1.73 – 1.61 (m, 2H), 1.57 – 1.51 (m, 1H), 1.50 (s, 3H), 1.33 – 1.26 (m, 1H), 1.24 (t, *J* = 7.6 Hz, 3H), 1.01 (s, 3H); **^{13}C NMR (101 MHz, CDCl$_3$)** δ 143.14, 142.35, 127.07, 126.02, 46.37, 28.70, 28.31, 27.61, 23.65, 23.40, 15.45, 13.78 (the benzylic quaternary carbon is not detected, because it is merged with the $CDCl_3$ peak); **HRMS-ESI:** Found [M+Na]$^+$ = 241.1586; $C_{15}H_{22}ONa$ requires 241.1563.

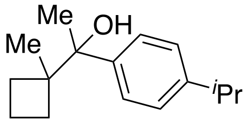

1-(4-Isopropylphenyl)-1-(1-methylcyclobutyl)ethan-1-ol (1m): (Synthesized according to GP-5, 0.7 mmol scale, yield = 71%, colorless oil): **^{1}H NMR (400 MHz, CDCl$_3$)** δ 7.33 (d, *J* = 8.3 Hz, 2H), 7.16 (d, *J* = 8.4 Hz, 2H), 2.89 (hept, *J* = 6.9 Hz, 1H), 2.59 (q, *J* = 9.9 Hz, 1H), 2.43 (q, *J* = 9.8 Hz, 1H), 1.96

– 1.79 (m, 1H), 1.73 – 1.62 (m, 2H), 1.56 – 1.51 (m, 1H), 1.50 (s, 3H), 1.32 – 1.26 (m, 1H), 1.25 (d, *J* = 7.0 Hz, 6H), 1.01 (s, 3H); **^{13}C NMR (101 MHz, CDCl₃)** δ 146.96, 143.24, 125.99, 125.62, 46.40, 33.56, 28.71, 27.63, 23.98, 23.96, 23.60, 23.42, 13.79. (the benzylic quaternary carbon is not detected, because it is merged with the $CDCl_3$ peak); **HRMS-ESI:** Found $[M+Na]^+$ = 255.1704; $C_{16}H_{24}ONa$ requires 255.1719.

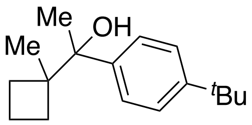

1-(4-(Tert-butyl)phenyl)-1-(1-methylcyclobutyl)ethan-1-ol (1n): (Synthesized according to GP-5, 0.65 mmol scale, yield = 57%, colorless solid): **^{1}H NMR (400 MHz, $CDCl_3$)** δ 7.38 – 7.27 (m, 4H), 2.60 (q, *J* = 9.9 Hz, 1H), 2.43 (q, *J* = 9.8 Hz, 1H), 1.96 – 1.80 (m, 1H), 1.74 – 1.62 (m, 2H), 1.58 – 1.52 (m, 1H), 1.50 (s, 3H), 1.31 (s, 9H), 1.30 – 1.22 (m, 1H), 1.01 (s, 3H); **^{13}C NMR (101 MHz, $CDCl_3$)** δ 149.22, 142.84, 125.73, 124.47, 76.62, 46.40, 34.31, 31.35, 28.71, 27.64, 23.56, 23.43, 13.80; **HRMS-ESI:** Found $[M+Na]^+$ = 269.1899; $C_{17}H_{26}ONa$ requires 269.1876.

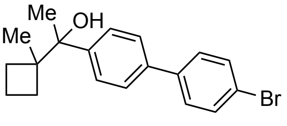

1-(4'-bromo-[1,1'-biphenyl]-4-yl)-1-(1-methylcyclobutyl)ethan-1-ol (1o): (Synthesized according to GP-5, 1.5 mmol scale, yield = 55%, colorless solid): **^{1}H NMR (400 MHz, $CDCl_3$)** δ 7.62 – 7.52 (m, 2H), 7.51 – 7.41 (m, 4H), 7.35 – 7.23 (m, 2H), 2.61 (q, *J* = 9.7 Hz, 1H), 2.45 (q, *J* = 9.8 Hz, 1H), 1.97 – 1.84 (m, 1H), 1.80 – 1.56 (m, 3H), 1.55 (s, 3H), 1.36 – 1.26 (m, 1H), 1.06 (s, 3H).

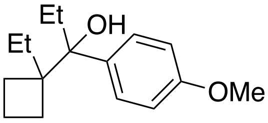

1-(1-Ethylcyclobutyl)-1-(4-methoxyphenyl)propan-1-ol (1p): (Synthesized according to GP-5, 0.46 mmol scale, yield = 79%, colorless oil): **^{1}H NMR (400 MHz, $CDCl_3$)** δ 7.27 (d, *J* = 6.8 Hz, 2H), 6.84 (d, *J* = 8.8 Hz, 2H), 3.80 (s, 3H), 2.53 – 2.31 (m, 2H), 2.10 (dq, *J* = 14.7, 7.4 Hz, 1H), 1.84 – 1.65 (m, 5H), 1.53 – 1.42 (m, 1H), 1.33 (dq, *J* = 14.8, 7.5 Hz, 1H), 1.19 (dq, *J* = 14.3, 7.4 Hz, 1H), 0.96 (t, *J* = 7.5 Hz, 3H), 0.75 (t, *J* = 7.4 Hz, 3H); **^{13}C NMR (101 MHz, $CDCl_3$)** δ 157.97, 135.47, 127.80, 112.79, 79.80, 55.12, 49.64, 28.27, 27.24, 25.38, 23.76, 14.27, 9.50, 7.75; **HRMS-ESI:** Found $[M+Na]^+$ = 271.1666; $C_{16}H_{24}O_2Na$ requires 271.1669.

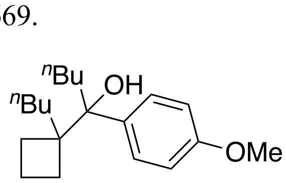

1-(1-Butylcyclobutyl)-1-(4-methoxyphenyl)pentan-1-ol (1q): (Synthesized according to GP-5, 0.46 mmol scale, yield = 81%, colorless oil): **^{1}H NMR (400 MHz, $CDCl_3$)** δ 7.27 (d, *J* = 6.4 Hz, 2H), 6.84 (d, *J* = 8.8 Hz, 2H), 3.81 (s, 3H), 2.53 – 2.32 (m, 2H), 2.03 (ddd, *J* = 13.9, 11.7, 4.1 Hz, 1H), 1.78 –

1.62 (m, 5H), 1.49 – 1.09 (m, 10H), 1.04 – 0.93 (m, 1H), 0.86 (td, J = 7.2, 1.6 Hz, 6H); **^{13}C NMR (101 MHz, $CDCl_3$)** δ 157.93, 135.90, 127.71, 112.76, 79.58, 55.11, 49.45, 35.96, 34.65, 27.38, 26.02, 25.74, 24.39, 23.93, 23.32, 14.24, 14.19, 14.13; **HRMS-ESI:** Found $[M+Na]^+$ = 327.2314; $C_{20}H_{32}O_2Na$ requires 327.2295.

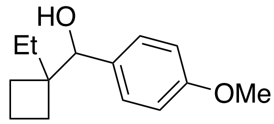

(1-Ethylcyclobutyl)(4-methoxyphenyl)methanol (1r): (Synthesized according to GP-6, 0.92 mmol scale, yield = 83%, colorless oil): **^{1}H NMR (400 MHz, $CDCl_3$)** δ 7.27 (dd, *J* = 2.2, 0.6 Hz, 1H), 7.26 – 7.24 (m, 1H), 6.89 – 6.83 (m, 2H), 4.55 (d, *J* = 2.7 Hz, 1H), 3.81 (s, 3H), 2.33 – 2.18 (m, 2H), 1.86 – 1.75 (m, 1H), 1.74 (d, *J* = 3.2 Hz, 1H), 1.72 – 1.55 (m, 4H), 1.26 (dq, *J* = 14.4, 7.4 Hz, 1H), 0.91 (t, *J* = 7.4 Hz, 3H); **^{13}C NMR (101 MHz, $CDCl_3$)** δ 158.87, 134.47, 128.12, 113.34, 77.39, 55.21, 46.50, 28.50, 26.21, 26.15, 14.88, 8.47; **HRMS-ESI:** Found $[M+Na]^+$ = 243.1370; $C_{14}H_{20}O_2Na$ requires 243.1356.

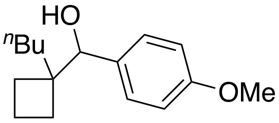

(1-Butylcyclobutyl)(4-methoxyphenyl)methanol (1s): (Synthesized according to GP-6, 0.81 mmol scale, yield = 76%, colorless oil): **^{1}H NMR (400 MHz, $CDCl_3$)** δ 7.28 – 7.26 (m, 1H), 7.26 – 7.23 (m, 1H), 6.89 – 6.81 (m, 2H), 4.54 (d, *J* = 2.7 Hz, 1H), 3.81 (s, 3H), 2.32 – 2.19 (m, 2H), 1.85 – 1.76 (m, 1H), 1.74 (d, *J* = 3.2 Hz, 1H), 1.71 – 1.59 (m, 3H), 1.54 – 1.44 (m, 1H), 1.41 – 1.19 (m, 5H), 0.88 (t, *J* = 7.1 Hz, 3H); **^{13}C NMR (101 MHz, $CDCl_3$)** δ 158.86, 134.48, 128.13, 113.32, 77.63, 55.21, 46.24, 36.09, 26.79, 26.74, 26.35, 23.61, 14.95, 14.13; **HRMS-ESI:** Found $[M+Na]^+$ = 271.1672; $C_{16}H_{24}O_2Na$ requires 271.1669.

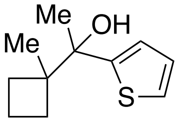

1-(1-Methylcyclobutyl)-1-(thiophen-2-yl)ethan-1-ol (1t): (Synthesized according to GP-5, 0.78 mmol scale, yield = 62%, colorless oil): **^{1}H NMR (400 MHz, $CDCl_3$)** δ 7.19 (dd, *J* = 5.1, 1.2 Hz, 1H), 6.94 (dd, *J* = 5.1, 3.6 Hz, 1H), 6.89 (dd, *J* = 3.6, 1.2 Hz, 1H), 2.62 (q, *J* = 9.9 Hz, 1H), 2.43 (q, *J* = 9.9 Hz, 1H), 1.97 (s, 1H), 1.95 – 1.83 (m, 1H), 1.75 – 1.65 (m, 1H), 1.59 – 1.54 (m, 1H), 1.54 (s, 3H), 1.44 – 1.36 (m, 1H), 1.07 (s, 3H); **^{13}C NMR (101 MHz, $CDCl_3$)** δ 150.56, 126.17, 123.97, 123.52, 76.30, 46.41, 28.15, 27.76, 24.33, 23.44, 13.50; **HRMS-ESI:** Found $[M+Na]^+$ = 219.0819; $C_{11}H_{16}SONa$ requires 219.0814.

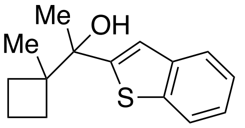

1-(Benzo[b]thiophen-2-yl)-1-(1-methylcyclobutyl)ethan-1-ol (1u): (Synthesized according to GP-5, 0.43 mmol scale, yield = 64%, colorless solid): **^{1}H NMR (400 MHz, CDCl$_3$)** δ 7.78 (d, *J* = 7.7 Hz, 1H), 7.71 (dd, *J* = 7.6, 1.5 Hz, 1H), 7.36 – 7.26 (m, 2H), 7.11 (s, 1H), 2.67 (q, *J* = 9.9 Hz, 1H), 2.47 (q, *J* = 9.9 Hz, 1H), 2.05 (s, 1H), 1.99 – 1.89 (m, 1H), 1.80 – 1.70 (m, 1H), 1.61 (s, 3H), 1.60 – 1.55 (m, 1H), 1.50 – 1.43 (m, 1H), 1.14 (s, 3H); **^{13}C NMR (101 MHz, CDCl$_3$)** δ 151.45, 139.47, 139.44, 124.03, 123.81, 123.19, 122.05, 120.07, 76.57, 46.29, 28.30, 27.88, 24.21, 23.34, 13.74; **HRMS-ESI:** Found [M+Na]$^+$ = 269.0964; $C_{15}H_{18}SONa$ requires 269.0971.

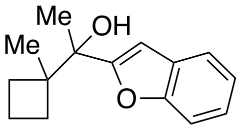

1-(Benzofuran-2-yl)-1-(1-methylcyclobutyl)ethan-1-ol (1v): (Synthesized according to GP-5, 0.28 mmol scale, yield = 88%, colorless solid): **^{1}H NMR (400 MHz, CDCl$_3$)** δ 7.32 (dd, *J* = 7.7, 1.7 Hz, 1H), 7.26 – 7.21 (m, 1H), 6.95 (dd, *J* = 8.3, 1.1 Hz, 1H), 6.86 (td, *J* = 7.5, 1.1 Hz, 1H), 5.79 (s, 1H), 2.43 – 2.31 (m, 2H), 2.10 (s, 1H), 2.03 – 1.91 (m, 1H), 1.84 – 1.74 (m, 1H), 1.69 – 1.59 (m, 2H), 1.46 (s, 3H), 1.34 (s, 3H); **^{13}C NMR (101 MHz, CDCl$_3$)** δ 156.70, 131.74, 130.35, 120.20, 114.85, 109.12, 99.26, 78.19, 73.45, 45.49, 28.71, 27.78, 24.06, 23.03, 13.56; **HRMS-ESI:** Found [M+Na]$^+$ = 253.1205; $C_{15}H_{18}O_2Na$ requires 253.1199.

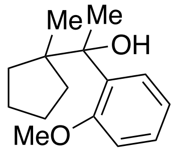

1-(2-Methoxyphenyl)-1-(1-methylcyclopentyl)ethan-1-ol (1w): (Synthesized according to GP-5, 0.60 mmol scale, yield = 73%, colorless oil): **^{1}H NMR (400 MHz, CDCl$_3$)** δ 7.26 – 7.18 (m, 2H), 7.00 – 6.86 (m, 2H), 5.26 (s, 1H), 3.87 (s, 3H), 1.94 – 1.75 (m, 2H), 1.67 – 1.60 (m, 1H), 1.59 (s, 3H), 1.58 – 1.55 (m, 3H), 1.26 (td, *J* = 8.4, 2.4 Hz, 1H), 1.11 – 1.00 (m, 1H), 0.88 (s, 3H); **^{13}C NMR (101 MHz, CDCl$_3$)** δ 157.19, 133.43, 130.14, 127.80, 120.52, 111.86, 80.64, 55.60, 52.46, 34.25, 34.03, 25.75, 24.07, 23.79, 22.75; **HRMS-ESI:** Found [M+Na]$^+$ = 257.1520; $C_{15}H_{22}O_2Na$ requires 257.1512.

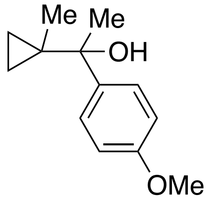

1-(4-Methoxyphenyl)-1-(1-methylcyclopropyl)ethan-1-ol (1x): (Synthesized according to GP-5, 0.63 mmol scale, yield = 60%, colorless oil): **^{1}H NMR (400 MHz, CDCl$_3$)** δ 7.49 – 7.37 (m, 2H), 6.93 – 6.79 (m, 2H), 3.81 (s, 3H), 1.57 (s, 1H), 1.49 (s, 3H), 0.90 (s, 3H), 0.90 – 0.83 (m, 1H), 0.80 – 0.71 (m, 1H), 0.33 (ddd, *J* = 9.4, 5.4, 4.0 Hz, 1H), 0.25 (ddd, *J* = 9.2, 5.5, 4.1 Hz, 1H); **^{13}C NMR (101 MHz, CDCl$_3$)** δ 158.27, 139.29, 126.93, 113.14, 74.95, 55.20, 26.52, 24.63, 21.60, 10.73, 9.95; **HRMS-ESI:** Found [M+Na]$^+$ = 229.1217; $C_{13}H_{18}O_2Na$ requires 229.1199.

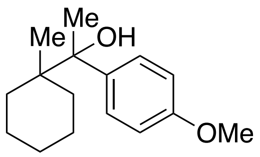

1-(4-Methoxyphenyl)-1-(1-methylcyclohexyl)ethan-1-ol (1y): (Synthesized according to GP-5, 0.86 mmol scale, yield = 81%, colorless oil): **^{1}H NMR (400 MHz, CDCl$_3$)** δ 7.37 – 7.30 (m, 2H), 6.87 – 6.79 (m, 2H), 3.80 (s, 3H), 1.58 (d, *J* = 1.4 Hz, 2H), 1.56 (s, 3H), 1.54 – 1.39 (m, 5H), 1.38 – 1.25 (m, 2H), 1.23 – 1.14 (m, 1H), 1.07 – 0.91 (m, 1H), 0.89 (s, 3H); **^{13}C NMR (101 MHz, CDCl$_3$)** δ 157.98, 138.21, 128.52, 112.23, 79.01, 55.17, 40.33, 31.17, 31.11, 26.03, 24.77, 22.15, 22.05, 17.52; **HRMS-ESI:** Found [M+Na]$^+$ = 271.1662; $C_{16}H_{24}O_2Na$ requires 271.1669.

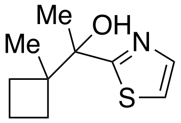

1-(1-Methylcyclobutyl)-1-(thiazol-2-yl)ethan-1-ol (1z): (Synthesized according to GP-5, 0.81 mmol scale, yield = 78%, colorless solid): **^{1}H NMR (400 MHz, CDCl$_3$)** δ 7.71 (d, *J* = 3.2 Hz, 1H), 7.27 (s, 1H), 3.03 (s, 1H), 2.51 (q, *J* = 9.9 Hz, 1H), 2.41 (q, *J* = 9.8 Hz, 1H), 1.96 – 1.84 (m, 1H), 1.76 – 1.60 (m, 2H), 1.59 (s, 3H), 1.40 – 1.32 (m, 1H), 1.15 (s, 3H); **^{13}C NMR (101 MHz, CDCl$_3$)** δ 176.08, 141.53, 118.97, 78.01, 45.88, 28.47, 27.55, 23.68, 23.24, 13.66; **HRMS-ESI:** Found [M+Na]$^+$ = 220.0787; $C_{10}H_{15}ONSNa$ requires 220.0767.

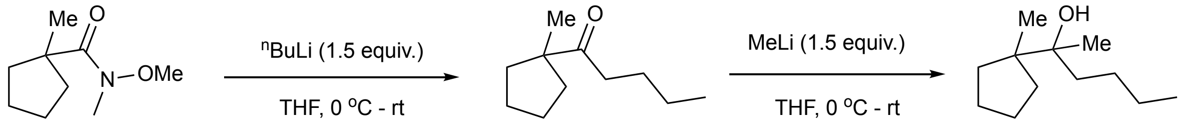

S9 **S10** **1''a**

2-(1-Methylcyclopentyl)hexan-2-ol (1''a): To an oven dried 50 mL round-bottom (RB) flask equipped with a magnetic stir bar, capped with a Suba seal was added Weinreb amide **S9** (2.0 mmol, 1.0 equiv.). The flask containing **S9** was evacuated under vacuum and back-filled with nitrogen three times. Freshly prepared dry THF (10 mL) was added and the solution was cooled down to 0 ºC in an ice bath. After 5 minutes, *ⁿ*BuLi (1.5 equiv., 3.0 mmol) was added dropwise under nitrogen atmosphere. The progress of the reaction was monitored by TLC. Upon completion, the reaction was quenched with saturated aqueous NH_4Cl. The organic layers separated and the aqueous layer was extracted three times with diethyl ether (3x10 mL). Dried over anhydrous sodium sulphate and evaporated under reduced pressure. The crude residue was purified by silica gel flash column chromatography and provides the desired ketone **S10**.

To an oven dried 50 mL round-bottom (RB) flask equipped with a magnetic stir bar, capped with a Suba seal was added ketone **S10** (1.0 mmol). The flask containing starting material **S10** was evacuated under vacuum and back-filled with nitrogen three times. Dry THF (5 mL) was added in to the flask under nitrogen atmosphere and was cooled down to 0 ºC in an ice bath. Then, methyl lithium (1.5 equiv., 1.5

mmol) was added dropwise manner at 0 °C. The progress of the reaction was monitored by TLC. Upon completion, the reaction was quenched with saturated aqueous NH_4Cl. The organic layers separated and the aqueous layer was extracted three times with diethyl ether (3x10 mL). Dried over anhydrous sodium sulphate and evaporated under reduced pressure. The crude product was purified by silica gel flash column chromatography and provides the desired compound **1''a** in 68% yield as colorless oil. **^{1}H NMR (400 MHz, $CDCl_3$)** δ 1.84 – 1.72 (m, 2H), 1.69 – 1.56 (m, 4H), 1.52 – 1.38 (m, 3H), 1.37 – 1.28 (m, 3H), 1.26 – 1.18 (m, 2H), 1.13 (s, 4H), 0.95 (s, 3H), 0.92 (t, *J* = 7.0 Hz, 3H); **^{13}C NMR (101 MHz, $CDCl_3$)** δ 76.38, 50.78, 37.18, 34.80, 34.34, 25.98, 25.65, 25.49, 24.32, 23.56, 22.20, 14.24; **HRMS-ESI:** Found $[M+Na]^+$ = 207.1708; $C_{12}H_{24}ONa$ requires 207.1719.

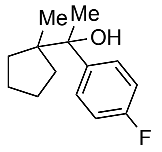

1-(4-fluorophenyl)-1-(1-methylcyclopentyl)ethan-1-ol (1''b): (Synthesized according to GP-5, 0.73 mmol scale, yield = 78%, colorless oil): **^{1}H NMR (400 MHz, $CDCl_3$)** δ 7.50 – 7.36 (m, 2H), 6.98 (t, *J* = 8.7 Hz, 2H), 2.00 – 1.78 (m, 2H), 1.66 (dd, *J* = 7.6, 3.7 Hz, 1H), 1.63 (s, 1H), 1.61 (s, 3H), 1.56 (d, *J* = 15.1 Hz, 3H), 1.35 – 1.19 (m, 1H), 1.02 – 0.90 (m, 1H), 0.86 (s, 3H).

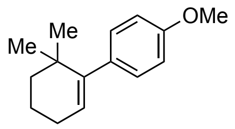

4'-methoxy-2,2-dimethyl-2,3,4,5-tetrahydro-1,1'-biphenyl (1ad)：（synthesized according to H_2 actived Iridium-catalyst experiment, 0.1 mmol scale, yield = 90%, colorless oil）：**^{1}H NMR (400 MHz, $CDCl_3$)** δ 7.06 – 7.04 (m, 2H), 6.82 – 6.80 (m, 2H), 5.40 (t, *J* = 3.8 Hz, 1H), 3.80 (s, 3H), 2.11 – 2.07 (m, 2H), 1.73 – 1.68 (m, 2H), 1.59 – 1.56 (m, 2H), 1.01 (s, 6H); **^{13}C NMR (101 MHz, $CDCl_3$)** δ 158.03, 147.24, 136.12, 130.35, 125.98, 112.80, 55.32, 39.92, 34.48, 28.89, 26.34, 19.51.

General Procedure 7 (GP-7): Iridium catalyzed rearrangement and asymmetric hydrogenation

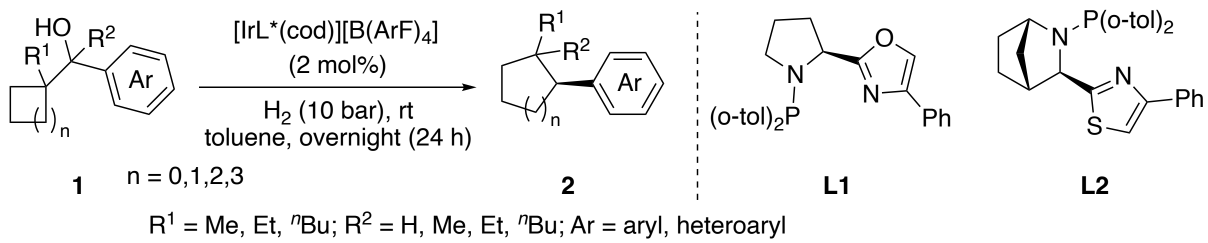

An oven-dried 6 mL glass vial equipped with a magnetic stirring bar was charged with an alcohol starting material **1** (0.1 mmol, 1.0 equiv.) and the Ir-catalyst (2.0 mol%). Freshly prepared dry toluene

(3 mL) was added and the vial was placed in a high-pressure hydrogenation apparatus. The reactor was first purged three times with argon and then three times with H_2 and finally pressurized with H_2 (10 bar). The reaction was stirred at room temperature for 24 h (unless otherwise specified) before the H_2 pressure was released and the solvent was removed under reduced pressure. The residue was purified by flash chromatography (pentane/Et_2O) on silica gel and provides the desired product. The stereoselectivity values (ee/dr) was determined by SFC analysis using a chiral stationary phase. The corresponding racemic product was used for comparison. The racemate was prepared by using racemic Ir-complex (*rac*-**L1**) on a 0.1 mmol scale, following the same asymmetric hydrogenation procedure.

Experimental data of final products:

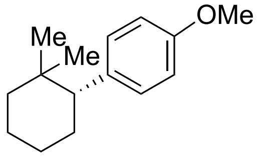

(*R*)-1-(2,2-Dimethylcyclohexyl)-4-methoxybenzene (2a): (Synthesized according to GP-7, 0.1 mmol scale, yield = 99%, colorless oil): **^{1}H NMR (400 MHz, $CDCl_3$)** δ 7.10 – 7.00 (m, 2H), 6.84 – 6.77 (m, 2H), 3.79 (s, 3H), 2.33 (dd, *J* = 12.8, 3.3 Hz, 1H), 1.92 – 1.77 (m, 2H), 1.59 – 1.47 (m, 4H), 1.38 – 1.26 (m, 2H), 0.79 (s, 3H), 0.75 (s, 3H); **^{13}C NMR (101 MHz, $CDCl_3$)** δ 157.69, 136.28, 129.99, 112.74, 55.15, 52.91, 42.17, 34.14, 31.34, 28.68, 27.40, 22.59, 19.57; **GC-APCI-TOF:** Found [m/z] = 218.1670; $C_{15}H_{22}O$ requires 218.1671; **SFC**: ee = >99%, Chiralcel IA column, CO_2/MeOH (96:4), flow: 2.0 mL/min, t_R = 5.44 mins (major); **$[\alpha]_D^{26}$** = +14 (c = 0.56, $CHCl_3$).

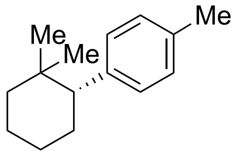

(*R*)-1-(2,2-Dimethylcyclohexyl)-4-methylbenzene (2b): (Synthesized according to GP-7, 0.1 mmol scale, yield = 87%, colorless oil): **^{1}H NMR (400 MHz, $CDCl_3$)** δ 7.06 (d, *J* = 8.0 Hz, 2H), 7.02 (d, *J* = 7.9 Hz, 2H), 2.39 – 2.32 (m, 1H), 2.32 (s, 3H), 1.94 – 1.78 (m, 2H), 1.61 – 1.56 (m, 1H), 1.54 – 1.42 (m, 2H), 1.42 – 1.06 (m, 3H), 0.80 (s, 3H), 0.75 (s, 3H); **^{13}C NMR (101 MHz, $CDCl_3$)** δ 141.06, 135.14, 129.10, 128.08, 53.37, 42.23, 34.06, 31.36, 28.54, 27.36, 22.59, 20.96, 19.63; **GC-APCI-TOF:** Found [m/z] = 202.1716; $C_{15}H_{22}$ requires 202.1722; **SFC**: ee = >99%, Chiralcel OJH column, CO_2/MeOH (95:5), flow: 2.0 mL/min, t_R = 10.15 mins (major); **$[\alpha]_D^{26}$** = +10 (c = 0.56, $CHCl_3$).

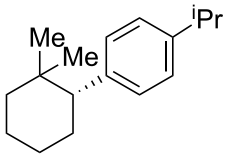

(*R*)-1-(2,2-Dimethylcyclohexyl)-4-isopropylbenzene (2c): (Synthesized according to GP-7, 0.1 mmol scale, yield = 99%, colorless oil): **^{1}H NMR (400 MHz, $CDCl_3$)** δ 7.10 (d, *J* = 8.1 Hz, 2H), 7.04 (d, *J* = 8.1 Hz, 2H), 2.87 (hept, *J* = 6.9 Hz, 1H), 2.34 (dd, *J* = 12.8, 3.1 Hz, 1H), 1.95 – 1.78 (m, 2H), 1.61 –

1.54 (m, 2H), 1.53 – 1.44 (m, 2H), 1.39 – 1.28 (m, 2H), 1.24 (d, *J* = 6.9 Hz, 6H), 0.80 (s, 3H), 0.76 (s, 3H); **^{13}C NMR (101 MHz, $CDCl_3$)** δ 146.08, 141.35, 129.07, 125.33, 53.37, 42.25, 34.11, 33.56, 31.39, 28.48, 27.38, 24.04, 22.60, 19.65; **GC-APCI-TOF:** Found [m/z] = 230.2033; $C_{17}H_{26}$ requires 230.2035; **SFC**: ee = >99%, Chiralcel OJH column, CO_2/MeOH (95:5), flow: 2.0 mL/min, t_R = 4.4 mins (major); **$[\alpha]_D^{26}$** = +12 (c = 0.41, $CHCl_3$).

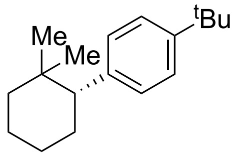

(*R*)-1-(Tert-butyl)-4-(2,2-dimethylcyclohexyl)benzene (2d): (Synthesized according to GP-7, 0.1 mmol scale, yield = 94%, colorless oil): **^{1}H NMR (400 MHz, $CDCl_3$)** δ 7.25 – 7.22 (m, 2H), 7.09 – 7.00 (m, 2H), 2.34 (dd, *J* = 12.8, 3.2 Hz, 1H), 1.93 – 1.79 (m, 2H), 1.62 – 1.55 (m, 2H), 1.54 – 1.45 (m, 2H), 1.36 – 1.33 (m, 1H), 1.31 (s, 9H), 1.29 – 1.22 (m, 1H), 0.80 (s, 3H), 0.76 (s, 3H); **^{13}C NMR (101 MHz, $CDCl_3$)** δ 148.35, 140.94, 128.81, 124.16, 53.25, 42.24, 34.28, 34.12, 31.43, 31.40, 28.44, 27.37, 22.59, 19.66; **GC-APCI-TOF:** Found [m/z] = 244.2186; $C_{18}H_{28}$ requires 244.2191; **SFC**: ee = >99%, Chiralcel OJH column, CO_2/MeOH (95:5), flow: 2.0 mL/min, t_R = 4.29 mins (major); **$[\alpha]_D^{26}$** = +12 (c = 0.39, $CHCl_3$).

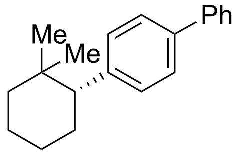

(*R*)-4-(2,2-Dimethylcyclohexyl)-1,1'-biphenyl (2e): (Synthesized according to GP-7, 0.1 mmol scale, yield = 99%, colorless solid): **^{1}H NMR (400 MHz, $CDCl_3$)** δ 7.61 – 7.58 (m, 2H), 7.49 (d, *J* = 8.2 Hz, 2H), 7.42 (t, *J* = 7.7 Hz, 2H), 7.32 (t, *J* = 7.2 Hz, 1H), 7.21 (d, *J* = 8.2 Hz, 2H), 2.43 (dd, *J* = 12.9, 3.2 Hz, 1H), 1.99 – 1.82 (m, 2H), 1.67 – 1.56 (m, 3H), 1.50 (dd, *J* = 3.7, 2.0 Hz, 1H), 1.40 – 1.32 (m, 2H), 0.85 (s, 3H), 0.81 (s, 3H); **^{13}C NMR (101 MHz, $CDCl_3$)** δ 143.28, 141.13, 138.58, 129.62, 128.66, 126.95, 126.90, 126.06, 53.49, 42.23, 34.17, 31.39, 28.46, 27.30, 22.57, 19.68; **GC-APCI-TOF:** Found [m/z] = 264.1882; $C_{20}H_{24}$ requires 264.1878; **SFC**: ee = >99%, Chiralcel ODH column, CO_2/MeOH (95:5), flow: 2.0 mL/min, t_R = 12.72 mins (major); **$[\alpha]_D^{26}$** = +8 (c = 0.32, $CHCl_3$). NB: the reaction was stirred 60 h for completion.

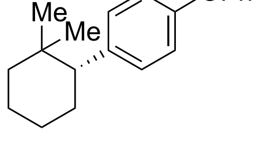

(*R*)-1-(2,2-Dimethylcyclohexyl)-4-phenoxybenzene (2f): (Synthesized according to GP-7, 0.1 mmol scale, yield = 99%, colorless oil): **^{1}H NMR (400 MHz, $CDCl_3$)** δ 7.35 – 7.29 (m, 2H), 7.12 – 7.05 (m, 3H), 7.03 – 6.97 (m, 2H), 6.93 – 6.88 (m, 2H), 2.37 (dd, *J* = 12.8, 3.3 Hz, 1H), 1.90 – 1.78 (m, 2H), 1.63 – 1.56 (m, 2H), 1.54 – 1.46 (m, 2H), 1.38 – 1.27 (m, 2H), 0.81 (s, 3H), 0.77 (s, 3H); **^{13}C NMR**

(101 MHz, $CDCl_3$) δ 157.58, 155.08, 139.09, 130.27, 129.62, 122.86, 118.60, 117.89, 53.11, 42.15, 34.11, 31.35, 28.60, 27.33, 22.55, 19.59; **GC-APCI-TOF:** Found [m/z] = 280.1835; $C_{20}H_{24}O$ requires 280.1827; **SFC**: ee = >99%, Chiralcel ODH column, CO_2/MeOH (95:5), flow: 2.0 mL/min, t_R = 10.93 mins (major); **$[\alpha]_D^{26}$** = +9 (c = 0.37, $CHCl_3$). NB: the reaction was stirred 60 h for completion.

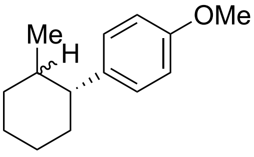

(*1R*)-1-Methoxy-4-(2-methylcyclohexyl)benzene (inseparable mixture of diastereomers; dr = 70:30) (2g): (Synthesized according to GP-7, 0.1 mmol scale, yield = 93%, colorless oil): **^{1}H NMR (400 MHz, $CDCl_3$)** δ 7.10 – 7.05 (m, 2H), 6.86 – 6.81 (m, 2H), 3.79 (s, 3H), 2.77 (dt, *J* = 12.5, 3.8 Hz, 0.70H), 2.07 – 2.03 (m, 0.69H), 2.00 (dd, *J* = 11.1, 3.3 Hz, 0.31H), 1.89 – 1.73 (m, 2.89H), 1.68 – 1.59 (m, 1.70H), 1.52 – 1.50 (m, 0.36H), 1.50 – 1.45 (m, 1.19H), 1.44 – 1.32 (m, 1.68H), 1.13 – 1.00 (m, 0.45H), 0.91 – 0.78 (m, 0.36H), 0.66 (t, *J* = 6.9 Hz, 3H); **^{13}C NMR (101 MHz, $CDCl_3$)** δ 157.61, 157.45, 139.09, 138.51, 128.33, 128.28, 113.55, 113.30, 55.19, 55.17, 51.58, 45.59, 37.90, 35.83, 34.65, 33.47, 27.03, 26.94, 26.70, 24.85, 20.78, 20.22, 12.27; **GC-APCI-TOF:** Found [m/z] = 204.1514; $C_{14}H_{20}O$ requires 204.1514; **SFC**: ee = 78%, dr = 70/30, Chiralcel OZH column, CO_2/MeOH (95:5), flow: 2.0 mL/min; **$[\alpha]_D^{26}$** = +15 (c = 0.66, $CHCl_3$).

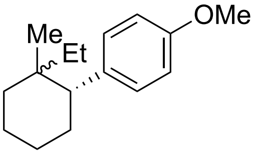

1-((*1R*)-2-Ethyl-2-methylcyclohexyl)-4-methoxybenzene: (inseparable mixture of diastereomers; dr = 1:1) (2h): (Synthesized according to GP-7, 0.1 mmol scale, yield = 92%, colorless oil): **1H NMR (400 MHz, CDCl3)** δ 7.08 – 7.04 (m, 2H), 6.82 – 6.78 (m, 2H), 3.79 (s, 3H), 2.41 (ddd, *J* = 12.8, 7.0, 3.3 Hz, 1H), 2.02 – 1.87 (m, 1H), 1.86 – 1.79 (m, 1H), 1.78 – 1.72 (m, 1H), 1.61 – 1.57 (m, 0.95H), 1.55 – 1.51 (m, 1.19H), 1.42 – 1.19 (m, 3.12H), 1.11 – 1.05 (m, 1.34H), 0.94 – 0.86 (m, 1H), 0.81 (s, 1.50H), 0.76 (t, *J* = 7.5 Hz, 1.54H), 0.72 (s, 1.54H), 0.71 – 0.66 (m, 1.51H); **^{13}C NMR (101 MHz, $CDCl_3$)** δ 157.66, 136.40, 135.84, 130.29, 130.01, 112.77, 112.65, 55.14, 54.33, 51.63, 37.31, 36.50, 36.22, 36.18, 35.29, 29.11, 28.04, 27.32, 27.24, 27.20, 22.42, 22.24, 22.05, 17.56, 7.46, 7.34; **GC-APCI-TOF:** Found [m/z] = 232.1827; $C_{16}H_{24}O$ requires 232.1827; **SFC**: ee = 96%, dr = 50/50, Chiralcel OJH column, CO_2/MeOH (95:5), flow: 2.0 mL/min; **$[\alpha]_D^{26}$** = +6 (c = 0.23, $CHCl_3$).

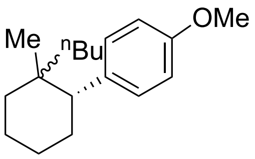

1-((*1R*)-2-Butyl-2-methylcyclohexyl)-4-methoxybenzene: (inseparable mixture of diastereomers; dr = 1:1) (2i): (Synthesized according to GP-7, 0.1 mmol scale, yield = 96%, colorless oil): **^{1}H NMR**

(400 MHz, $CDCl_3$) δ 7.10 – 7.00 (m, 2H), 6.83 – 6.76 (m, 2H), 3.79 (s, 3H), 2.39 (td, J = 12.7, 3.4 Hz, 1H), 1.94 (dtd, *J* = 29.4, 13.0, 4.0 Hz, 1H), 1.86 – 1.77 (m, 1H), 1.75 – 1.65 (m, 1H), 1.60 – 1.55 (m, 1H), 1.52 – 1.47 (m, 1H), 1.46 – 1.12 (m, 6H), 1.12 – 0.93 (m, 3H), 0.86 (t, *J* = 7.5 Hz, 3H), 0.81 (s, 1.5H), 0.73 (s, 1.5H); **^{13}C NMR (101 MHz, $CDCl_3$)** δ 157.65, 157.63, 136.42, 135.86, 130.30, 130.01, 112.76, 112.65, 55.13, 54.48, 51.61, 42.94, 38.07, 37.05, 36.52, 36.14, 29.96, 29.09, 28.06, 27.90, 27.32, 27.25, 25.31, 25.25, 23.83, 23.62, 22.30, 22.13, 18.22, 14.28, 14.24; **GC-APCI-TOF:** Found [m/z] = 260.2146; $C_{18}H_{28}O$ requires 260.2140; **SFC**: ee = >99%, dr = 50/50, Chiralcel OJH column, CO_2/MeOH (95:5), flow: 2.0 mL/min; **$[\alpha]_D^{26}$** = +6 (c = 0.54, $CHCl_3$).

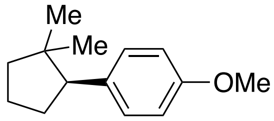

(*S*)-1-(2,2-Dimethylcyclopentyl)-4-methoxybenzene (2j): (Synthesized according to GP-7, 0.1 mmol scale, yield = 99%, colorless oil): **^{1}H NMR (400 MHz, $CDCl_3$):** δ 7.15 – 7.06 (m, 2H), 6.88 – 6.78 (m, 2H), 3.80 (s, 3H), 2.64 (dd, *J* = 10.8, 8.2 Hz, 1H), 2.11 – 1.91 (m, 2H), 1.84 – 1.65 (m, 2H), 1.65 – 1.54 (m, 2H), 0.97 (s, 3H), 0.62 (s, 3H); **^{13}C NMR (101 MHz, $CDCl_3$)** δ 157.86, 134.02, 129.53, 113.05, 55.36, 55.19, 42.08, 41.42, 30.14, 28.43, 22.88, 21.33; **GC-APCI-TOF:** Found [m/z] = 204.1512; $C_{14}H_{20}O$ requires 204.1514; **SFC**: ee = > 99%, Chiralcel ADH column, CO_2/MeOH (95:5), flow: 2.0 mL/min, t_R = 4.14 mins (major); **$[\alpha]_D^{26}$** = - 14 (c = 0.5, $CHCl_3$).

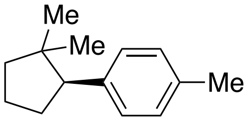

(*S*)-1-(2,2-Dimethylcyclopentyl)-4-methylbenzene (2k): (Synthesized according to GP-7, 0.1 mmol scale, yield = 99%, colorless oil): **^{1}H NMR (400 MHz, $CDCl_3$)** δ 7.14 – 7.04 (m, 4H), 2.66 (dd, *J* = 11.0, 8.0 Hz, 1H), 2.33 (s, 3H), 2.11 – 1.93 (m, 2H), 1.85 – 1.66 (m, 2H), 1.65 – 1.54 (m, 2H), 0.98 (s, 3H), 0.63 (s, 3H); **^{13}C NMR (101 MHz, $CDCl_3$)** δ 138.91, 135.32, 128.62, 128.36, 55.78, 42.12, 41.53, 30.07, 28.48, 22.96, 21.40, 20.97; **GC-APCI-TOF:** Found [m/z] = 188.1562; $C_{14}H_{20}$ requires 188.1565; **SFC**: ee = 97%, Chiralcel OJH column, CO_2/MeOH (95:5), flow: 2.0 mL/min, t_R = 6.20 mins (major) and 7.50 mins (minor); **$[\alpha]_D^{26}$** = - 26 (c = 0.40, $CHCl_3$).

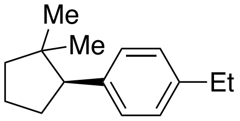

(*S*)-1-(2,2-Dimethylcyclopentyl)-4-ethylbenzene (2l): (Synthesized according to GP-7, 0.1 mmol scale, yield = 99%, colorless oil): **^{1}H NMR (400 MHz, $CDCl_3$)** δ 7.16 – 7.06 (m, 4H), 2.72 – 2.57 (m, 3H), 2.12 – 1.92 (m, 2H), 1.86 – 1.75 (m, 1H), 1.75 – 1.65 (m, 1H), 1.65 – 1.54 (m, 2H), 1.24 (t, *J* = 7.6 Hz, 3H), 0.99 (s, 3H), 0.63 (s, 3H); **^{13}C NMR (101 MHz, $CDCl_3$)** δ 141.70, 139.15, 128.66, 127.10, 55.80, 42.17, 41.55, 30.05, 28.49, 28.38, 22.97, 21.42, 15.54; **GC-APCI-TOF:** Found [m/z] =

202.1715; $C_{15}H_{22}$ requires 202.1722; **SFC**: ee = 94%, Chiralcel OJH column, CO_2/MeOH (95:5), flow: 2.0 mL/min, t_R = 4.67 mins (major) and 5.63 mins (minor); **$[\alpha]_D^{26}$** = - 24 (c = 0.42, $CHCl_3$).

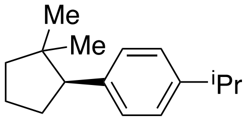

(*S*)-1-(2,2-Dimethylcyclopentyl)-4-isopropylbenzene (2m): (Synthesized according to GP-7, 0.1 mmol scale, yield = 99%, colorless oil): **^{1}H NMR (400 MHz, $CDCl_3$)** δ 7.18 – 7.07 (m, 4H), 2.89 (hept, *J* = 6.9 Hz, 1H), 2.67 (dd, *J* = 11.0, 7.9 Hz, 1H), 2.15 – 1.92 (m, 2H), 1.86 – 1.75 (m, 1H), 1.74 – 1.64 (m, 1H), 1.62 – 1.53 (m, 2H), 1.25 (d, *J* = 6.9 Hz, 6H), 0.99 (s, 3H), 0.63 (s, 3H); **^{13}C NMR (101 MHz, $CDCl_3$)** δ 146.30, 139.25, 128.61, 125.64, 55.77, 42.19, 41.55, 33.61, 30.03, 28.50, 24.05, 24.04, 22.98, 21.42; **GC-APCI-TOF:** Found [m/z] = 216.1875; $C_{16}H_{24}$ requires 216.1878; **SFC**: ee = 97.4%, Chiralcel OJH column, CO_2/MeOH (95:5), flow: 2.0 mL/min, t_R = 4.16 mins (major) and 4.98 mins (minor); **$[\alpha]_D^{26}$** = - 16 (c = 0.37, $CHCl_3$).

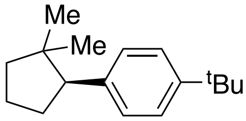

(*S*)-1-(Tert-butyl)-4-(2,2-dimethylcyclopentyl)benzene (2n): (Synthesized according to GP-7, 0.1 mmol scale, yield = 99%, colorless solid): **^{1}H NMR (400 MHz, $CDCl_3$)** δ 7.32 – 7.27 (m, 2H), 7.14 – 7.09 (m, 2H), 2.67 (dd, *J* = 11.0, 7.9 Hz, 1H), 2.12 – 1.93 (m, 2H), 1.86 – 1.66 (m, 2H), 1.65 – 1.54 (m, 2H), 1.32 (s, 9H), 0.99 (s, 3H), 0.63 (s, 3H); **^{13}C NMR (101 MHz, $CDCl_3$)** δ 148.56, 138.85, 128.35, 124.48, 55.66, 42.22, 41.57, 34.30, 31.42, 30.01, 28.51, 23.00, 21.44; **GC-APCI-TOF:** Found [m/z] = 230.2034; $C_{17}H_{26}$ requires 230.2035; **SFC**: ee = 96.2%, Chiralcel OJH column, CO_2/MeOH (95:5), flow: 2.0 mL/min, t_R = 3.71 mins (major) and 4.67 mins (minor); **$[\alpha]_D^{26}$** = - 25 (c = 0.56, $CHCl_3$).

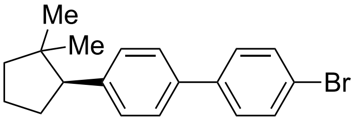

(*S*)-4-Bromo-4'-(2,2-dimethylcyclopentyl)-1,1'-biphenyl (2o): (Synthesized according to GP-7, 0.1 mmol scale, yield = 64%, colorless oil): **^{1}H NMR (400 MHz, $CDCl_3$)** δ 7.63 – 7.40 (m, 5H), 7.29 – 7.26 (m, 1H), 7.25 – 7.20 (m, 2H), 2.79 – 2.69 (m, 1H), 2.19 – 2.06 (m, 1H), 2.09 – 1.97 (m, 1H), 1.89 – 1.79 (m, 1H), 1.79 – 1.69 (m, 1H), 1.68 – 1.57 (m, 2H), 1.03 (s, 3H), 0.68 (s, 3H); **SFC**: ee = > 99%, Chiralcel OZH column, CO_2/MeOH (95:5), flow: 2.0 mL/min, t_R = 7.68 mins (major); **$[\alpha]_D^{26}$** = - 14 (c = 0.53, $CHCl_3$). NB: We couldn't purify the compound **2o** after several attempts-because both the products and and impurities move out from silica column in pure pentane.

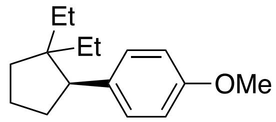

(*S*)-1-(2,2-Diethylcyclopentyl)-4-methoxybenzene (2p): (Synthesized according to GP-7, 0.1 mmol scale, yield = 82%, colorless oil): **^{1}H NMR (400 MHz, $CDCl_3$)** δ 7.09 (d, *J* = 8.7 Hz, 2H), 6.81 (d, *J* = 8.6 Hz, 2H), 3.79 (s, 3H), 2.88 (t, *J* = 8.5 Hz, 1H), 2.11 – 1.99 (m, 1H), 1.96 – 1.87 (m, 1H), 1.81 – 1.71 (m, 2H), 1.71 – 1.58 (m, 2H), 1.51 – 1.41 (m, 1H), 1.36 – 1.22 (m, 2H), 1.09 – 0.98 (m, 1H), 0.88 (t, *J* = 7.4 Hz, 3H), 0.63 (t, *J* = 7.4 Hz, 3H); **^{13}C NMR (101 MHz, $CDCl_3$)** δ 157.64, 135.56, 129.70, 113.05, 55.17, 51.77, 47.98, 35.17, 31.54, 28.76, 27.07, 22.57, 8.72, 8.69; **GC-APCI-TOF:** Found [m/z] = 232.1830; $C_{16}H_{24}O$ requires 232.1827; **SFC**: ee = > 99%, Chiralcel ADH column, CO_2/MeOH (95:5), flow: 2.0 mL/min, t_R = 4.77 mins (major); **$[\alpha]_D^{26}$** = - 11 (c = 1.06, $CHCl_3$).

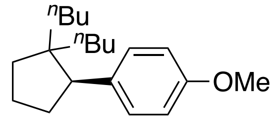

(*S*)-1-(2,2-Dibutylcyclopentyl)-4-methoxybenzene (2q): (Synthesized according to GP-7, 0.1 mmol scale, yield = 85%, colorless oil): **^{1}H NMR (400 MHz, $CDCl_3$)** δ 7.08 (d, *J* = 8.7 Hz, 2H), 6.81 (d, *J* = 8.7 Hz, 2H), 3.80 (s, 3H), 2.85 (t, *J* = 8.5 Hz, 1H), 2.09 – 1.88 (m, 2H), 1.82 – 1.61 (m, 3H), 1.55 – 1.52 (m, 1H), 1.43 – 1.34 (m, 1H), 1.33 – 1.22 (m, 6H), 1.14 – 1.03 (m, 2H), 1.02 – 0.94 (m, 1H), 0.92 (t, *J* = 6.8 Hz, 3 H), 0.84 (q, *J* = 7.2 Hz, 2H), 0.77 (t, *J* = 7.1 Hz, 3H); **^{13}C NMR (101 MHz, $CDCl_3$)** δ 157.64, 135.57, 129.70, 113.04, 55.19, 52.68, 47.73, 37.27, 36.26, 34.74, 31.46, 26.64, 26.60, 23.72, 22.50, 14.23, 14.14; **GC-APCI-TOF:** Found [m/z] = 288.2454; $C_{20}H_{32}O$ requires 288.2453; **SFC**: ee = 98%, Chiralcel OJH column, CO_2/MeOH (95:5), flow: 2.0 mL/min, t_R = 3.62 mins (major) and 4.78 mins (minor); **$[\alpha]_D^{26}$** = - 2 (c = 0.9, $CHCl_3$).

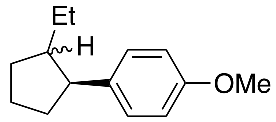

1-((1*S*)-2-Ethylcyclopentyl)-4-methoxybenzene (inseparable mixture of diastereomers; dr = 58:42) (2r): (Synthesized according to GP-7, 0.1 mmol scale, yield = 84%, colorless oil): **^{1}H NMR (400 MHz, $CDCl_3$)** δ 7.17 – 7.02 (m, 2H), 6.88 – 6.74 (m, 2H), 3.79 (s, 3H), 3.13 (q, *J* = 7.4 Hz, 0.59H), 2.44 (q, *J* = 7.4 Hz, 0.42H), 2.08 – 1.91 (m, 2H), 1.89 – 1.79 (m, 1.78H), 1.78 – 1.61 (m, 2.25H), 1.50 – 1.40 (m, 1H), 1.35 – 1.21 (m, 0.59H), 1.17 – 1.05 (m, 0.48H), 1.05 – 0.93 (m, 0.64H), 0.85 – 0.73 (m, 3.61H); **^{13}C NMR (101 MHz, $CDCl_3$)** δ 157.69, 157.52, 137.93, 136.09, 129.35, 128.35, 113.64, 113.19, 55.21, 55.18, 51.99, 49.97, 48.08, 46.33, 35.70, 31.68, 30.81, 30.31, 26.98, 23.94, 23.59, 23.57, 12.94, 12.77; **GC-APCI-TOF:** Found [m/z] = 204.1509; $C_{14}H_{20}O$ requires 204.1514; **SFC**: ee = > 99%, d.r. = 58:42, Chiralcel ADH column, CO_2/MeOH (95:5), flow: 2.0 mL/min; **$[\alpha]_D^{26}$** = - 5 (c = 0.26, $CHCl_3$).

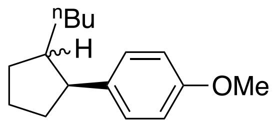

1-((1*S*)-2-Butylcyclopentyl)-4-methoxybenzene (inseparable mixture of diastereomers; dr = 60:40) (2s): (0.1 mmol scale, yield = 83%, colorless oil): **^{1}H NMR (400 MHz, $CDCl_3$)** δ 7.17 – 7.02 (m, 2.09H), 6.87 – 6.77 (m, 2.03H), 3.79 (s, 3H), 3.11 (q, *J* = 7.3 Hz, 0.62H), 2.43 (q, *J* = 9.9 Hz, 0.42H), 2.10 – 1.92 (m, 2.12H), 1.91 – 1.57 (m, 4.34H), 1.50 – 1.33 (m, 1.20H), 1.33 – 1.02 (m, 5.40H), 1.03 – 0.90 (m, 0.70H), 0.85 – 0.73 (m, 3.73H). **^{13}C NMR (101 MHz, $CDCl_3$)** δ 157.68, 157.48, 137.90, 136.14, 129.34, 128.34, 113.64, 113.18, 55.21, 55.18, 52.32, 48.14, 48.09, 44.32, 35.69, 34.10, 32.18, 30.82, 30.80, 30.74, 30.70, 30.29, 23.97, 23.56, 22.96, 22.85, 14.12, 14.08. **GC-APCI-TOF:** Found [m/z] = 232.1830; $C_{16}H_{24}O$ requires 232.1827; **SFC**: ee = 92%, d.r. = 60:40, Chiralcel OJH column, CO_2/MeOH (95:5), flow: 1.0 mL/min; **$[\alpha]_D^{26}$** = - 13 (c = 0.28, $CHCl_3$).

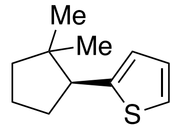

(*S*)-2-(2,2-Dimethylcyclopentyl)thiophene (2t): (Synthesized according to GP-7, 0.1 mmol scale, yield = 66%, colorless oil): **^{1}H NMR (400 MHz, $CDCl_3$)** δ 7.12 (dd, *J* = 5.1, 1.2 Hz, 1H), 6.96 (dd, *J* = 5.1, 3.4 Hz, 1H), 6.80 (dt, *J* = 3.5, 1.0 Hz, 1H), 2.95 (dd, *J* = 11.2, 7.8 Hz, 1H), 2.19 – 2.10 (m, 1H), 2.04 – 1.95 (m, 1H), 1.87 – 1.66 (m, 3H), 1.63 – 1.59 (m, 1H), 1.08 (s, 3H), 0.68 (s, 3H); **^{13}C NMR (101 MHz, $CDCl_3$)** δ 145.88, 126.34, 124.11, 122.44, 51.41, 42.13, 41.28, 32.09, 28.18, 22.76, 21.21. **GC-APCI-TOF:** Found [m/z] = 180.0967; $C_{11}H_{16}S$ requires 180.0973; **SFC**: ee = 50%, Chiralcel ADH column, CO_2/MeOH (96:4), flow: 2.0 mL/min, t_R = 3.34 mins (major) and 3.60 mins (minor); **$[\alpha]_D^{26}$** = -10 (c = 0.09, $CHCl_3$).

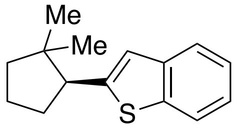

(*S*)-2-(2,2-Dimethylcyclopentyl)benzo[b]thiophene (2u): (Synthesized according to GP-7, 0.1 mmol scale, yield = 65%, colorless oil): **^{1}H NMR (400 MHz, $CDCl_3$)** δ 7.77 (dd, *J* = 7.9, 1.2 Hz, 1H), 7.68 (dd, *J* = 7.5, 1.4 Hz, 1H), 7.30 (td, *J* = 7.5, 1.3 Hz, 1H), 7.24 (dd, *J* = 7.1, 1.3 Hz, 1H), 7.03 (s, 1H), 3.00 (dd, *J* = 10.9, 7.8 Hz, 1H), 2.26 – 2.15 (m, 1H), 2.13 – 2.04 (m, 1H), 1.90 – 1.81 (m, 1H), 1.80 – 1.72 (m, 1H), 1.72 – 1.60 (m, 2H), 1.15 (s, 3H), 0.77 (s, 3H). **^{13}C NMR (101 MHz, $CDCl_3$)** δ 147.09, 139.95, 138.99, 123.88, 123.24, 122.61, 121.90, 120.70, 52.11, 42.53, 41.45, 31.85, 28.39, 23.03, 21.42. **GC-APCI-TOF:** Found [m/z] = 230.1129; $C_{15}H_{18}S$ requires 230.1129; **SFC**: ee = 98%, Chiralcel ADH column, CO_2/MeOH (95:5), flow: 2.0 mL/min, t_R = 7.10 mins (major) and 9.59 mins (minor); **$[\alpha]_D^{26}$** = -13 (c = 0.03, $CHCl_3$).

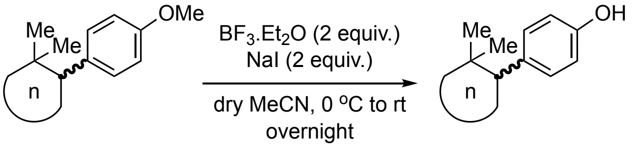

(*R*)-2a (n = 6)
(*S*)-2j (n = 5)

(*R*)-3 (n = 6)
(*S*)-5 (n = 5)

General Procedure 8 (GP-8): Cleavage of methoxy group

Synthesized following the known literature procedure.[4] To a stirred solution of ether **(*R*)-2a/(*S*)-2j** (1.0 mmol) and sodium iodide (2.0 mmol) in dry acetonitrile (5 mL) was added boron trifluoride etherate (2.0 mmol) slowly under nitrogen atmosphere at 0 °C. The mixture was stirred at room temperature for overnight. Upon completion, quenched with saturated sodium hydrogen carbonate solution (5 mL) and extracted with ether (3 × 10 mL). The combined organic layers was washed with aqueous sodium thiosulfate solution (10 mL) followed by water (15 mL), dried with anhydrous sodium sulfate, and evaporated. The residue was purified by silica gel column chromatography to yield the pure alcohol **(*R*)-3/(*S*)-5** as colorless solid.

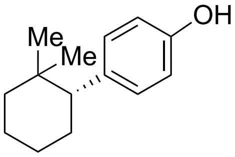

(*R*)-4-(2,2-dimethylcyclohexyl)phenol (3): (Synthesized according to GP-8, 1.0 mmol scale, yield = 65%, colorless solid)**:** **^{1}H NMR (400 MHz, CDCl$_3$)** δ 7.00 (d, *J* = 8.2 Hz, 2H), 6.72 (d, *J* = 8.3 Hz, 2H), 4.49 (s, 1H), 2.31 (dd, *J* = 12.9, 3.3 Hz, 1H), 1.90 – 1.76 (m, 2H), 1.60 – 1.56 (m, 1H), 1.53 – 1.20 (m, 5H), 0.78 (s, 3H), 0.74 (s, 3H); **^{13}C NMR (101 MHz, CDCl$_3$)** δ 153.46, 136.47, 130.18, 114.20, 52.90, 42.14, 34.10, 31.31, 28.65, 27.37, 22.56, 19.54; **HRMS-ESI** [M-H]: Found [m/z] = 203.1439; $C_{14}H_{19}O$ requires 203.1436; **[α]$_D$^{26}** = +13 (c = 0.31, $CHCl_3$).

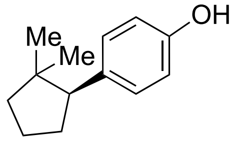

(*S*)-4-(2,2-dimethylcyclopentyl)phenol (5) (Synthesized according to GP-8, 1.0 mmol scale, yield = 43%, colorless solid)**:** **^{1}H NMR (400 MHz, CDCl$_3$)** δ 7.05 (d, *J* = 8.5 Hz, 2H), 6.75 (d, *J* = 8.4 Hz, 2H), 4.52 (s, 1H), 2.63 (dd, *J* = 10.7, 8.3 Hz, 1H), 2.07 – 1.82 (m, 3H), 1.80 – 1.59 (m, 3H), 0.96 (s, 3H), 0.61 (s, 3H); **^{13}C NMR (101 MHz, CDCl$_3$)** δ 153.66, 134.23, 129.72, 114.49, 55.35, 42.04, 41.39, 30.12, 28.42, 22.87, 21.32; **HRMS-APCI**: Found [m/z] = 190.1348; $C_{13}H_{18}O$ requires 190.1348; **[α]$_D$^{26}** = -17 (c = 0.32, $CHCl_3$).

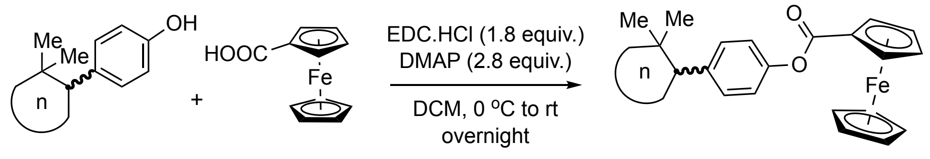

(***R***)**-3** (n = 6) (***S***)**-5** (n = 5) ferrocene carboxylic acid (***R***)**-4** (n = 6) (***S***)**-6** (n = 5)

General Procedure 9 (GP-9): Synthesis of ferrocene ester complex (*R*)-4/(*S*)-6

The reaction was modified from a literature method.[5] An oven dried 25 mL flask under nitrogen was charged with **(*R*)-3/(*S*)-5** (0.125 mmol, 1.00 equiv.), ferrocene-carboxylic acid (1.30 equiv.) and 4-dimethylaminopyridine (DMAP, 2.80 equiv.) followed by addition of anhydrous DCM (5 mL). The reaction mixture was cooled to 0 °C and 1-ethyl-3-(3-dimethyllaminopropyl) carbodiimide hydrochloride (EDC, 1.80 equiv.) was added. The mixture was stirred overnight at room temperature and then diluted with saturated aqueous solution of $NaHCO_3$ and extracted with DCM. The combined organics were dried over Na_2SO_4 and concentrated under reduced pressure. The crude product was purified by column chromatography (silica gel, *ⁿ*Pentane/EtOAc). The product **(*R*)-4/(*S*)-6** was obtained as an orange-yellow solid.

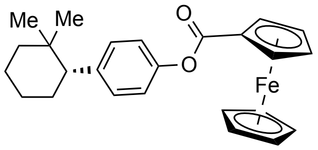

Synthesis of ferrocene ester complex (*R*)-4: (Synthesized according to GP-9, 0.125 mmol scale, yield = 75%, orange-yellow solid): **^{1}H NMR (400 MHz, CDCl$_3$)** δ 7.17 (d, *J* = 8.5 Hz, 2H), 7.07 (d, *J* = 8.5 Hz, 2H), 4.96 (t, *J* = 1.9 Hz, 2H), 4.49 (t, *J* = 1.9 Hz, 2H), 4.30 (s, 5H), 2.41 (dd, *J* = 12.7, 3.3 Hz, 1H), 1.97 – 1.80 (m, 2H), 1.65 – 1.57 (m, 2H), 1.55 – 1.47 (m, 2H), 1.40 – 1.28 (m, 2H), 0.82 (s, 3H), 0.79 (s, 3H); **^{13}C NMR (101 MHz, CDCl$_3$)** δ 170.35, 148.95, 141.37, 130.00, 120.45, 71.82, 70.60, 70.31, 69.91, 53.27, 42.19, 34.10, 31.30, 28.54, 27.29, 22.54, 19.55; **HRMS-ESI** [M+H]$^+$**:** Found [m/z] = 417.1505; $C_{25}H_{29}FeO_2$ requires 417.1506; **[α]$_D$^{26}** = +8.1 (c = 0.33, $CHCl_3$).

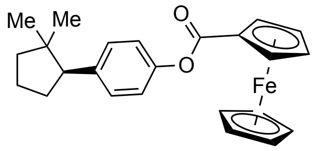

Synthesis of ferrocene ester complex (*S*)-6: (Synthesized according to GP-9, 0.125 mmol scale, yield = 82%, orange-yellow solid): **^{1}H NMR (400 MHz, CDCl$_3$)** δ 7.23 (d, *J* = 8.5 Hz, 2H), 7.10 (d, *J* = 8.6 Hz, 2H), 4.96 (t, *J* = 2.0 Hz, 2H), 4.49 (t, *J* = 1.9 Hz, 2H), 4.30 (s, 5H), 2.72 (dd, *J* = 10.7, 8.2 Hz, 1H), 2.10 – 2.00 (m, 2H), 1.89 – 1.69 (m, 3H), 1.66 – 1.59 (m, 1H), 1.01 (s, 3H), 0.66 (s, 3H); **^{13}C NMR (101 MHz, CDCl$_3$)** δ 170.35, 149.10, 139.34, 129.51, 120.75, 71.82, 70.61, 70.30, 69.92, 55.66, 42.26, 41.48, 30.19, 28.44, 22.95, 21.40; **HRMS-ESI** [M+H]$^+$**:** Found [m/z] = 403.1345; $C_{24}H_{27}FeO_2$ requires 403.1349; **[α]$_D$^{26}** = -5 (c = 0.59, $CHCl_3$).

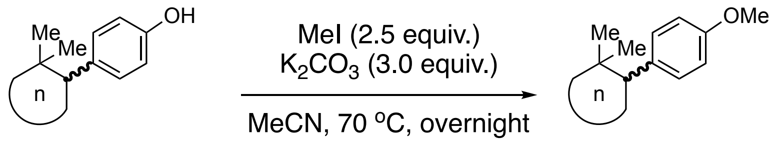

(*R*)-3 (n = 6)
(*S*)-5 (n = 5)

(*R*)-2a (n = 6)
(*S*)-2j (n = 5)

General procedure for O-methylation: Methyl iodide (2.5 equiv.) and K_2CO_3 (3.0 equiv.) was added to a stirred solution of corresponding phenol **(*R*)-3/(*S*)-5** (1.0 equiv., 0.1 mmol) in MeCN (2 mL). The reaction mixture was stirred at 70°C for 12 hours. After completion, the crude product was filtered through celite to remove the solid waste and finally purified by silica gel column chromatography using 2-5% diethyl ether in pentane as the eluent.

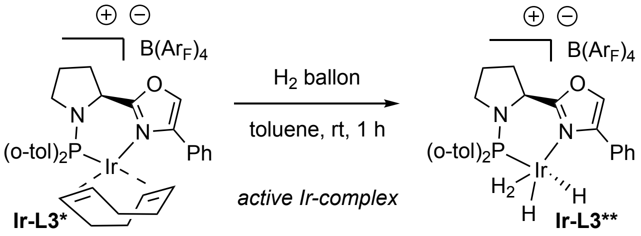

Procedure for H_2 actived Ir-catalyst preparation: An oven-dried 6 mL glass vial equipped with a magnetic stirring bar was charged with the Iridium pre-catalyst (2.0 mol% with respect to alcohol starting material). Evacuated and refilled with argon three time. Finally, the argon gas was evacuated and refilled with hydrogen gas (H_2 ballon) and dry toluene (2.5 mL) was added. The H_2 gas was bubbled through the solution via the needles and was stirred for further 10 minutes.

X-Ray Crystallography

Data collection was done with a Bruker Venture D8 equipped with a Incotec MoKα microfocus X-ray source and a Photon III detector. All data collections were done at 291K. All data collections were done with the detector at 50 mm distance from the crystal.

The data collection was controlled by the Apex 5 software package and also used for the reduction of diffraction data. The crystal structures were solved with conventional direct method procedures using SHELXT and refined with full matrix least square calculations using SHELXL. The hydrogen positions was geometrically positioned and refined with riding motion using the different AFIX possibilities available in SHELXL. The absolute configuration of both strucutres was determined by refining the structure model as an inversion twin.

Crystal data and refinement results can be found in tables below. Cif files have been deposited with the Cambridge Crystallographic Data Centre.

Table S2. Crystal data for the ferrocene complexes (*R*)-4 and (*S*)-6

X-ray crystal structure	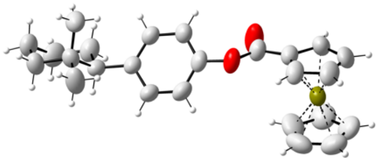 **(*R*)-4**	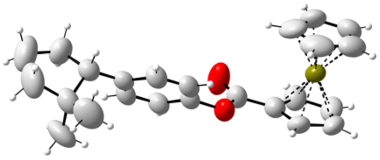 **(*S*)-6**
Sum formula	C_{25} H_{28} Fe O_2	C_{24} H_{26} Fe O_2
Formula Weight / g·mol^{-1}	416.32	402.30
CCDC-code	2494033	2501261
Temperature / K	291	293
Wavelength / Å	0.71073 (MoKα)	0.71073 (MoKα)
Crystal size / mm	0.20 × 0.05 × 0.04	0.15 × 0.08 × 0.05
Crystal system	monoclinic	monoclinic
Space group	$P2_1$ (nr. 4)	$P2_1$ (nr. 4)
a / Å	6.58250(10) Å	6.5932(2)
b / Å	11.1628(3) Å	10.8543(2)
c / Å	14.5389(4) Å	14.3720(4)
α / °	90	90
β / °	99.0660(10)	101.0010(10)
γ / °	90	90
Volume / Å^3	1054.96(4) Å^3	1009.61(5)
Z	2	2
Density, ρcalc / g cm^{-3}	1.311	1.323
μ / mm^{-1}	0.732	0.762
F(000)	440	424

Θmin, Θmax	2.31, 25.58	3.20, 26.84
Index ranges,	−8 ≤ h ≤ 6,<br>−13 ≤ k ≤ 13,<br>−18 ≤ l ≤ 18	−8 ≤ h ≤ 8,<br>−13 ≤ k ≤ 13,<br>−17 ≤ l ≤ 17
Reflections collected	26983	19001
Unique reflections	4203	3842
Obs. refl. (I ≥ 2σ(I))	3955	3654
Rint	0.0387	0.0460
Parameters	256	247
R1[a] (obs), R1 (all)	0.0257, 0.0285	0.0294, 0.0315
wR2[b] (obs), wR2 (all)	0.0650, 0.0666	0.0703, 0.0717
GOOF	1.065	1.037
Flack parameter	0.010(17)	0.018(19)
Residual densities (min,max,rms)	−0.122, 0.118, 0.023	-0.118, 0.122, 0.021

[a]$R1 = \sum\cdot(||F_o|-|F_c||)/\sum(|F_o|)$, [b]$wR2 = (\sum\cdot w(F_o^2-F_c^2)^2/\sum F_o^2)^{1/2}$

References:

1) Yan, J.-X. et al. Palladium-Catalyzed C(sp3)-H Activation: A Facile Method for the Synthesis of 3,4- Dihydroquinolinone Derivatives. *Angew. Chem., Int. Ed.* **2014,** *53*, 4945–4949.
2) Wakchaure, V. N., DeSnoo, W., Laconsay, C. J. et al. Catalytic asymmetric cationic shifts of aliphatic hydrocarbons. *Nature*, **2024**, 625, 287–292.
3) Houston, S. D. et al. Cyclooctatetraenes through Valence Isomerization of Cubanes: Scope and Limitations. *Chem. Eur. J.* **2019**, *25*, 2735–2739.
4) Cao, Y.; Yang, X.; Du, D.; Xu, X.; Song, F.; Xu, L. New System of Deprotection Step for the Hydroxide Radicals: Boron Trifluoride Etherate/Sodium Iodide. *International Journal of Chemistry, 2011, 3,* DOI: 10.5539/ijc.v3n3p113.
5) Shi, L.; Tsuji, N.; Zhu, C.; Leutzsch, M.; Grimm, J. A. A.; List, B. Catalytic Asymmetric (ene−endo)-Carbonyl−Ene Type Cyclizations. *J. Am. Chem. Soc.* **2025**, *147*, 34225−34230.

NMR spectra of starting materials

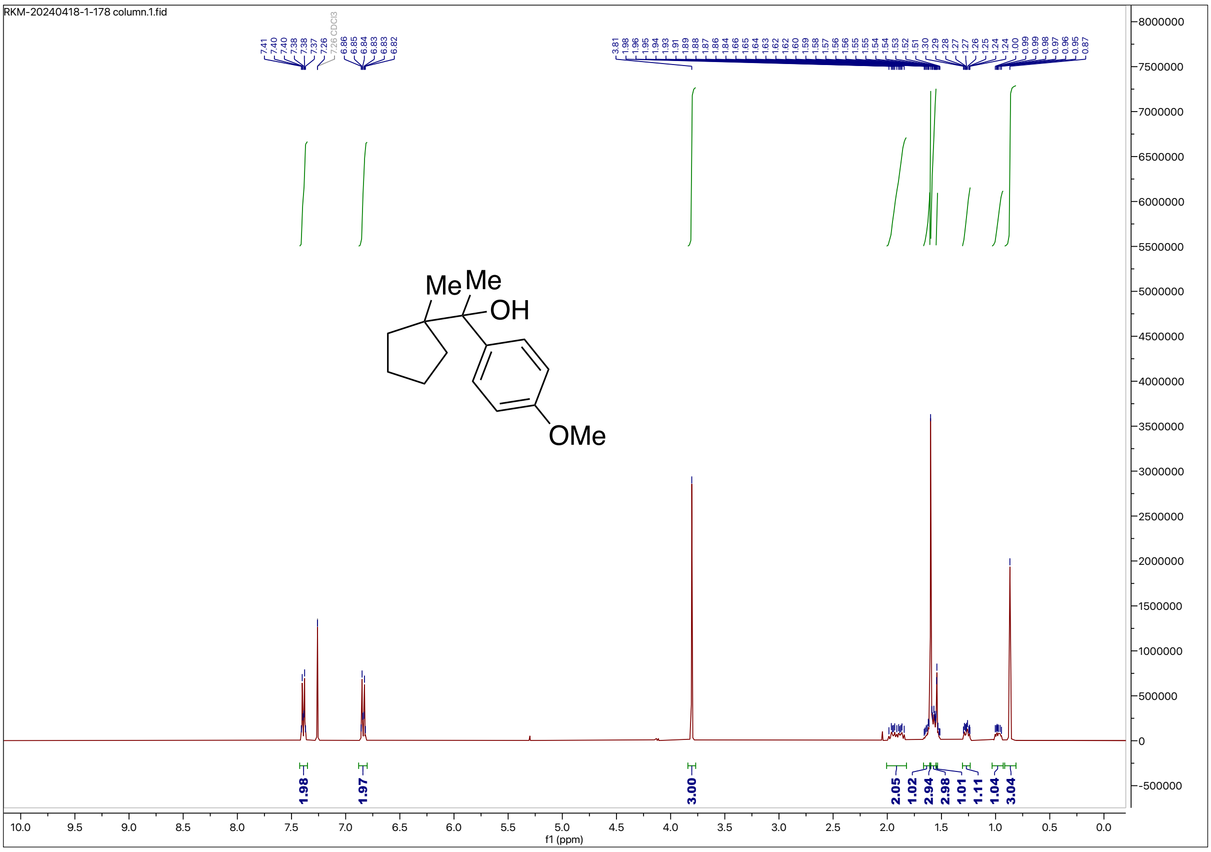

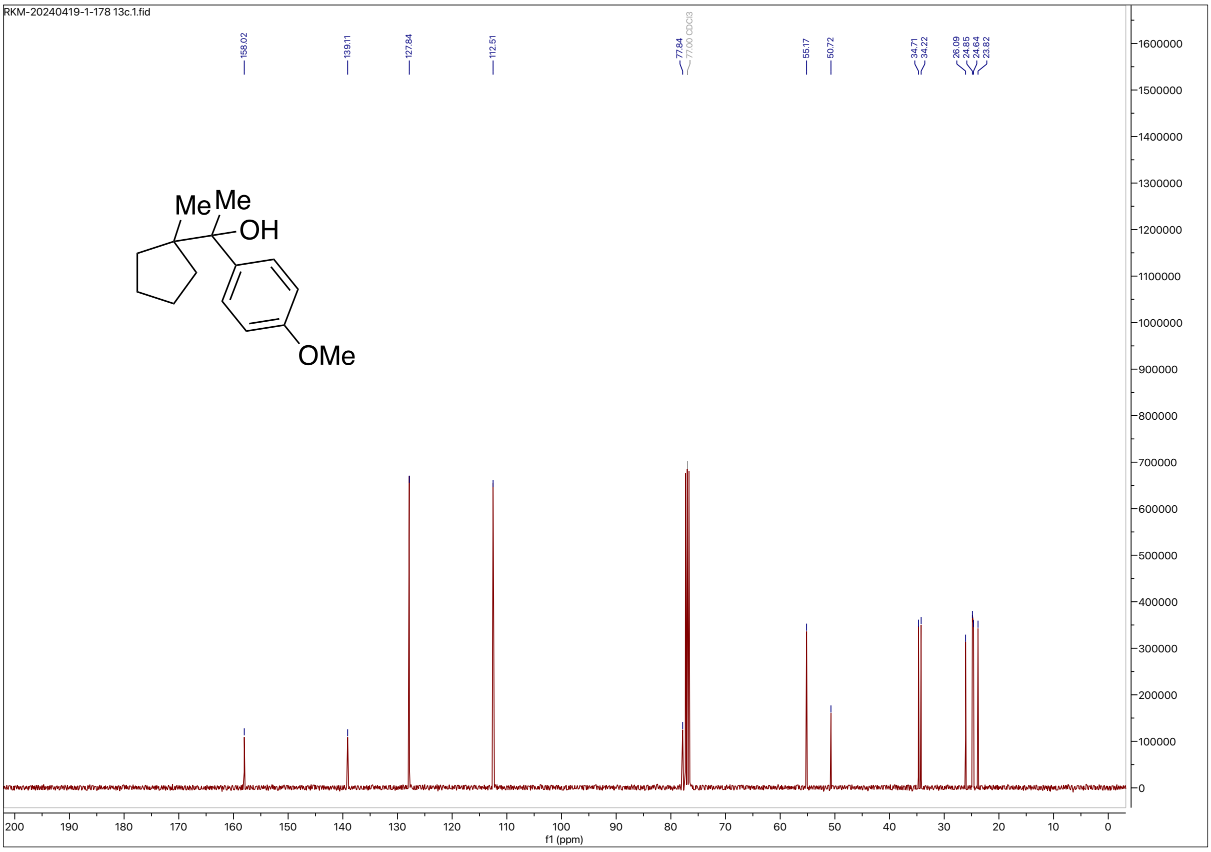

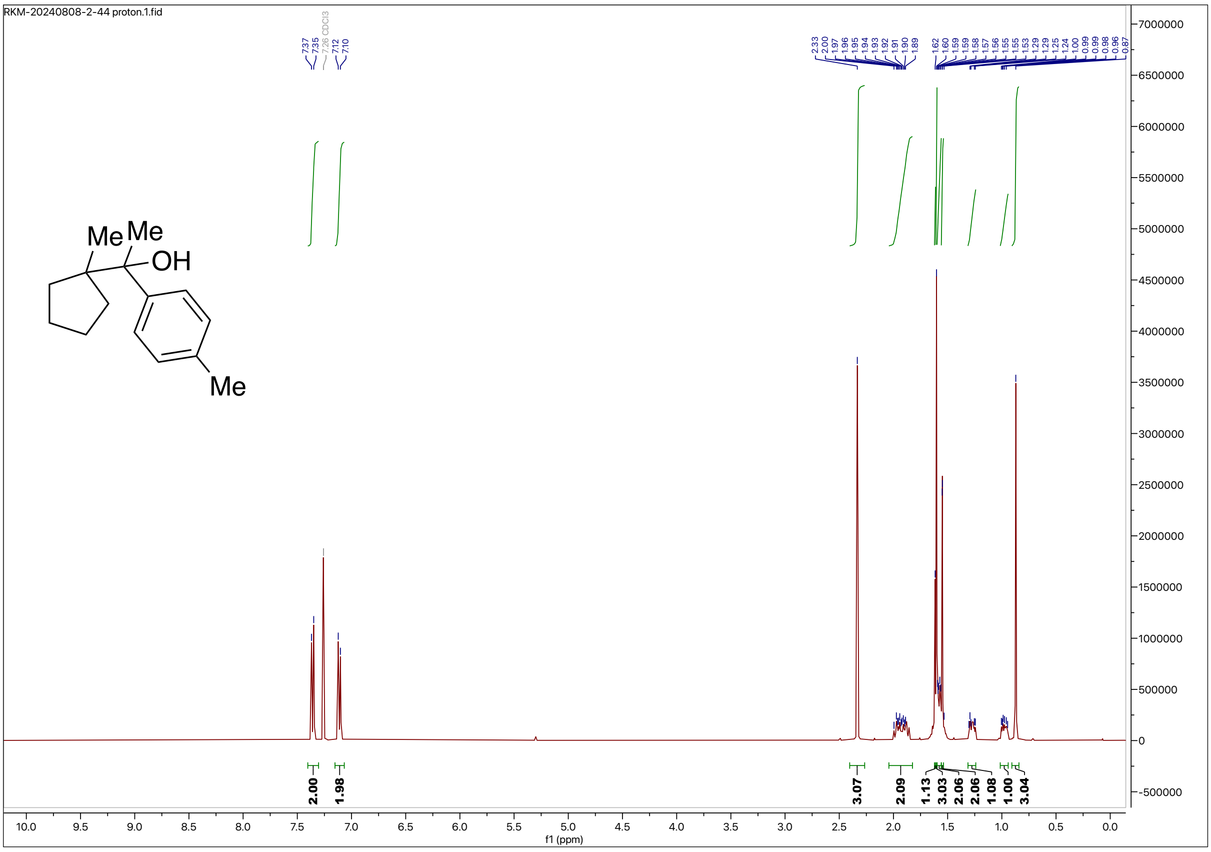
RKM-20240808-2-44 proton.1.fid
f1 (ppm)
Me
Me
OH
Me

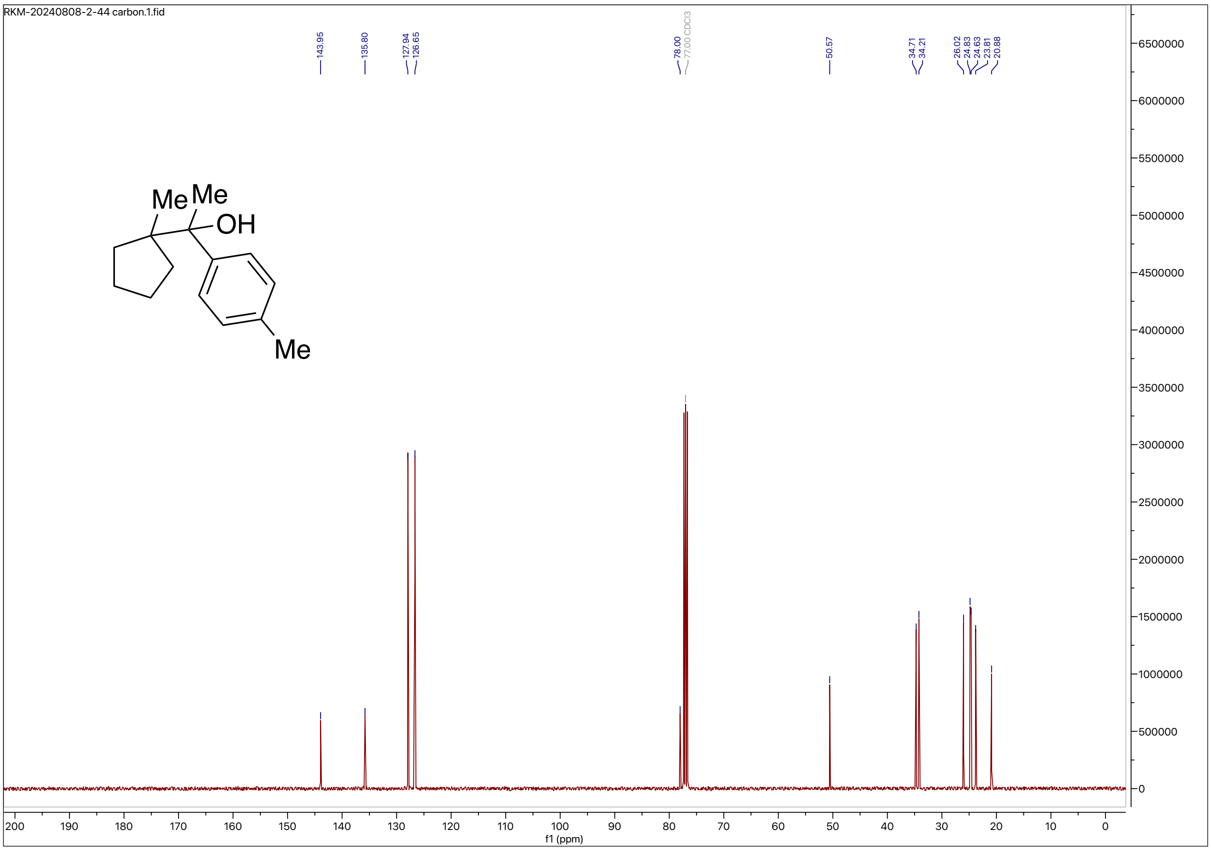
RKM-20240808-2-44 carbon.1.fid
f1 (ppm)
143.95
135.80
127.94
126.65
78.00
77.00 CDCl3
50.57
34.71
34.21
26.02
24.83
24.63
23.81
20.88
Me
Me
OH
Me

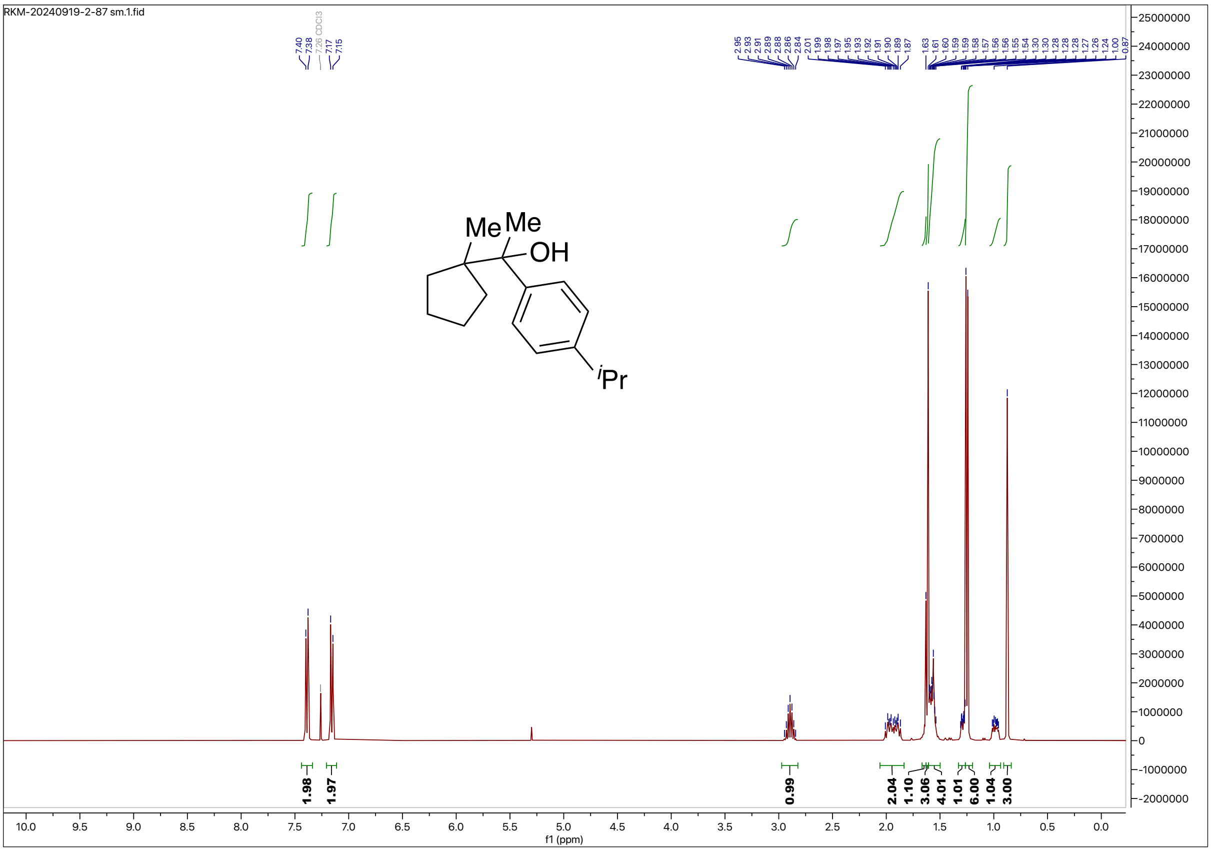
RKM-20240919-2-87 sm.1.fid
Me
Me
OH
iPr
1.98
1.97
0.99
2.04
1.10
3.06
4.01
1.01
6.00
1.04
3.00
f1 (ppm)

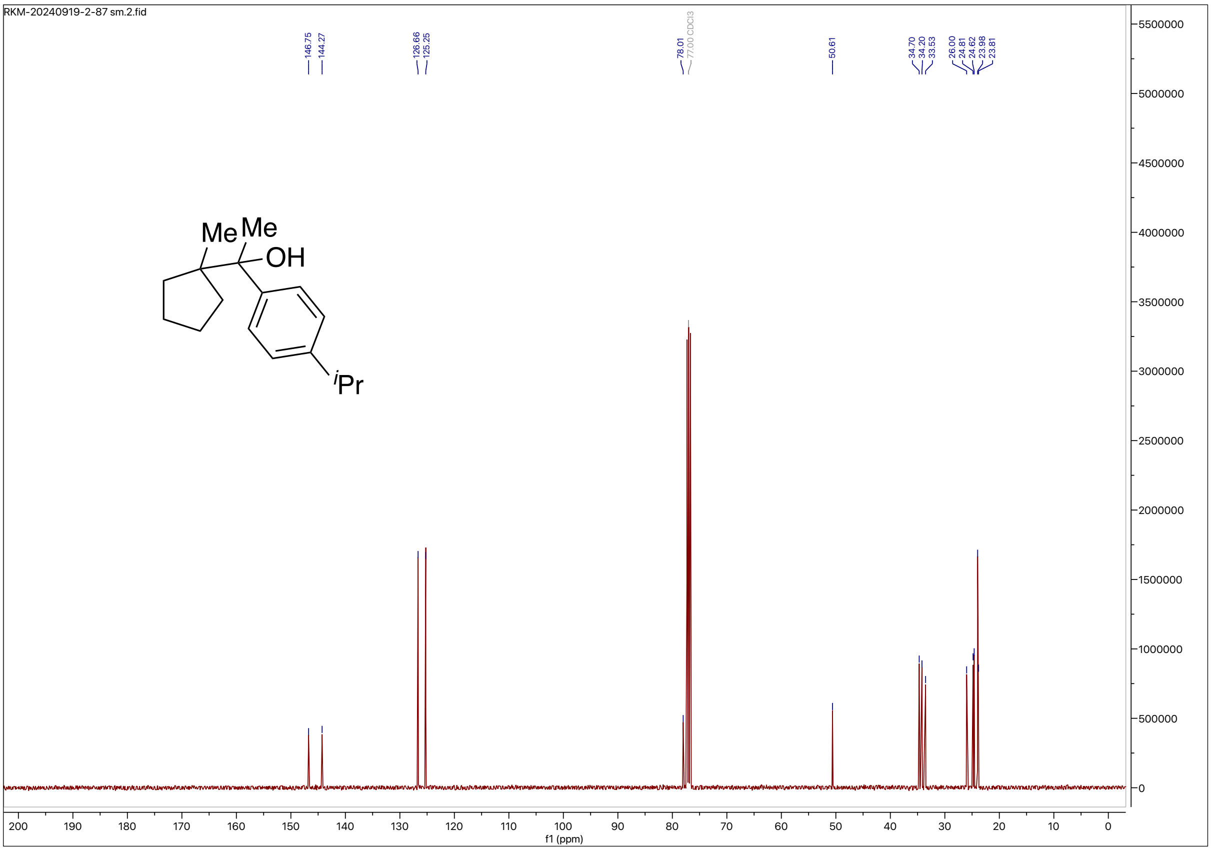
RKM-20240919-2-87 sm.2.fid
146.75
144.27
126.66
125.25
78.01
77.00 CDCl3
50.61
34.70
34.20
33.53
26.00
24.81
24.62
23.98
23.81
Me
Me
OH
iPr
f1 (ppm)

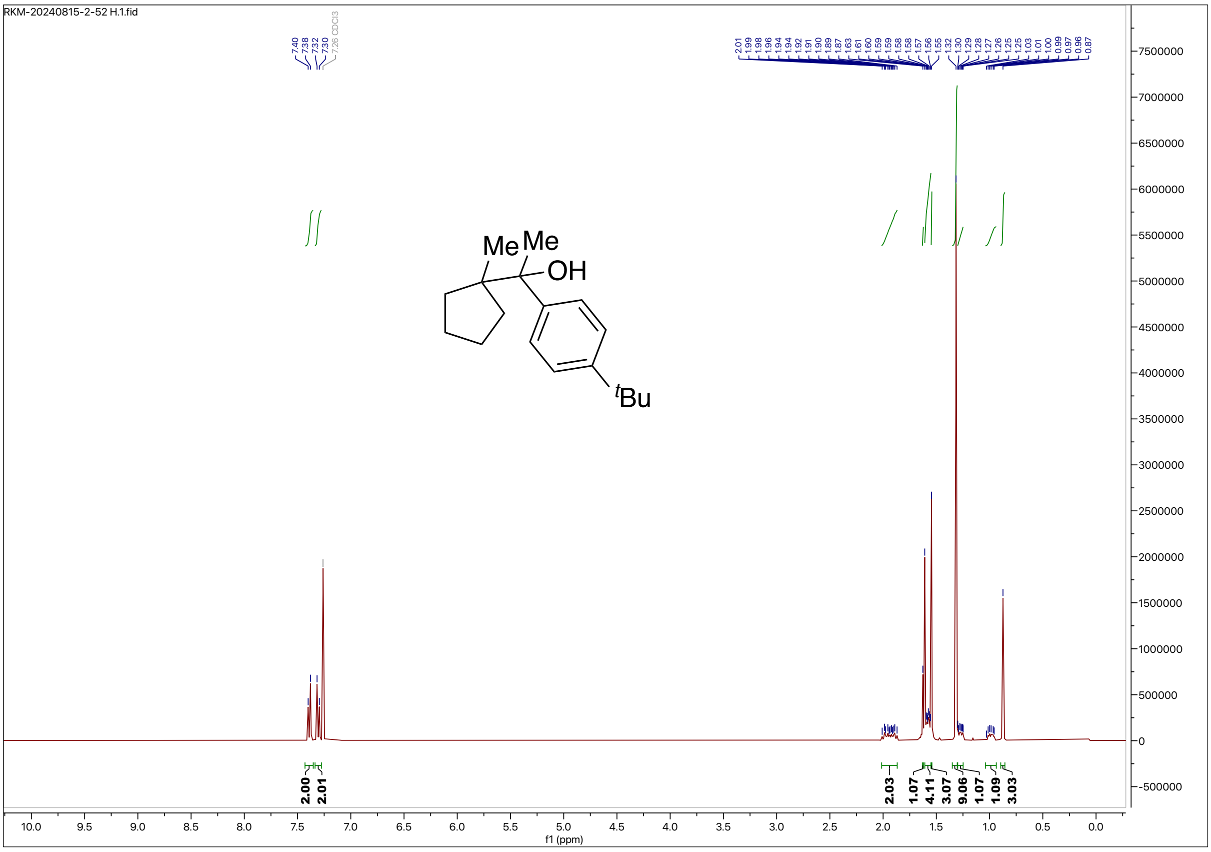

RKM-20240815-2-52 H.1.fid
Me
Me
OH
tBu
f1 (ppm)

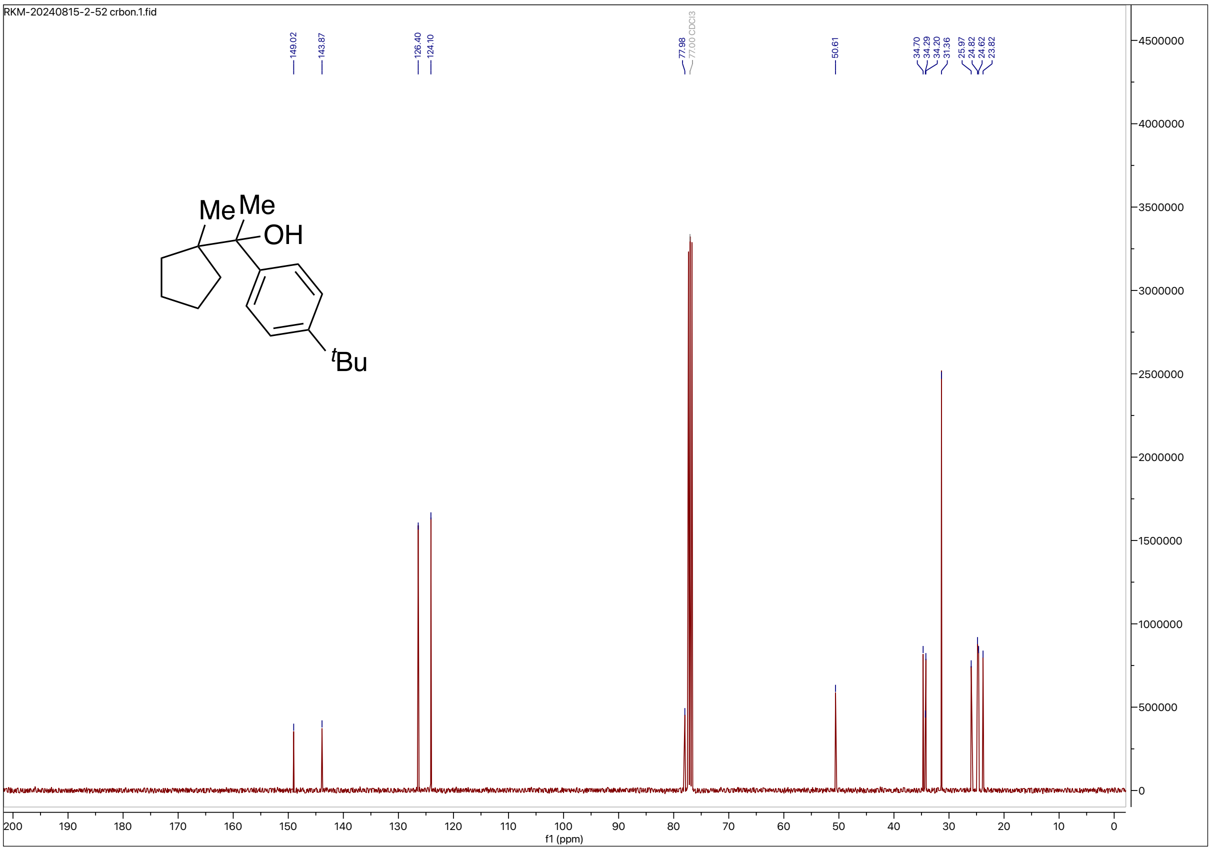

RKM-20240815-2-52 crbon.1.fid
149.02
143.87
126.40
124.10
77.98
77.00 CDCl3
50.61
34.70
34.29
34.20
31.36
25.97
24.82
24.62
23.82
Me
Me
OH
tBu
f1 (ppm)

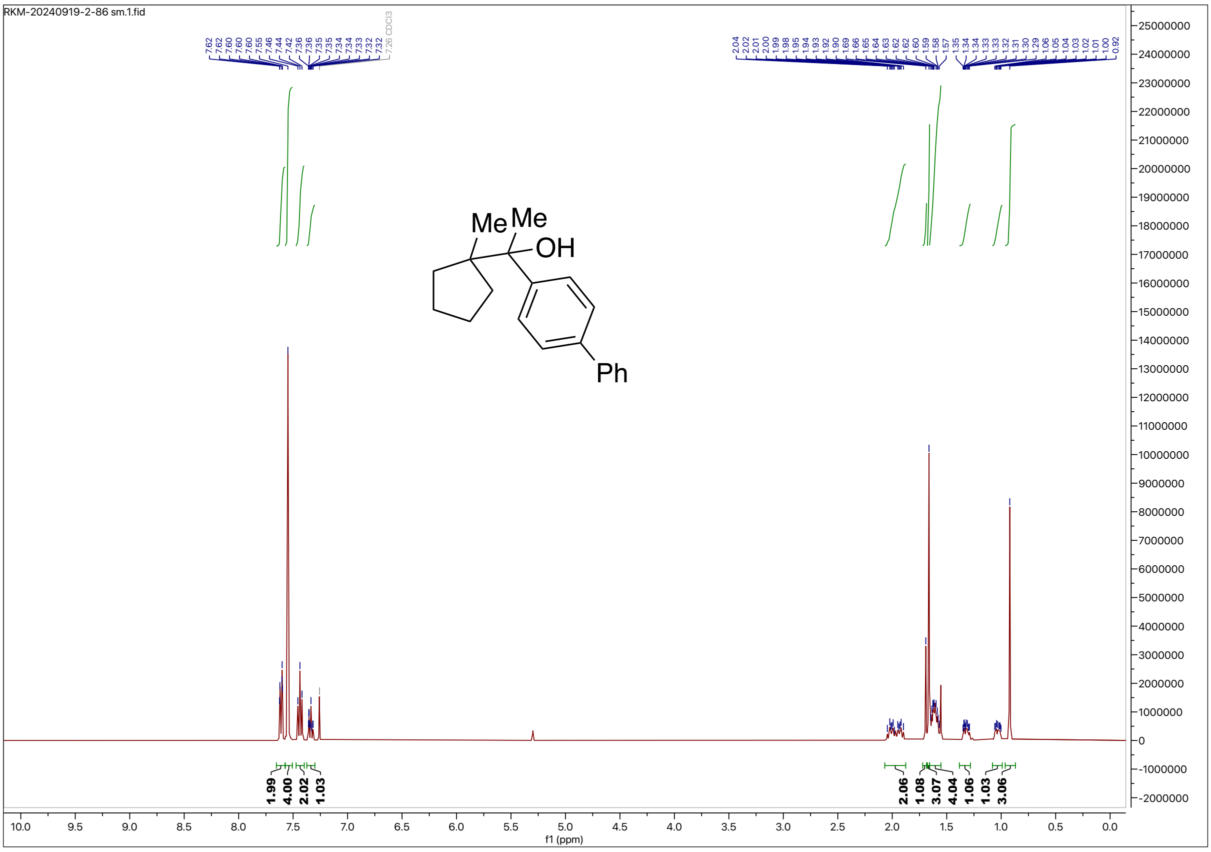

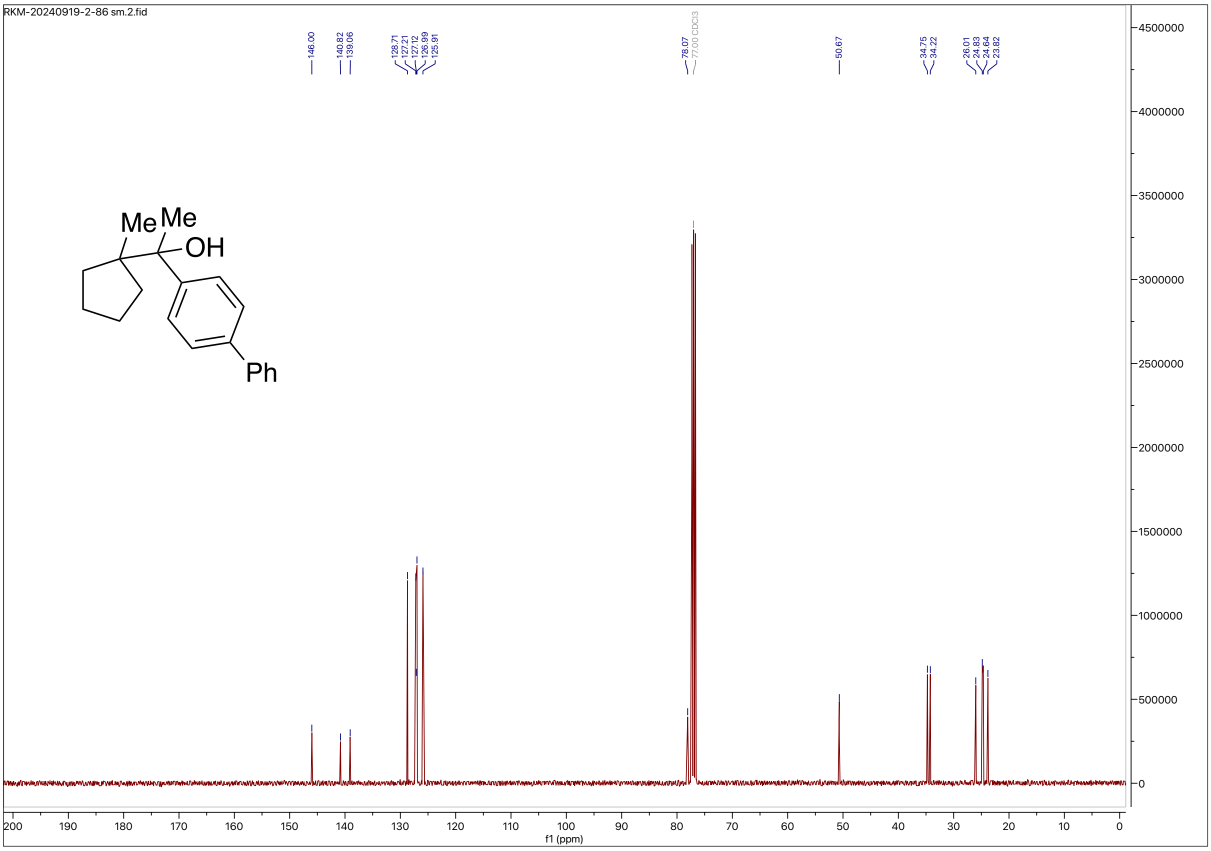

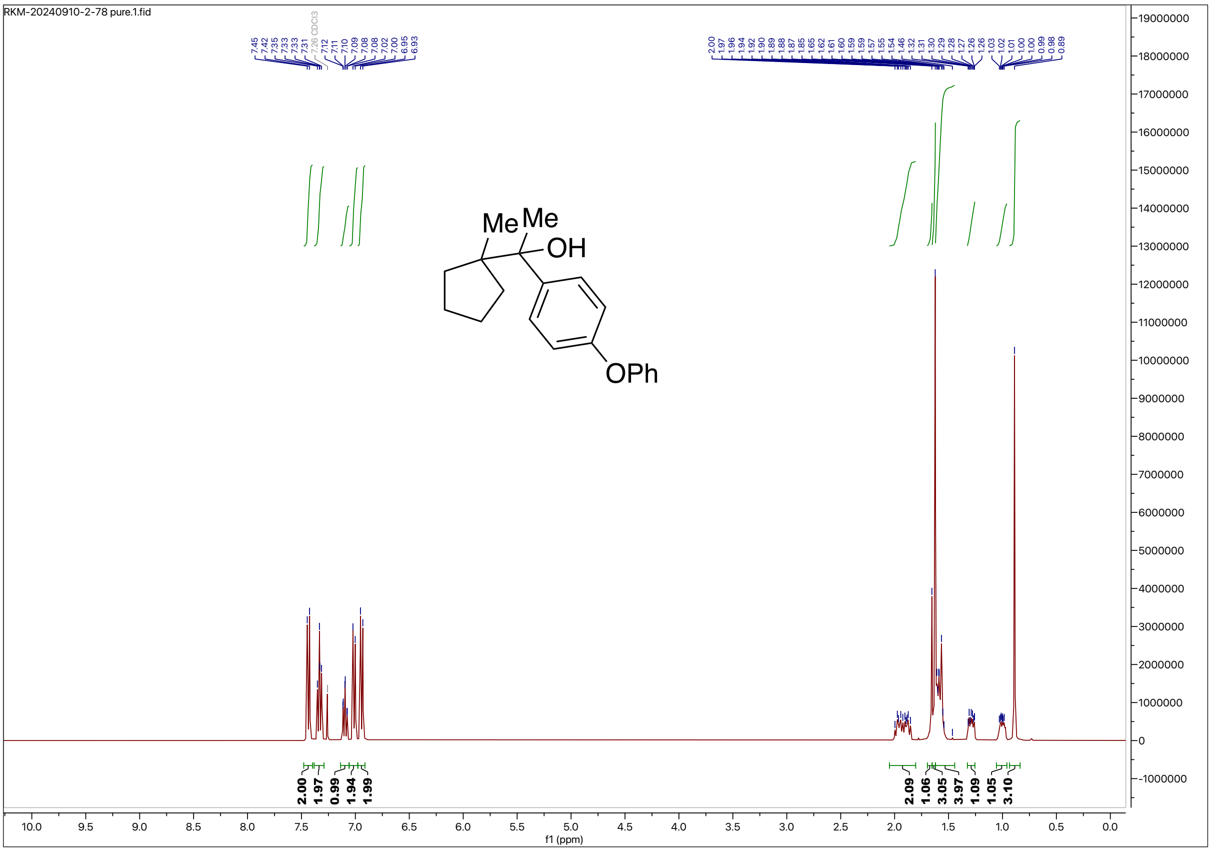

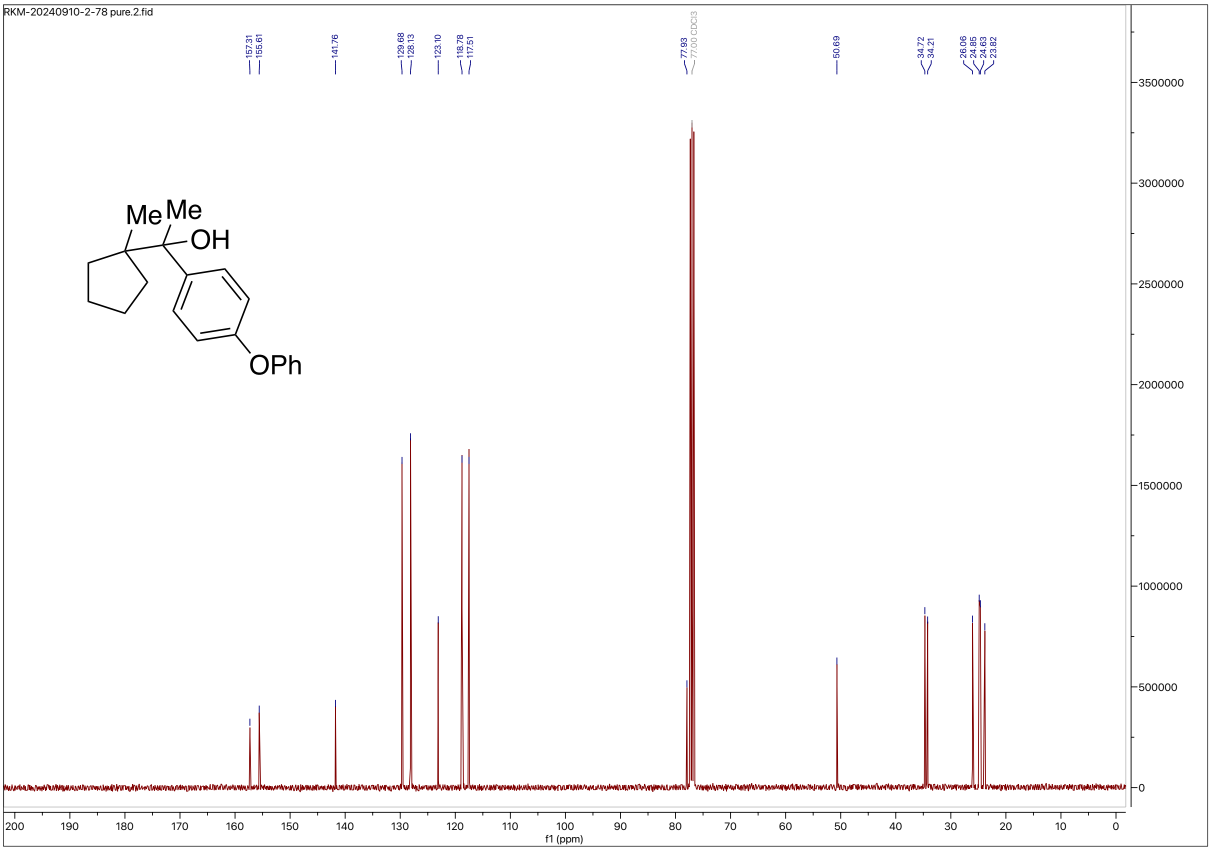

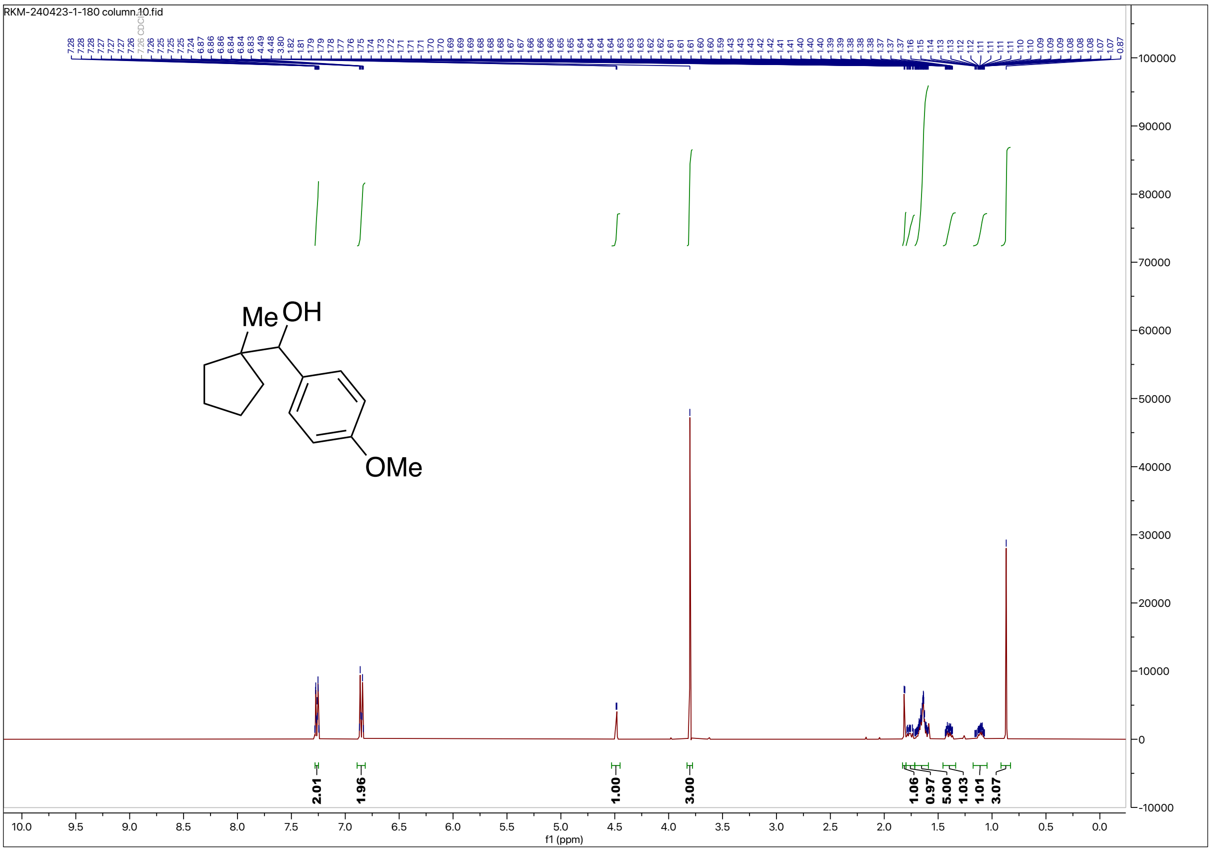
RKM-240423-1-180 column.10.fid
Me OH
OMe
2.01
1.96
1.00
3.00
1.06
0.97
5.00
1.03
1.01
3.07
f1 (ppm)

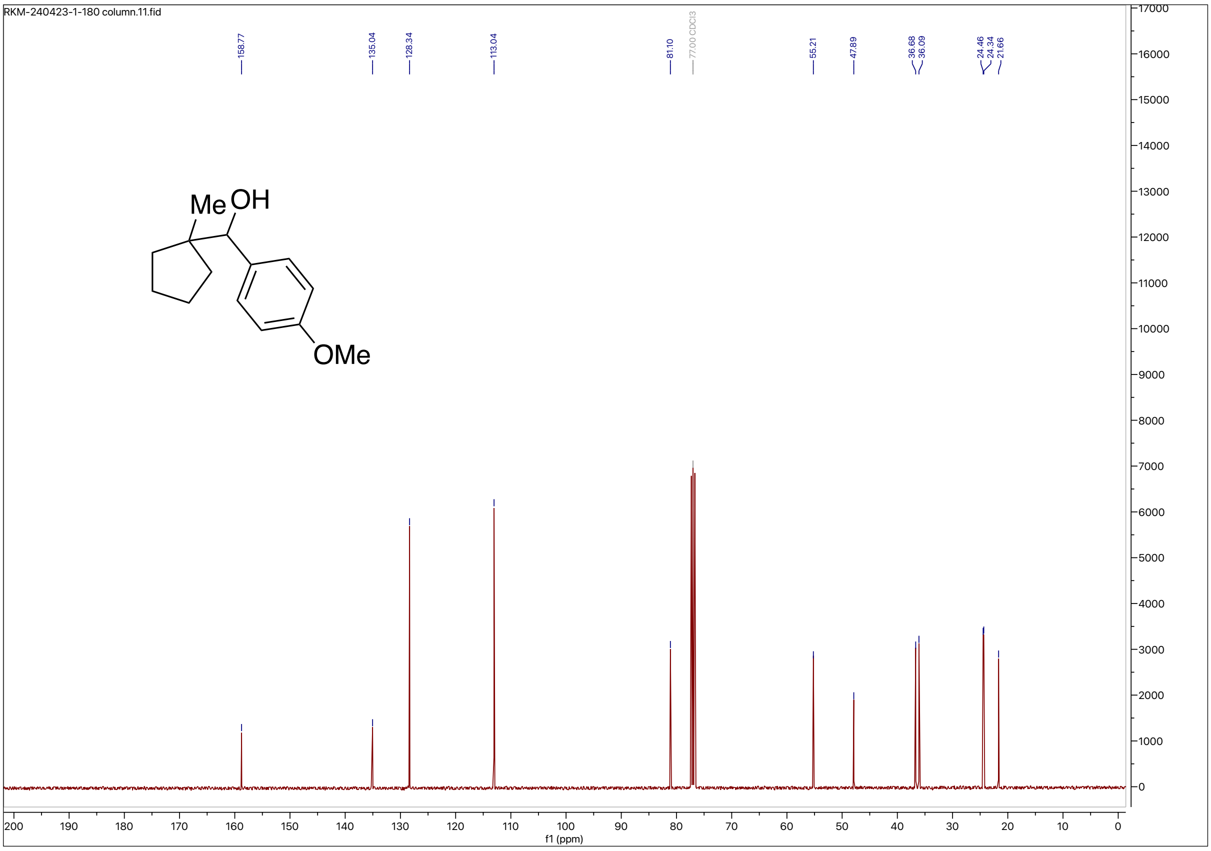
RKM-240423-1-180 column.11.fid
158.77
135.04
128.34
113.04
81.10
77.00 CDCl3
55.21
47.89
36.68
36.09
24.46
24.34
21.66
Me OH
OMe
f1 (ppm)

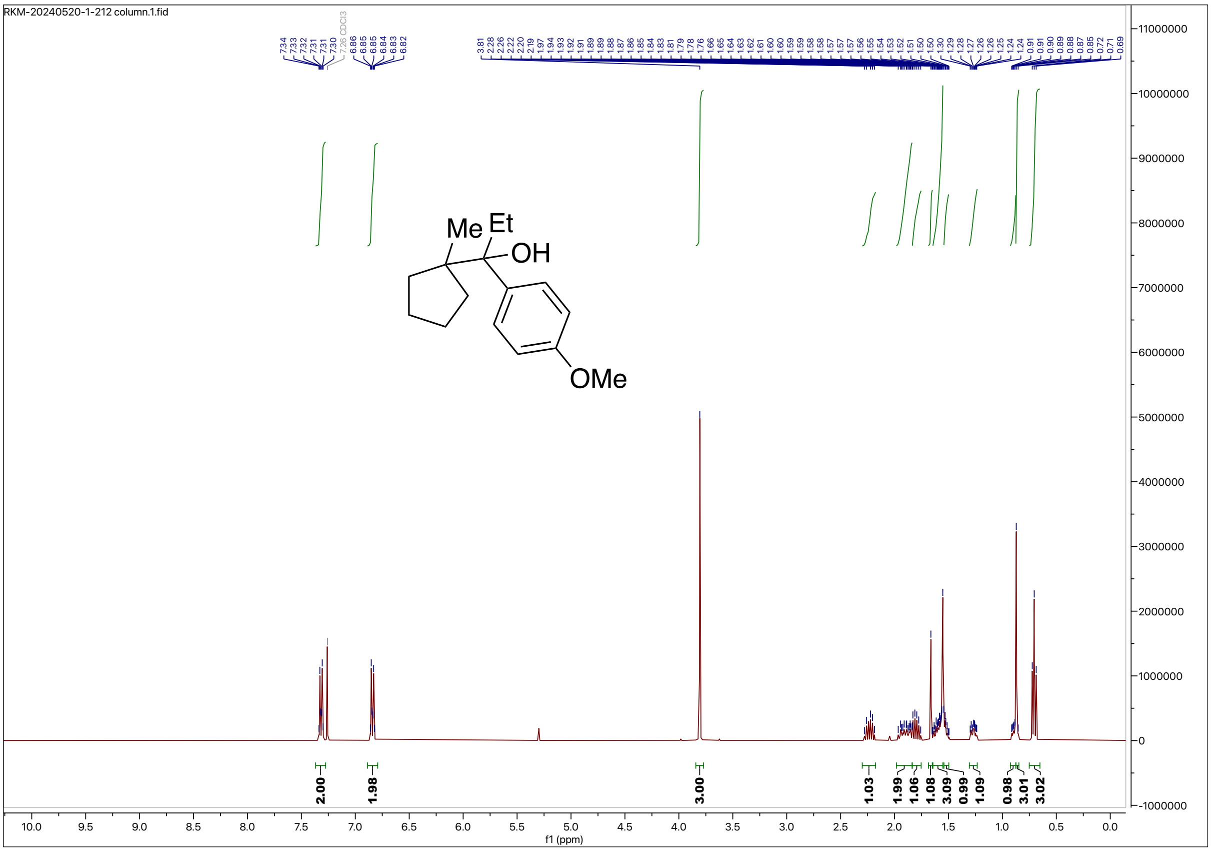
RKM-20240520-1-212 column.1.fid
Me
Et
OH
OMe
f1 (ppm)

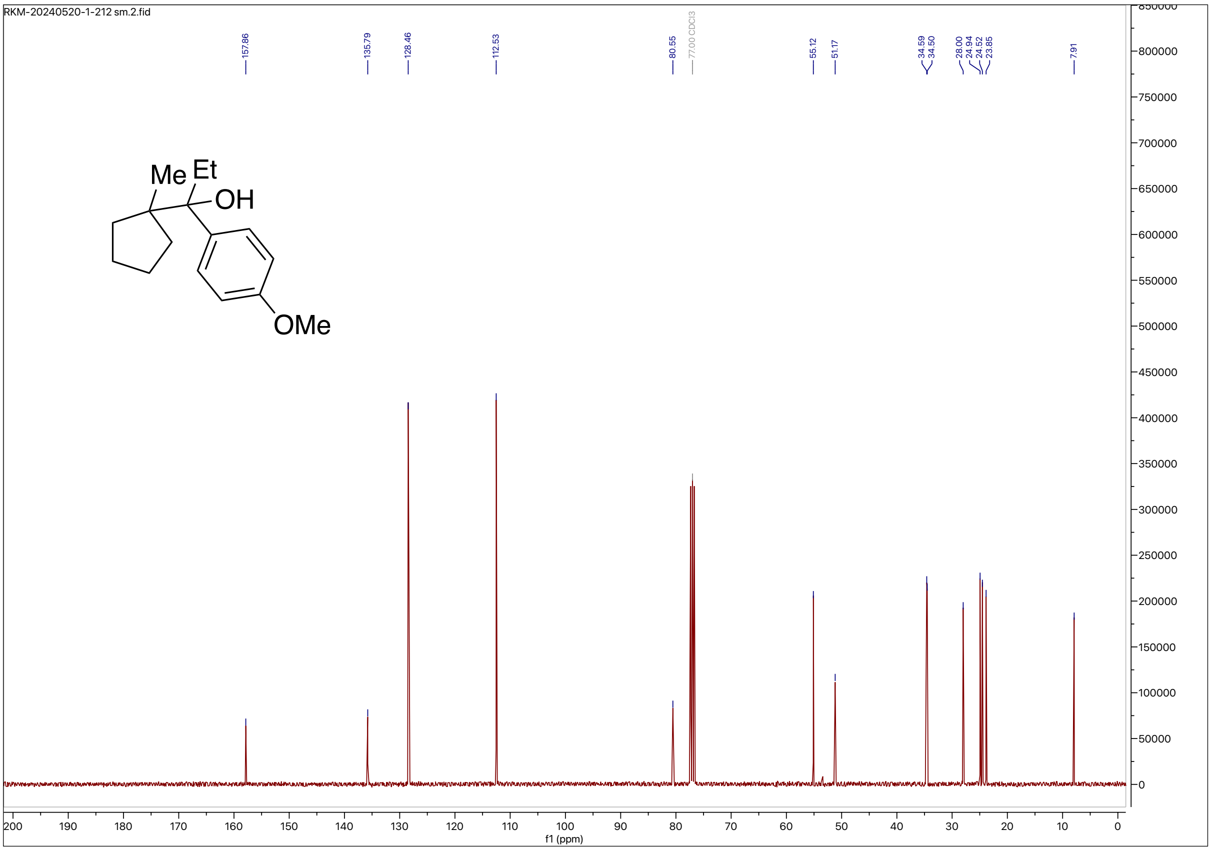
RKM-20240520-1-212 sm.2.fid
157.86
135.79
128.46
112.53
80.55
77.00 CDCl3
55.12
51.17
34.59
34.50
28.00
24.94
24.52
23.85
7.91
Me
Et
OH
OMe
f1 (ppm)

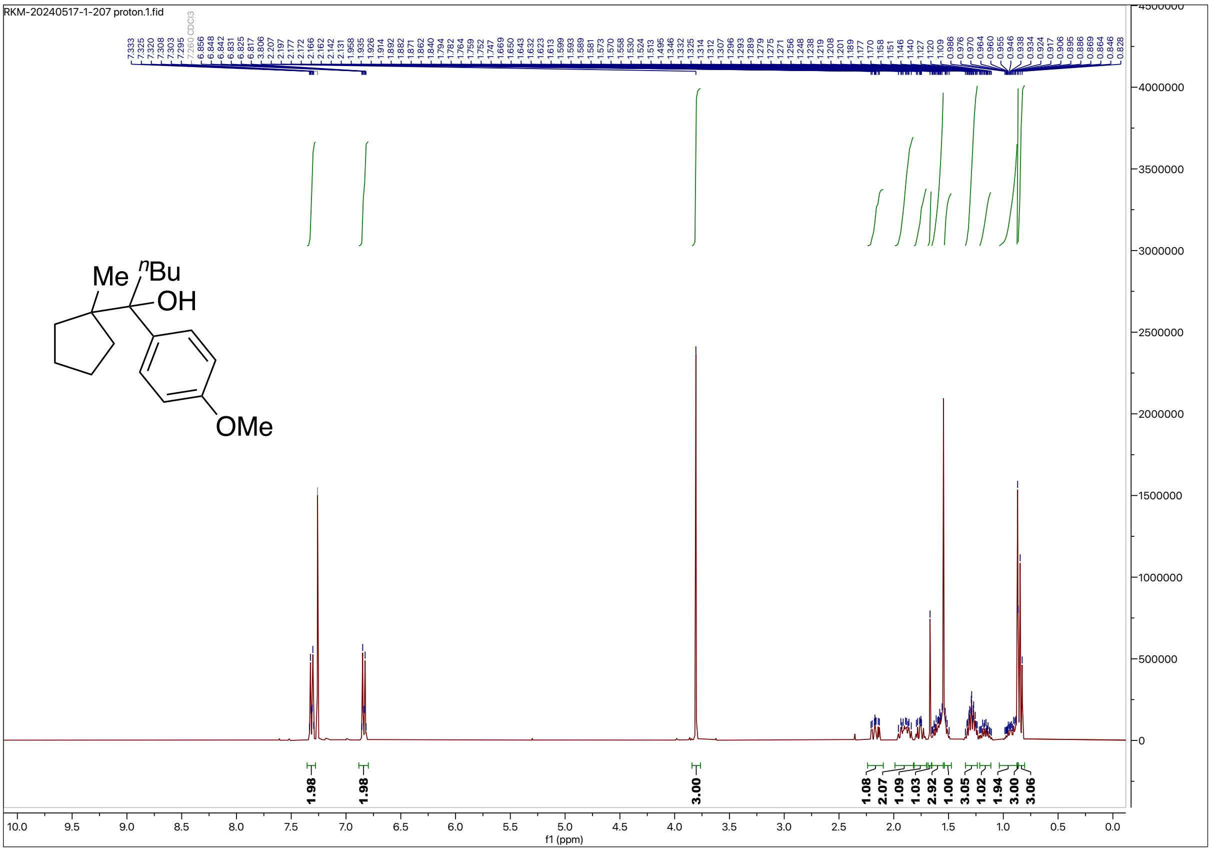

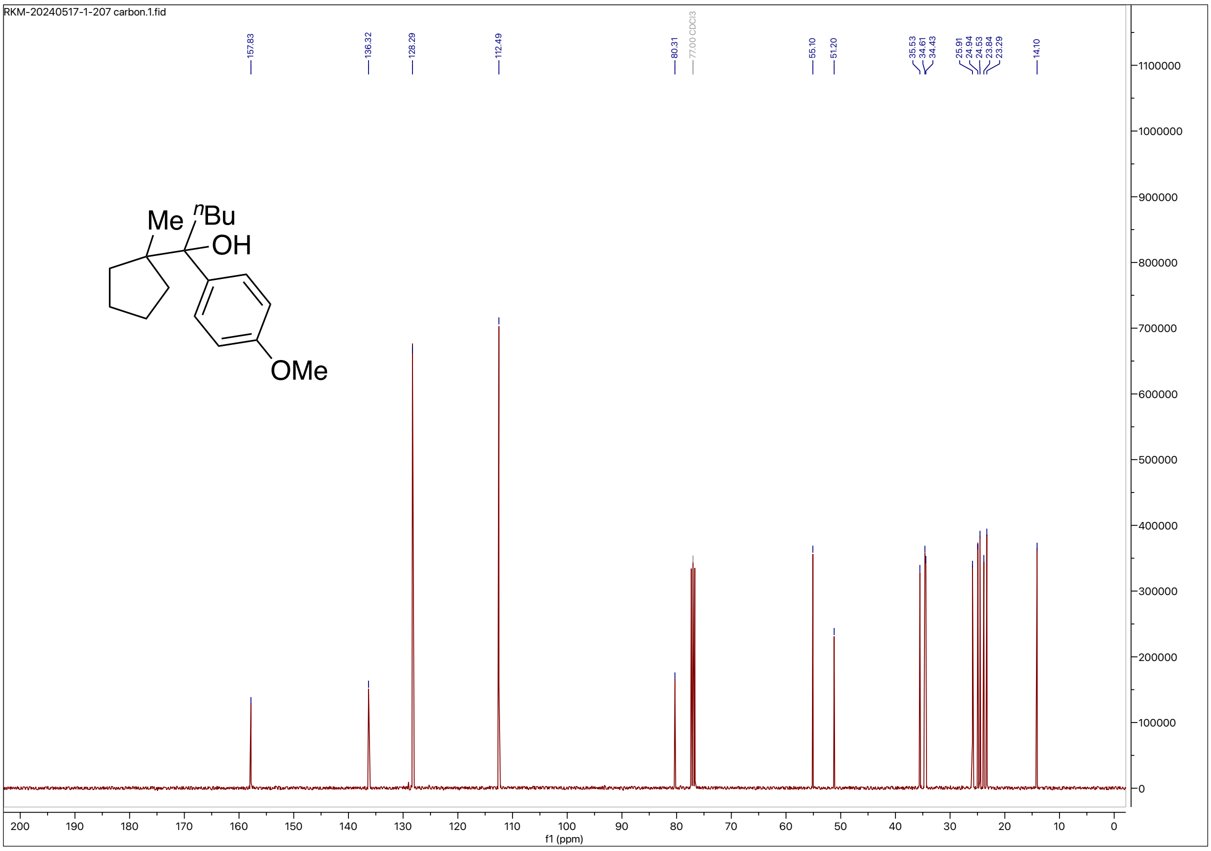

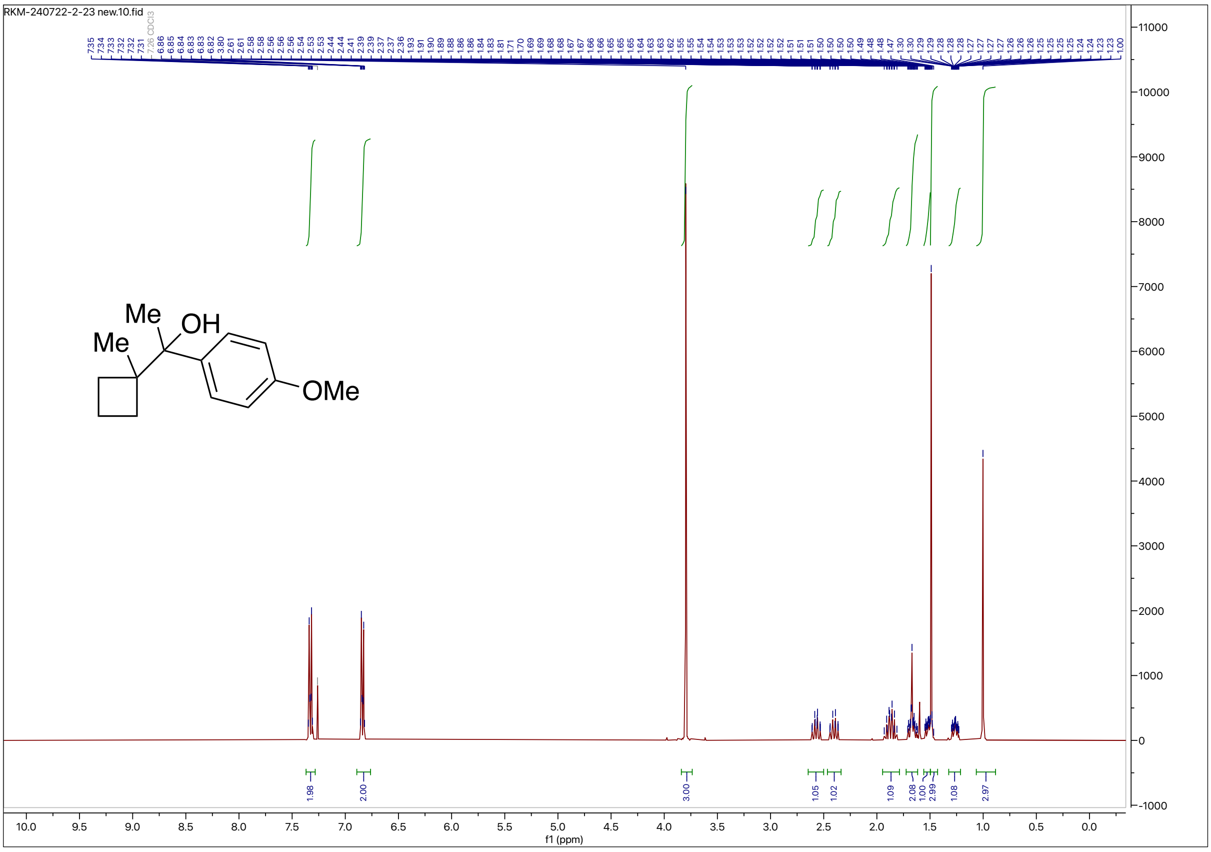

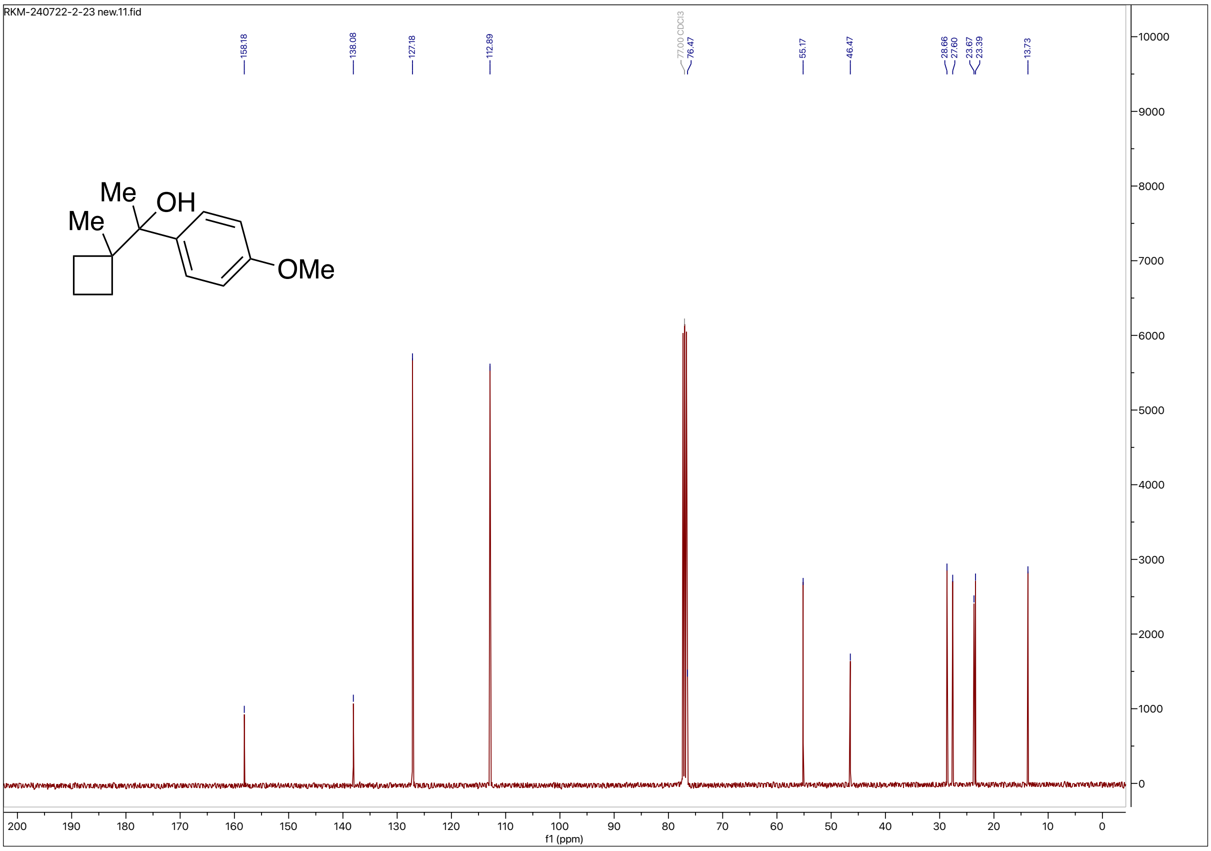

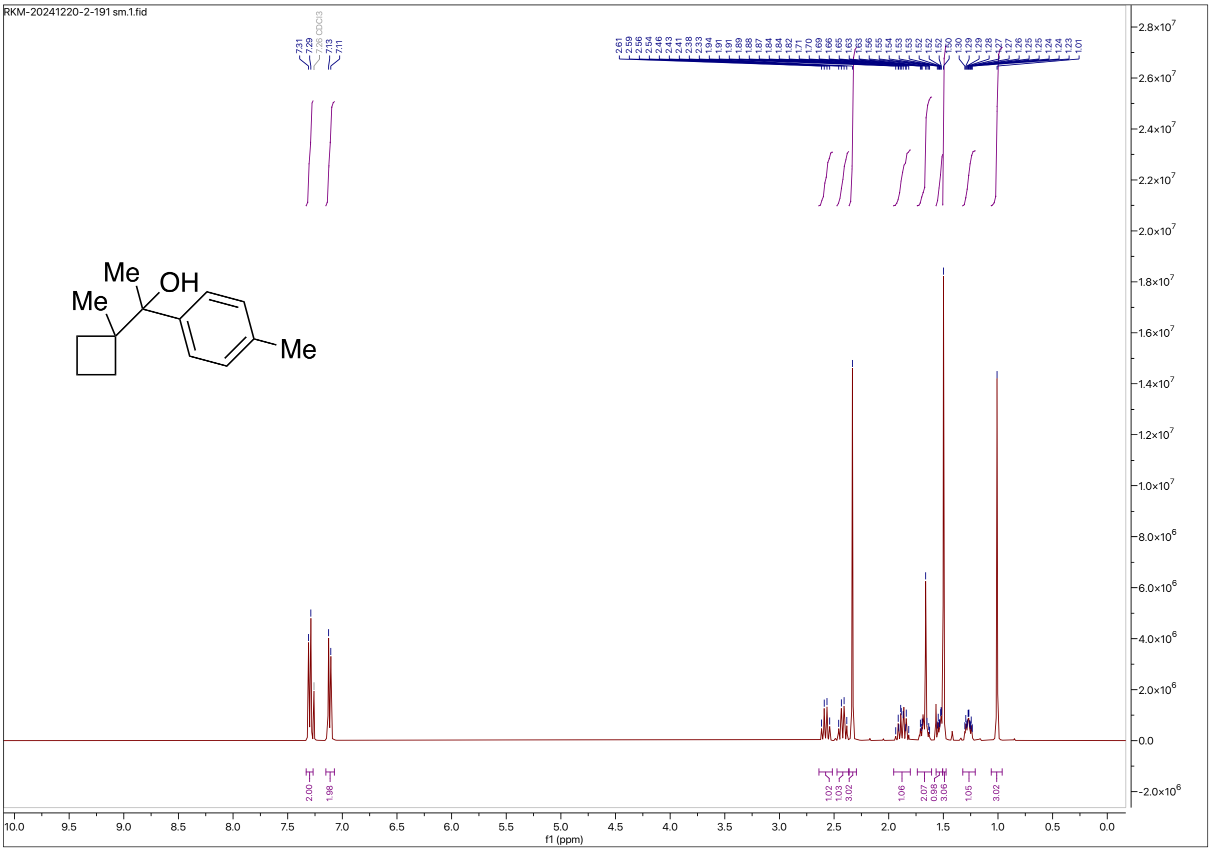

RKM-20241220-2-191 sm.1.fid
Me
OH
Me
Me
f1 (ppm)

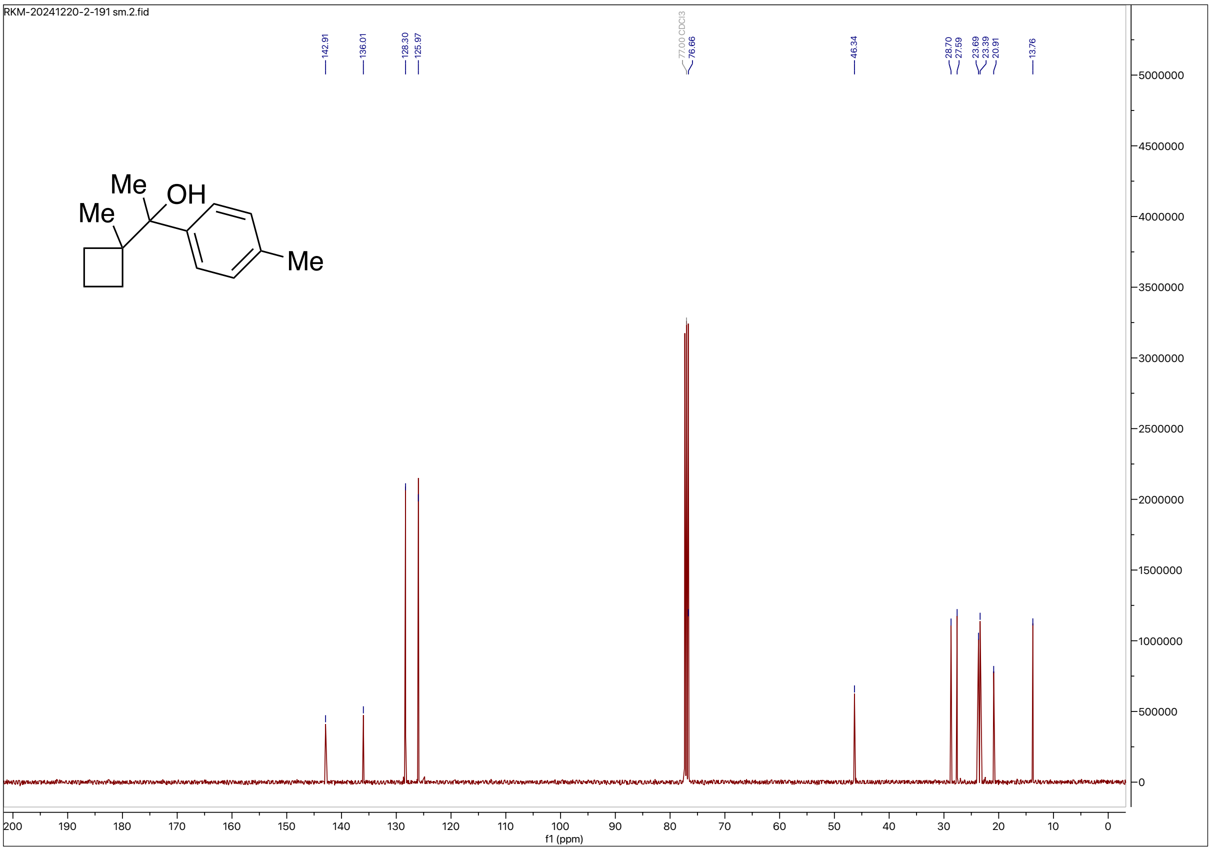

RKM-20241220-2-191 sm.2.fid
142.91
136.01
128.30
125.97
77.00 CDCl3
76.66
46.34
28.70
27.59
23.69
23.39
20.91
13.76
Me
OH
Me
Me
f1 (ppm)

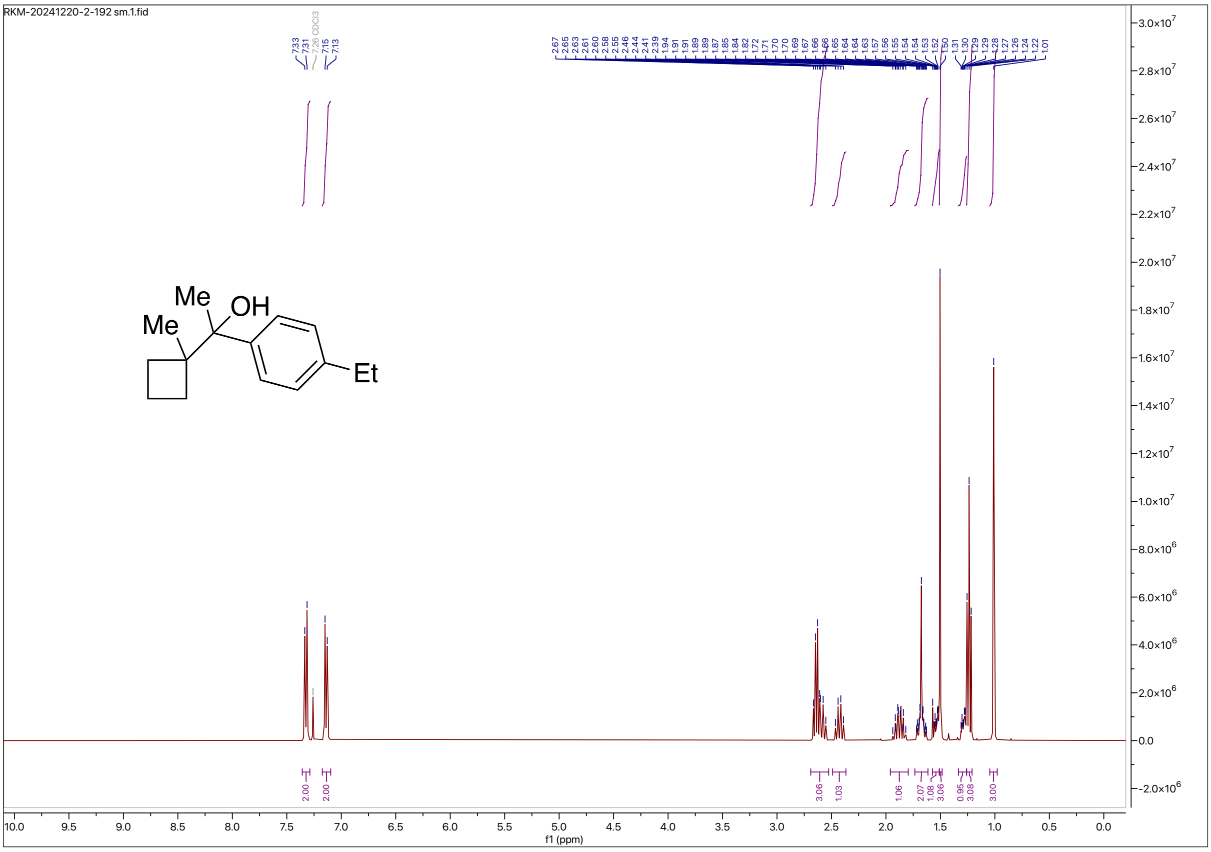

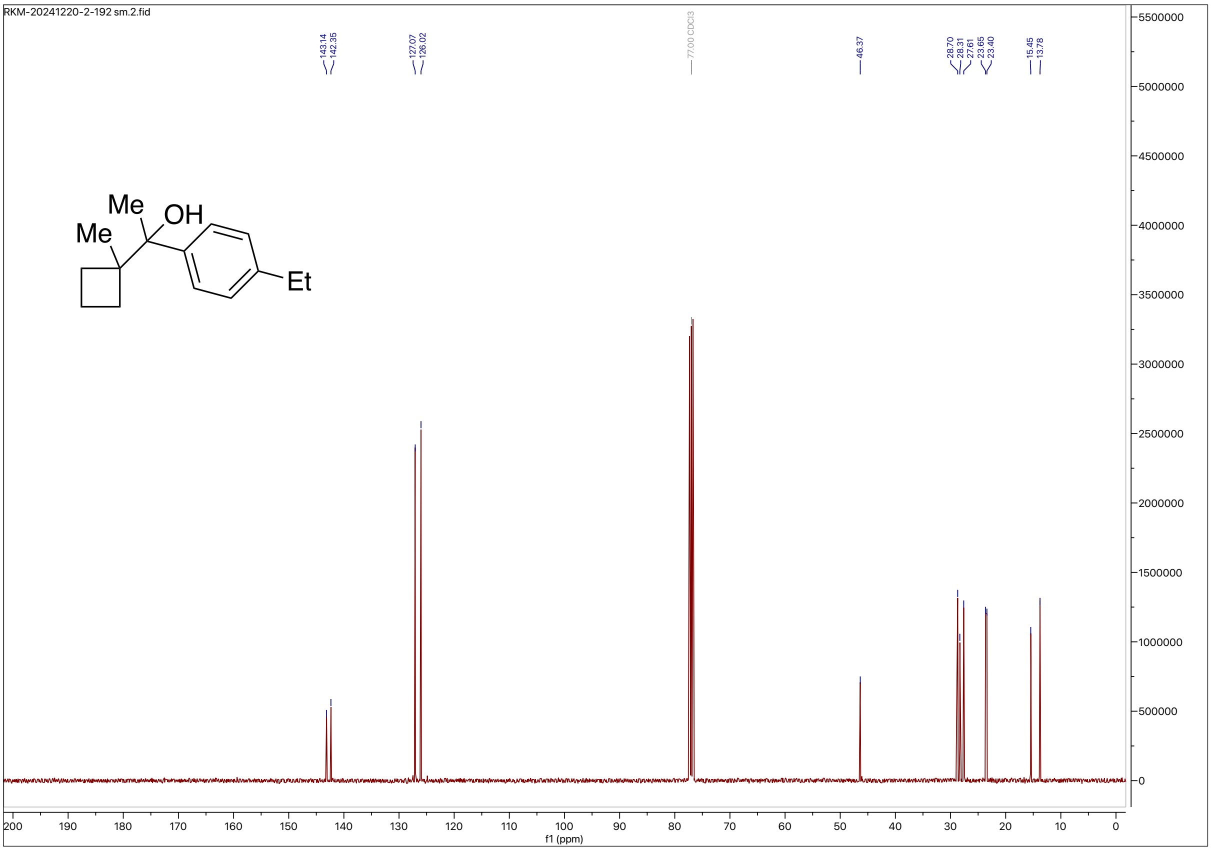

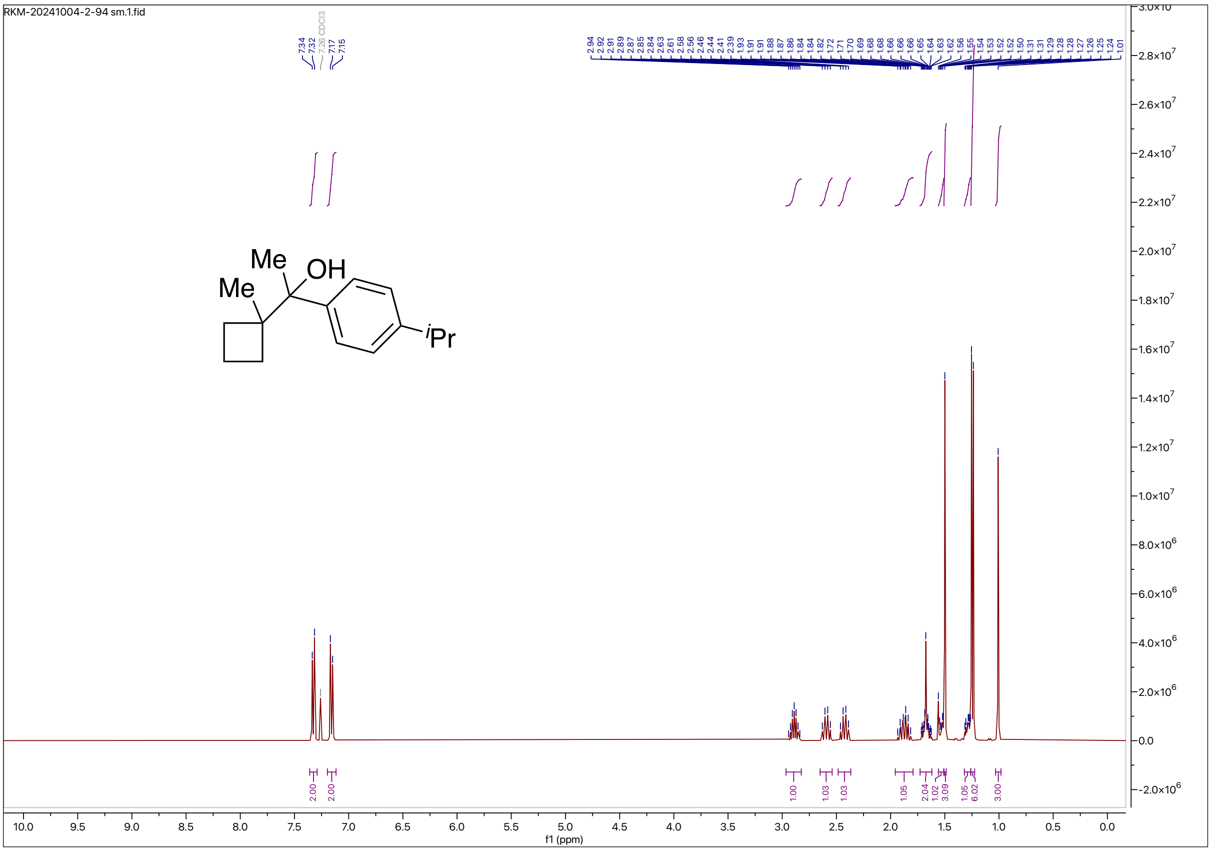

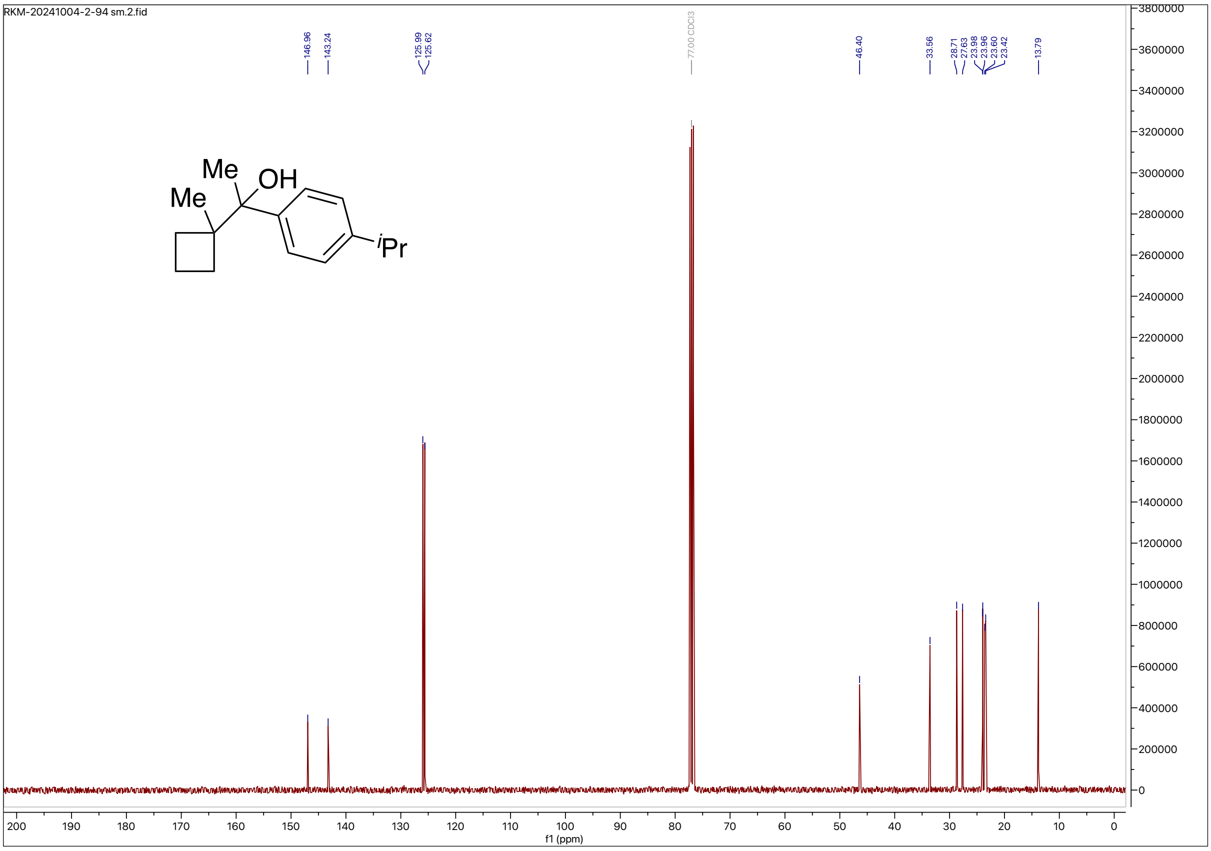

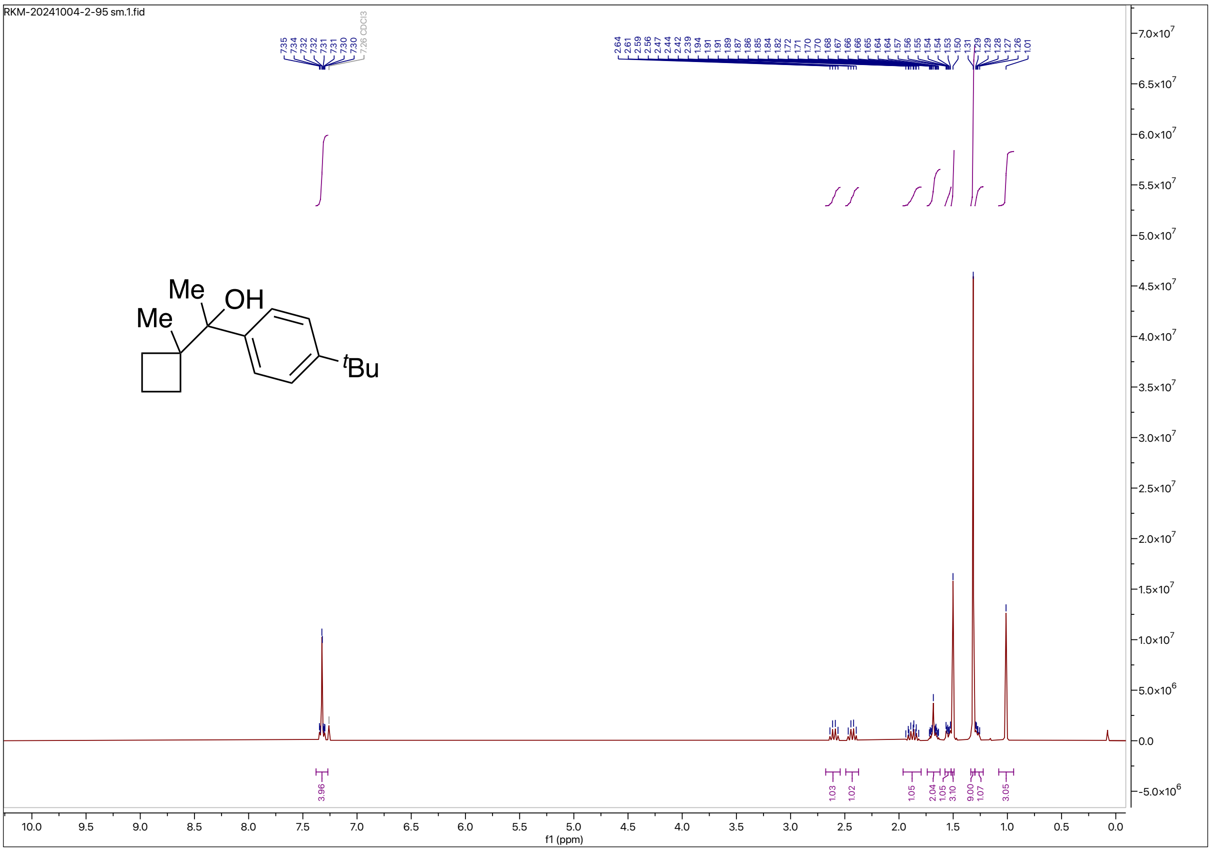
RKM-20241004-2-95 sm.1.fid
Me
OH
Me
tBu
f1 (ppm)

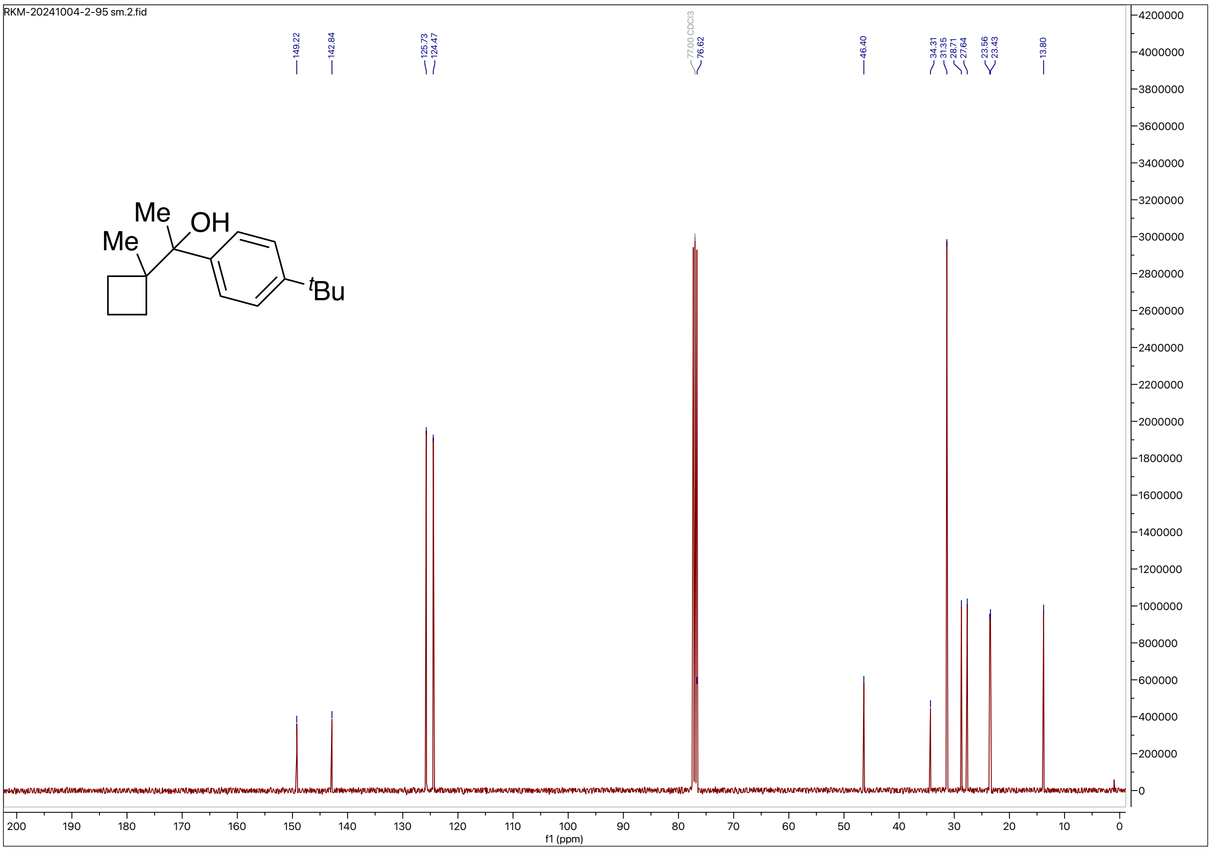
RKM-20241004-2-95 sm.2.fid
149.22
142.84
125.73
124.47
77.00 CDCl3
76.62
46.40
34.31
31.35
28.71
27.64
23.56
23.43
13.80
Me
OH
Me
tBu
f1 (ppm)

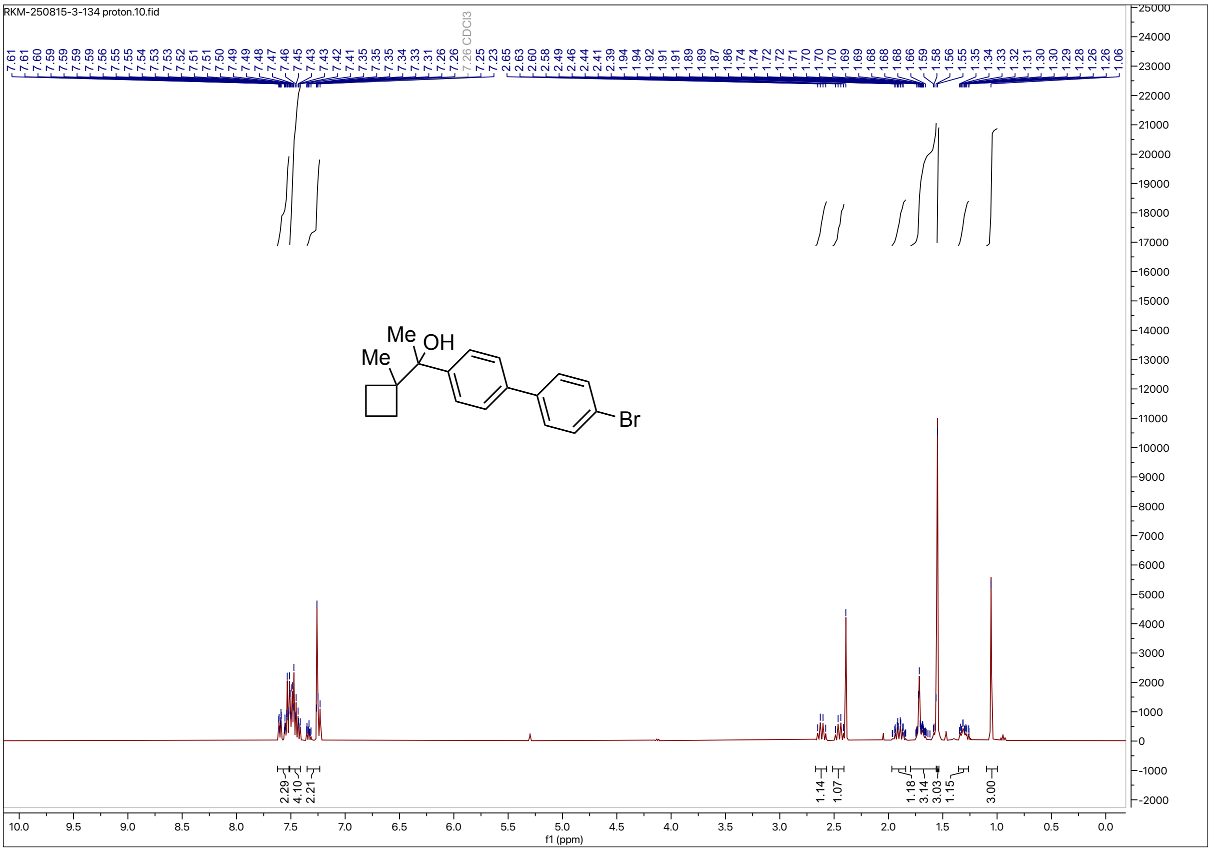
RKM-250815-3-134 proton.10.fid
Me
OH
Me
Br
f1 (ppm)

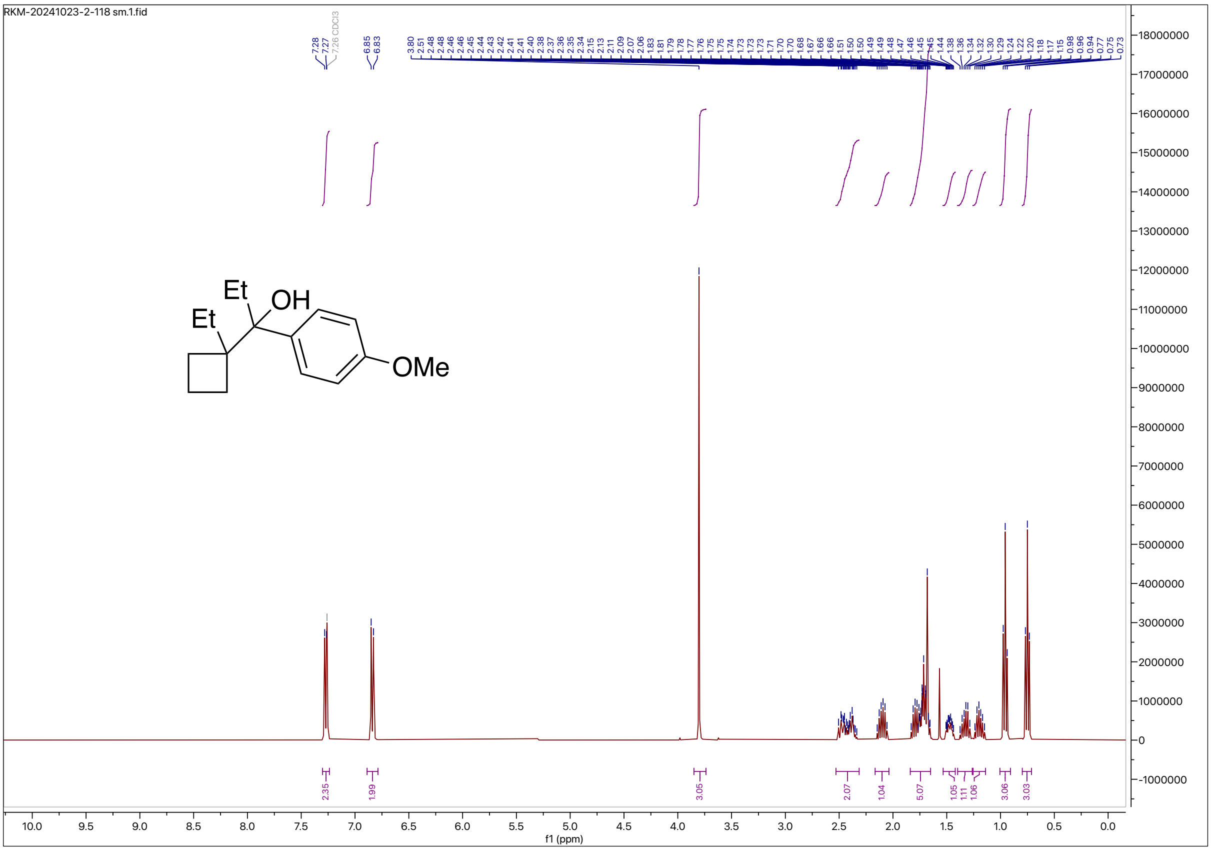
RKM-20241023-2-118 sm.1.fid
Et
OH
Et
OMe
f1 (ppm)

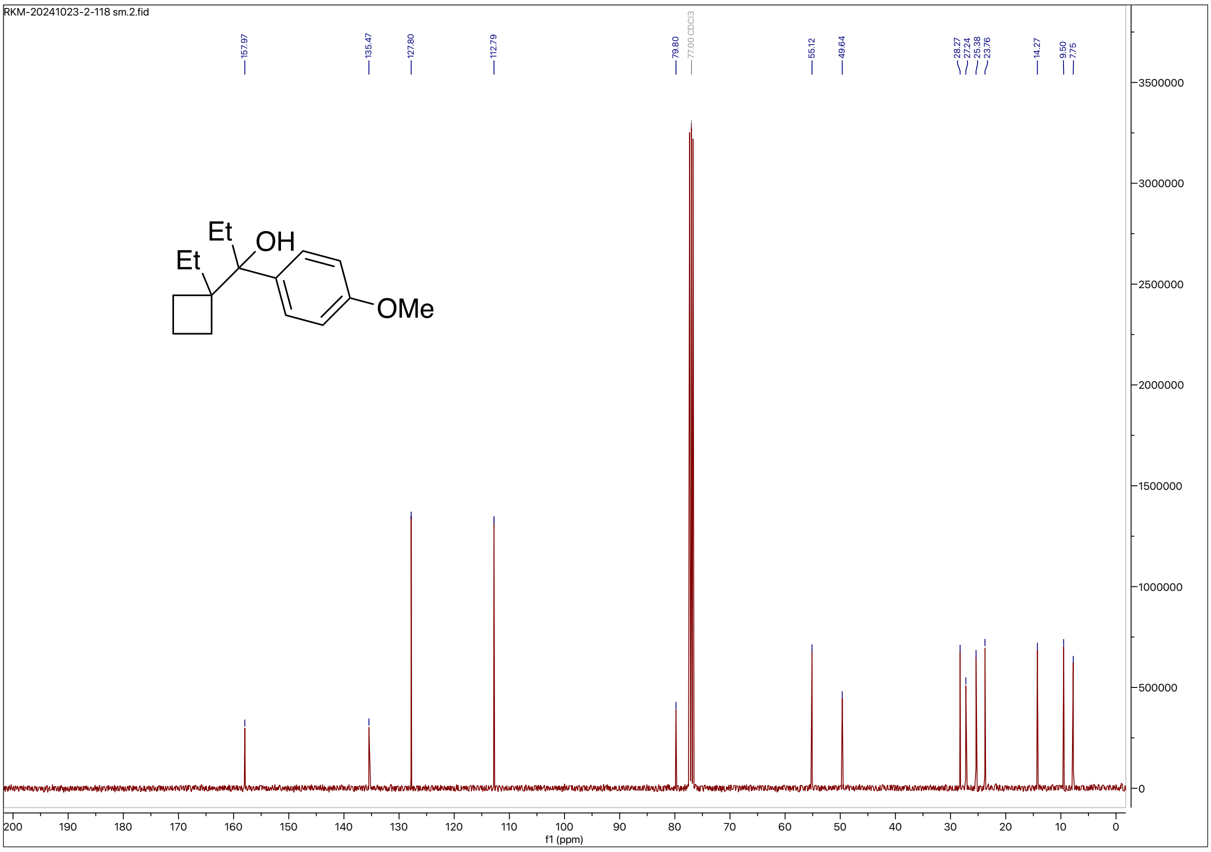
RKM-20241023-2-118 sm.2.fid
157.97
135.47
127.80
112.79
79.80
77.00 CDCl3
55.12
49.64
28.27
27.24
25.38
23.76
14.27
9.50
7.75
Et
OH
Et
OMe
f1 (ppm)

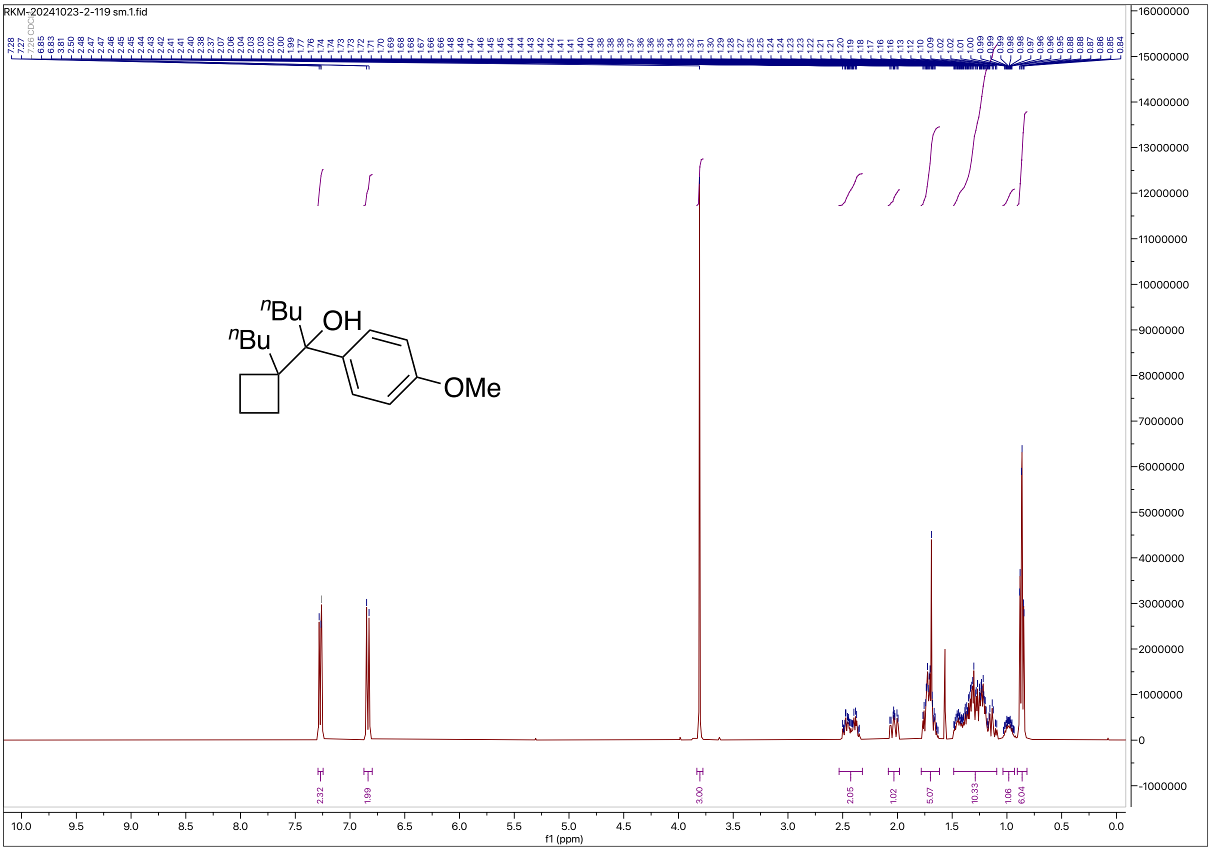
RKM-20241023-2-119 sm.1.fid
^{n}Bu
OH
^{n}Bu
OMe
2.32
1.99
3.00
2.05
1.02
5.07
10.33
1.06
6.04
f1 (ppm)

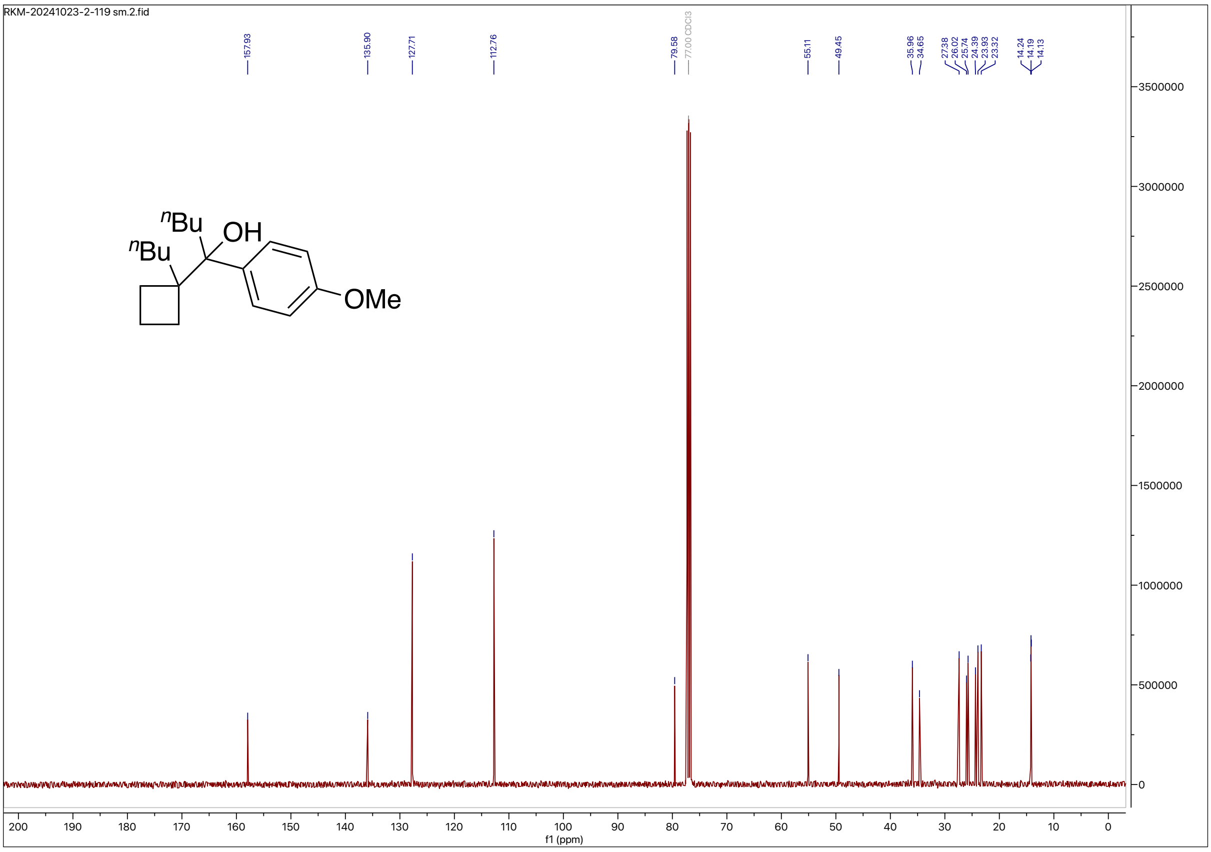
RKM-20241023-2-119 sm.2.fid
157.93
135.90
127.71
112.76
79.58
77.00 CDCl3
55.11
49.45
35.96
34.65
27.38
26.02
25.74
24.39
23.93
23.32
14.24
14.19
14.13
f1 (ppm)
OH
OMe

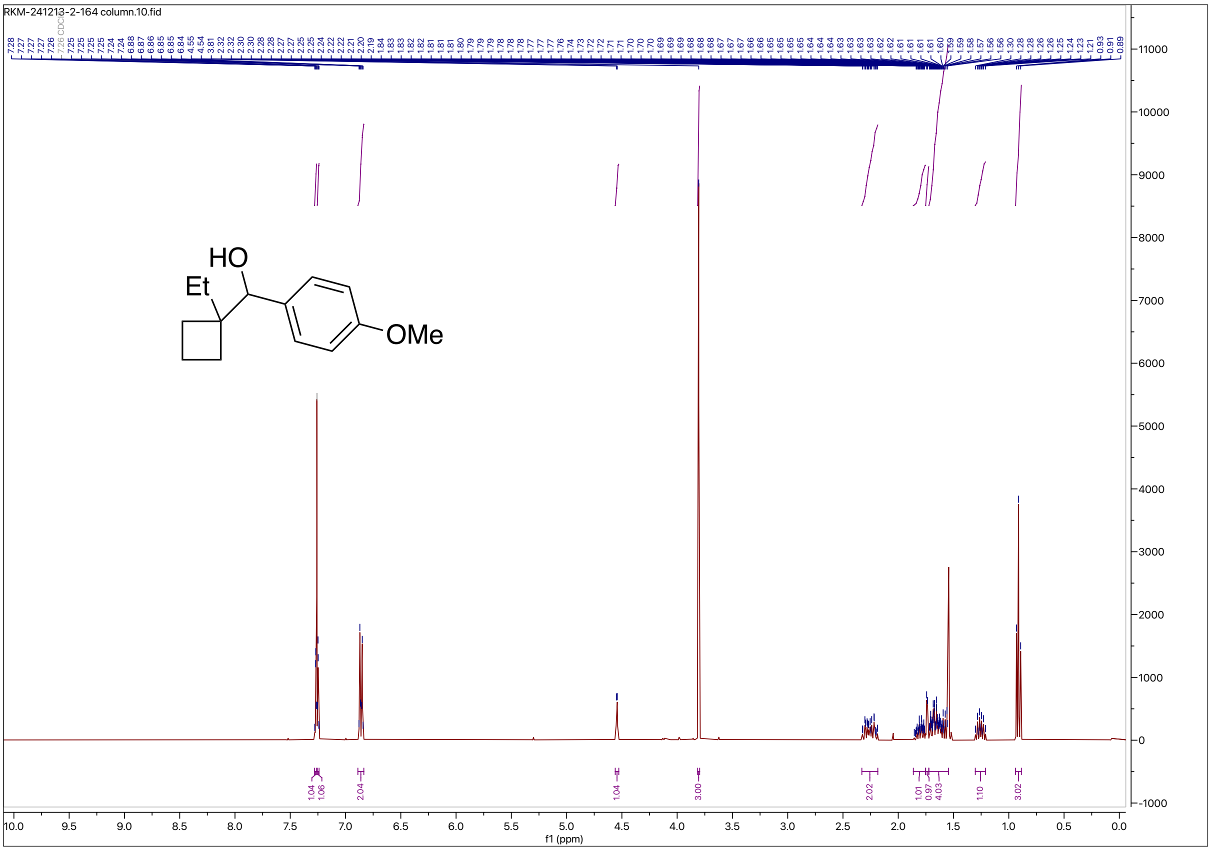
RKM-241213-2-164 column.10.fid
HO
Et
OMe
f1 (ppm)
1.04
1.06
2.04
1.04
3.00
2.02
1.01
0.97
4.03
1.10
3.02

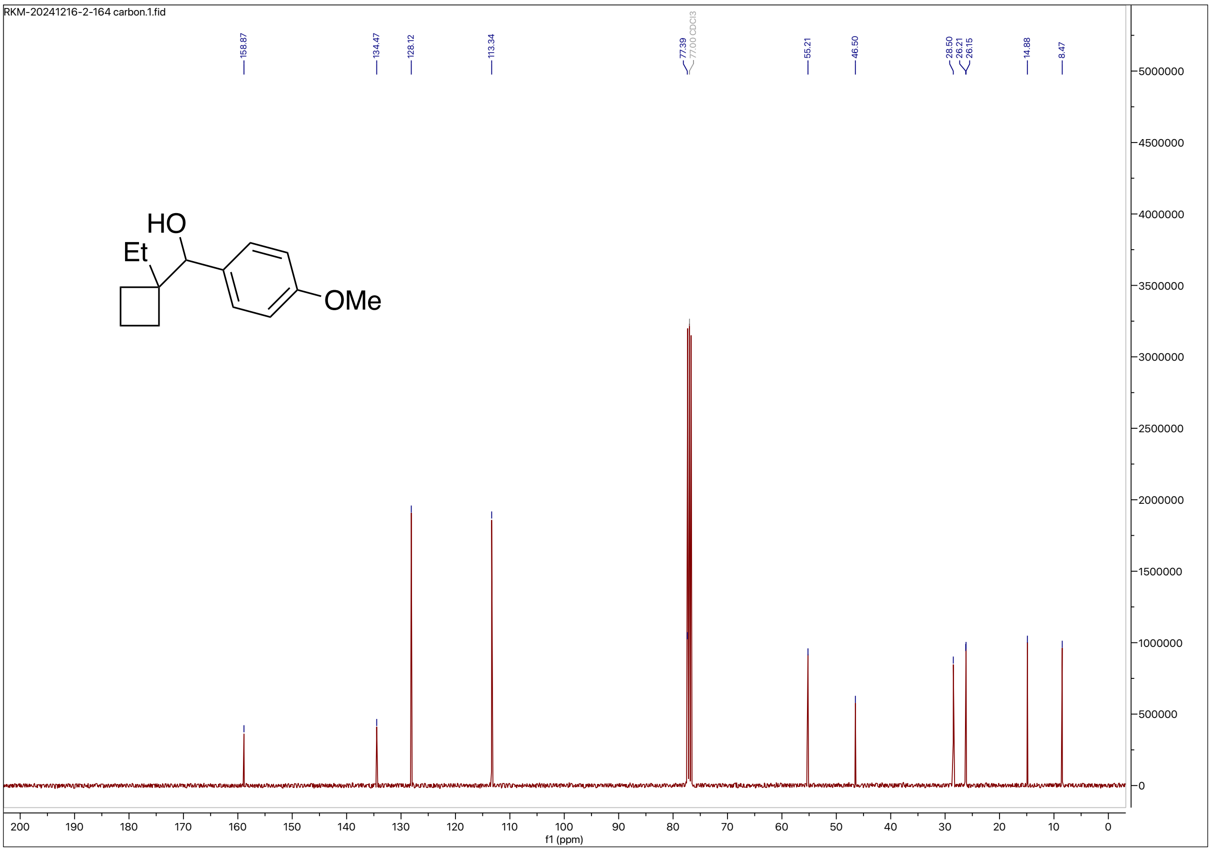

RKM-20241216-2-164 carbon.1.fid
158.87
134.47
128.12
113.34
77.39
77.00 CDCl3
55.21
46.50
28.50
26.21
26.15
14.88
8.47
HO
Et
OMe
f1 (ppm)

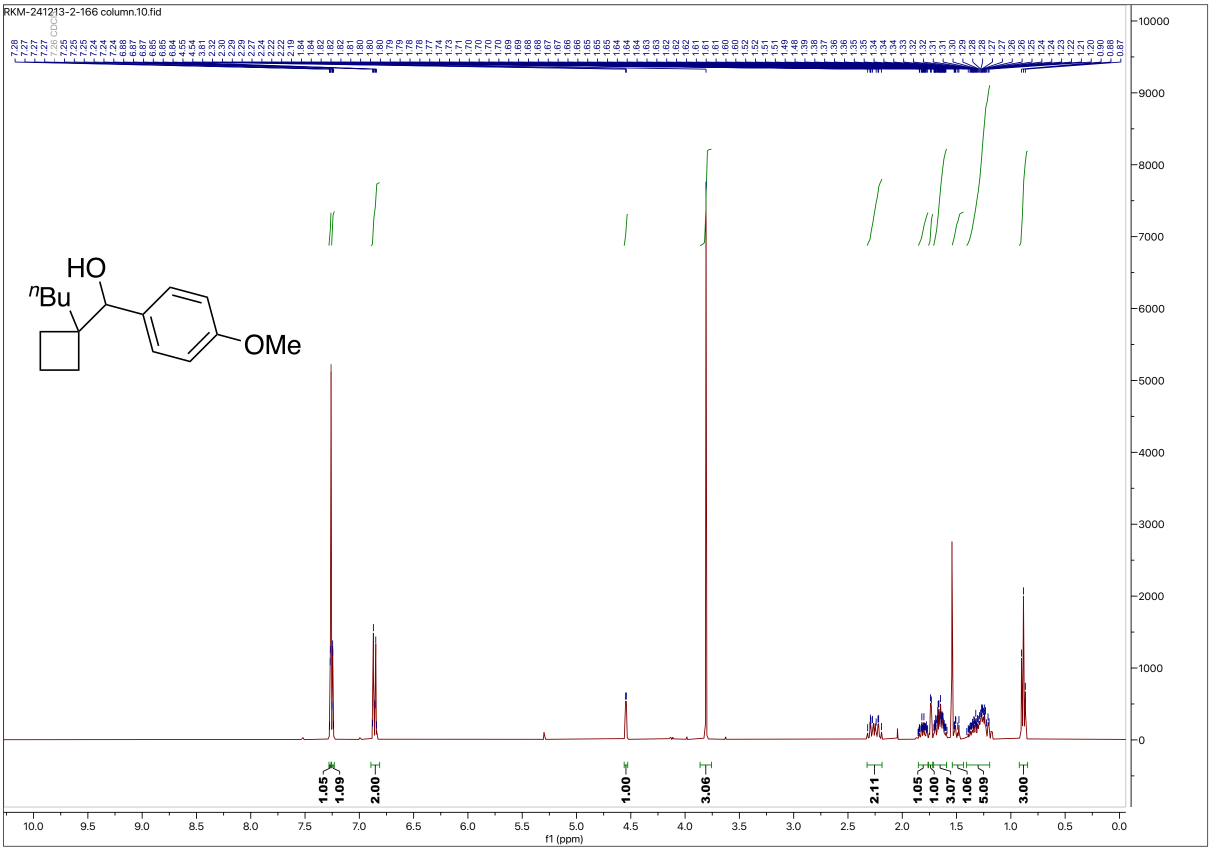

RKM-241213-2-166 column.10.fid
HO
nBu
OMe
1.05
1.09
2.00
1.00
3.06
2.11
1.05
1.00
3.07
1.06
5.09
3.00
f1 (ppm)

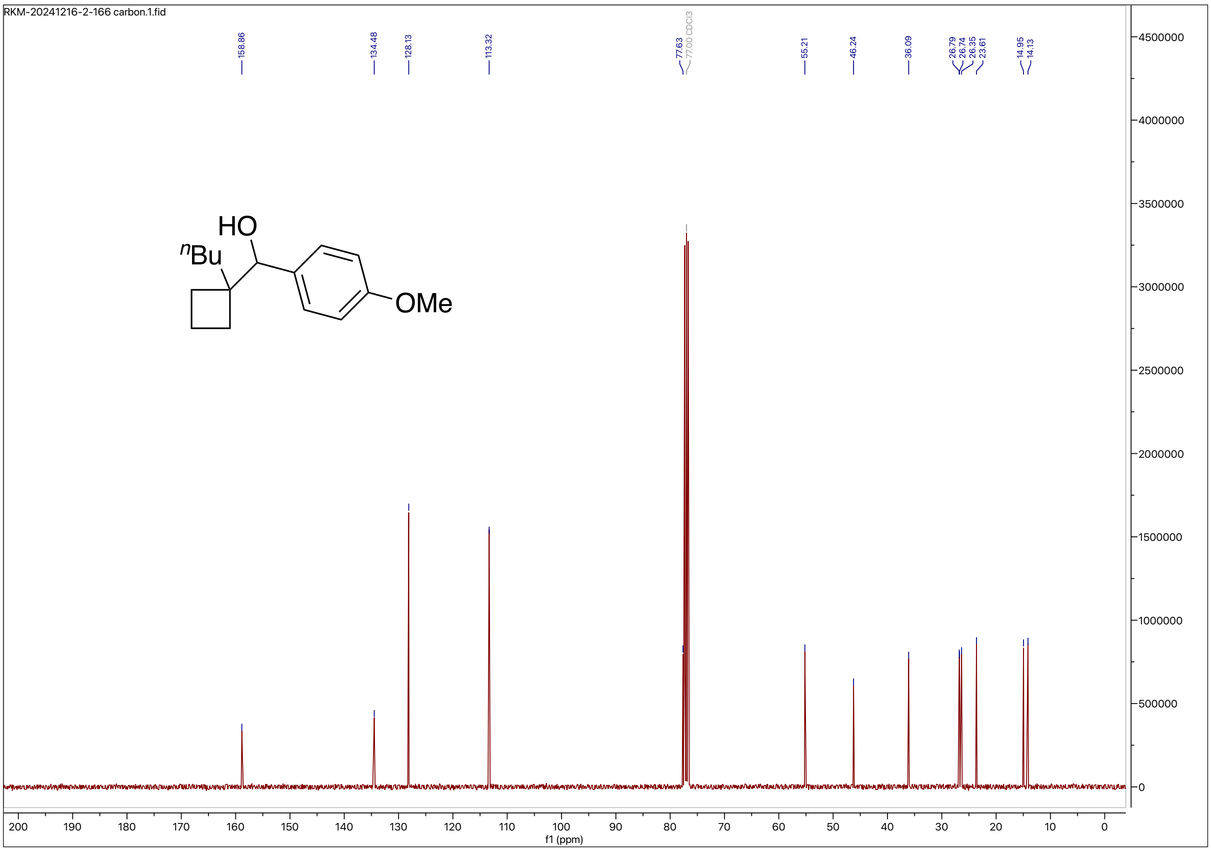
RKM-20241216-2-166 carbon.1.fid
158.86
134.48
128.13
113.32
77.63
77.00 CDCl3
55.21
46.24
36.09
26.79
26.74
26.35
23.61
14.95
14.13
HO
nBu
OMe
f1 (ppm)

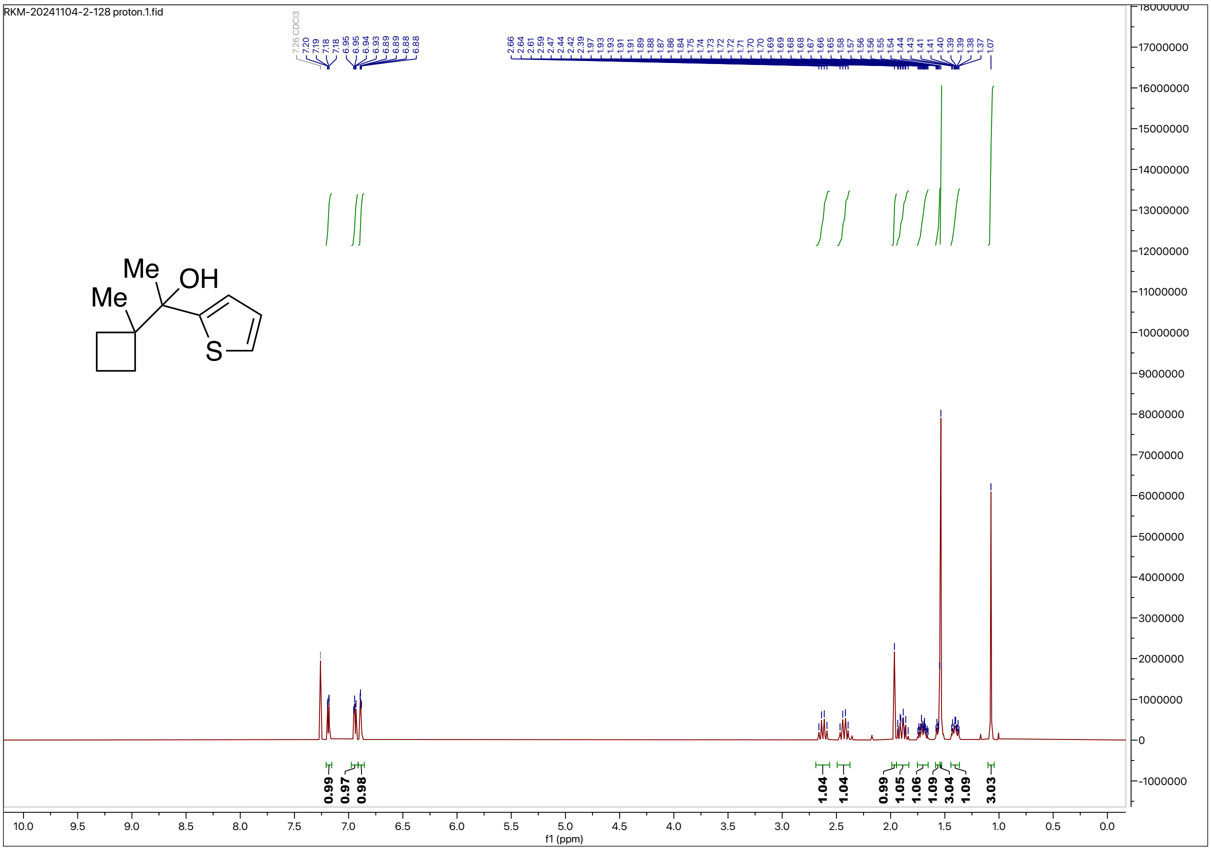
RKM-20241104-2-128 proton.1.fid
Me
Me
OH
S
0.99
0.97
0.98
1.04
1.04
0.99
1.05
1.06
1.09
3.04
1.09
3.03
f1 (ppm)

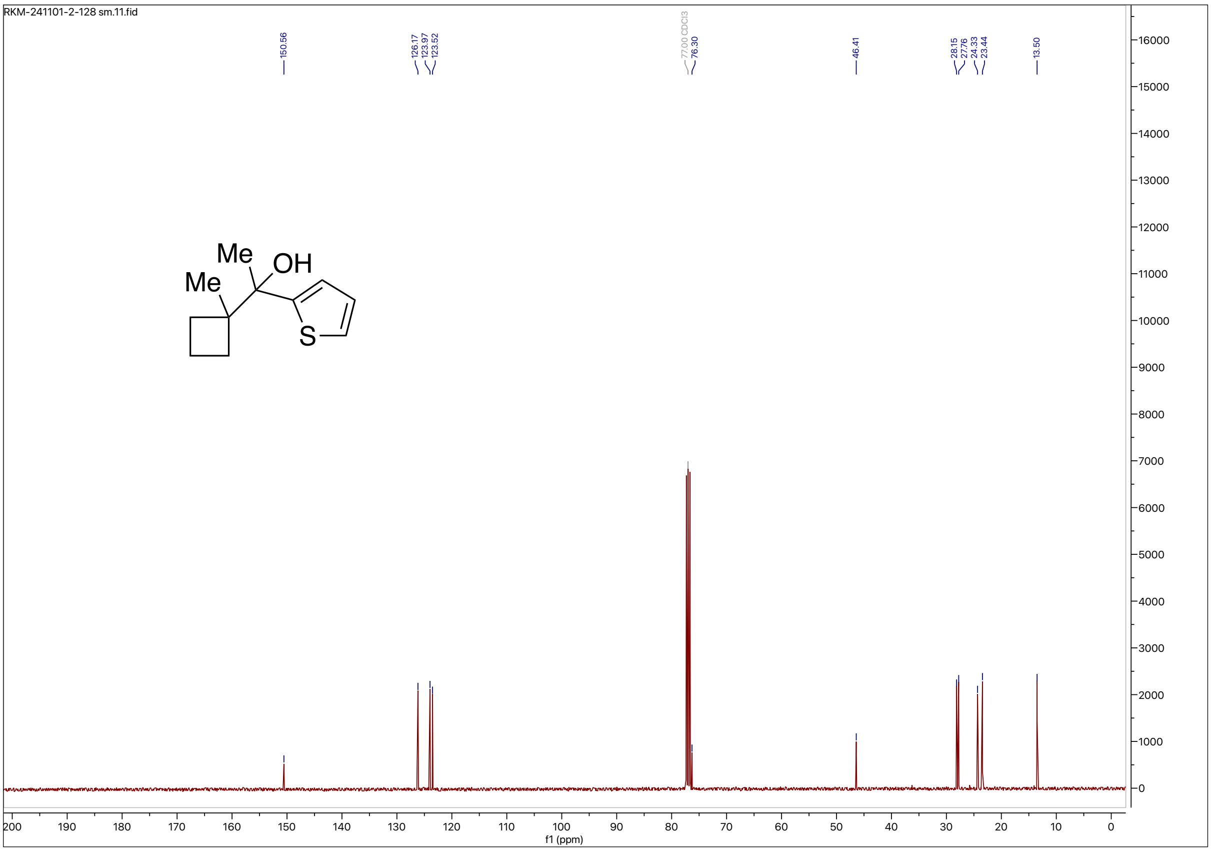
RKM-241101-2-128 sm.11.fid
150.56
126.17
123.97
123.52
77.00 CDCl3
76.30
46.41
28.15
27.76
24.33
23.44
13.50
Me
OH
Me
S
f1 (ppm)

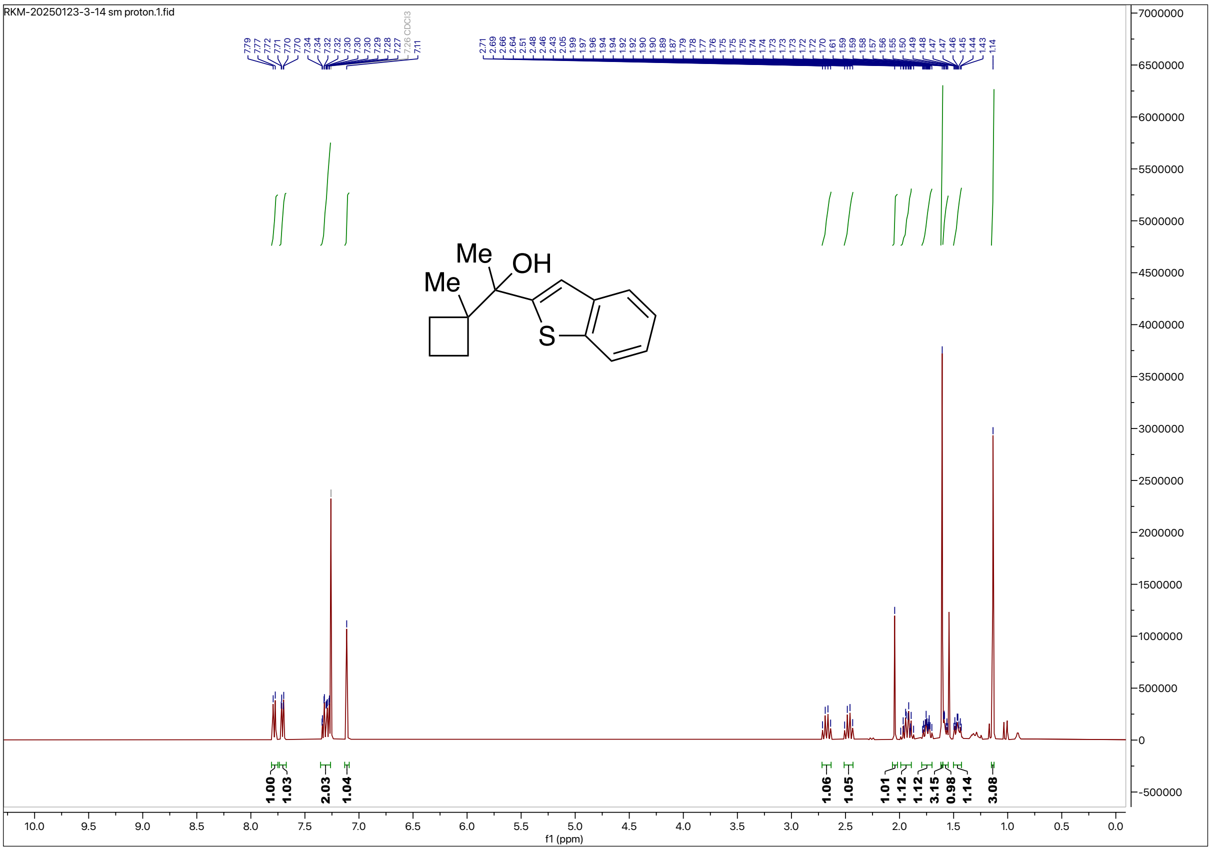
RKM-20250123-3-14 sm proton.1.fid
7.26 CDCl3
Me
OH
Me
S
1.00
1.03
2.03
1.04
1.06
1.05
1.01
1.12
1.12
3.15
0.98
1.14
3.08
f1 (ppm)

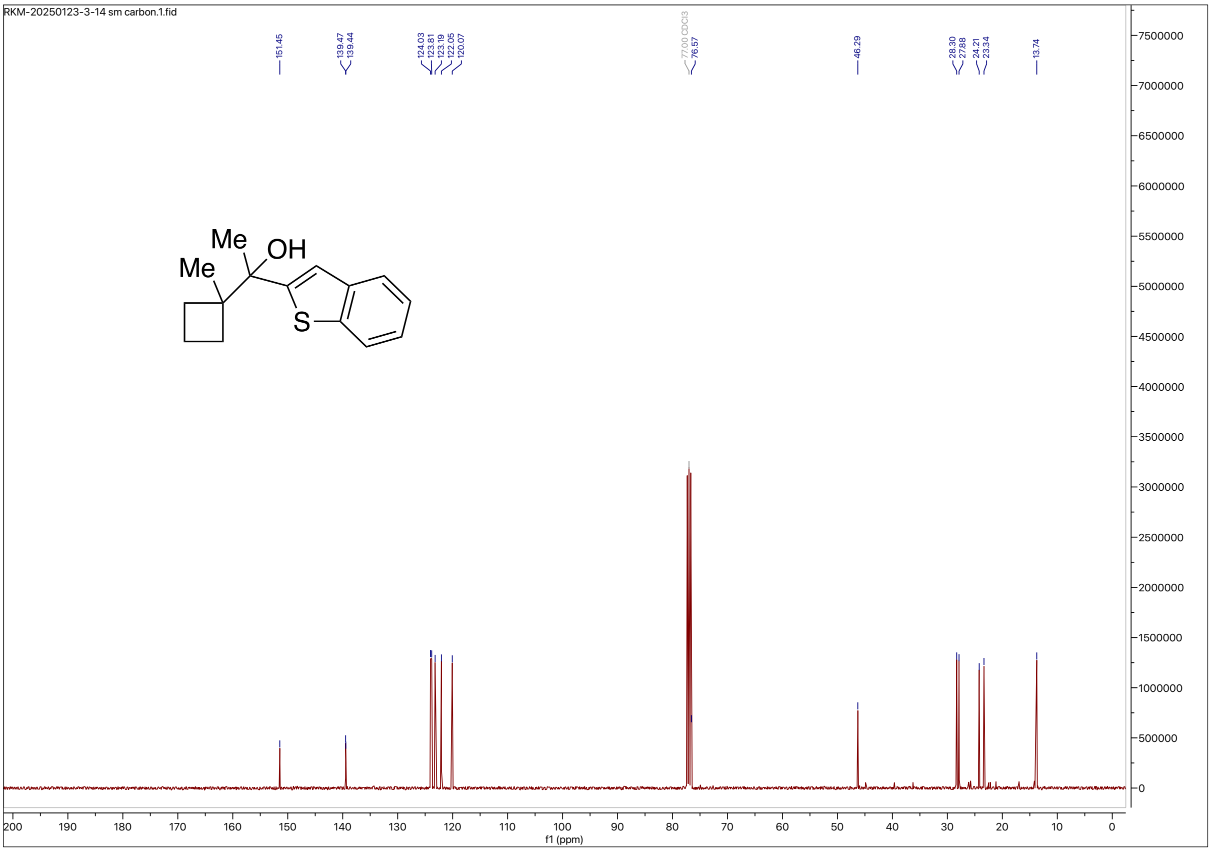
RKM-20250123-3-14 sm carbon.1.fid
Me
OH
Me
S
f1 (ppm)

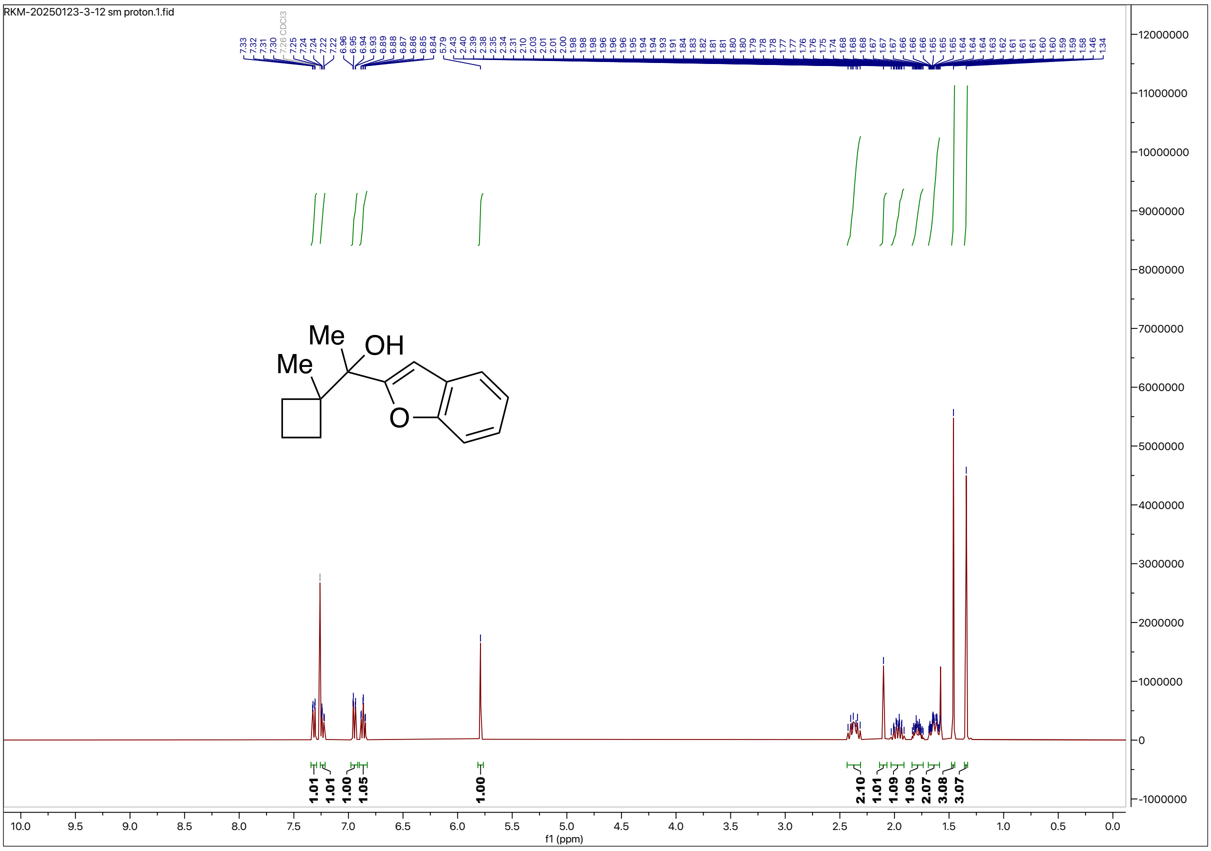
RKM-20250123-3-12 sm proton.1.fid
Me
OH
Me
O
f1 (ppm)

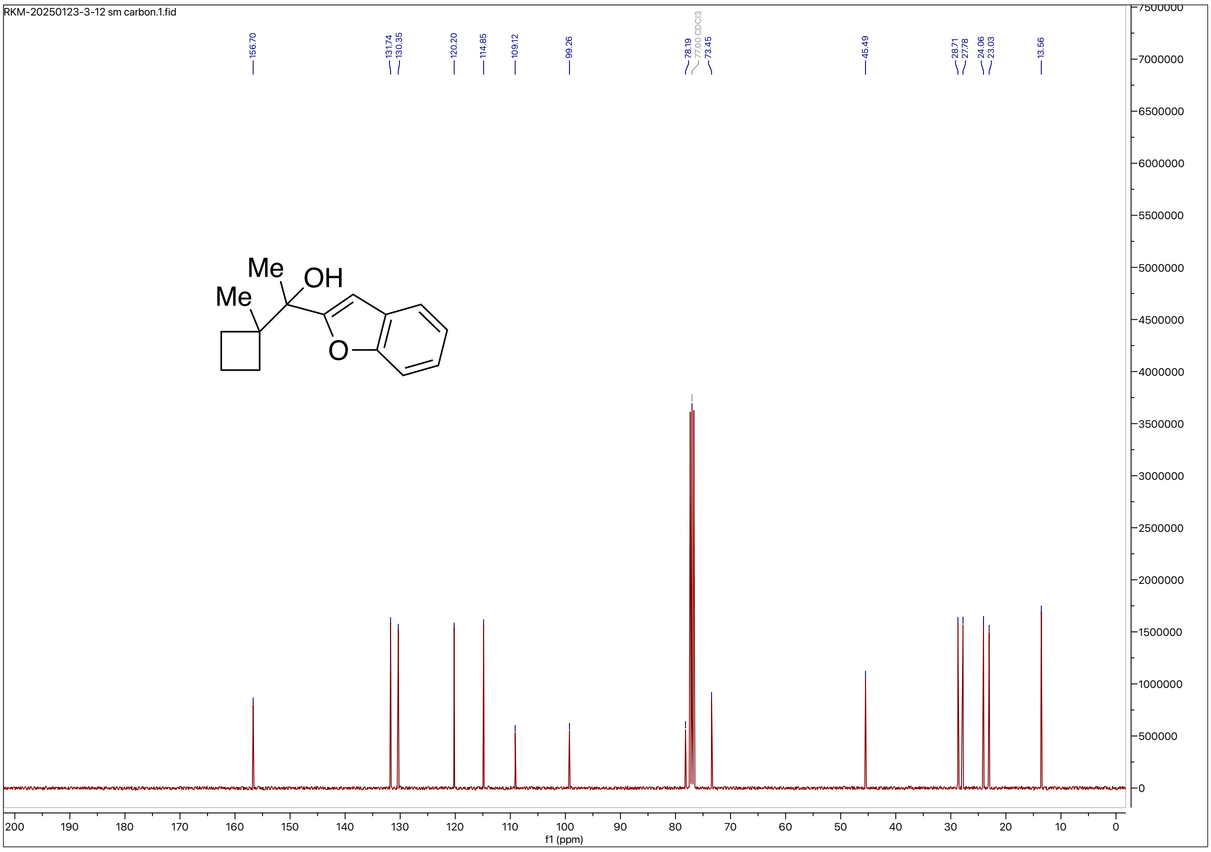

RKM-20250123-3-12 sm carbon.1.fid
156.70
131.74
130.35
120.20
114.85
109.12
99.26
78.19
77.00 CDCl3
73.45
45.49
28.71
27.78
24.06
23.03
13.56
Me
Me
OH
O
f1 (ppm)

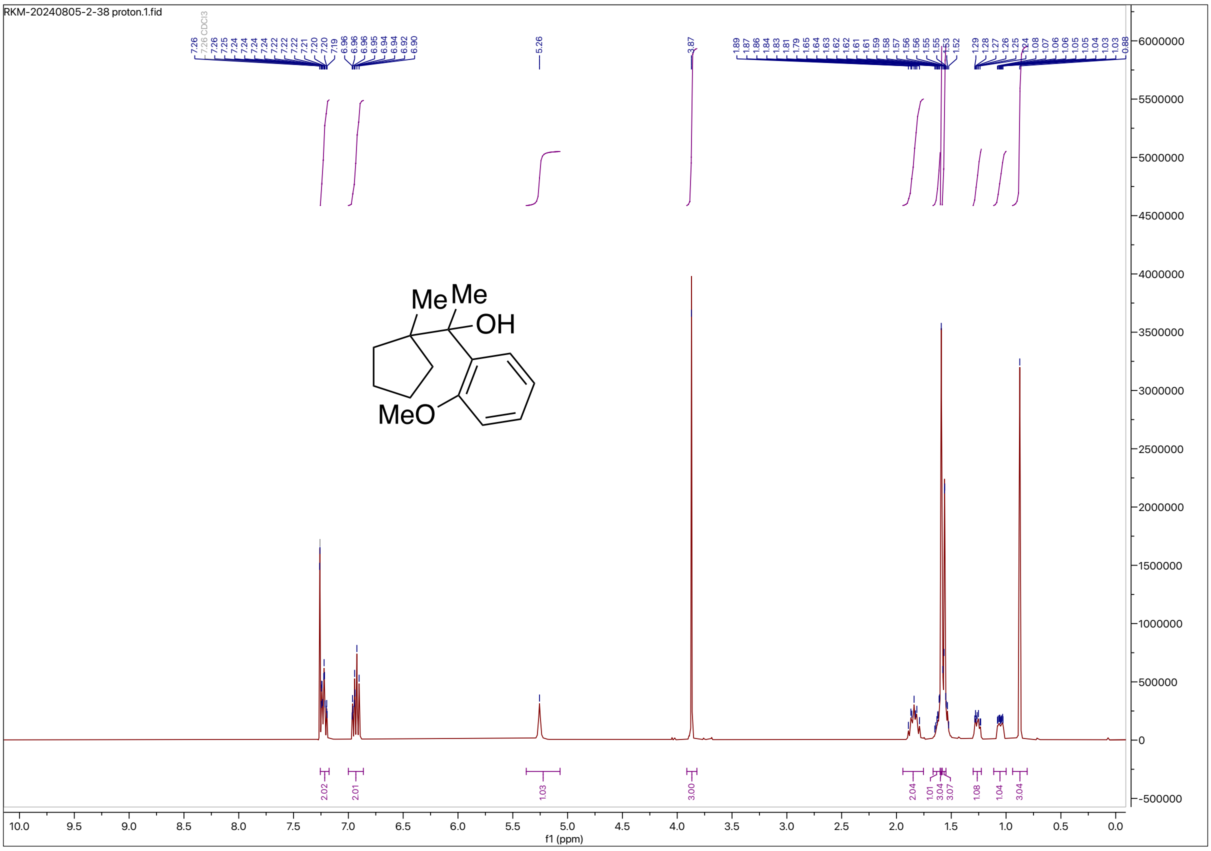

RKM-20240805-2-38 proton.1.fid
5.26
3.87
Me
Me
OH
MeO
2.02
2.01
1.03
3.00
2.04
1.01
3.04
3.07
1.08
1.04
3.04
f1 (ppm)

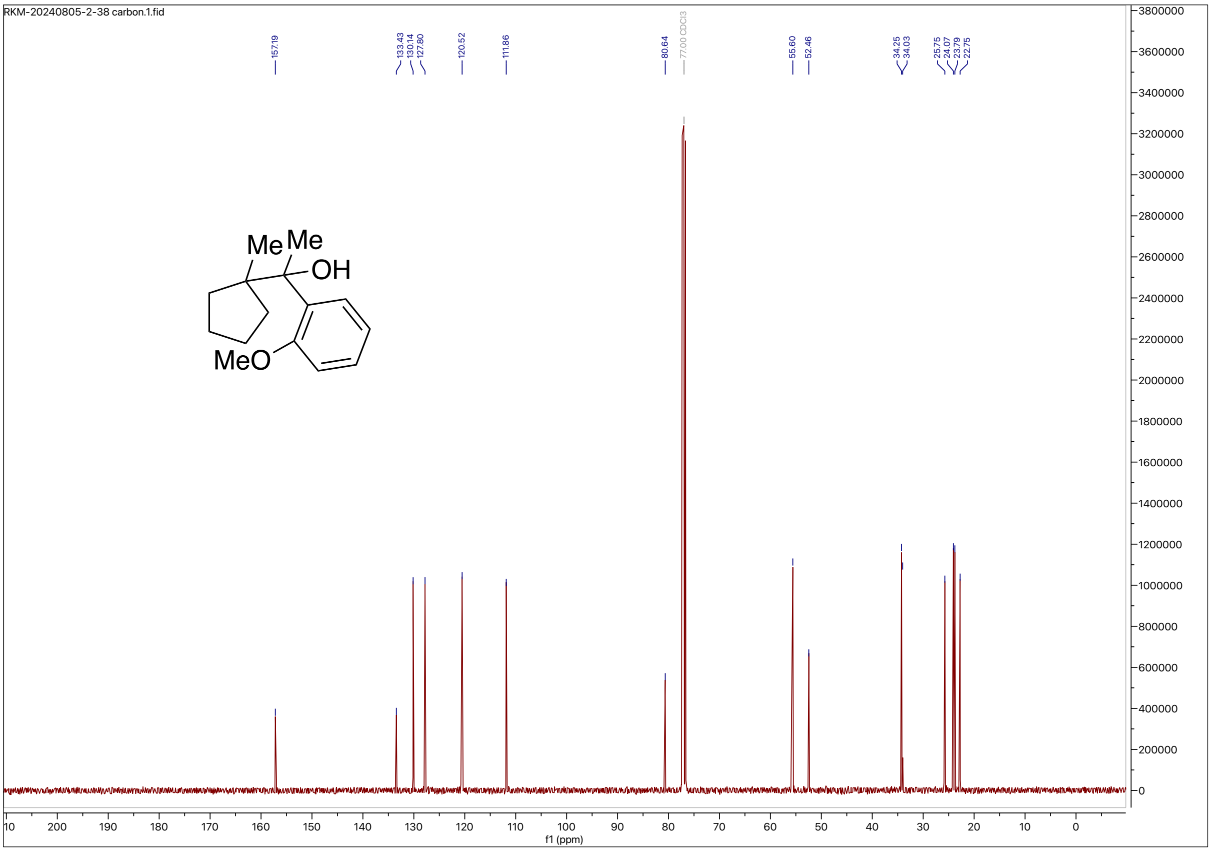

RKM-20240805-2-38 carbon.1.fid
157.19
133.43
130.14
127.80
120.52
111.86
80.64
77.00 CDCl3
55.60
52.46
34.25
34.03
25.75
24.07
23.79
22.75
Me
Me
OH
MeO
f1 (ppm)

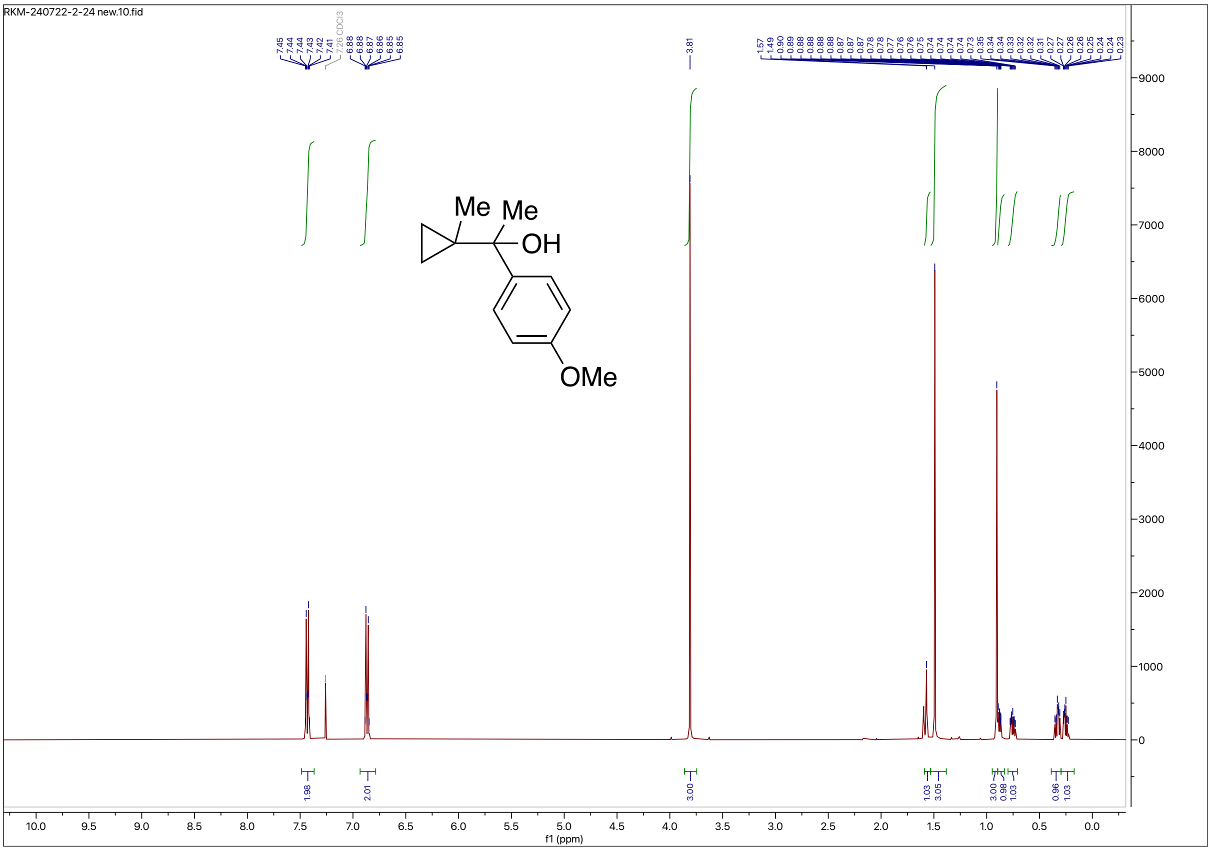

RKM-240722-2-24 new.10.fid
7.45
7.44
7.44
7.43
7.42
7.41
7.26 CDCl3
6.88
6.88
6.87
6.86
6.85
6.85
3.81
Me
Me
OH
OMe
1.98
2.01
3.00
1.03
3.05
3.00
0.98
1.03
0.96
1.03
f1 (ppm)

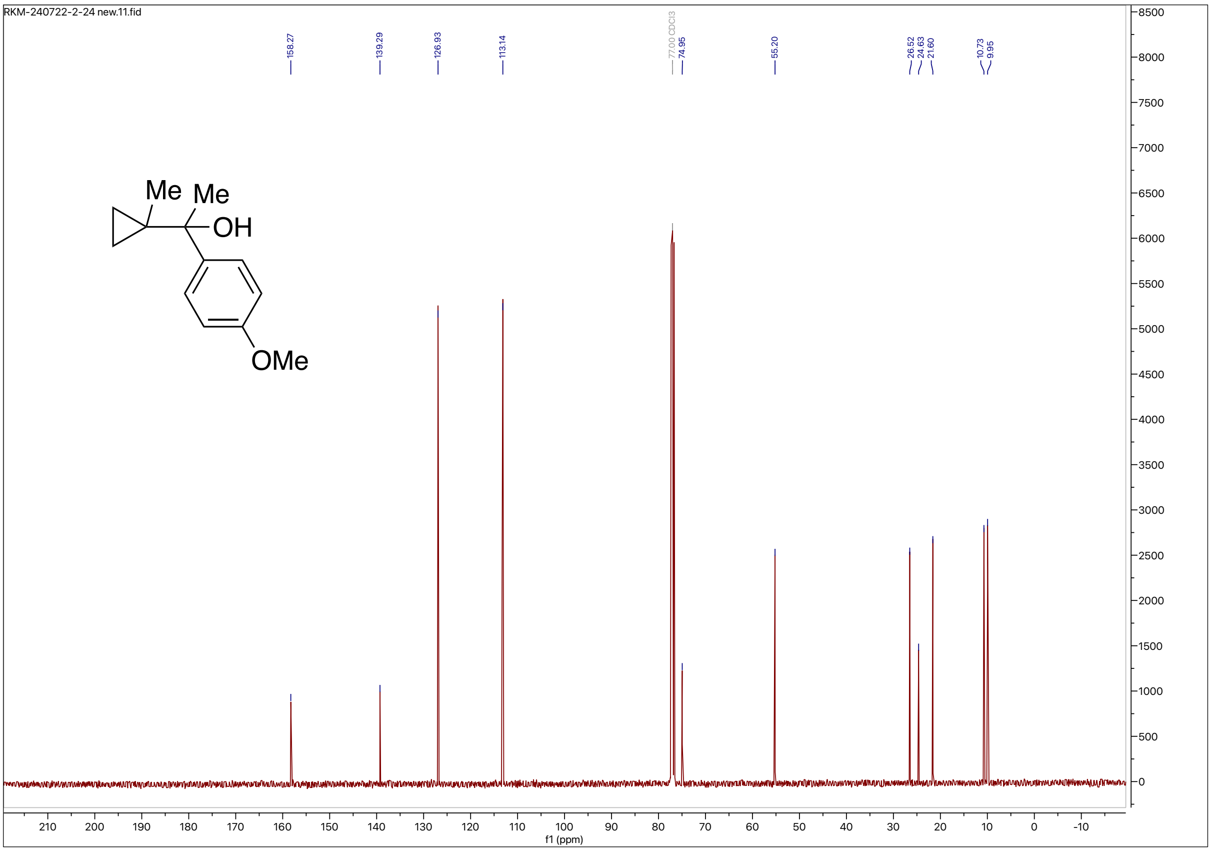
RKM-240722-2-24 new.11.fid
158.27
139.29
126.93
113.14
77.00 CDCl3
74.95
55.20
26.52
24.63
21.60
10.73
9.95
Me
Me
OH
OMe
f1 (ppm)

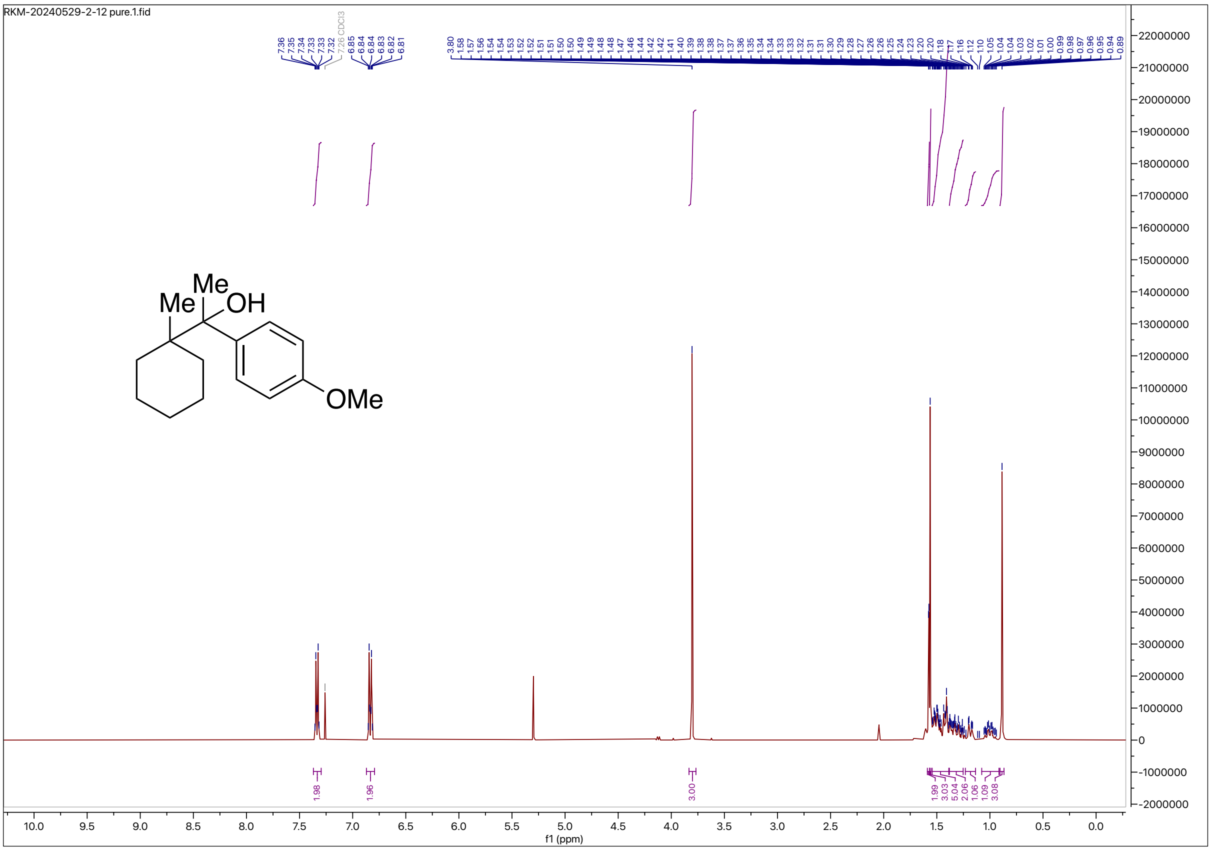
RKM-20240529-2-12 pure.1.fid
7.26 CDCl3
Me
Me
OH
OMe
1.98
1.96
3.00
f1 (ppm)

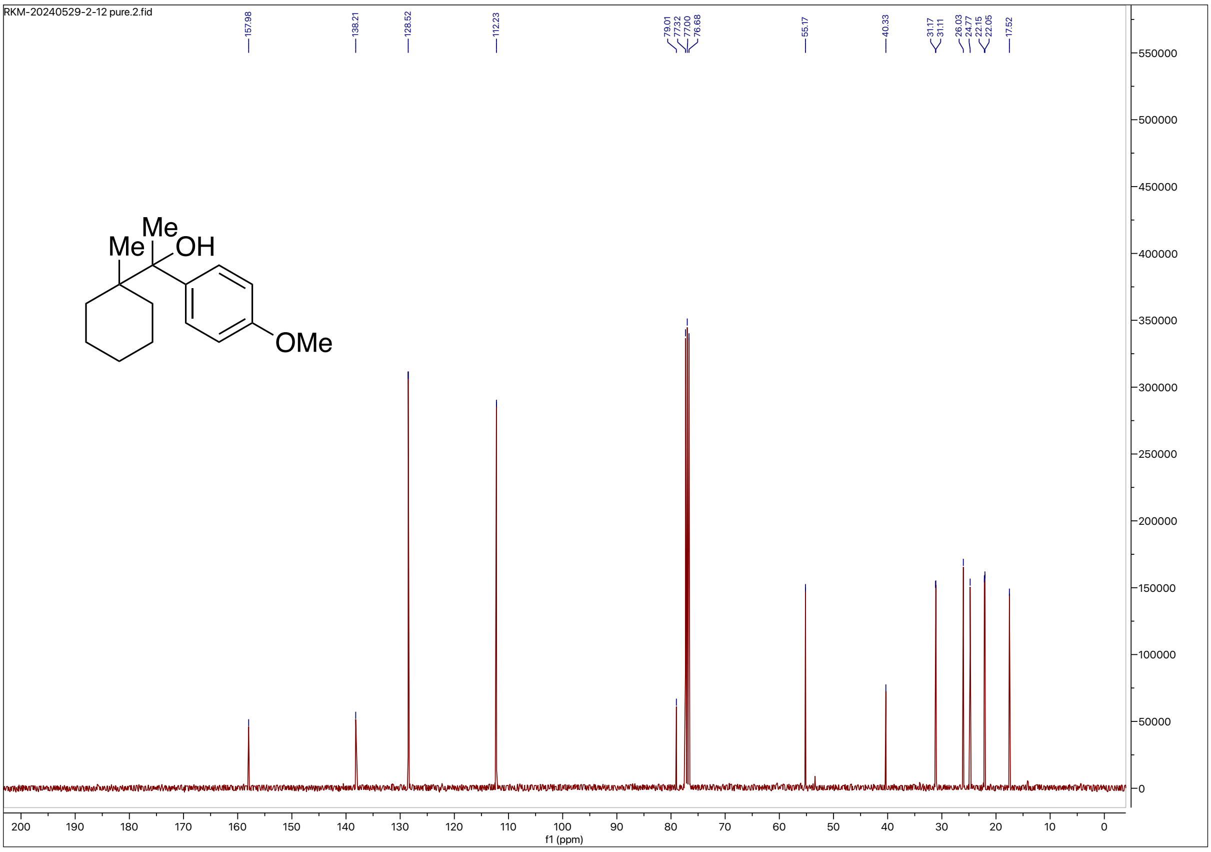
RKM-20240529-2-12 pure.2.fid
157.98
138.21
128.52
112.23
79.01
77.32
77.00
76.68
55.17
40.33
31.17
31.11
26.03
24.77
22.15
22.05
17.52
Me
Me
OH
OMe
f1 (ppm)

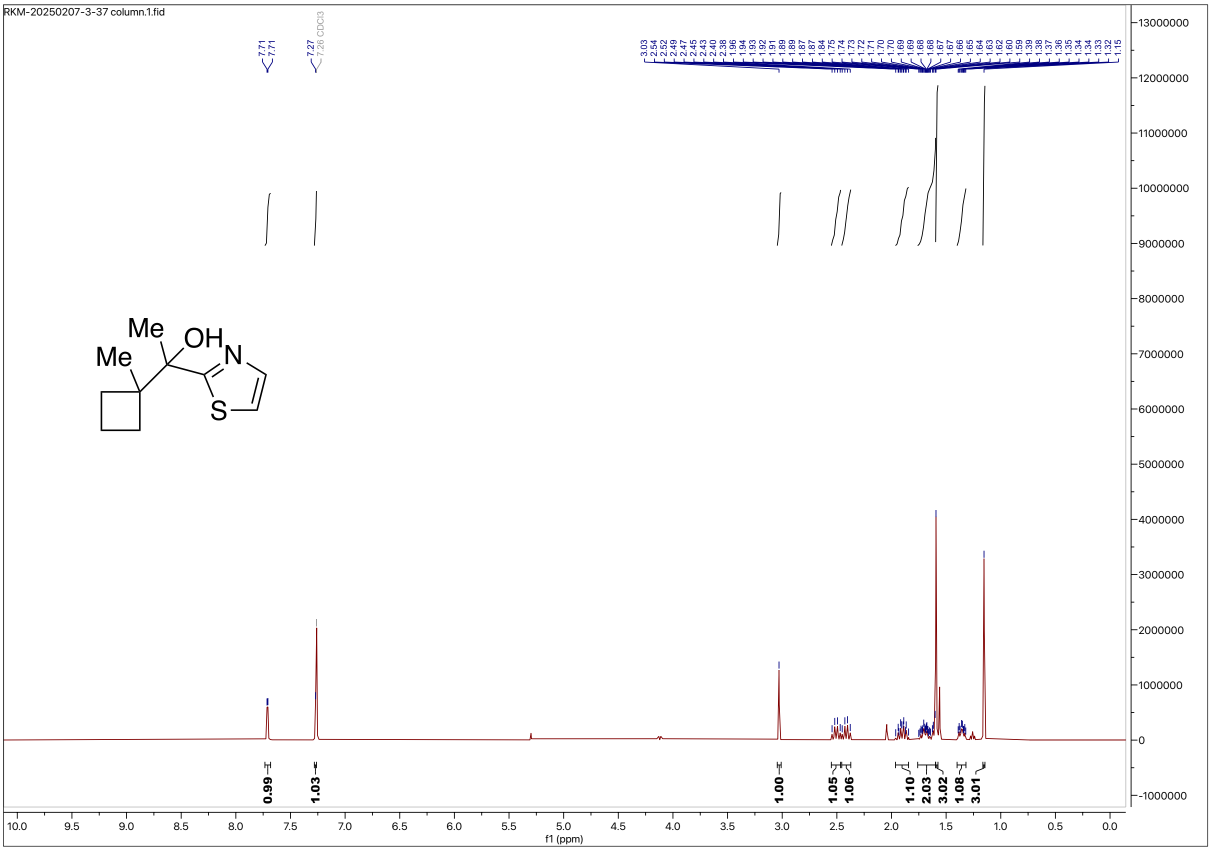
RKM-20250207-3-37 column.1.fid
7.71
7.71
7.27
7.26 CDCl3
Me
Me
OH
N
S
0.99
1.03
1.00
1.05
1.06
1.10
2.03
3.02
1.08
3.01
f1 (ppm)

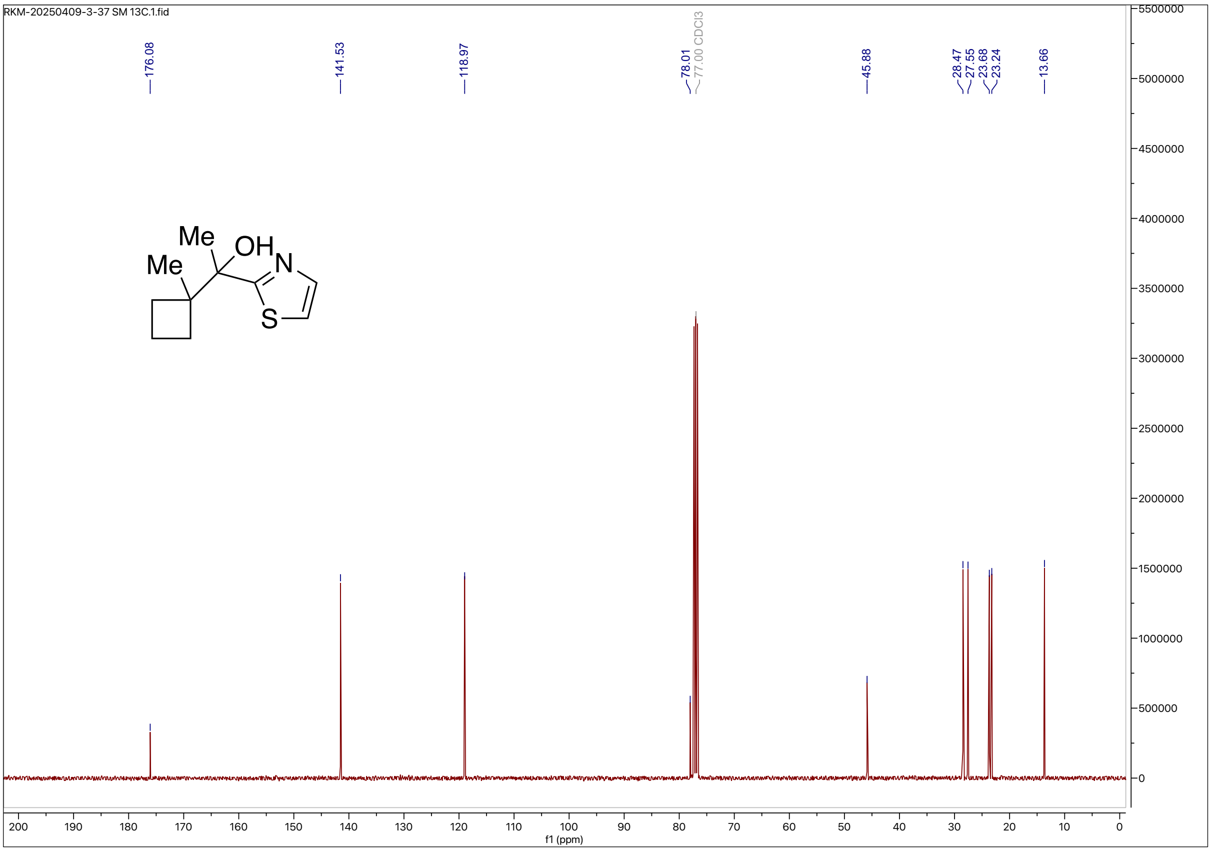
RKM-20250409-3-37 SM 13C.1.fid
176.08
141.53
118.97
78.01
77.00 CDCl3
45.88
28.47
27.55
23.68
23.24
13.66
Me
Me
OH
N
S
f1 (ppm)

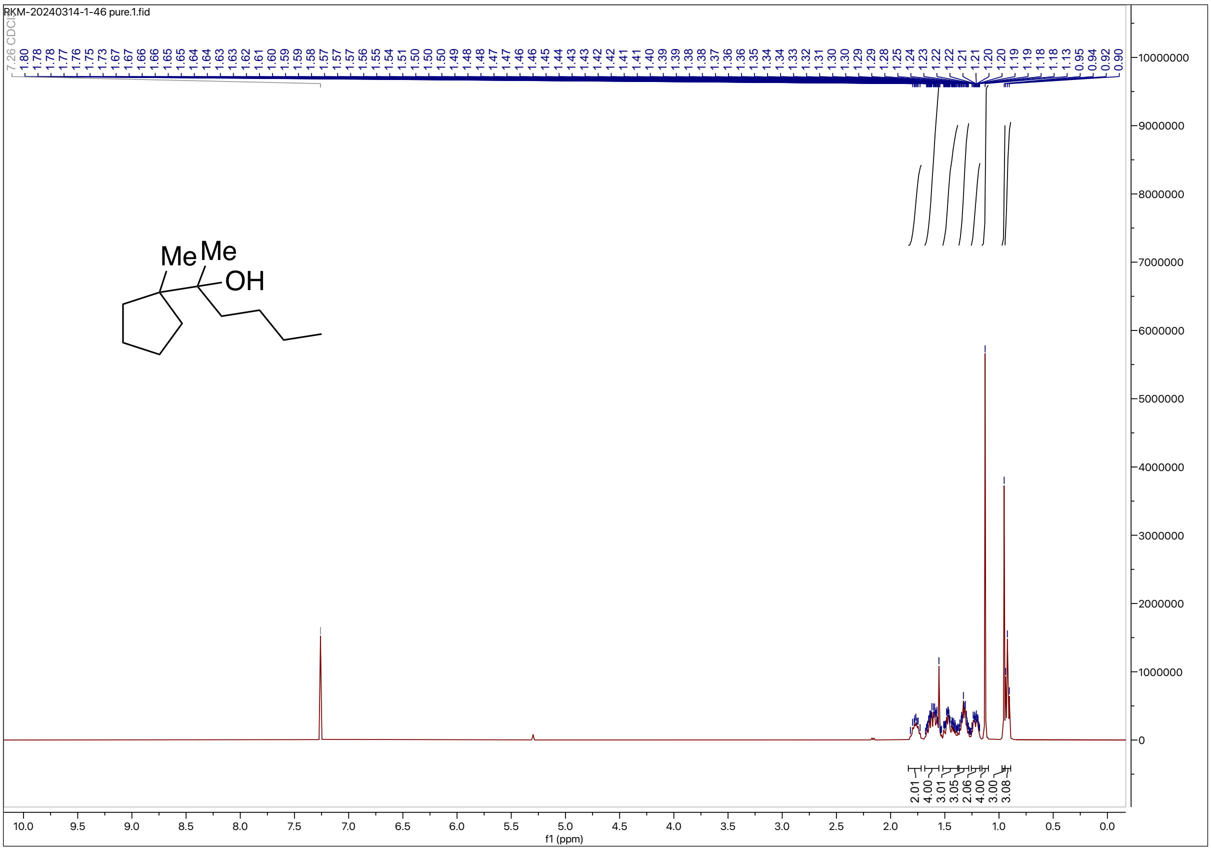
RKM-20240314-1-46 pure.1.fid
7.26 CDCl3
1.80 1.78 1.78 1.77 1.76 1.75 1.73 1.67 1.67 1.66 1.65 1.65 1.64 1.64 1.63 1.63 1.62 1.61 1.60 1.59 1.59 1.58 1.57 1.57 1.56 1.55 1.54 1.51 1.50 1.50 1.50 1.49 1.48 1.48 1.47 1.47 1.46 1.46 1.45 1.44 1.43 1.43 1.42 1.42 1.41 1.41 1.40 1.39 1.39 1.38 1.38 1.37 1.36 1.36 1.35 1.34 1.34 1.33 1.32 1.31 1.30 1.30 1.29 1.29 1.28 1.25 1.24 1.23 1.22 1.22 1.21 1.21 1.20 1.20 1.19 1.19 1.18 1.18 1.13 0.95 0.94 0.92 0.90
Me
Me
OH
2.01
4.00
3.01
3.05
2.06
4.00
3.00
3.08
f1 (ppm)

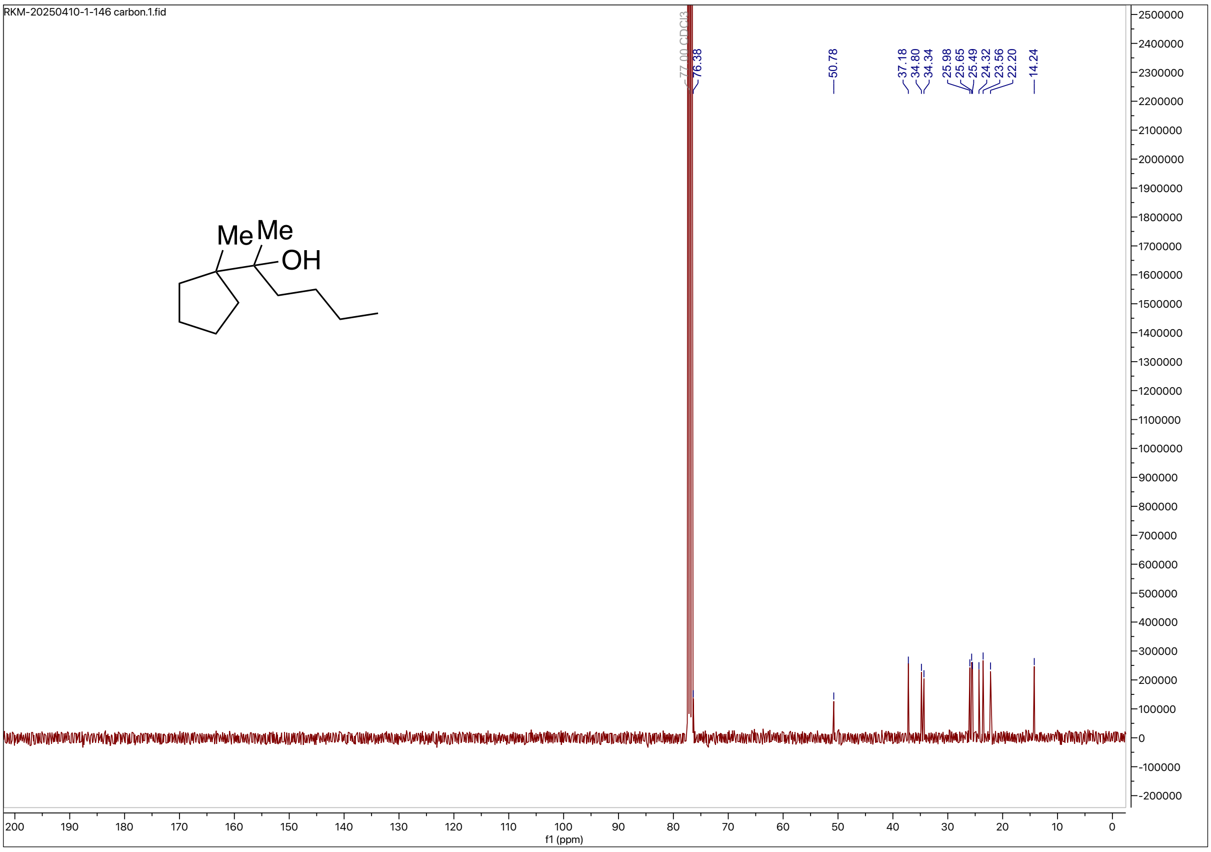
RKM-20250410-1-146 carbon.1.fid
77.00 CDCl3
76.38
50.78
37.18
34.80
34.34
25.98
25.65
25.49
24.32
23.56
22.20
14.24
Me
Me
OH
f1 (ppm)

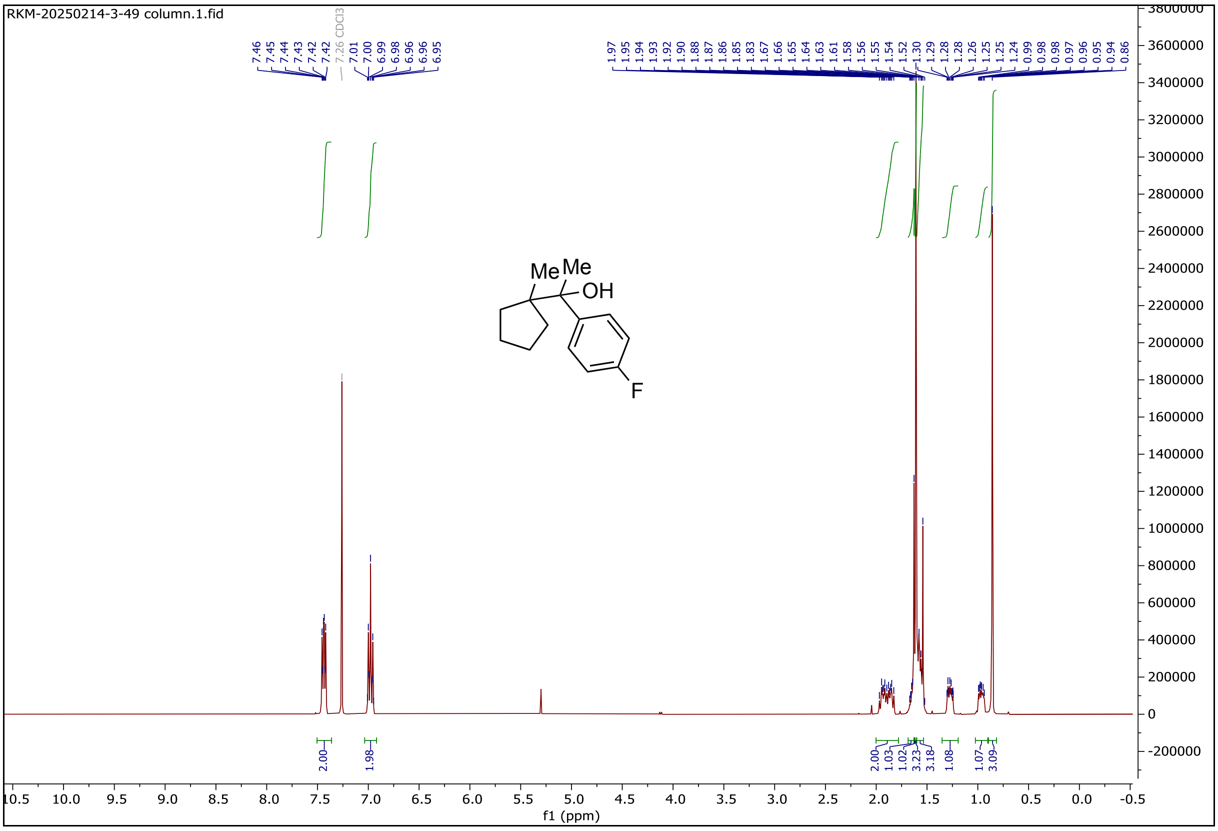
RKM-20250214-3-49 column.1.fid
7.46
7.45
7.44
7.43
7.42
7.42
7.26 CDCl3
7.01
7.00
6.99
6.98
6.96
6.96
6.95
Me
Me
OH
F
2.00
1.98
2.00
1.03
1.02
3.23
3.18
1.08
1.07
3.09
f1 (ppm)

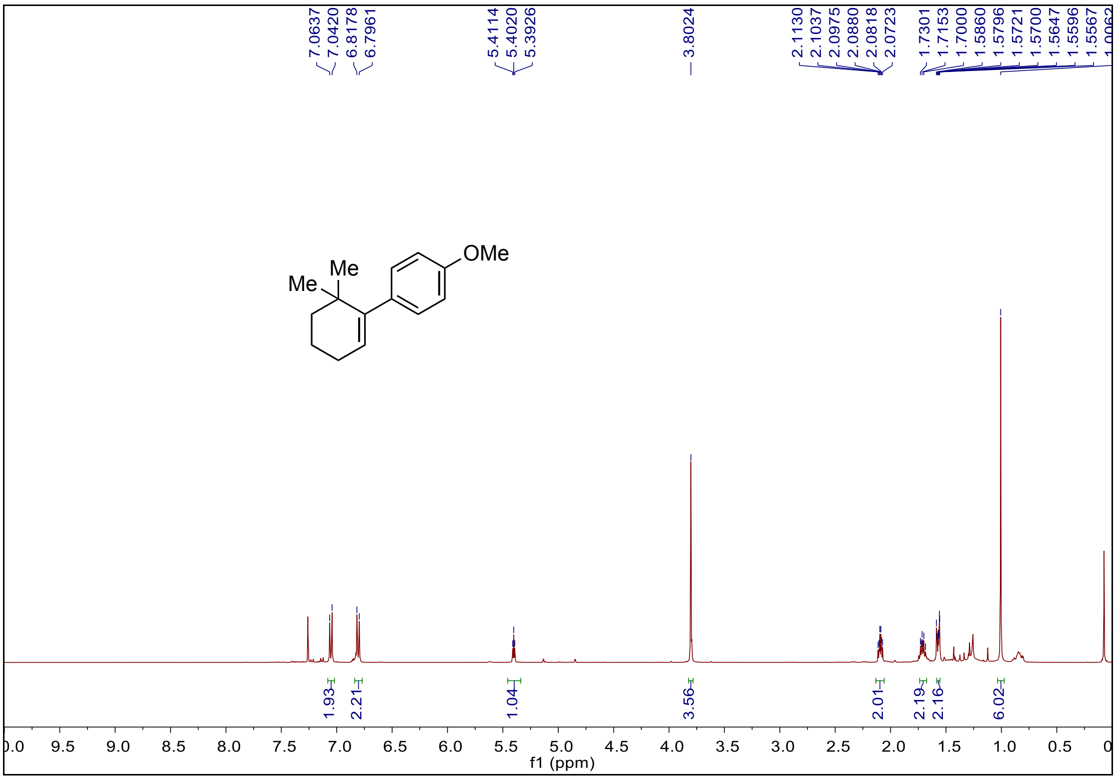

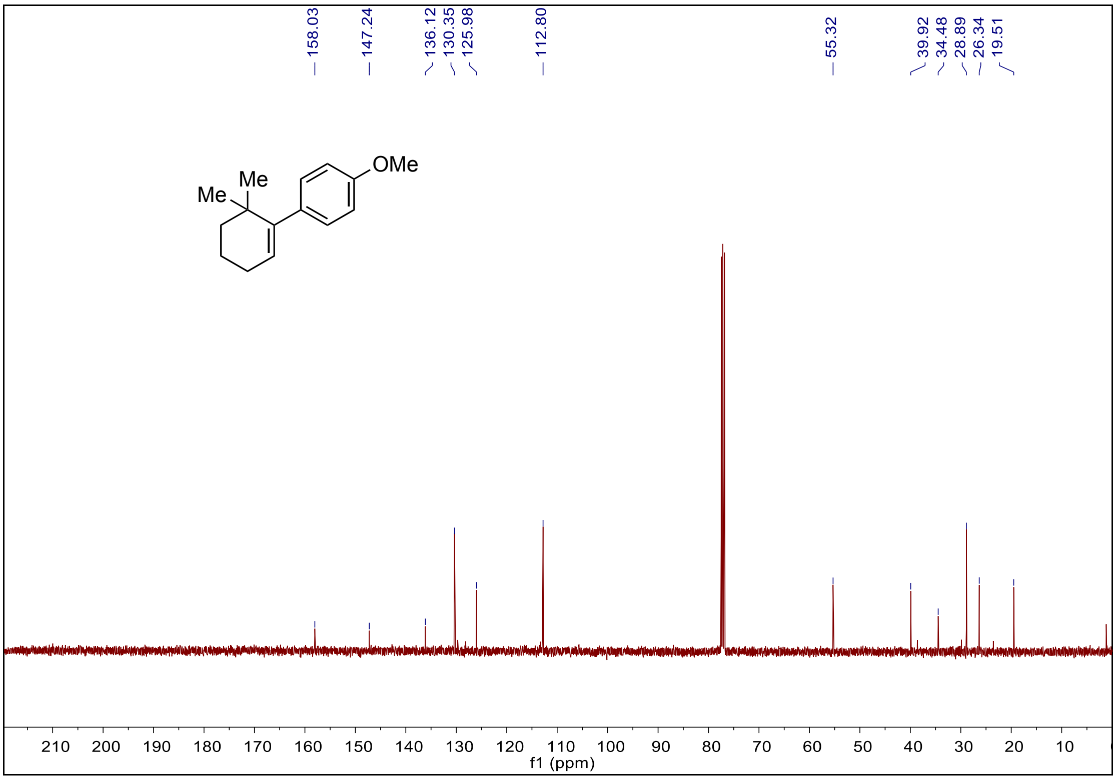

NMR spectra of final products

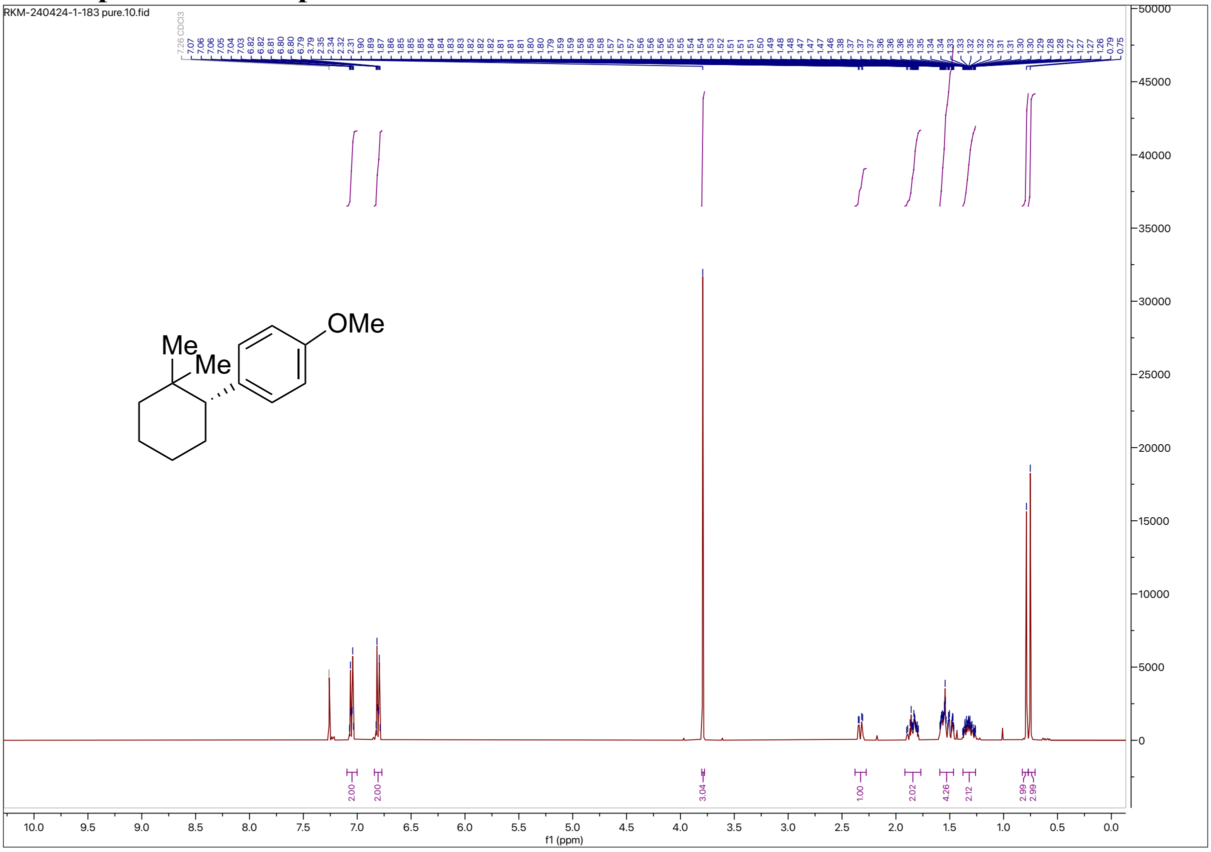

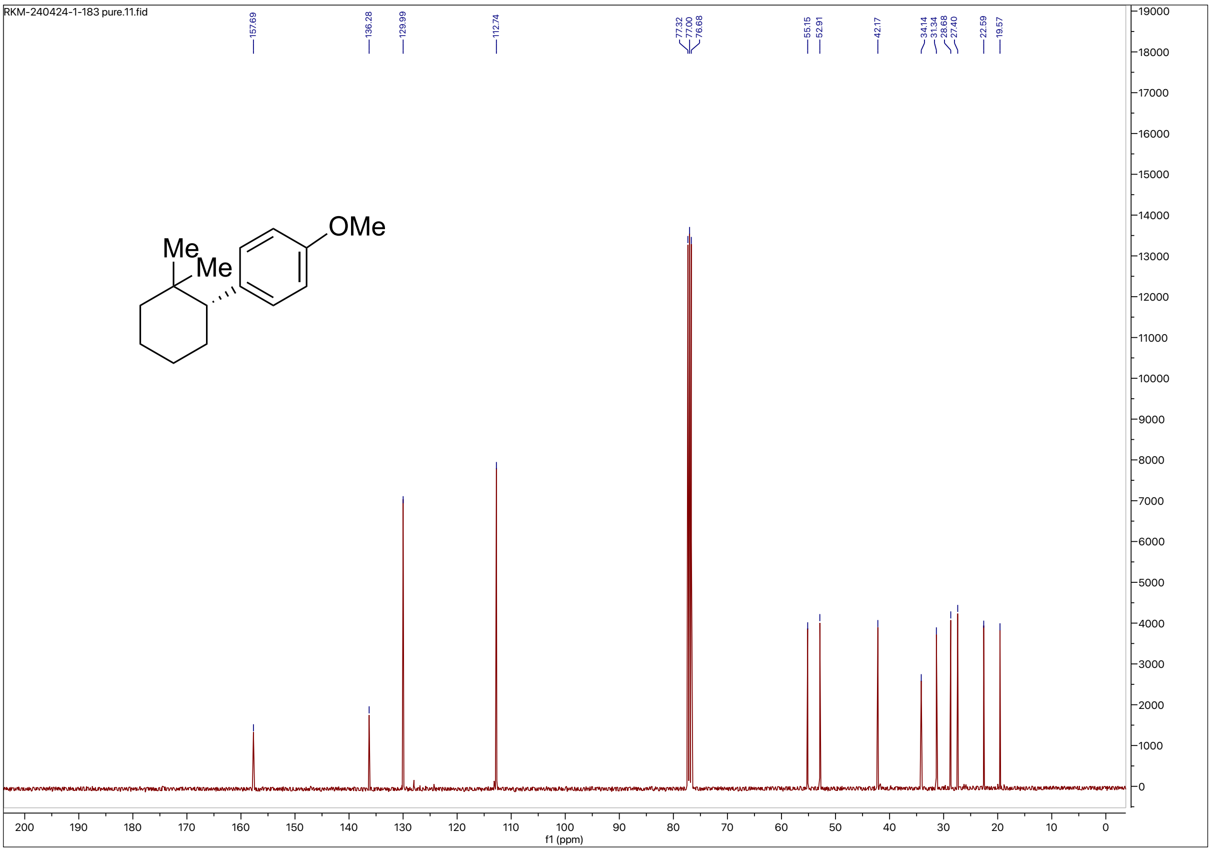

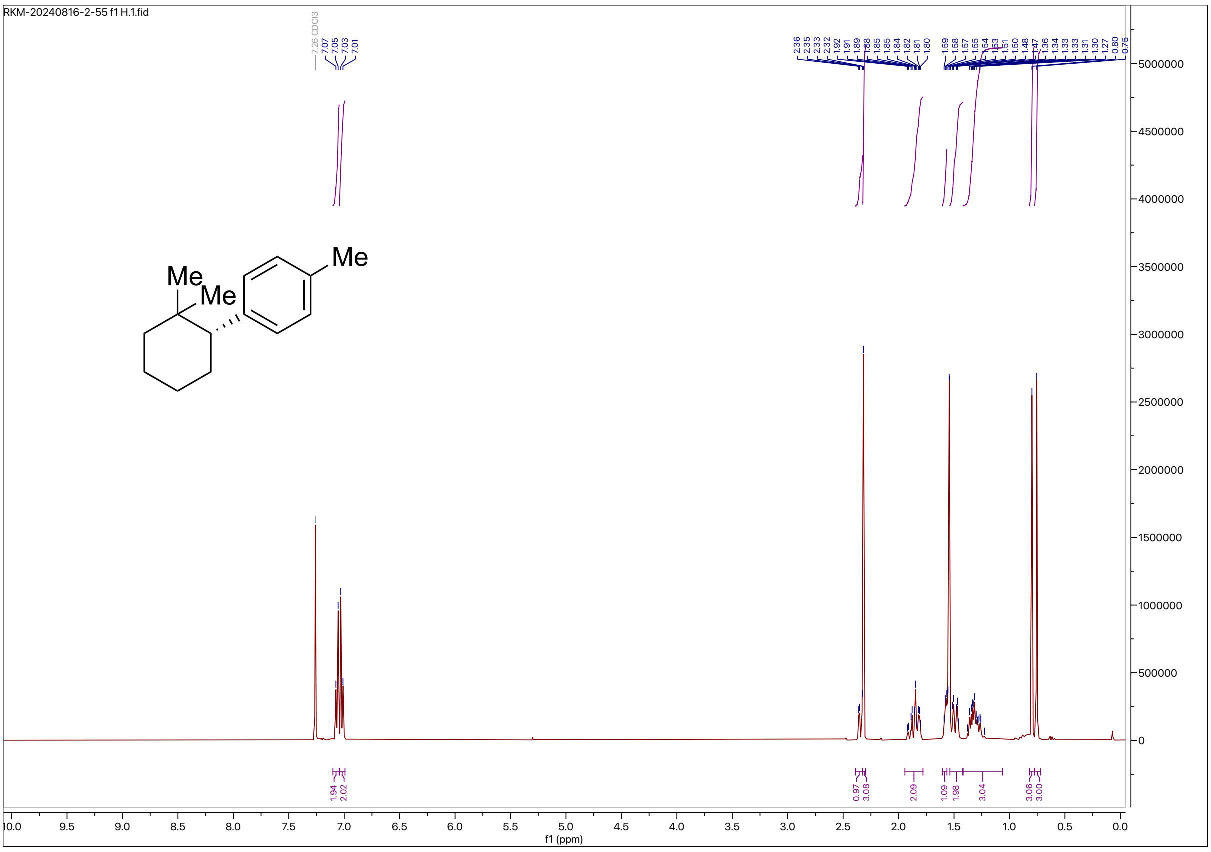
RKM-20240816-2-55 f1 H.1.fid
7.26 CDCl3
7.07
7.05
7.03
7.01
2.36
2.35
2.33
2.32
1.92
1.91
1.89
1.88
1.85
1.85
1.84
1.82
1.81
1.80
1.59
1.58
1.57
1.56
1.55
1.54
1.53
1.51
1.50
1.48
1.47
1.36
1.34
1.33
1.33
1.31
1.30
1.27
0.80
0.75
Me
Me
Me
1.94
2.02
0.97
3.08
2.09
1.09
1.98
3.04
3.06
3.00
f1 (ppm)

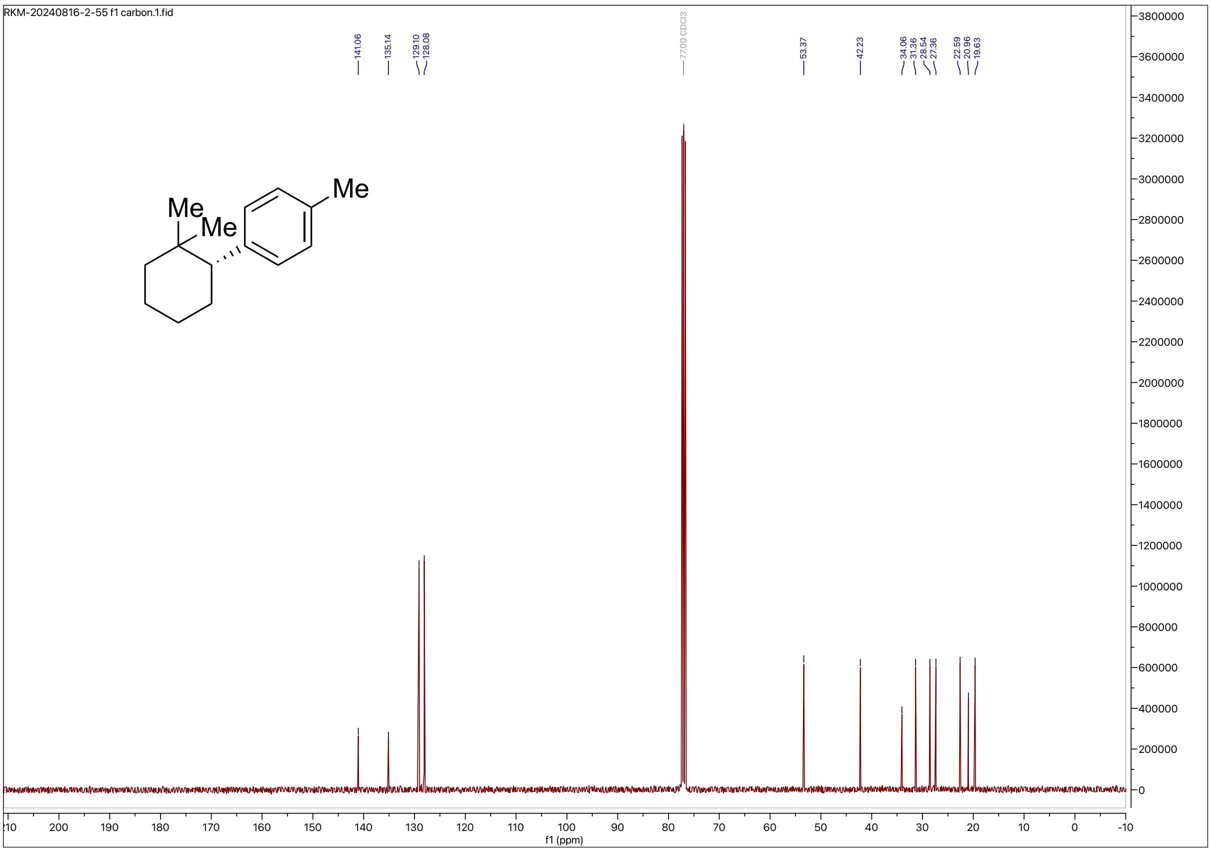
RKM-20240816-2-55 f1 carbon.1.fid
141.06
135.14
129.10
128.08
77.00 CDCl3
53.37
42.23
34.06
31.36
28.54
27.36
22.59
20.96
19.63
Me
Me
Me
f1 (ppm)

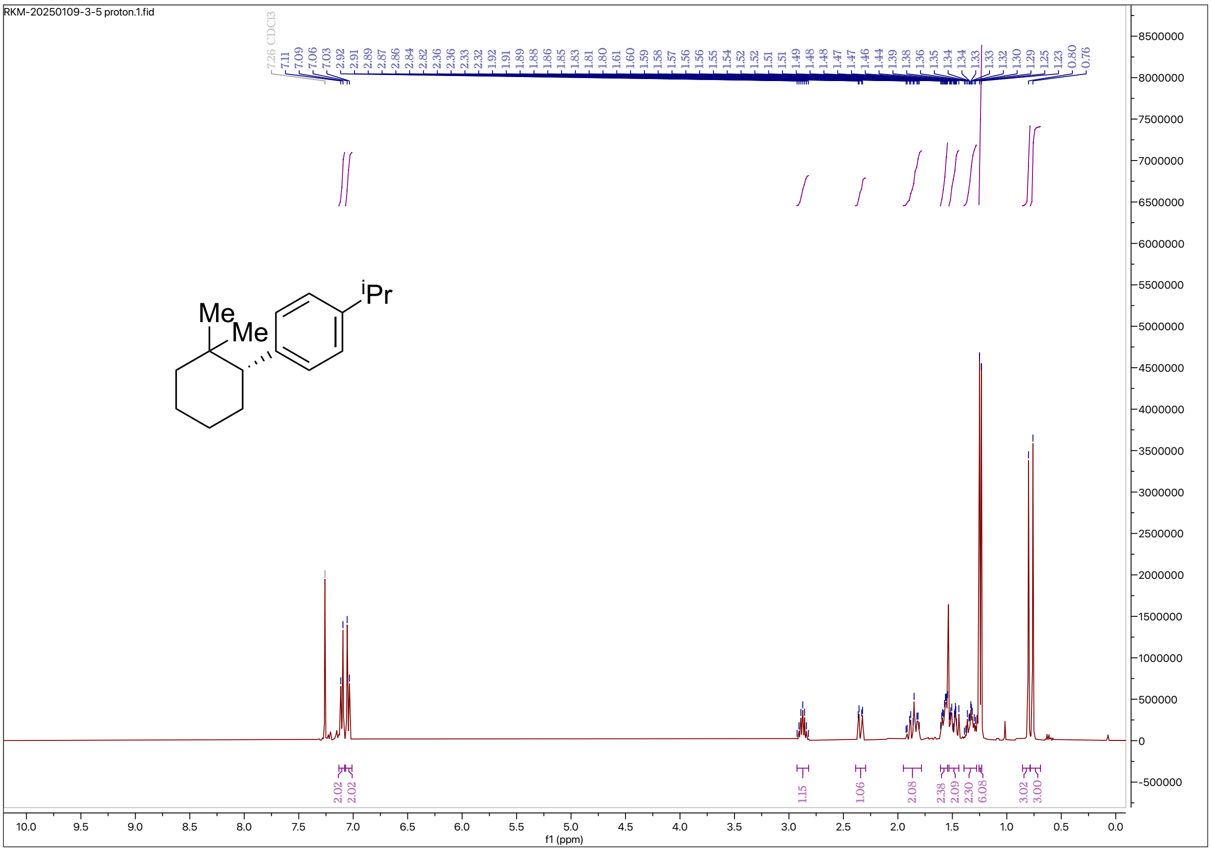
RKM-20250109-3-5 proton.1.fid
Me
Me
iPr
f1 (ppm)

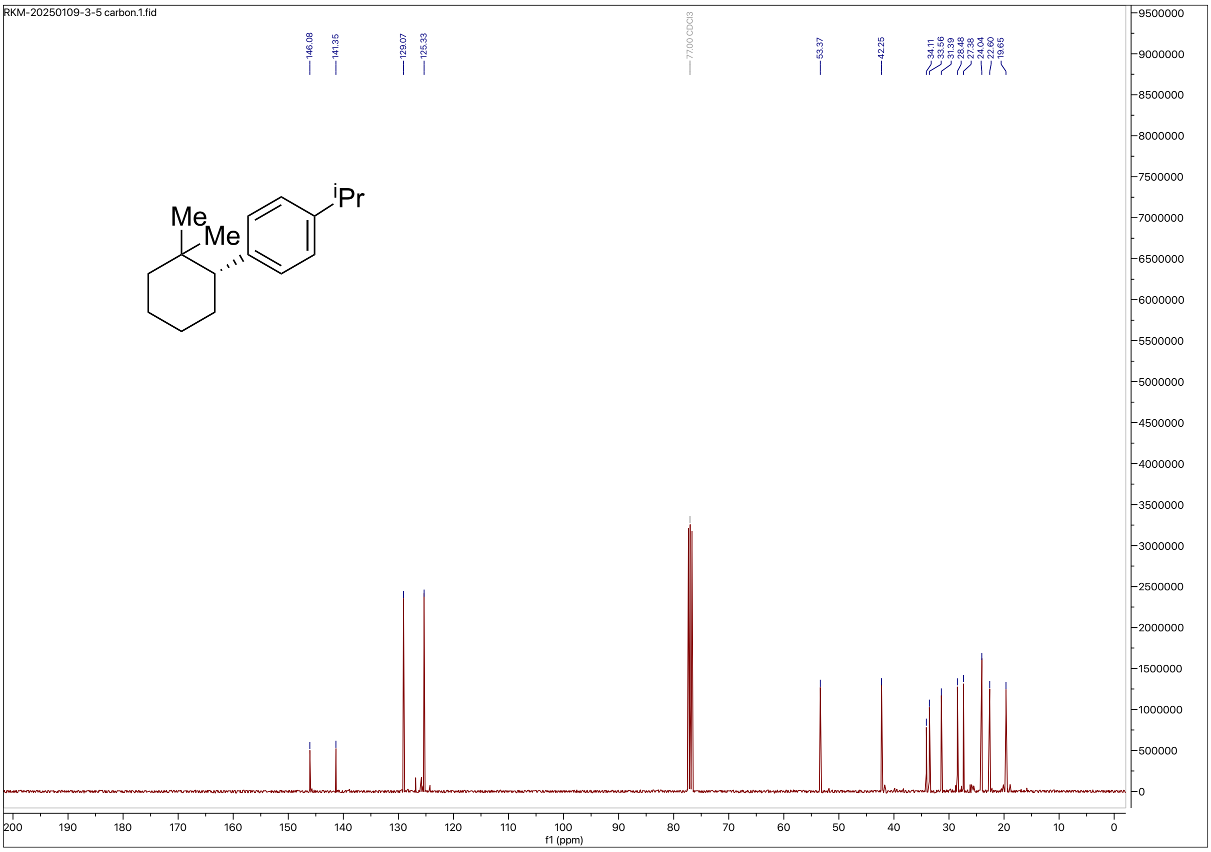
RKM-20250109-3-5 carbon.1.fid
146.08
141.35
129.07
125.33
77.00 CDCl3
53.37
42.25
34.11
33.56
31.39
28.48
27.38
24.04
22.60
19.65
Me
Me
iPr
f1 (ppm)

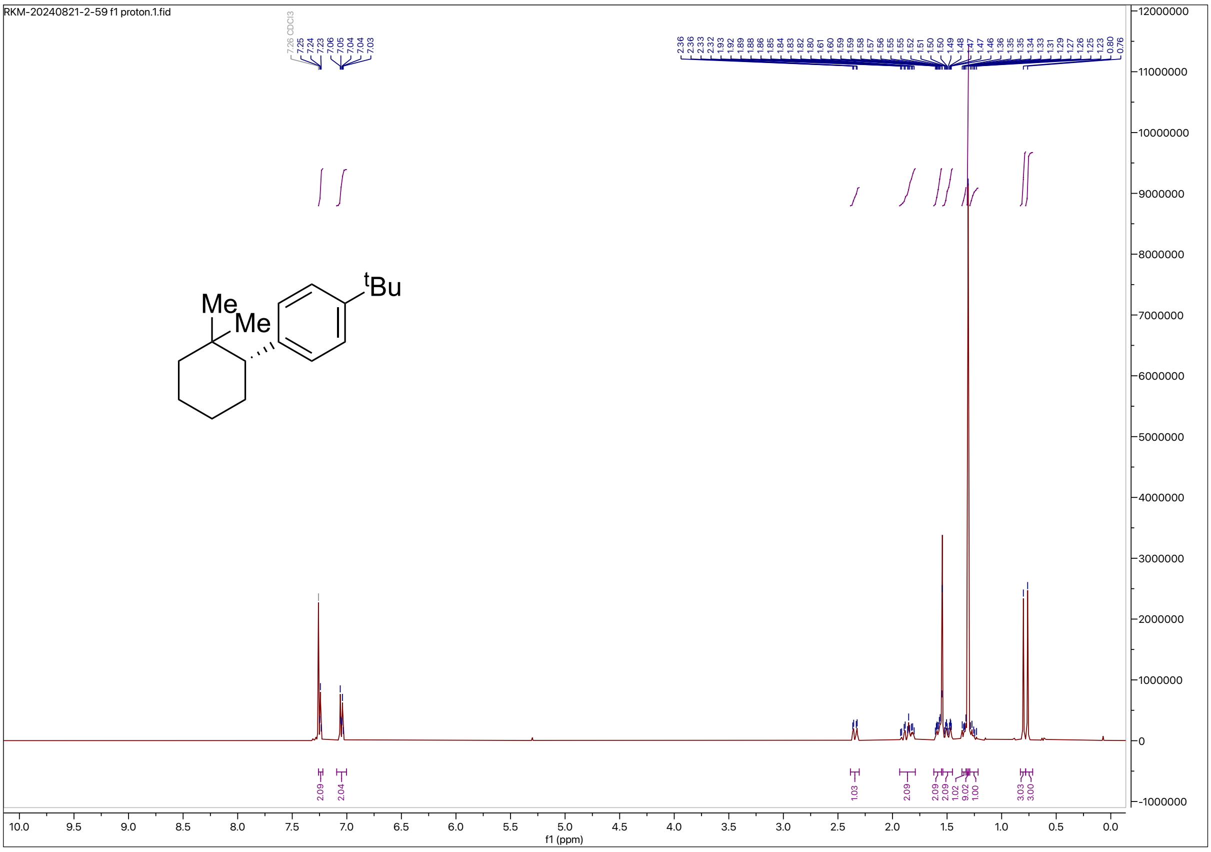
RKM-20240821-2-59 f1 proton.1.fid
tBu
Me
Me
f1 (ppm)

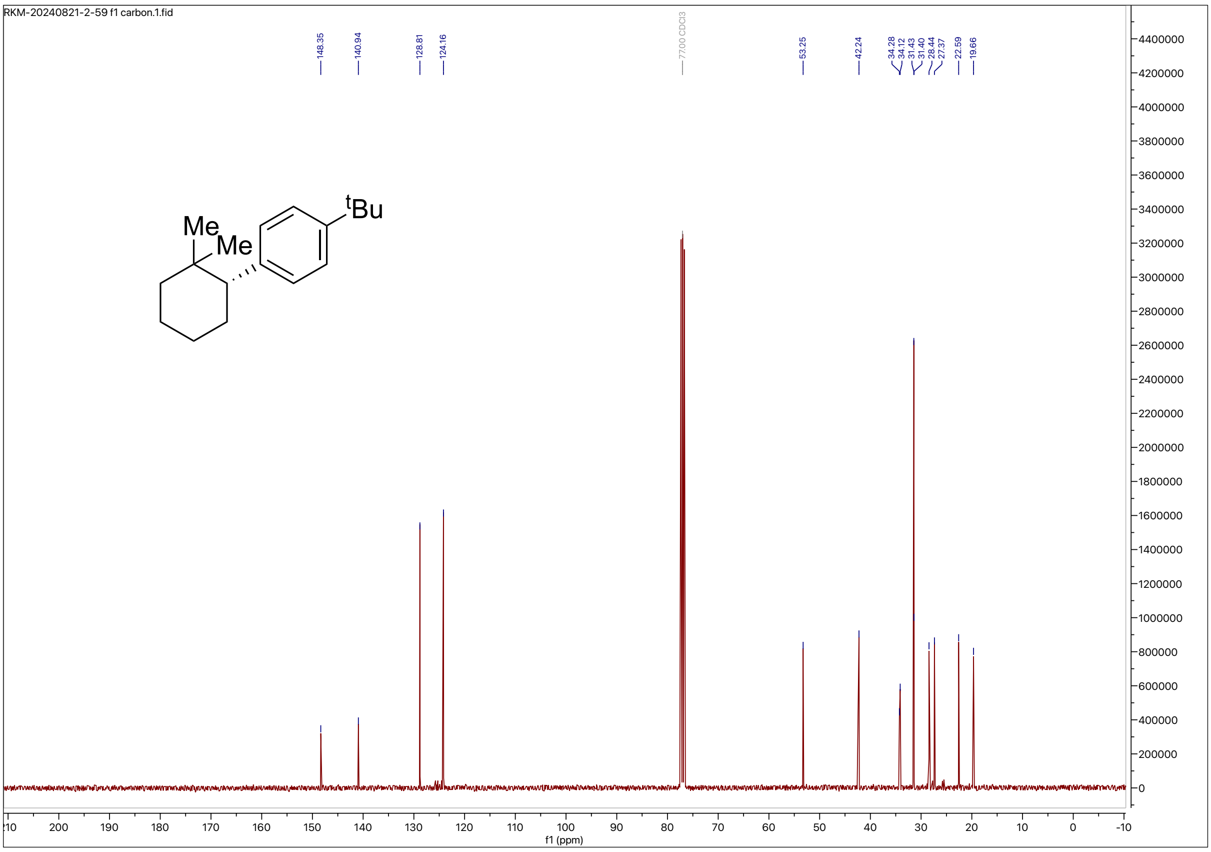
RKM-20240821-2-59 f1 carbon.1.fid
148.35
140.94
128.81
124.16
77.00 CDCl3
53.25
42.24
34.28
34.12
31.43
31.40
28.44
27.37
22.59
19.66
tBu
Me
Me
f1 (ppm)

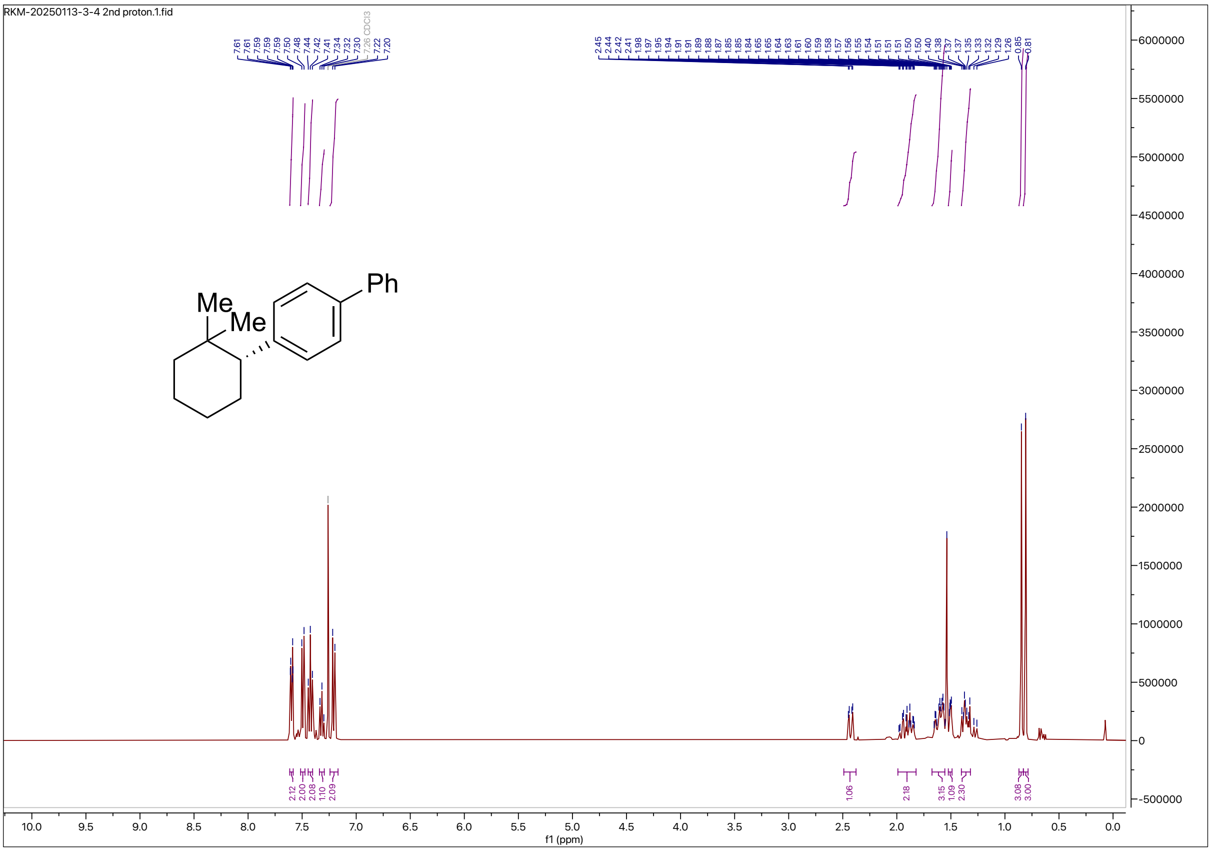
RKM-20250113-3-4 2nd proton.1.fid
Me
Me
Ph
f1 (ppm)

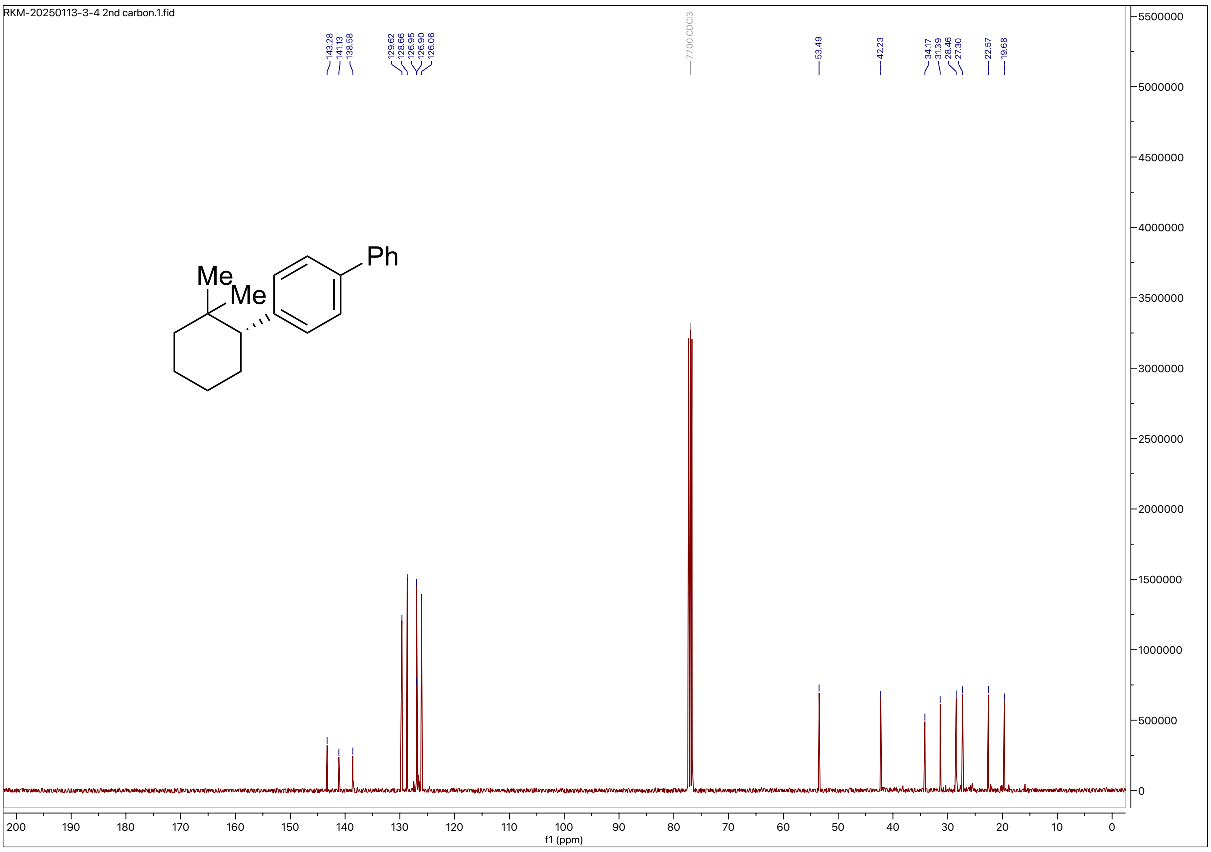
RKM-20250113-3-4 2nd carbon.1.fid
143.28
141.13
138.58
129.62
128.66
126.95
126.90
126.06
77.00 CDCl3
53.49
42.23
34.17
31.39
28.46
27.30
22.57
19.68
Me
Me
Ph
f1 (ppm)

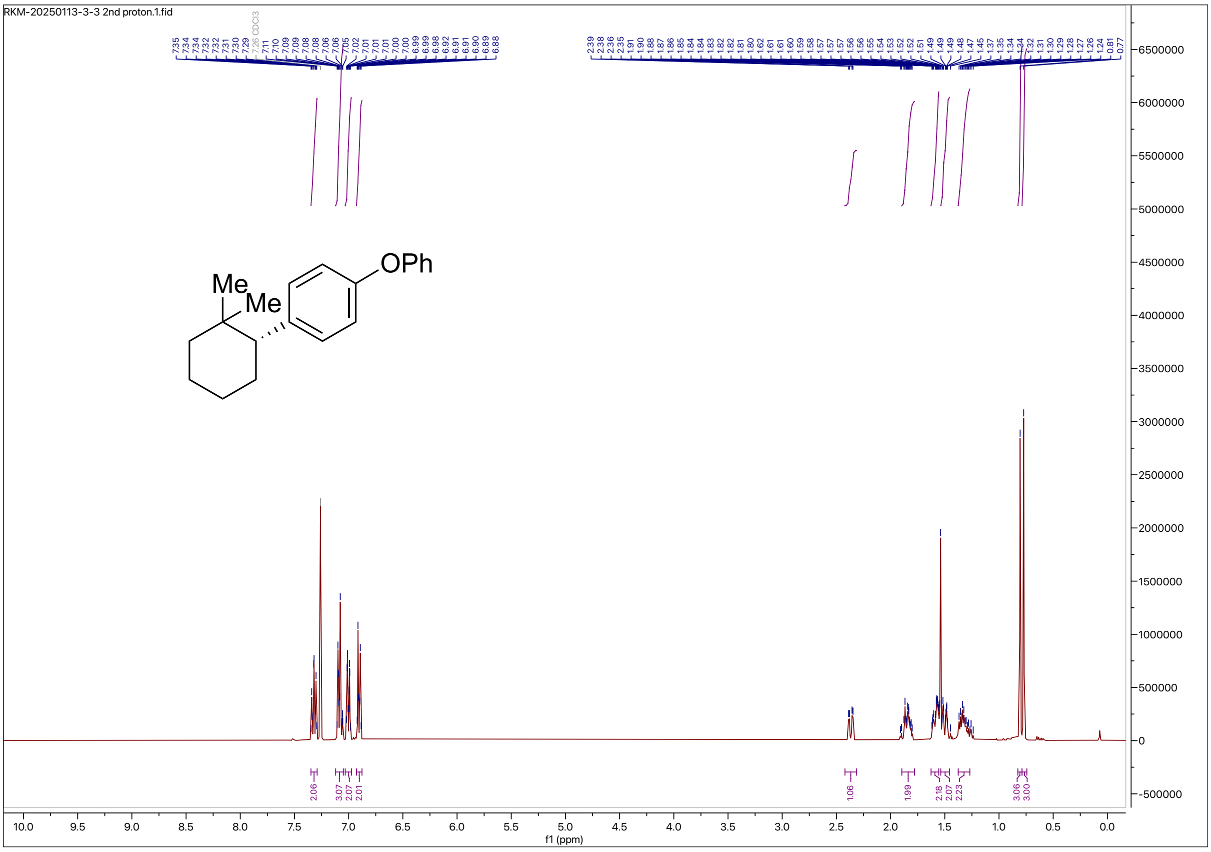
RKM-20250113-3-3 2nd proton.1.fid
OPh
Me
Me
f1 (ppm)

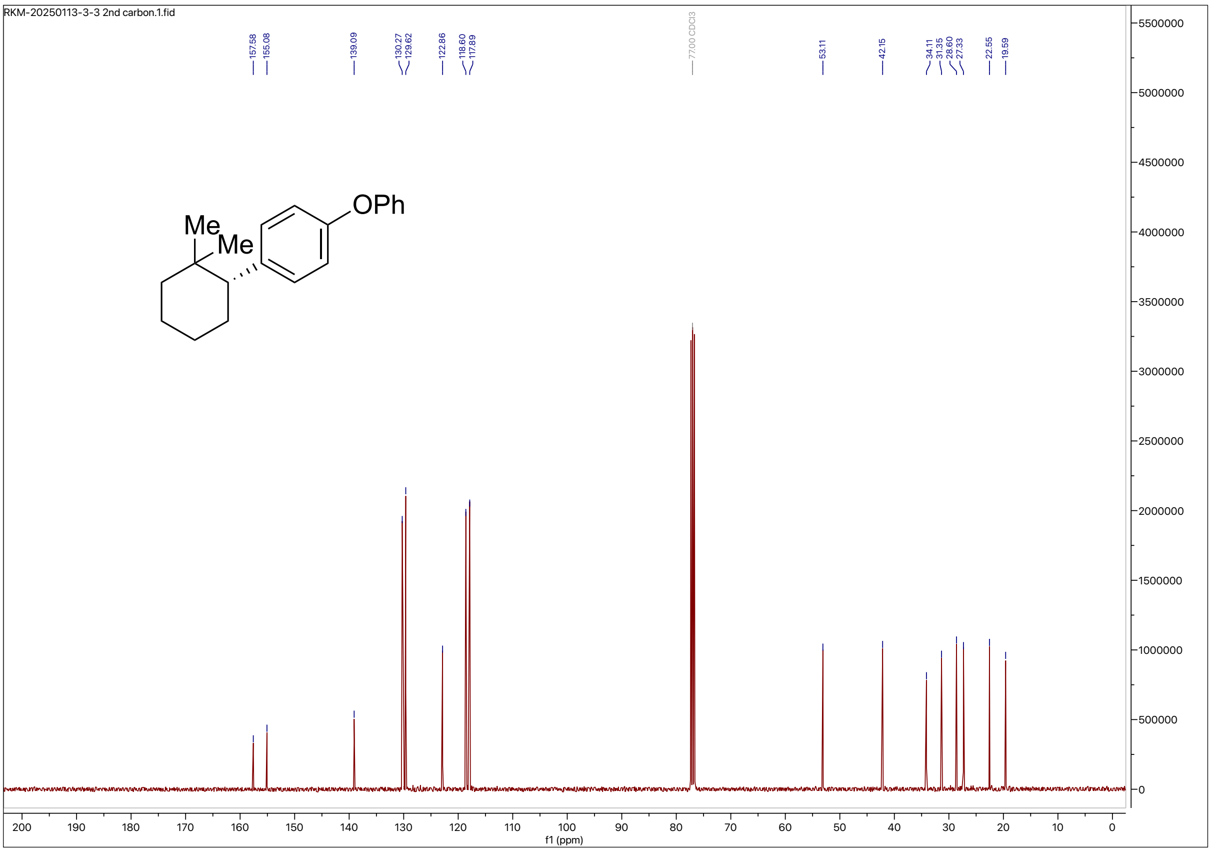
RKM-20250113-3-3 2nd carbon.1.fid
157.58
155.08
139.09
130.27
129.62
122.86
118.60
117.89
77.00 CDCl3
53.11
42.15
34.11
31.35
28.60
27.33
22.55
19.59
OPh
Me
Me
f1 (ppm)

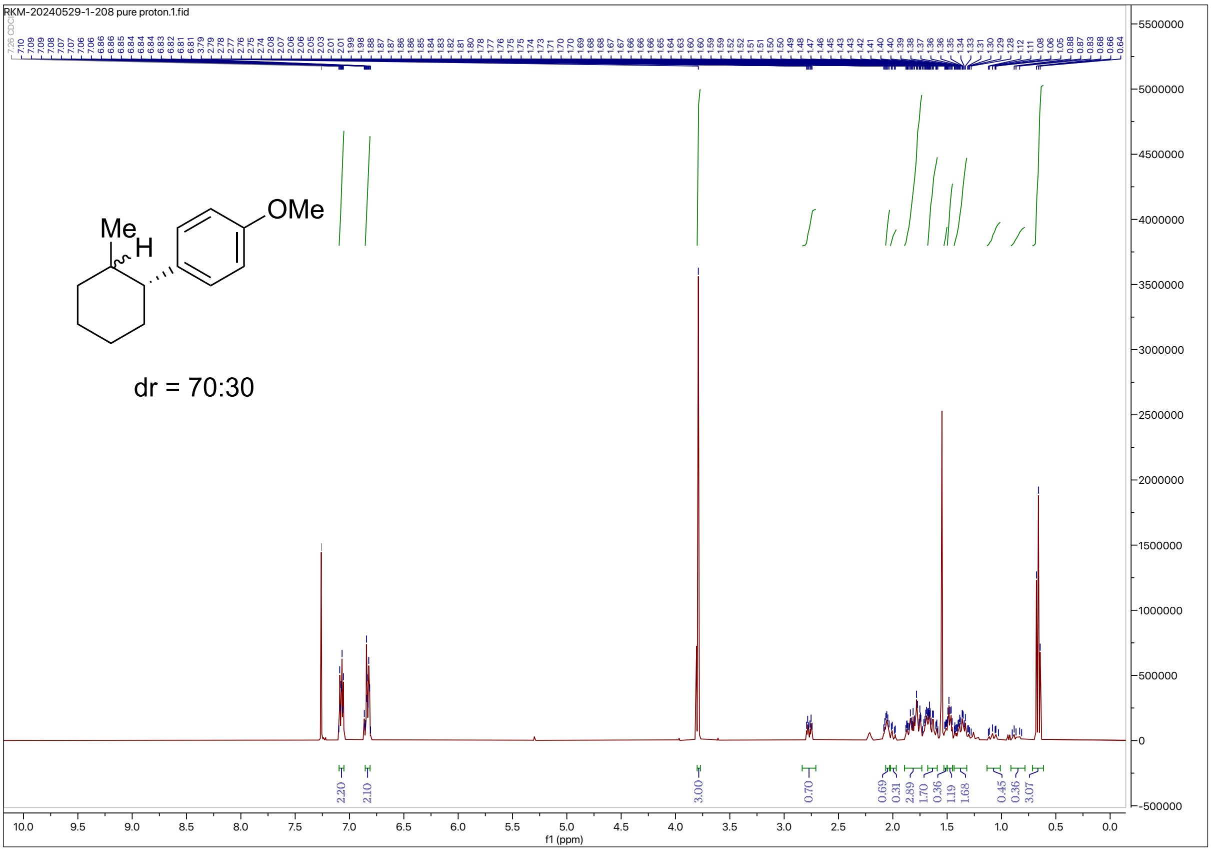
RKM-20240529-1-208 pure proton.1.fid
OMe
Me
H
dr = 70:30
f1 (ppm)

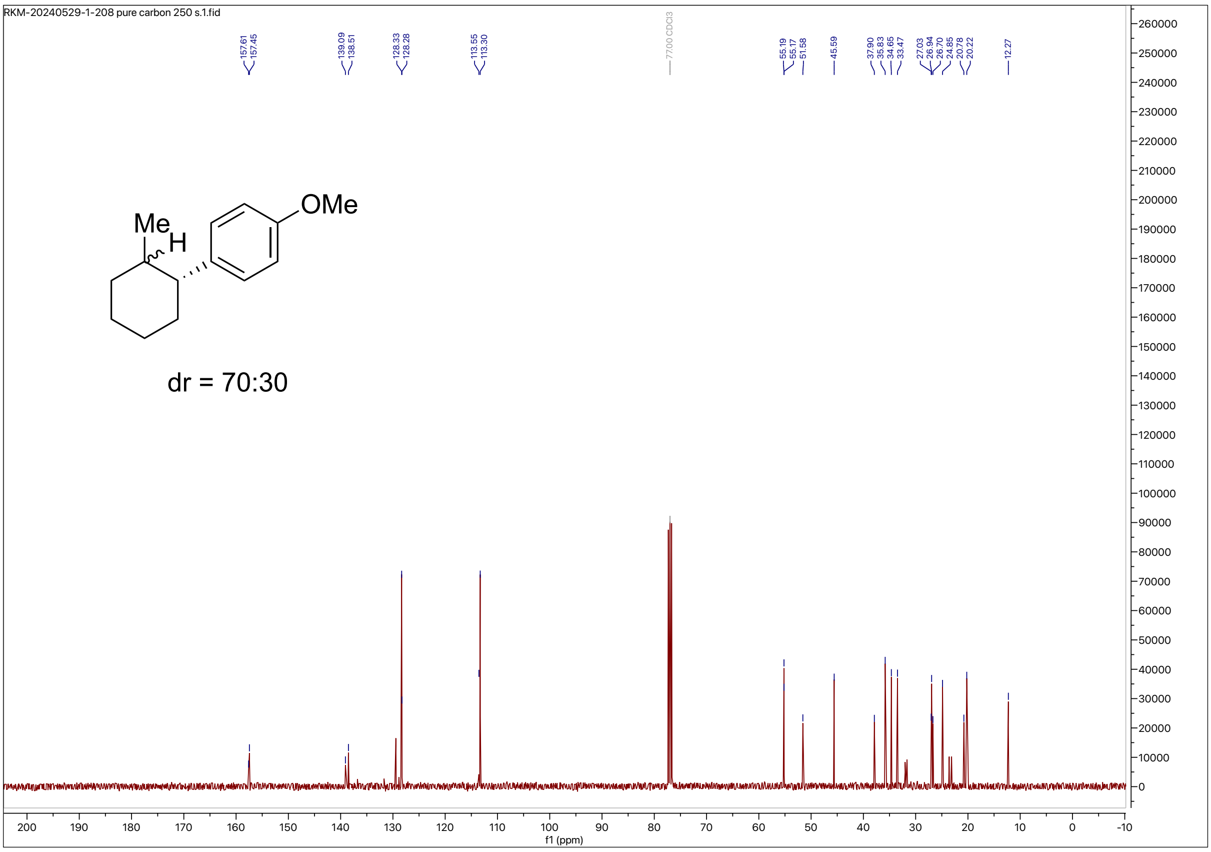
RKM-20240529-1-208 pure carbon 250 s.1.fid
OMe
Me
H
dr = 70:30
f1 (ppm)

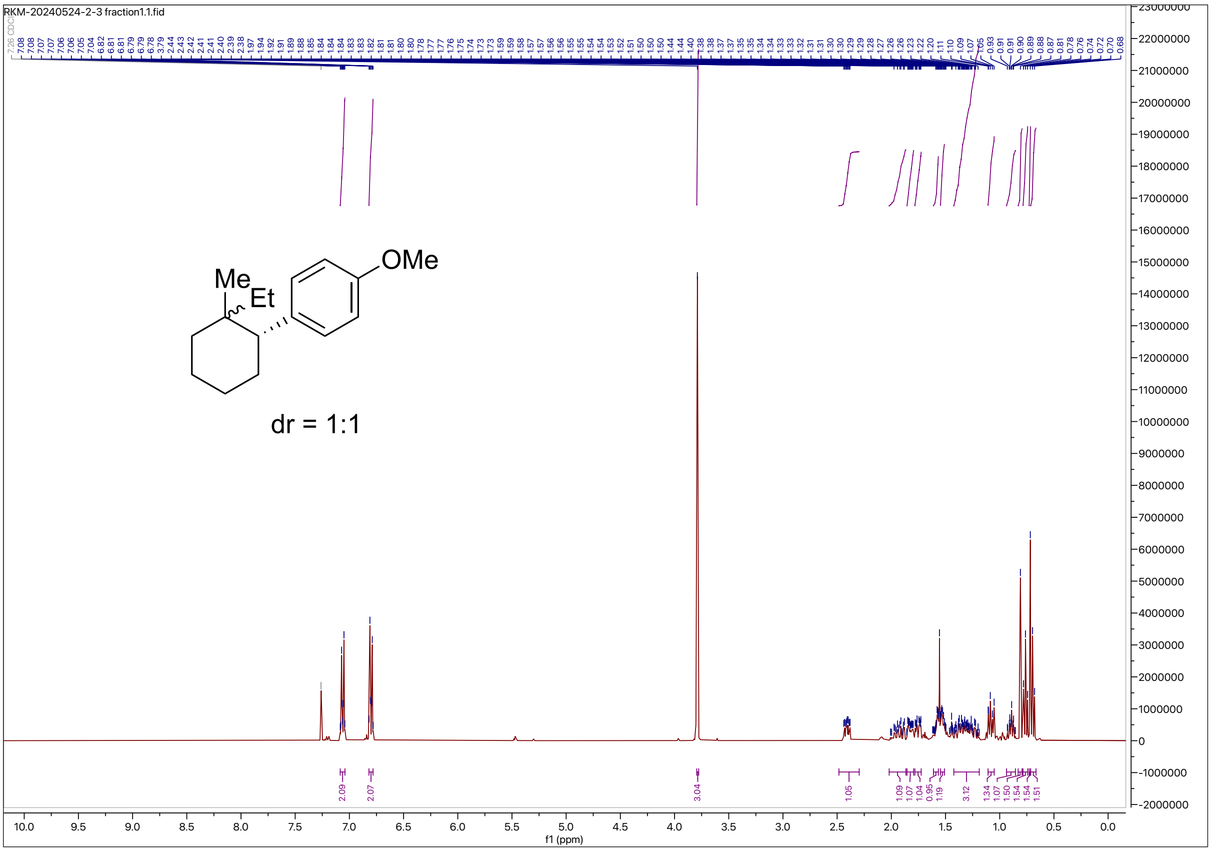
RKM-20240524-2-3 fraction1.1.fid
OMe
Me
Et
dr = 1:1
f1 (ppm)

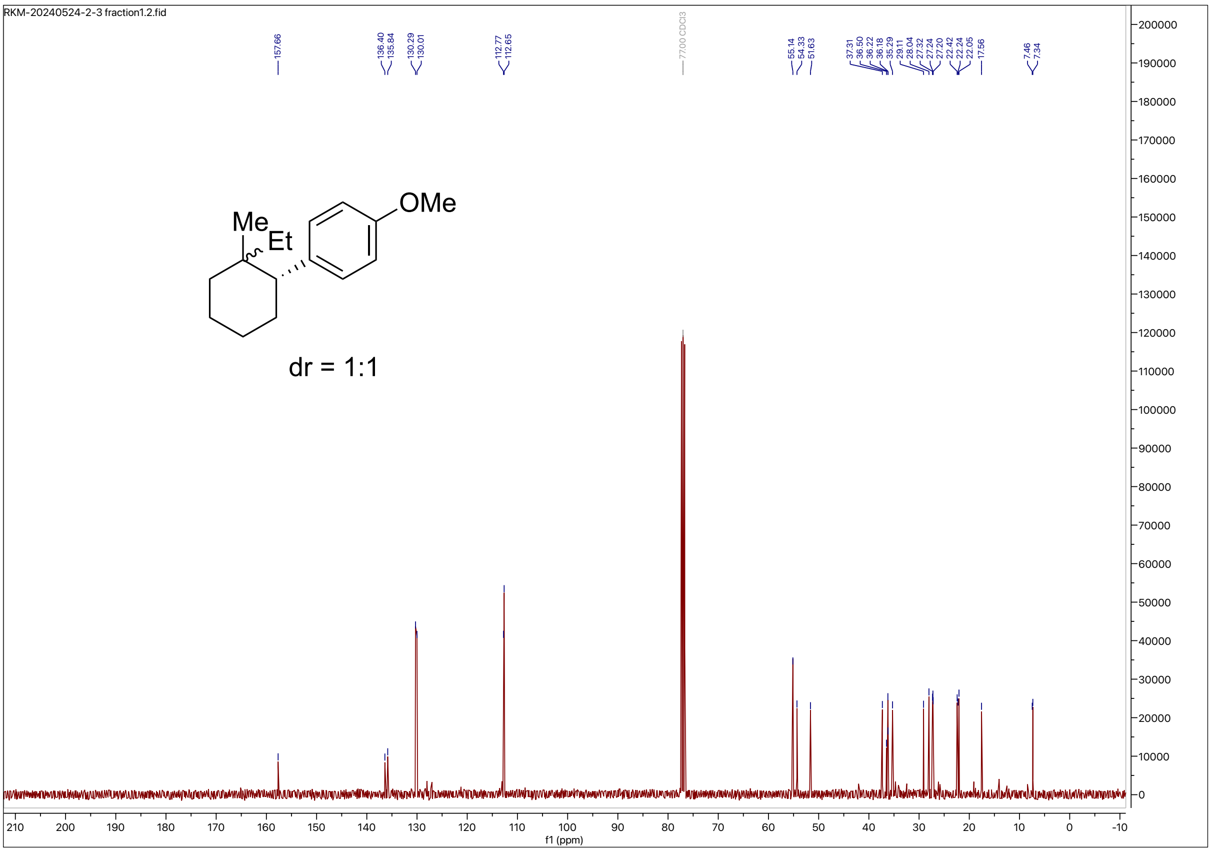
RKM-20240524-2-3 fraction1.2.fid
OMe
Me
Et
dr = 1:1
f1 (ppm)

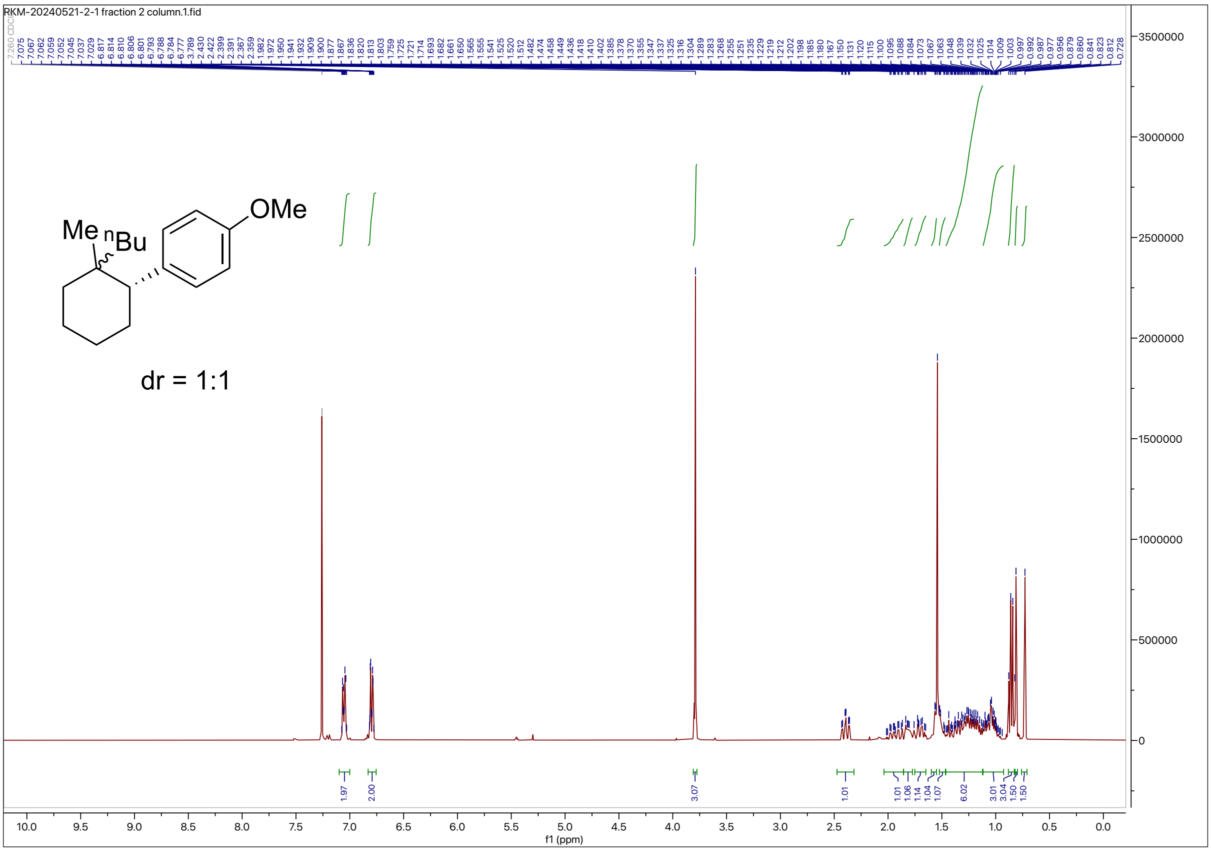

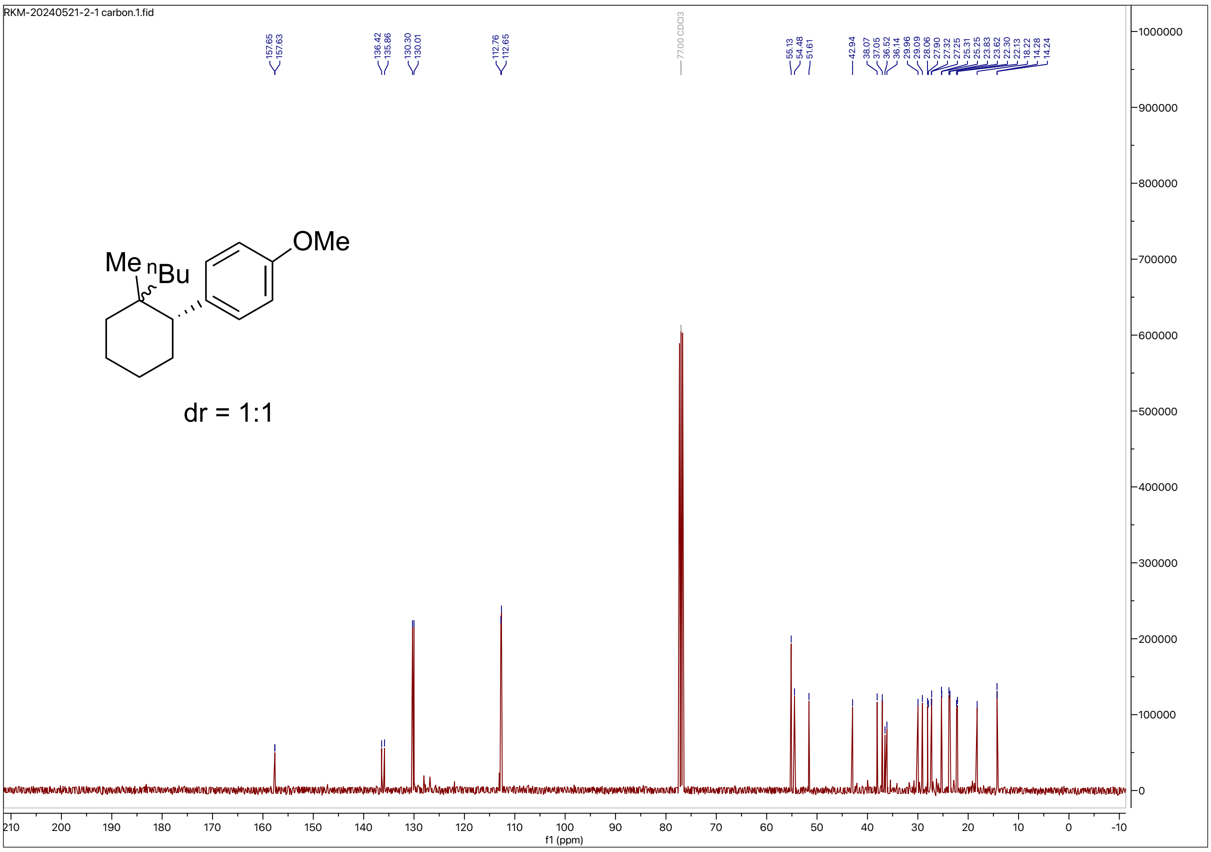

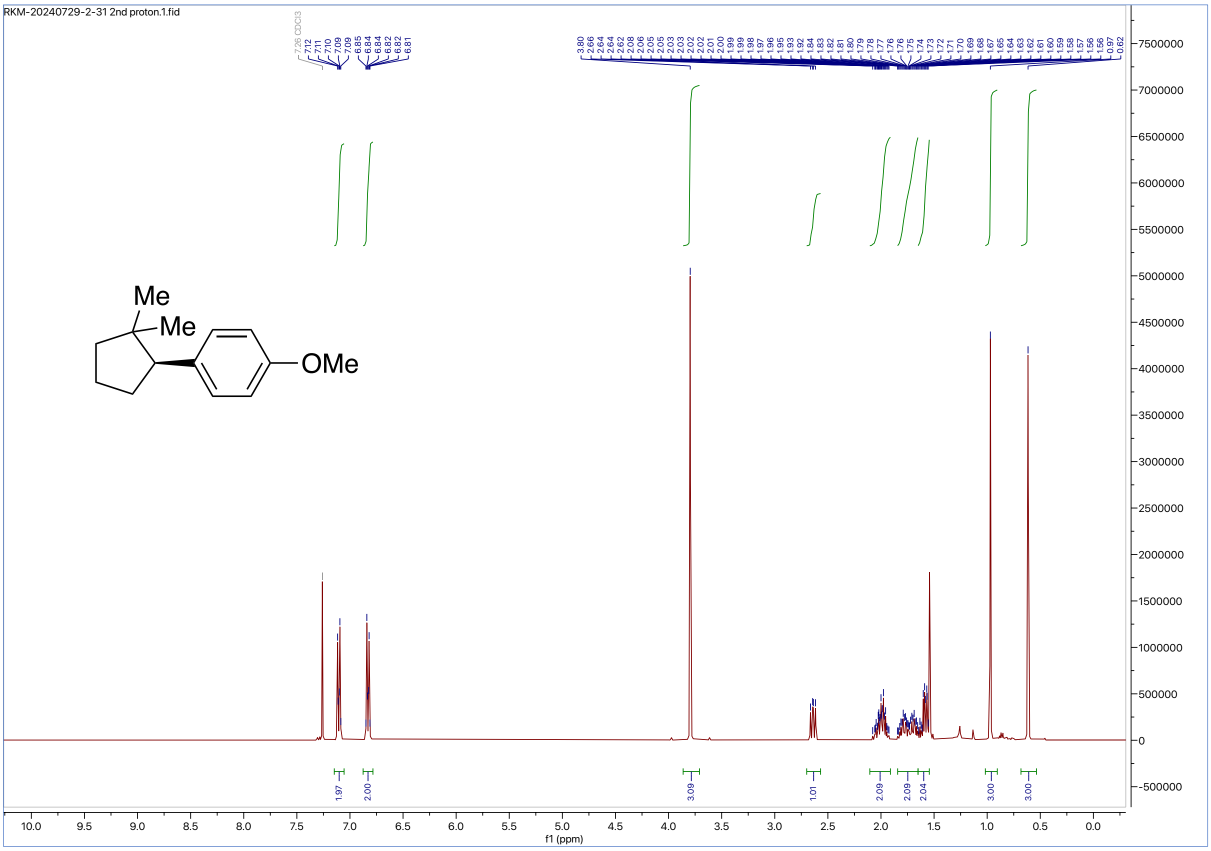

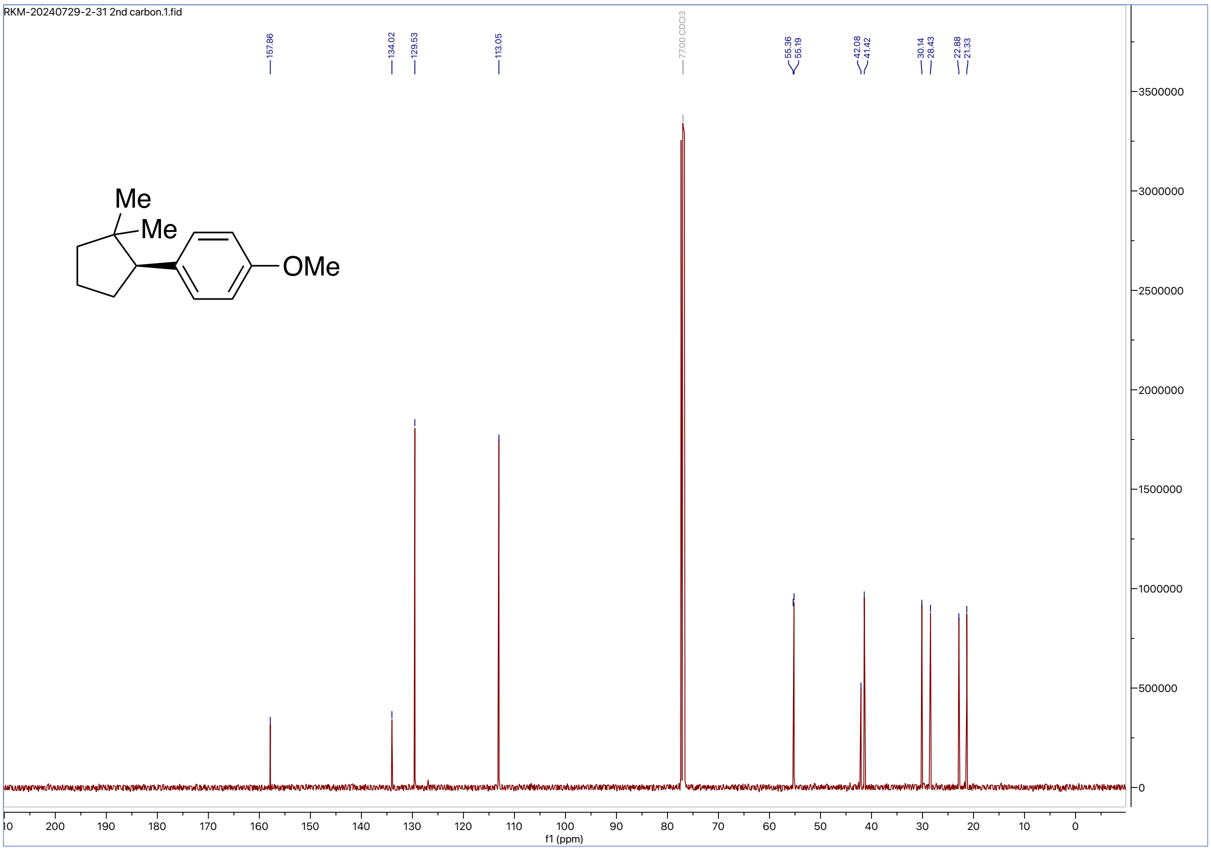

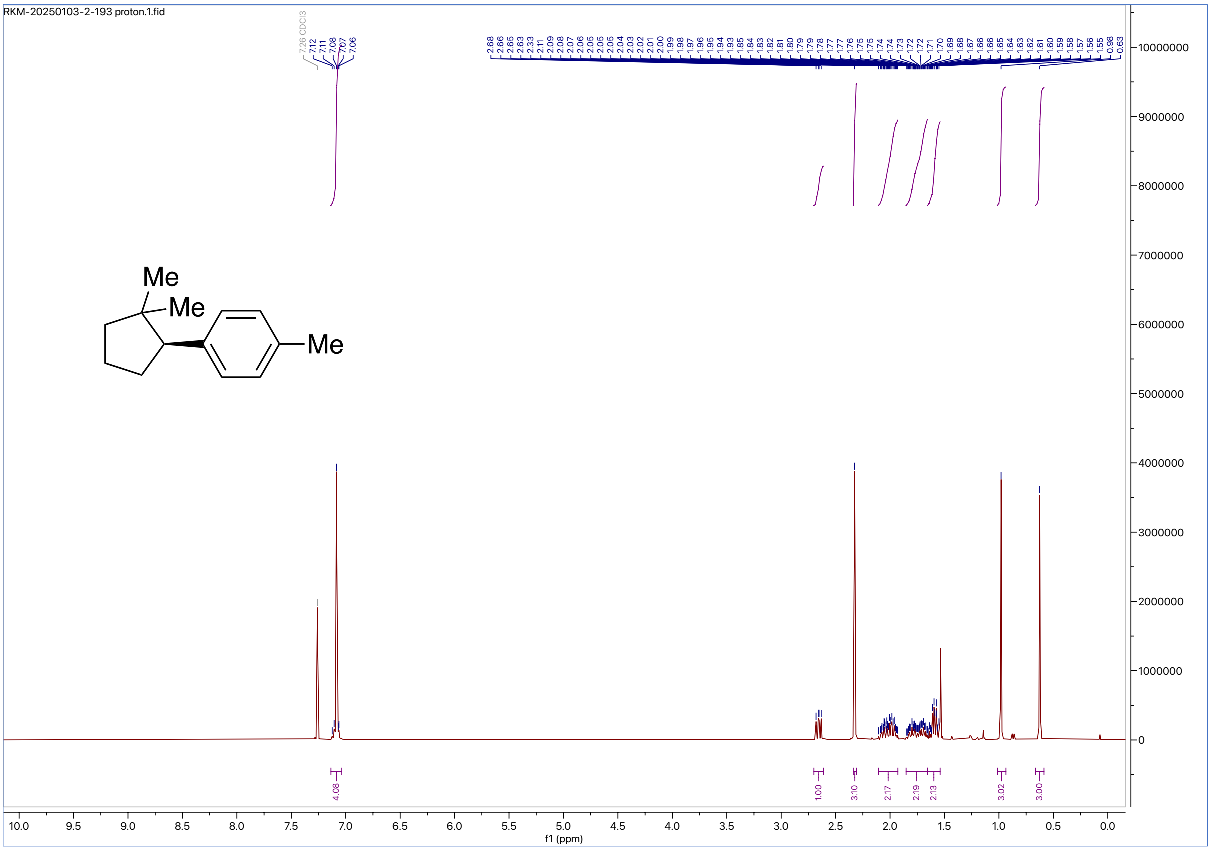
RKM-20250103-2-193 proton.1.fid
Me
Me
Me
f1 (ppm)

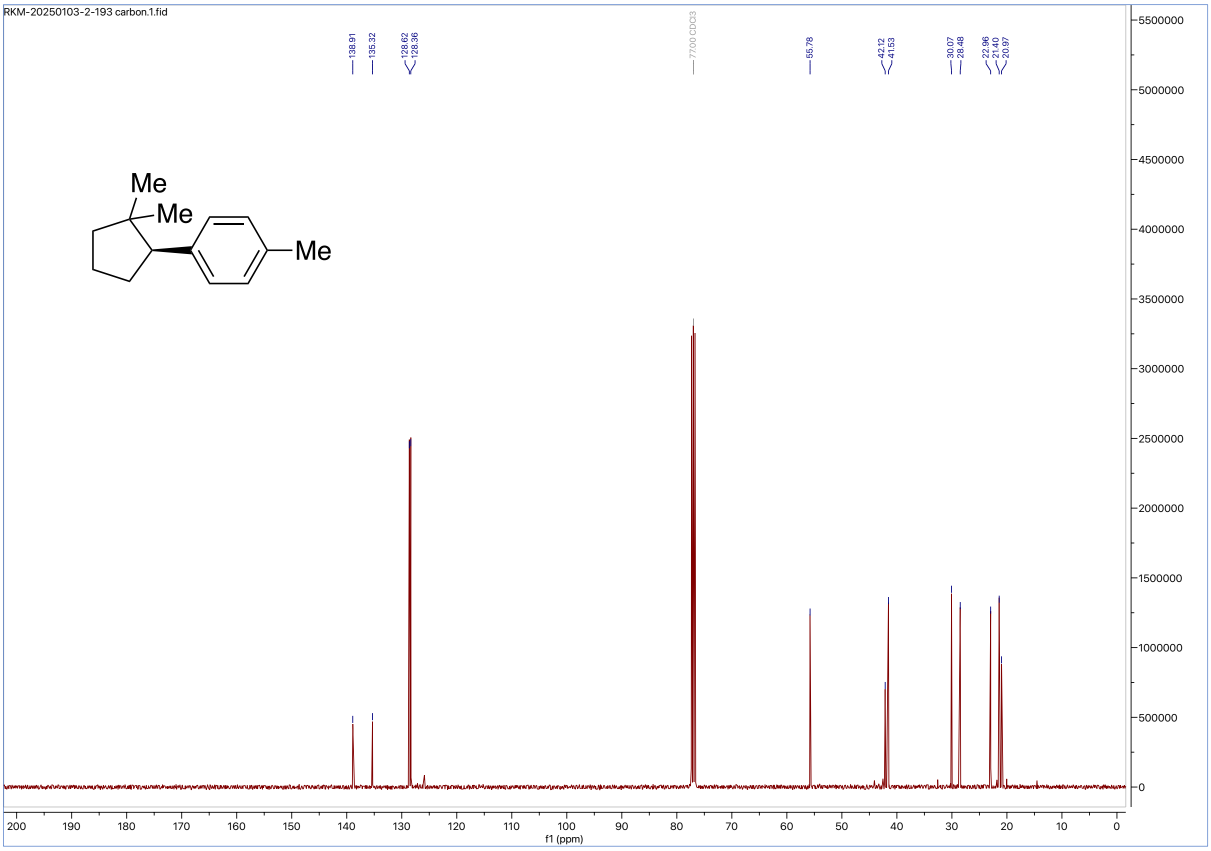
RKM-20250103-2-193 carbon.1.fid
138.91
135.32
128.62
128.36
77.00 CDCl3
55.78
42.12
41.53
30.07
28.48
22.96
21.40
20.97
Me
Me
Me
f1 (ppm)

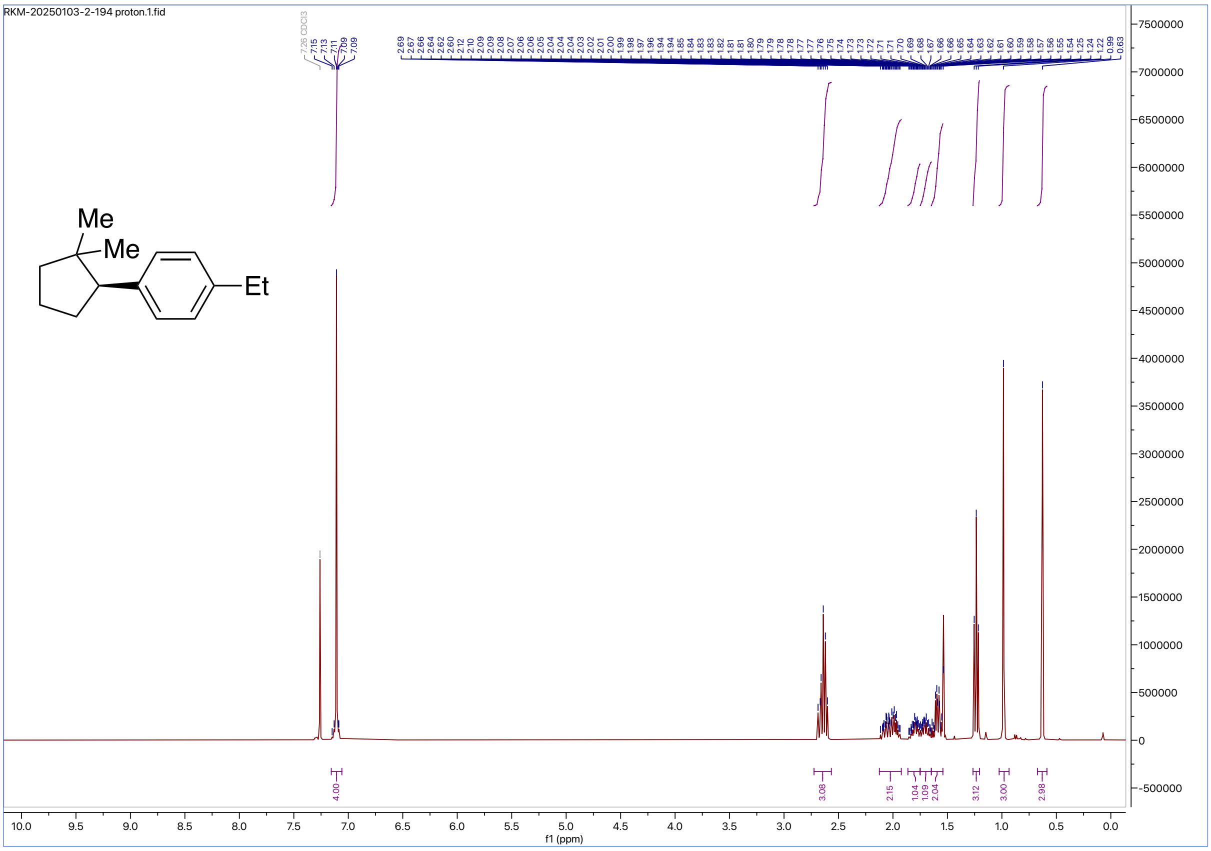
RKM-20250103-2-194 proton.1.fid
Me
Me
Et
4.00
3.08
2.15
1.04
1.09
2.04
3.12
3.00
2.98
f1 (ppm)

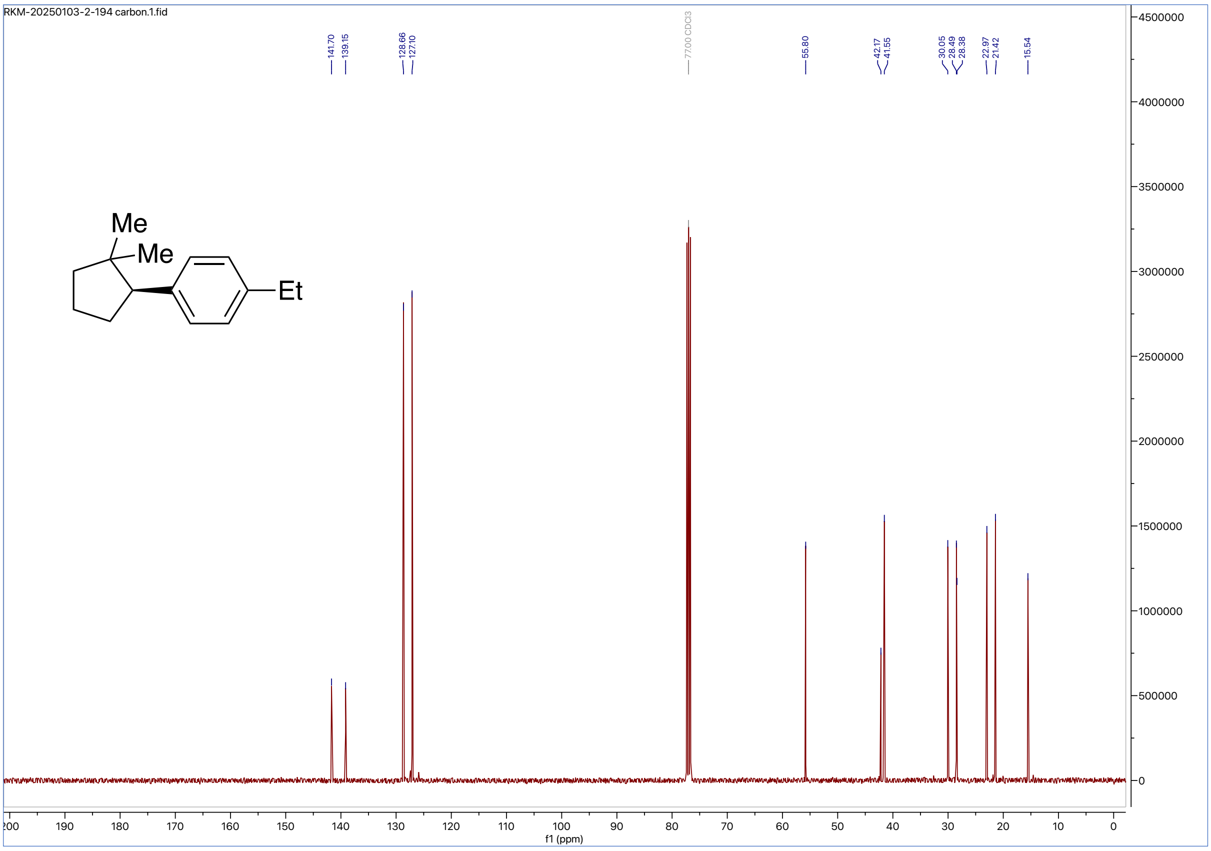
RKM-20250103-2-194 carbon.1.fid
141.70
139.15
128.66
127.10
77.00 CDCl3
55.80
42.17
41.55
30.05
28.49
28.38
22.97
21.42
15.54
Me
Me
Et
f1 (ppm)

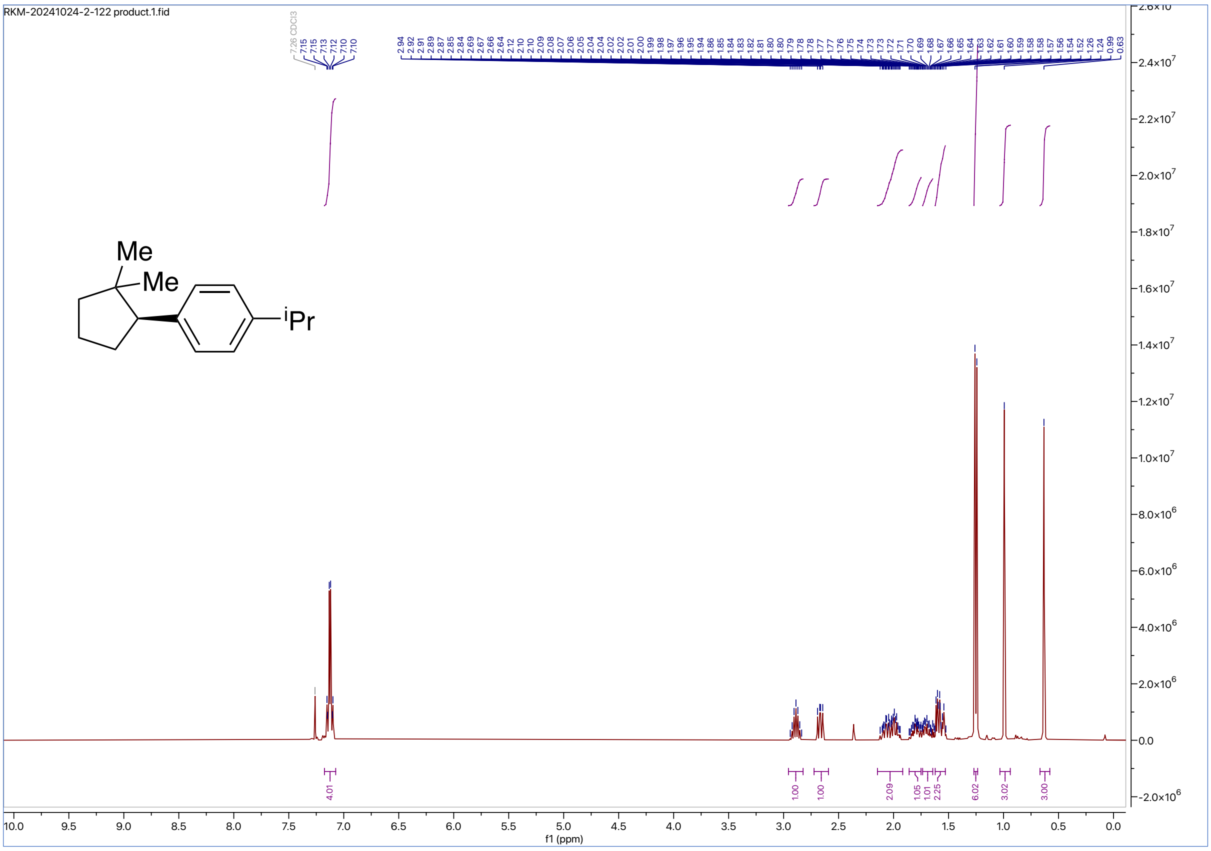

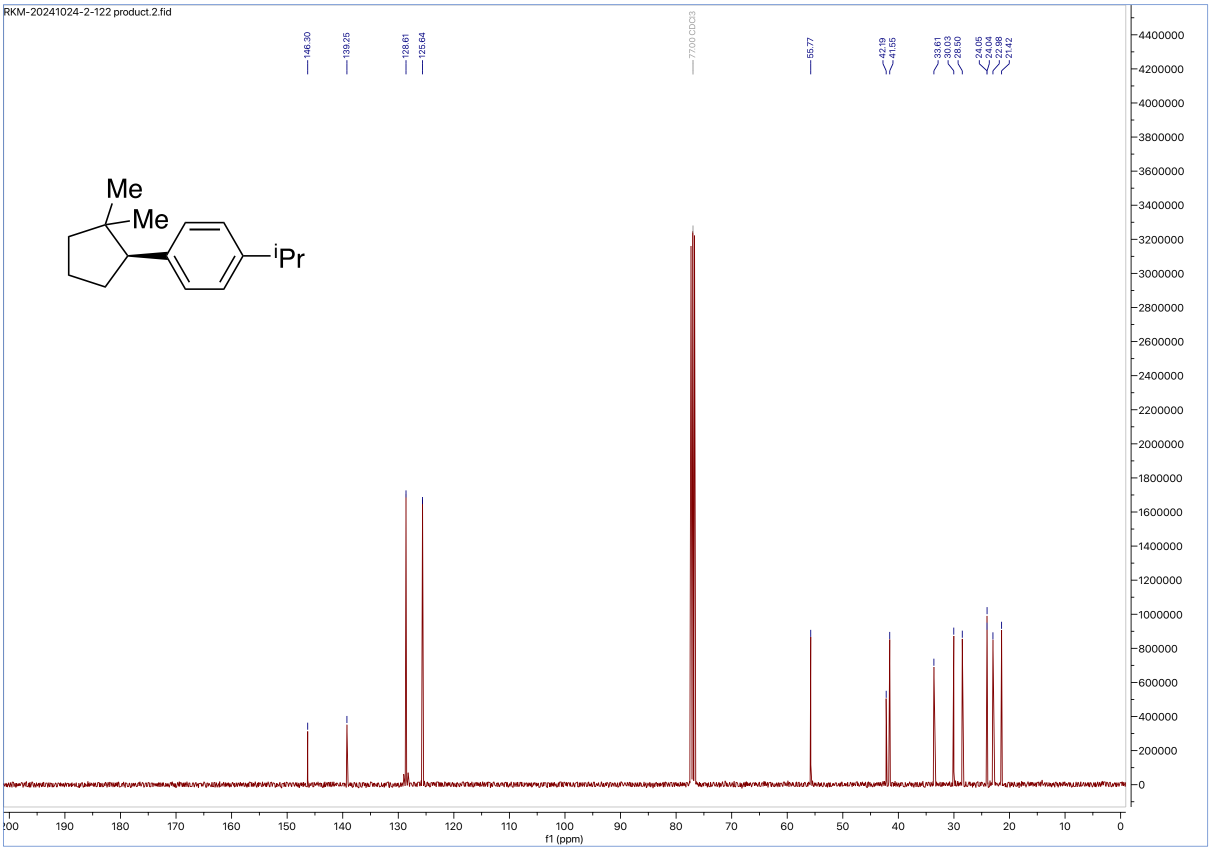

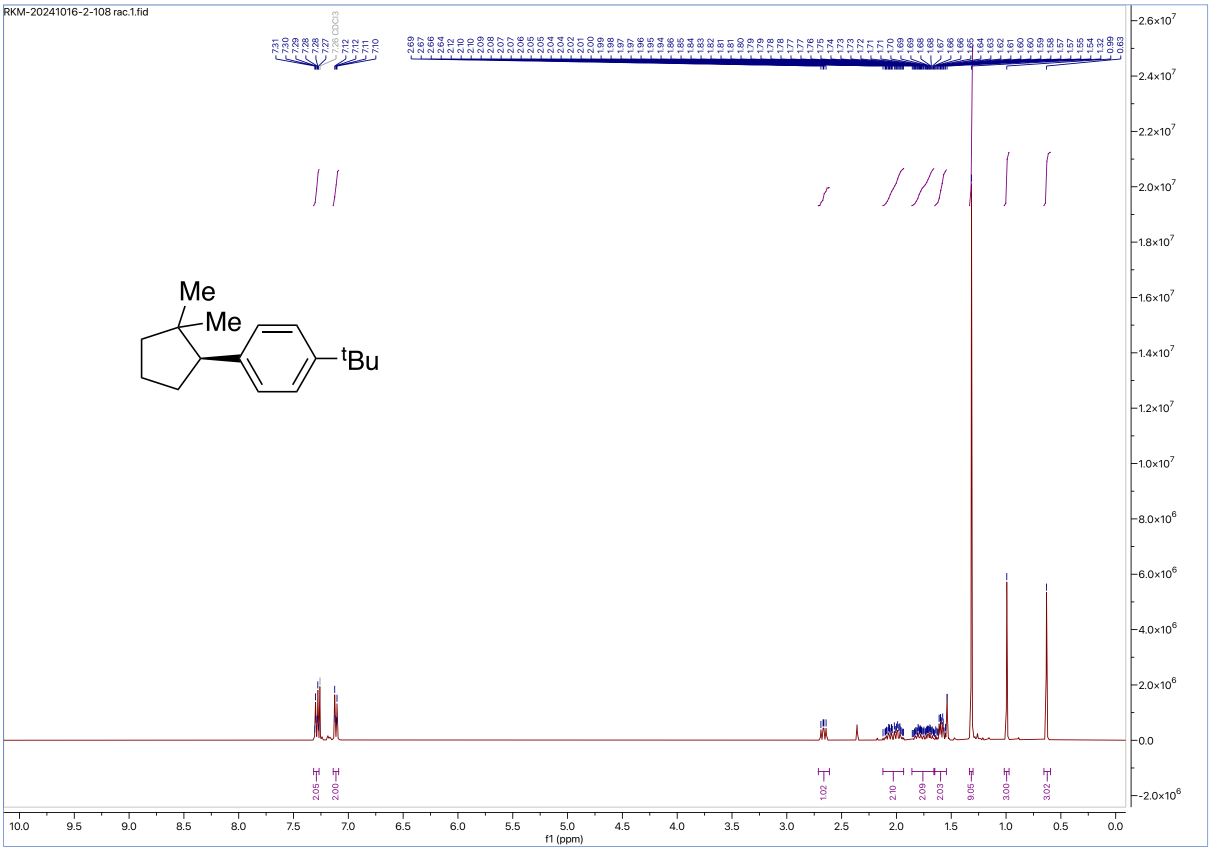
RKM-20241016-2-108 rac.1.fid
Me
Me
tBu
f1 (ppm)

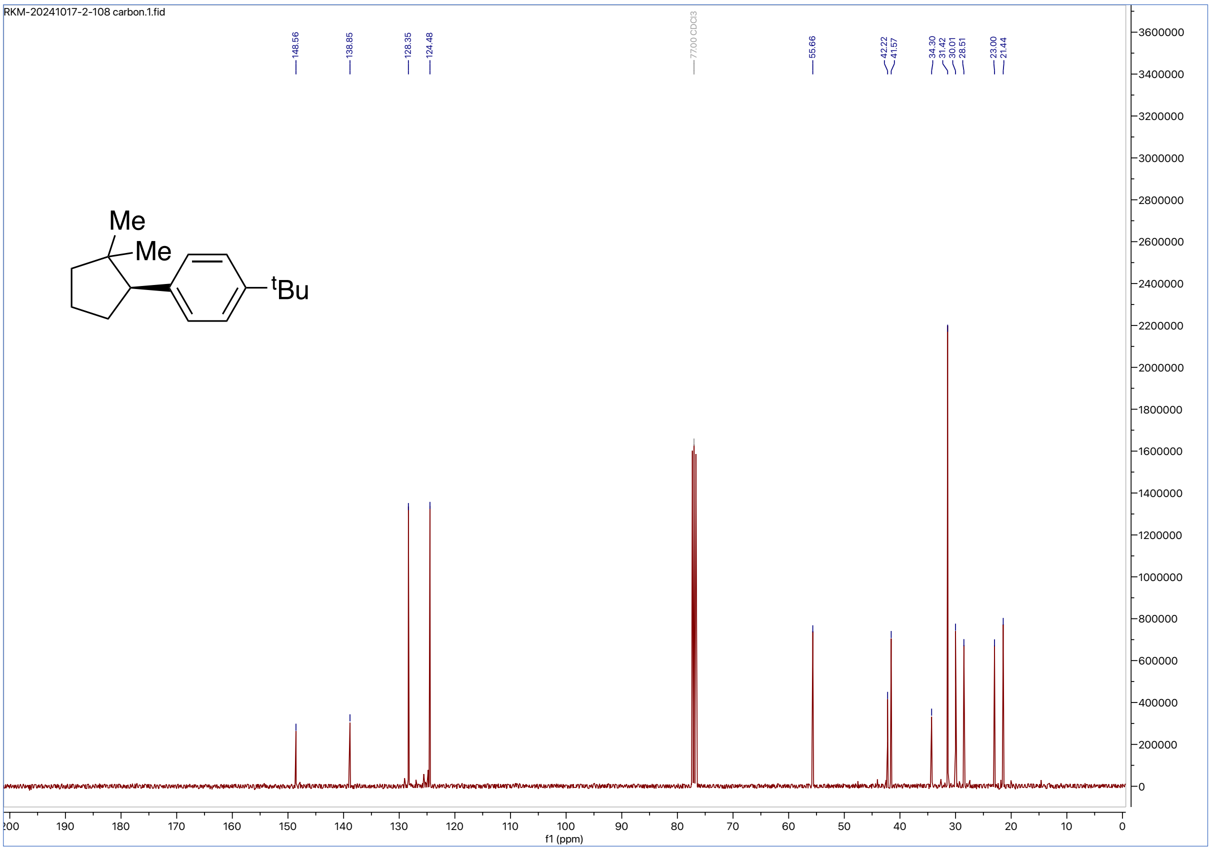
RKM-20241017-2-108 carbon.1.fid
148.56
138.85
128.35
124.48
77.00 CDCl3
55.66
42.22
41.57
34.30
31.42
30.01
28.51
23.00
21.44
Me
Me
tBu
f1 (ppm)

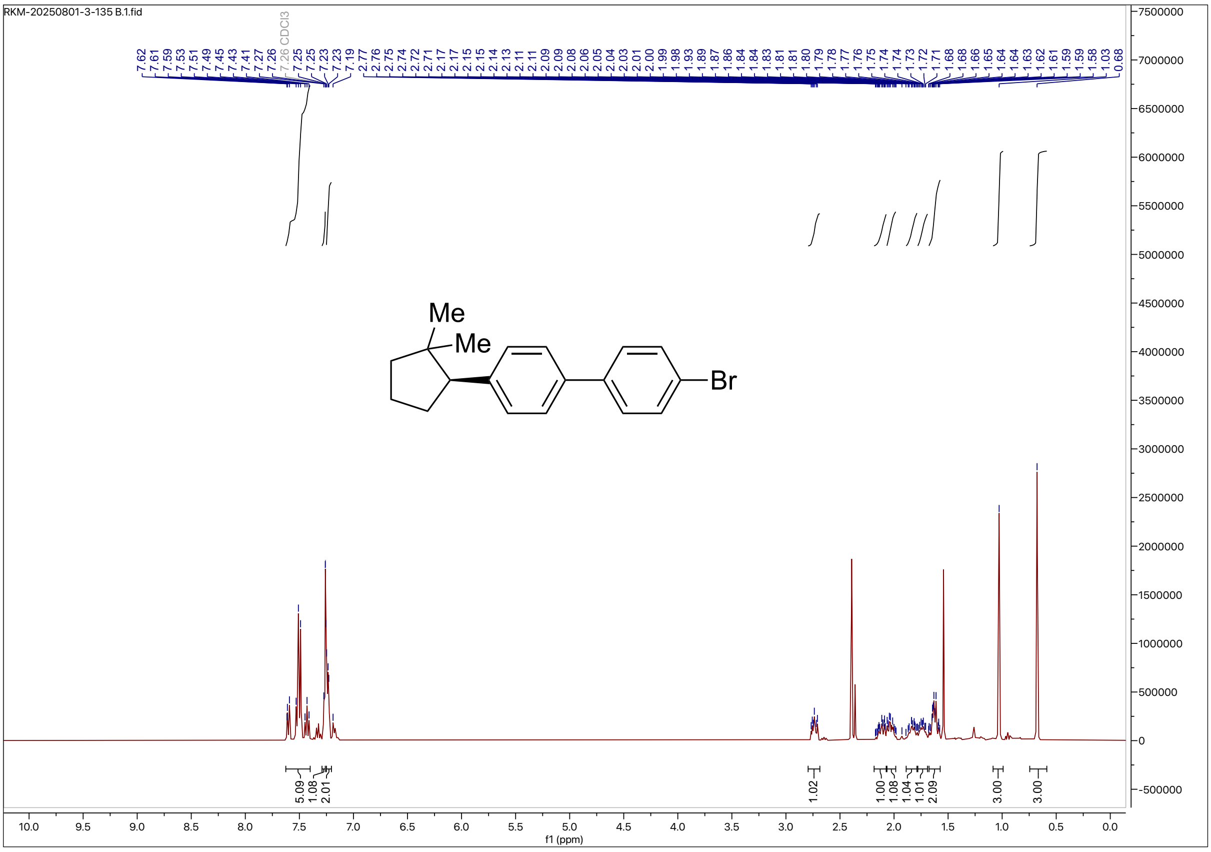
RKM-20250801-3-135 B.1.fid
Me
Me
Br
f1 (ppm)

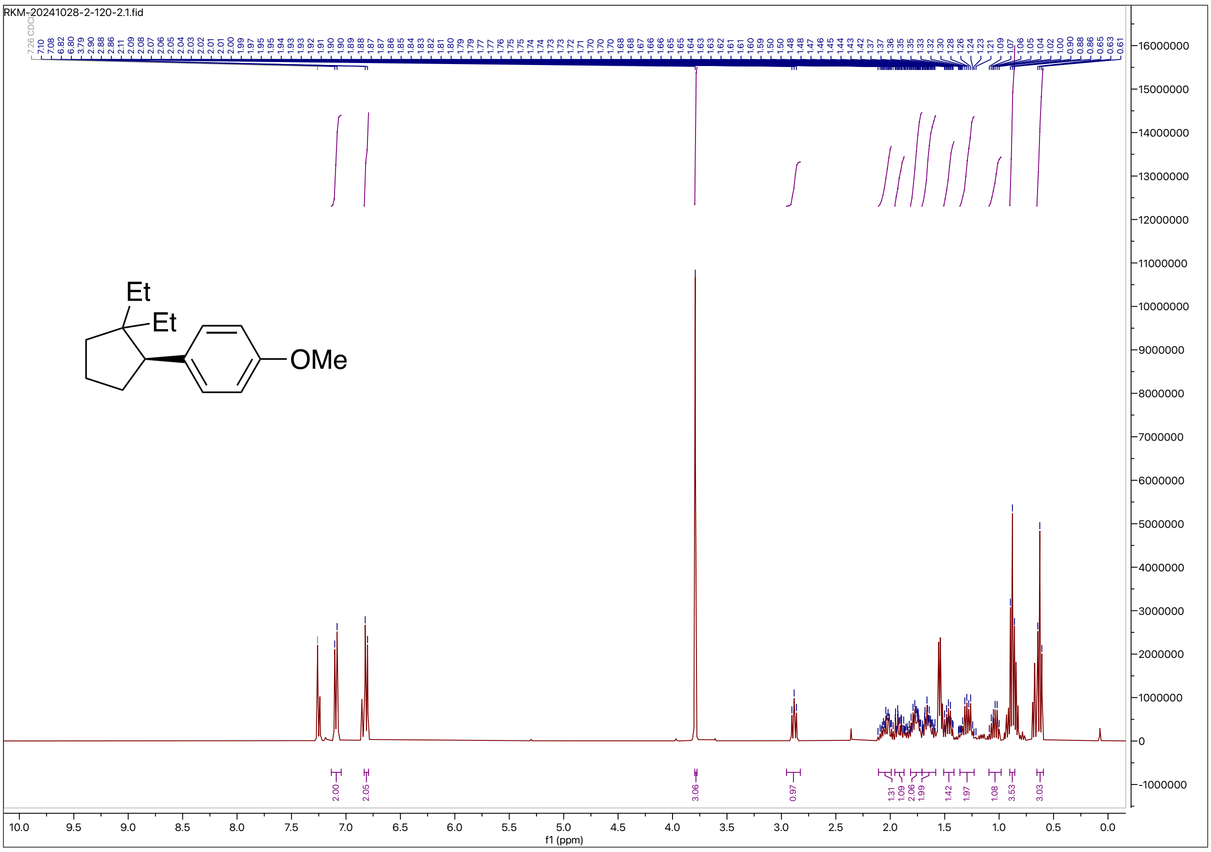
RKM-20241028-2-120-2.1.fid
Et
Et
OMe
f1 (ppm)

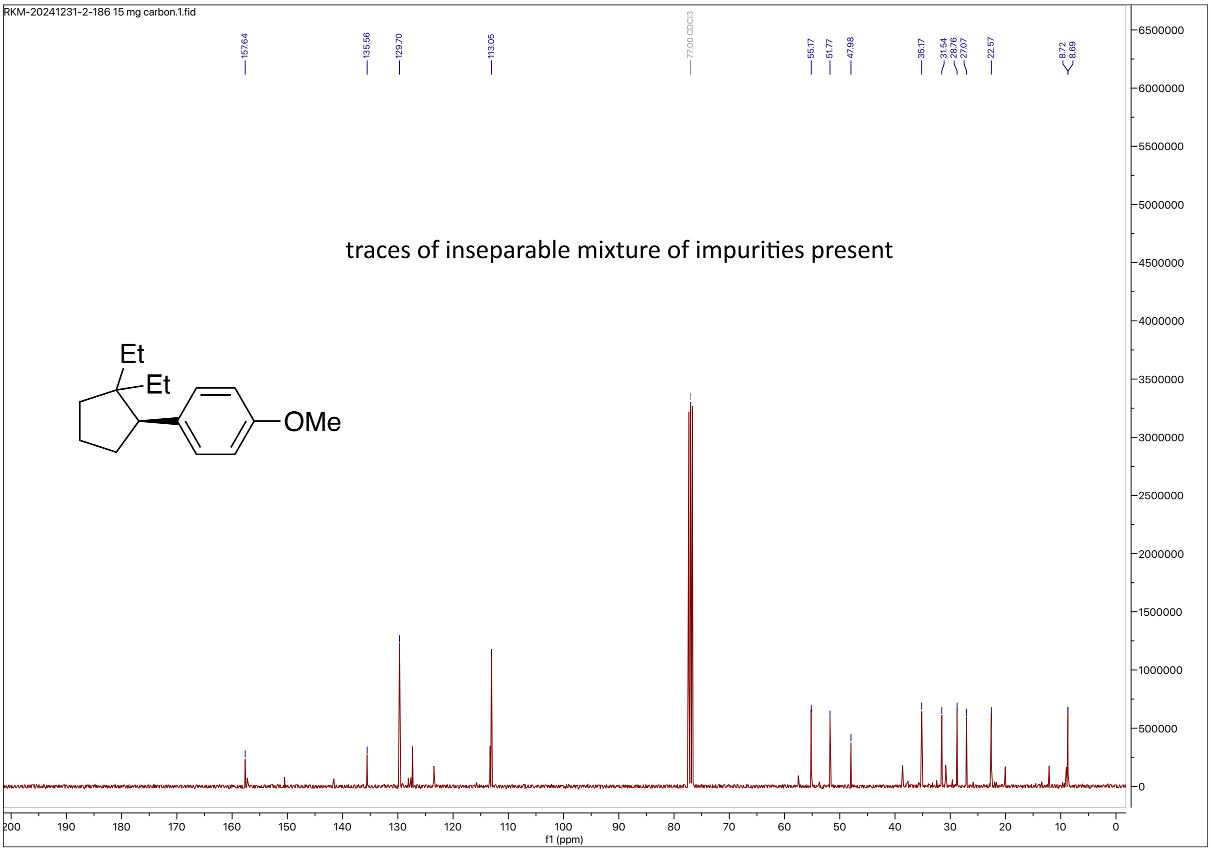
RKM-20241231-2-186 15 mg carbon.1.fid
traces of inseparable mixture of impurities present
Et
Et
OMe
f1 (ppm)

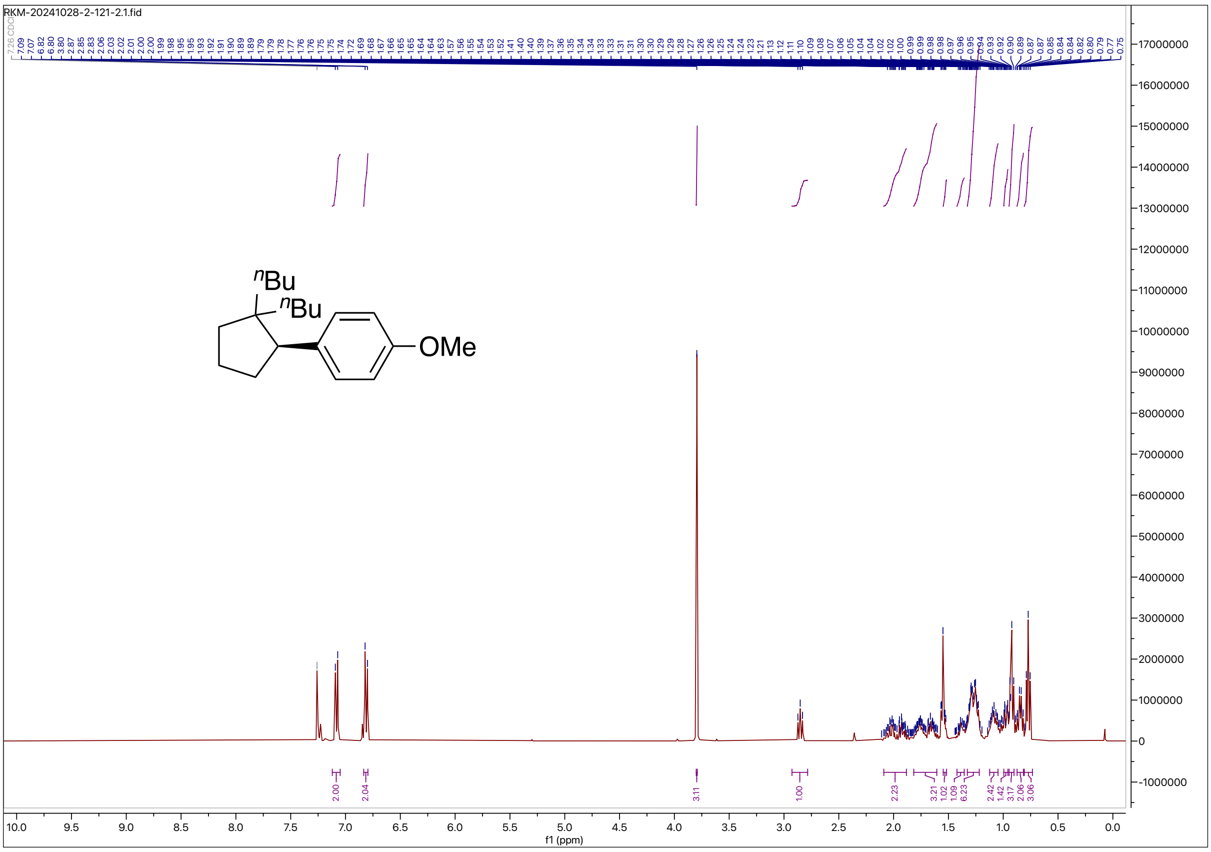
RKM-20241028-2-121-2.1.fid
^{n}Bu
^{n}Bu
OMe
f1 (ppm)

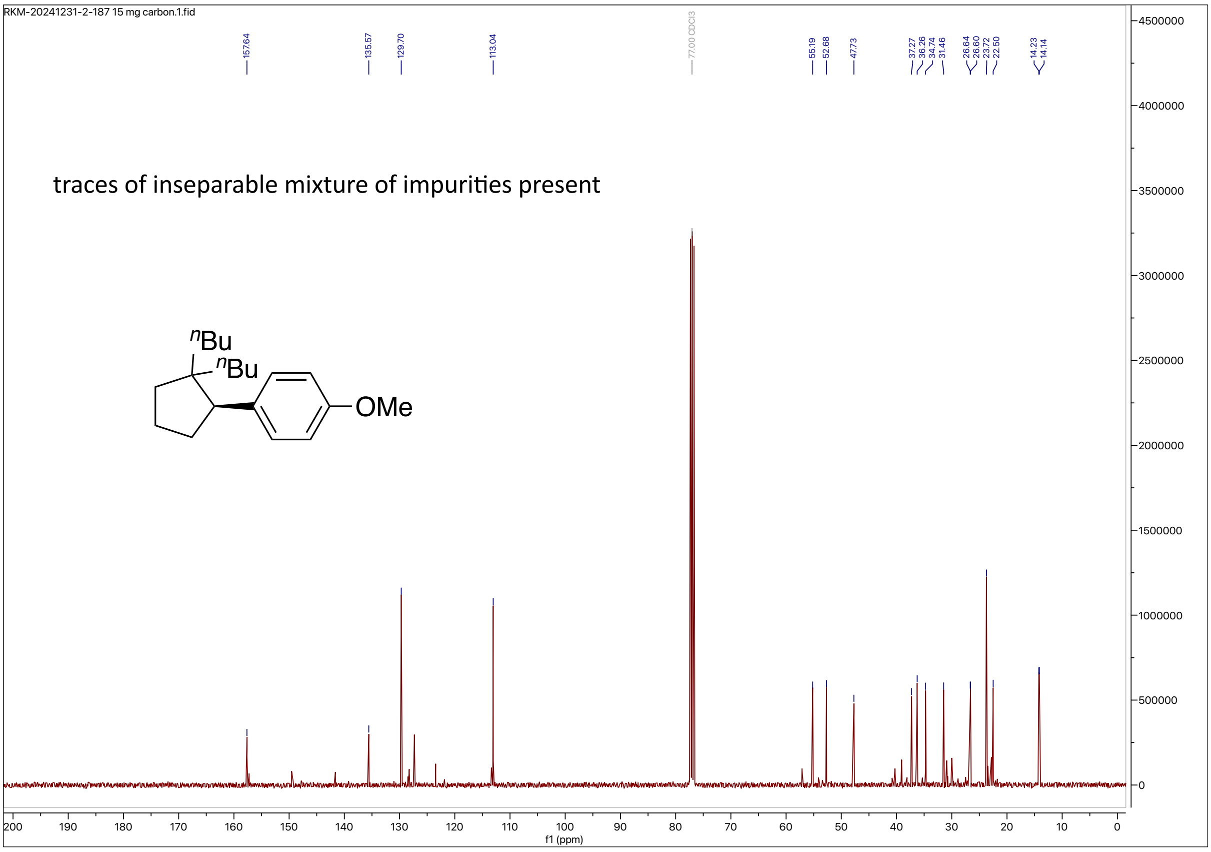
RKM-20241231-2-187 15 mg carbon.1.fid
traces of inseparable mixture of impurities present
157.64
135.57
129.70
113.04
77.00 CDCl3
55.19
52.68
47.73
37.27
36.26
34.74
31.46
26.64
26.60
23.72
22.50
14.23
14.14
OMe
f1 (ppm)

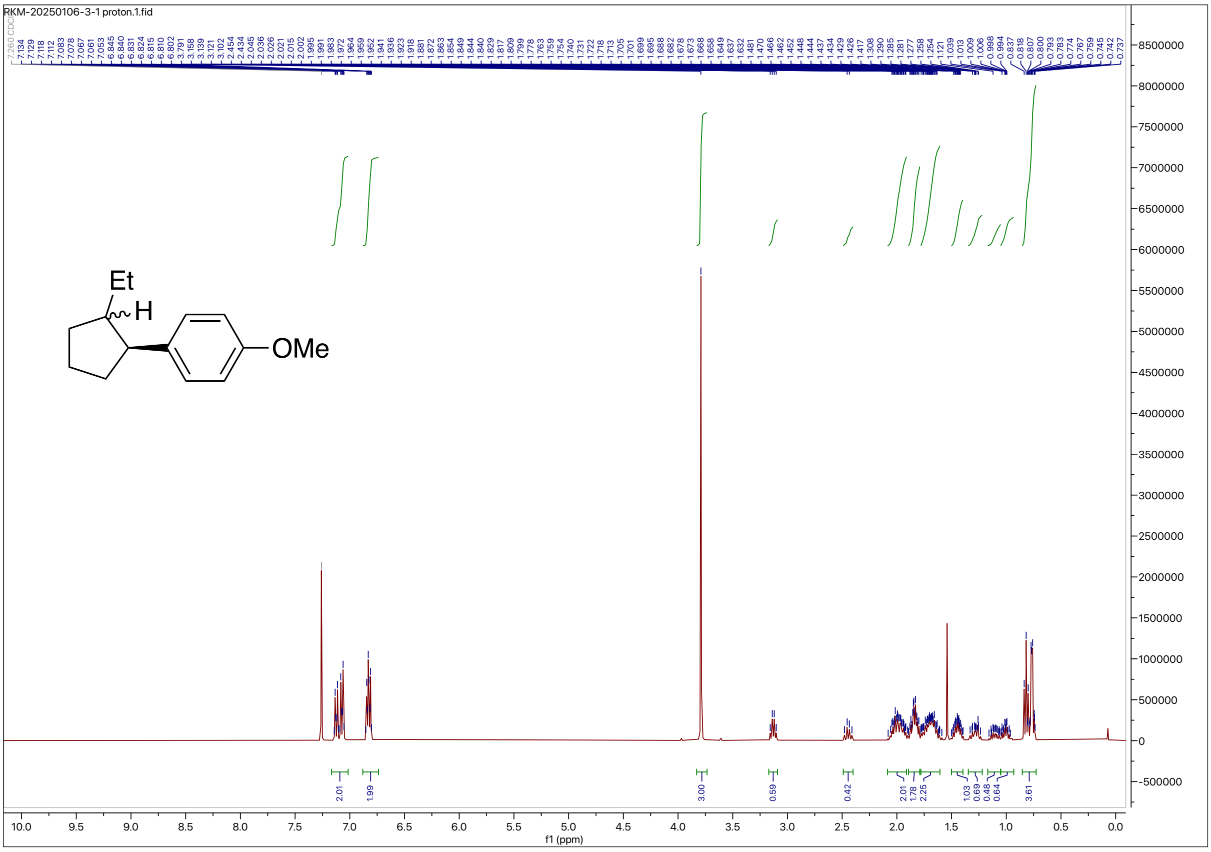
RKM-20250106-3-1 proton.1.fid
Et
H
OMe
2.01
1.99
3.00
0.59
0.42
2.01
1.78
2.25
1.03
0.69
0.48
0.64
3.61
f1 (ppm)

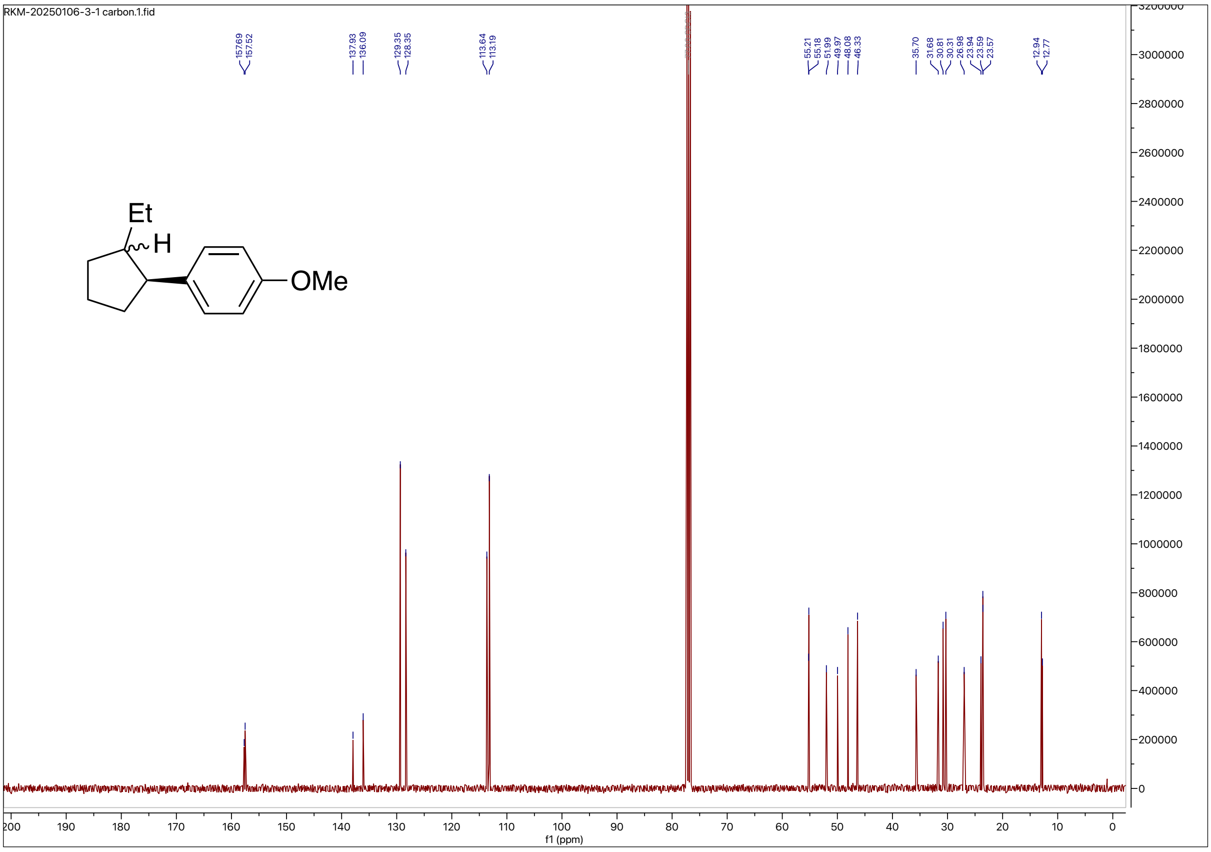

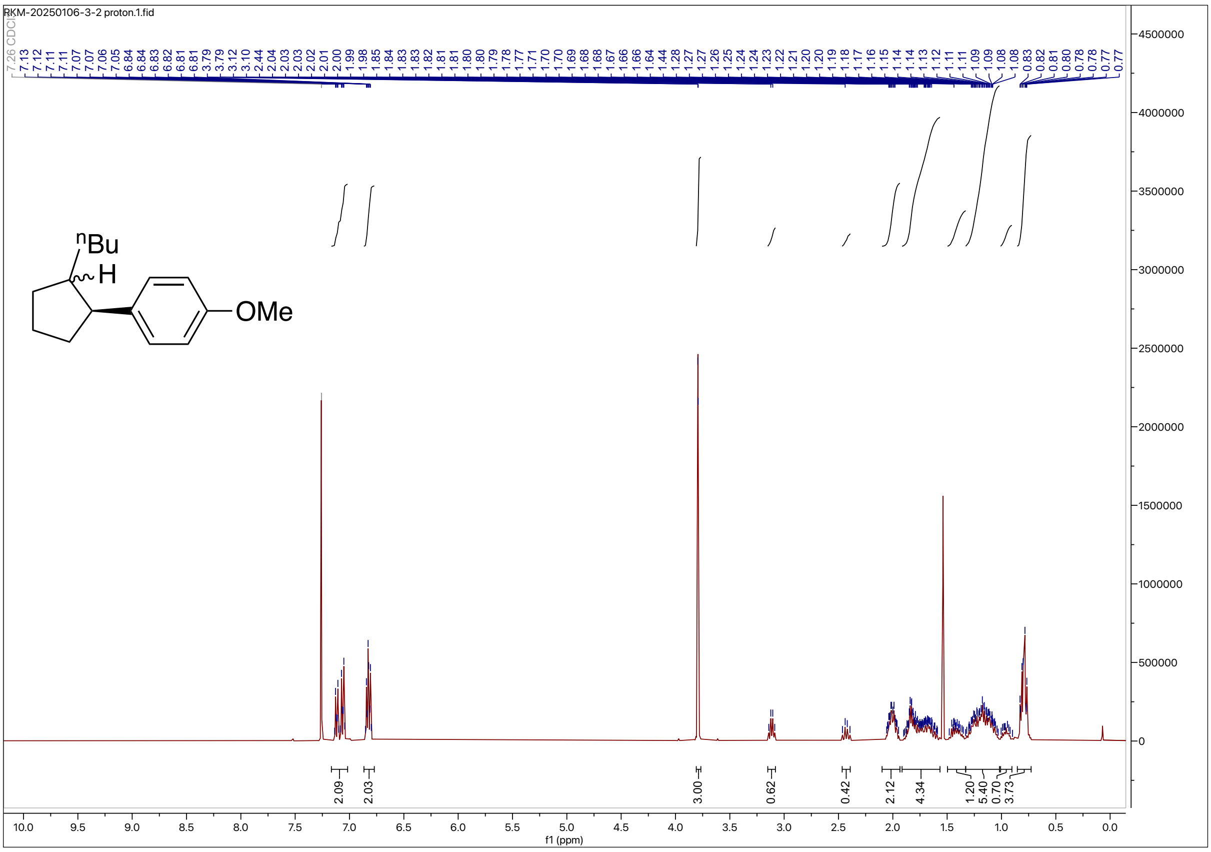

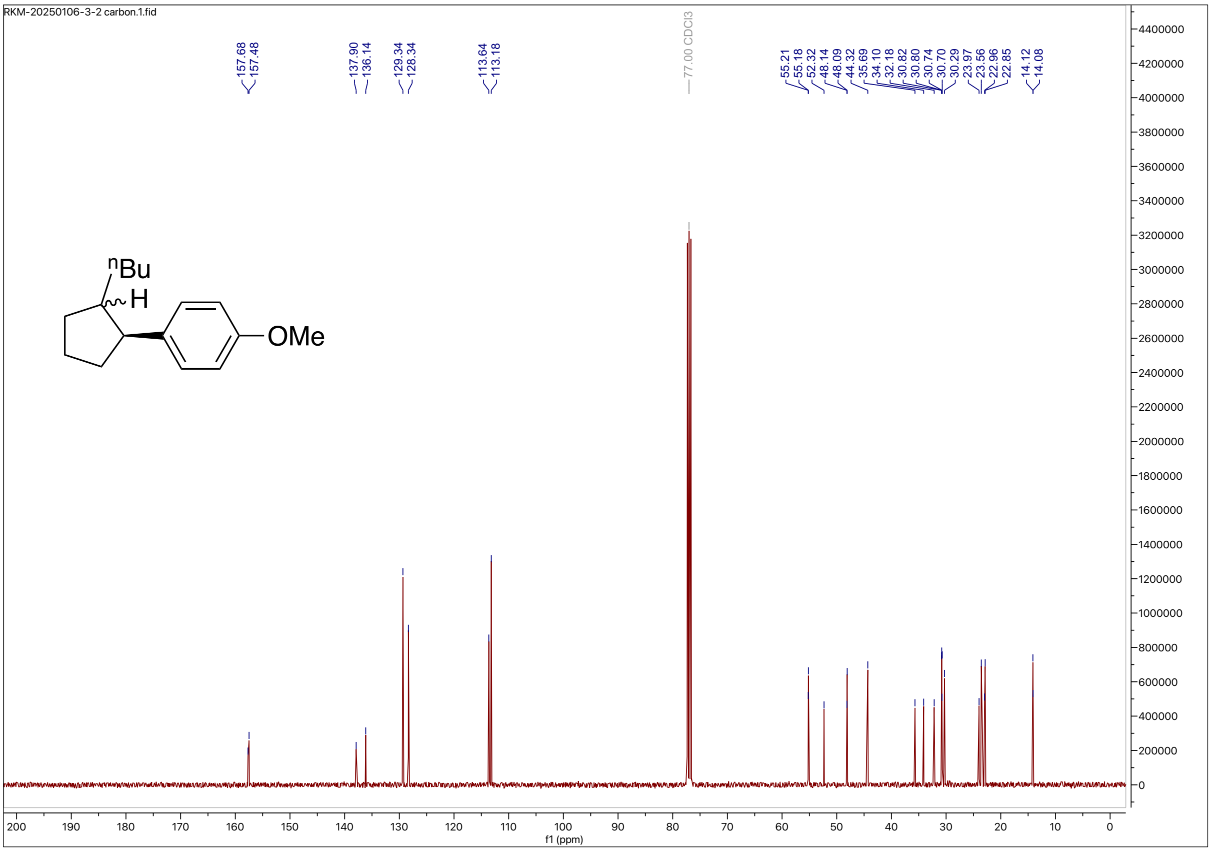

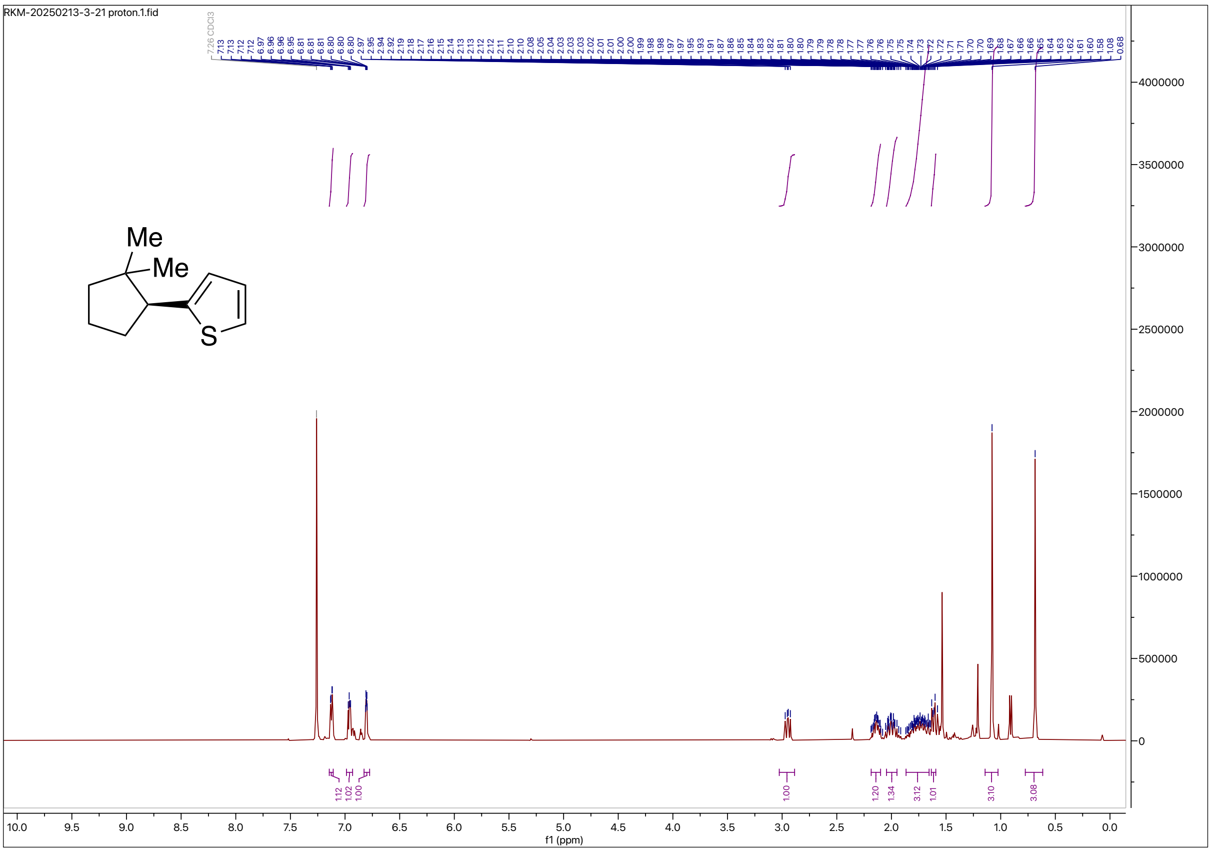

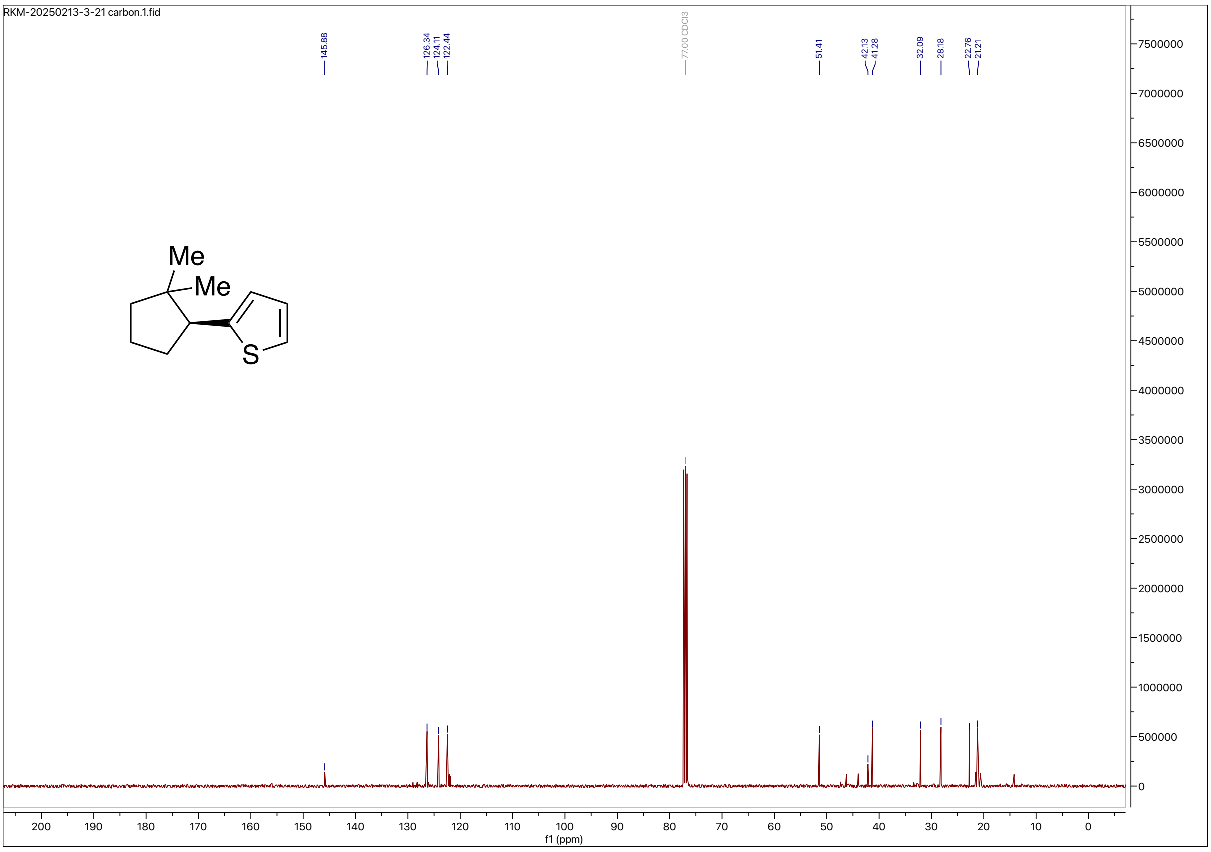
RKM-20250213-3-21 carbon.1.fid
145.88
126.34
124.11
122.44
77.00 CDCl3
51.41
42.13
41.28
32.09
28.18
22.76
21.21
Me
Me
S
f1 (ppm)

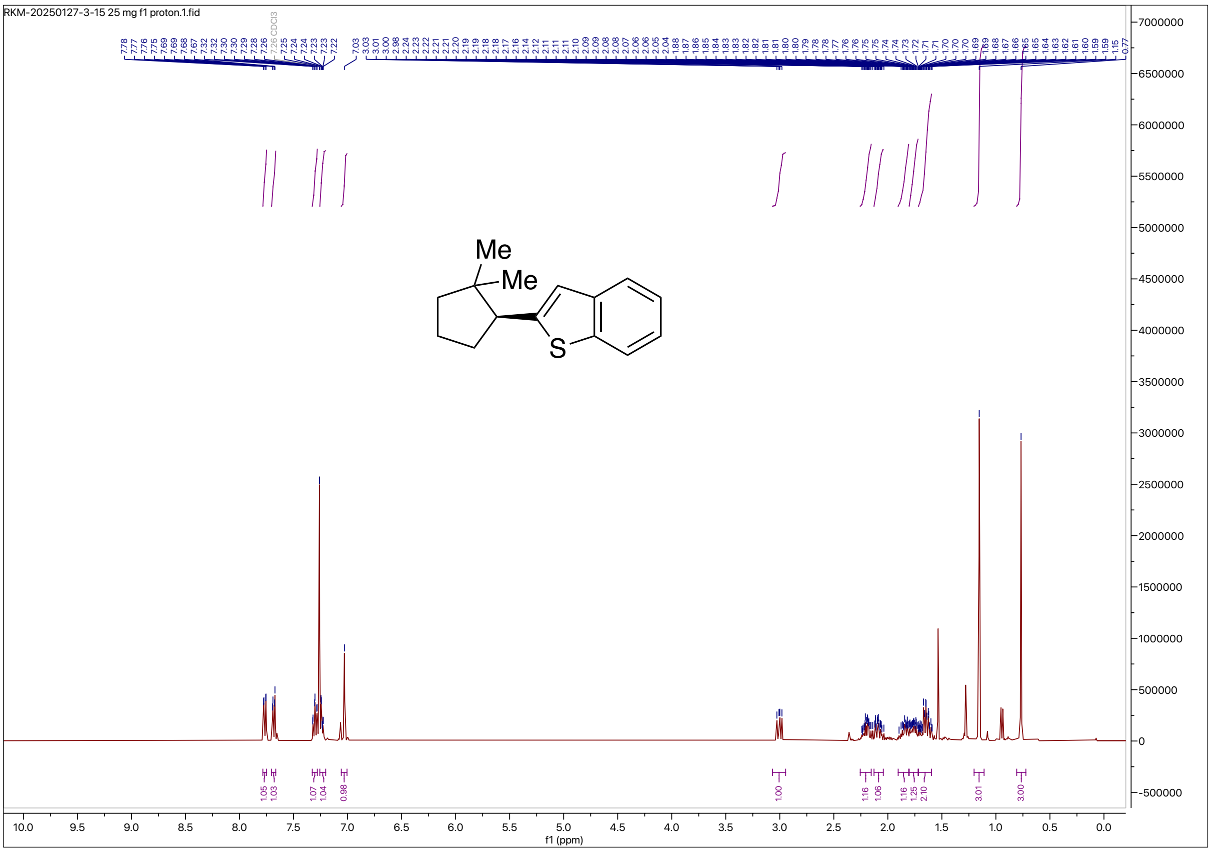
RKM-20250127-3-15 25 mg f1 proton.1.fid
Me
Me
S
1.05
1.03
1.07
1.04
0.98
1.00
1.16
1.06
1.16
1.25
2.10
3.01
3.00
f1 (ppm)

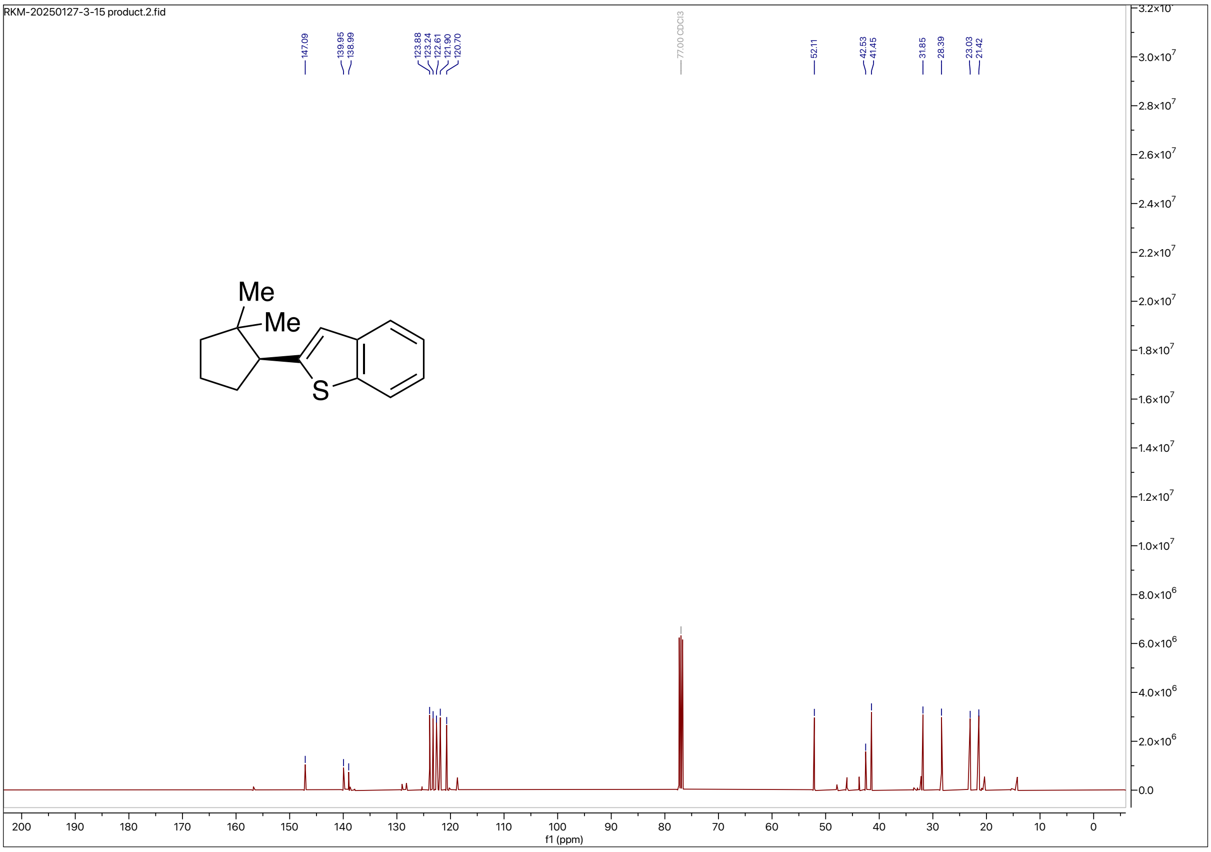
RKM-20250127-3-15 product.2.fid
147.09
139.95
138.99
123.88
123.24
122.61
121.90
120.70
77.00 CDCl3
52.11
42.53
41.45
31.85
28.39
23.03
21.42
Me
Me
S
f1 (ppm)

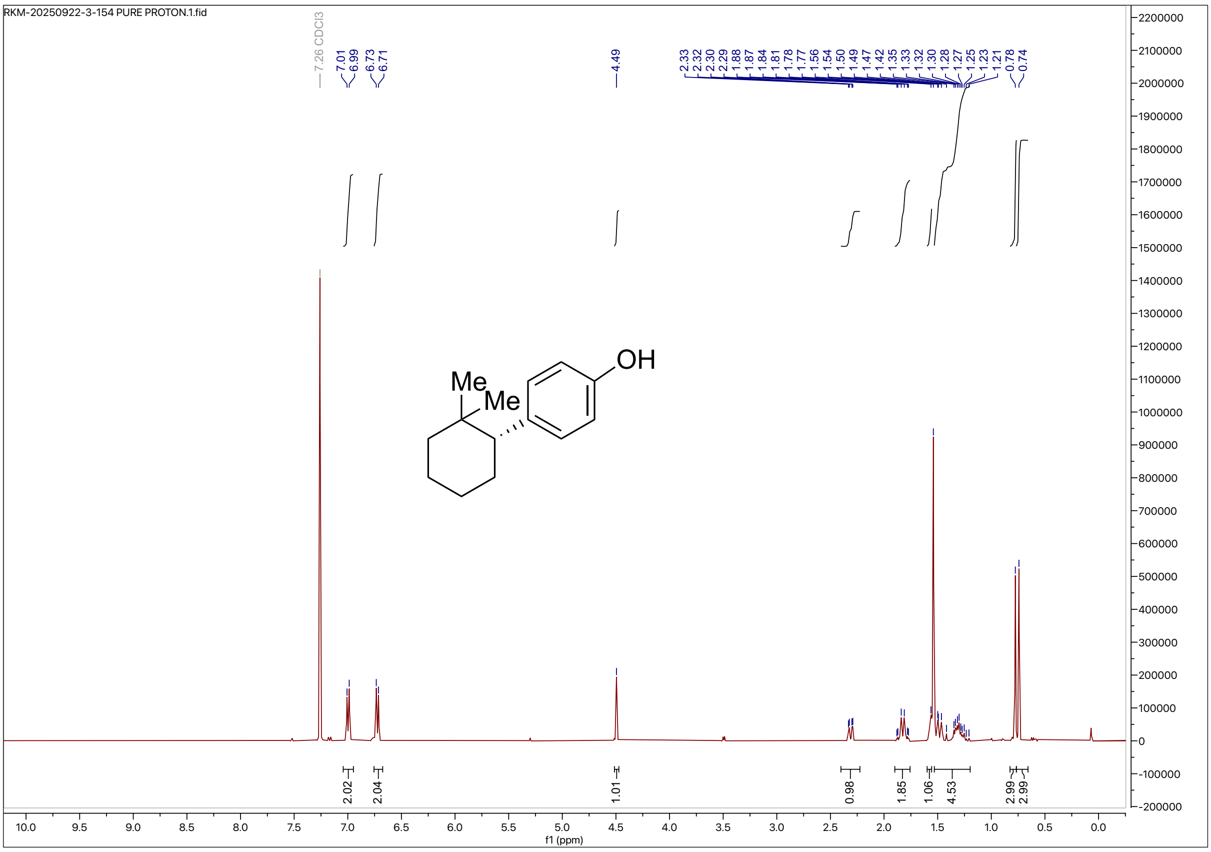
RKM-20250922-3-154 PURE PROTON.1.fid
7.26 CDCl3
7.01
6.99
6.73
6.71
4.49
2.33
2.32
2.30
2.29
1.88
1.87
1.84
1.81
1.78
1.77
1.56
1.54
1.50
1.49
1.47
1.42
1.35
1.33
1.32
1.30
1.28
1.27
1.25
1.23
1.21
0.78
0.74
OH
Me
Me
2.02
2.04
1.01
0.98
1.85
1.06
4.53
2.99
2.99
f1 (ppm)

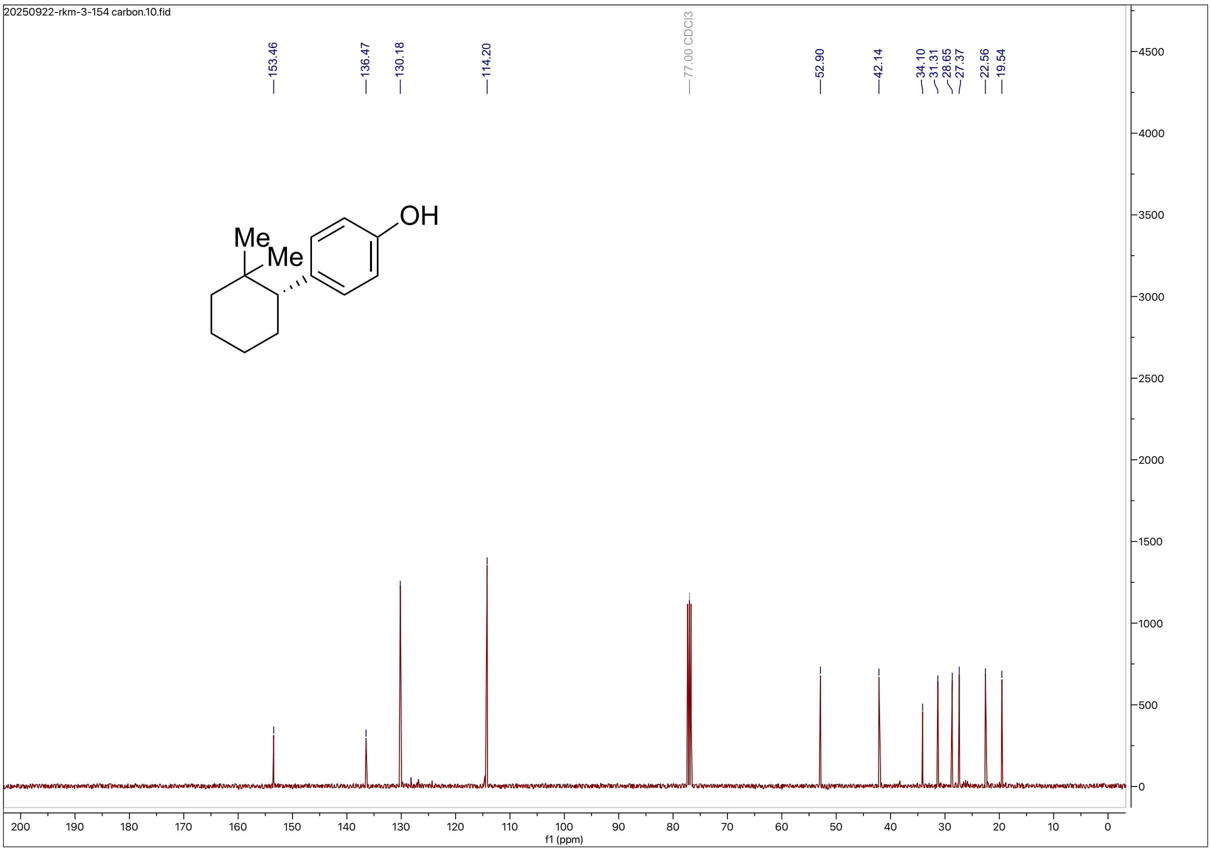
20250922-rkm-3-154 carbon.10.fid
153.46
136.47
130.18
114.20
77.00 CDCl3
52.90
42.14
34.10
31.31
28.65
27.37
22.56
19.54
OH
Me
Me
f1 (ppm)

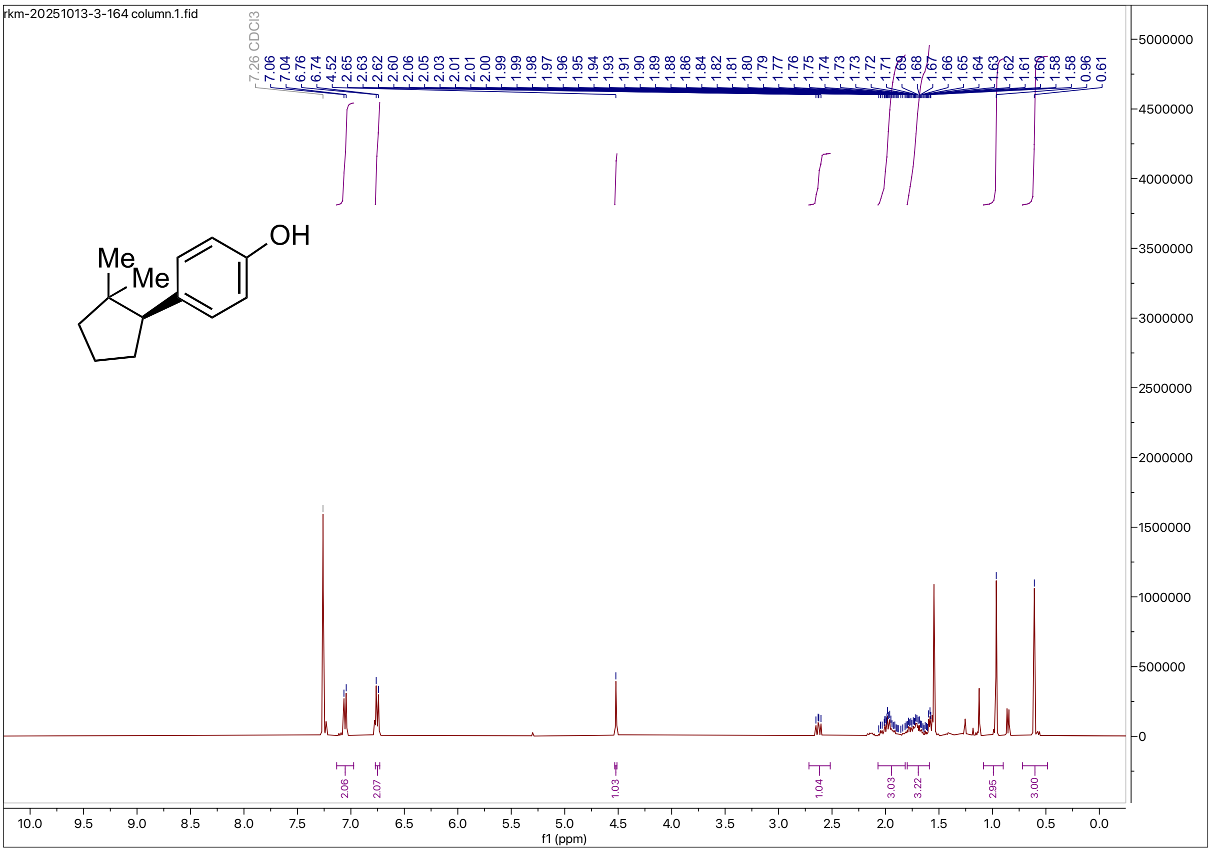
rkm-20251013-3-164 column.1.fid
7.26 CDCl3
OH
Me
Me
2.06
2.07
1.03
1.04
3.03
3.22
2.95
3.00
f1 (ppm)

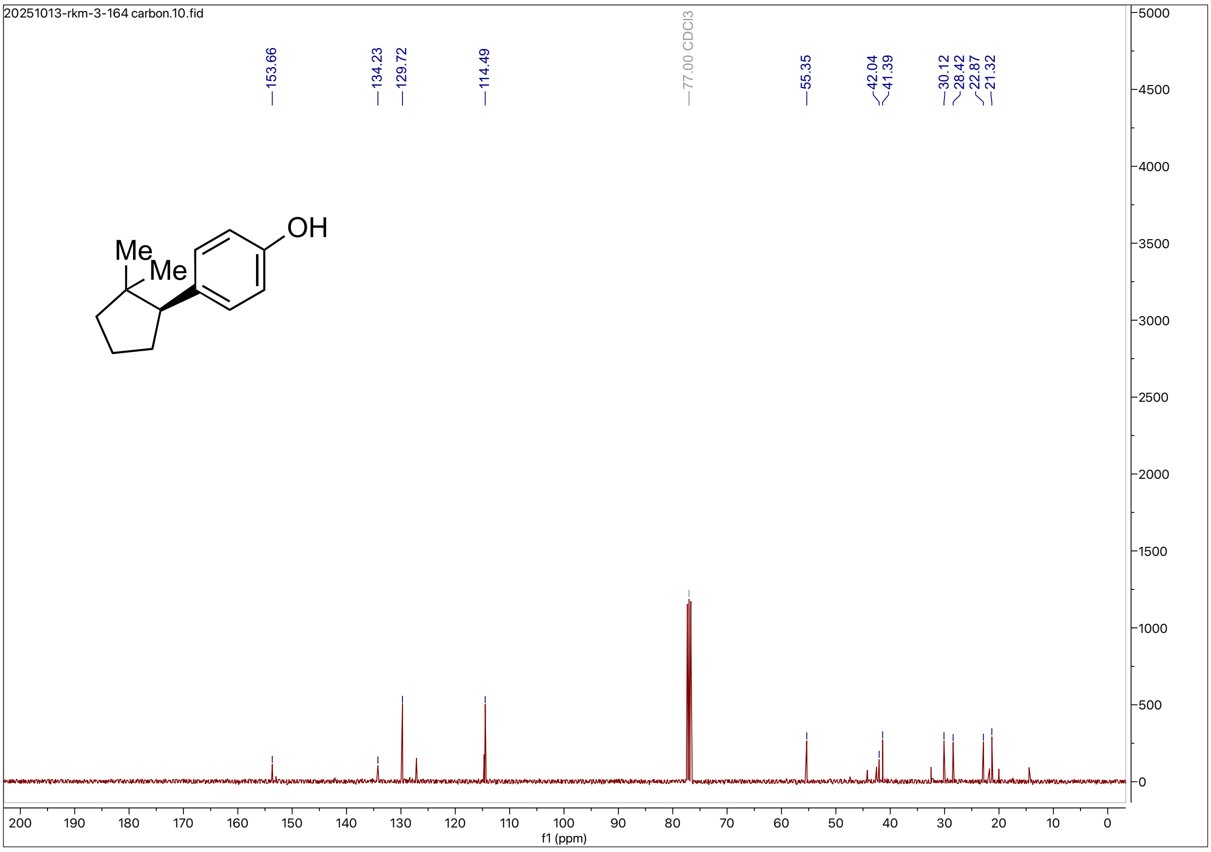
20251013-rkm-3-164 carbon.10.fid
153.66
134.23
129.72
114.49
77.00 CDCl3
55.35
42.04
41.39
30.12
28.42
22.87
21.32
Me
Me
OH
f1 (ppm)

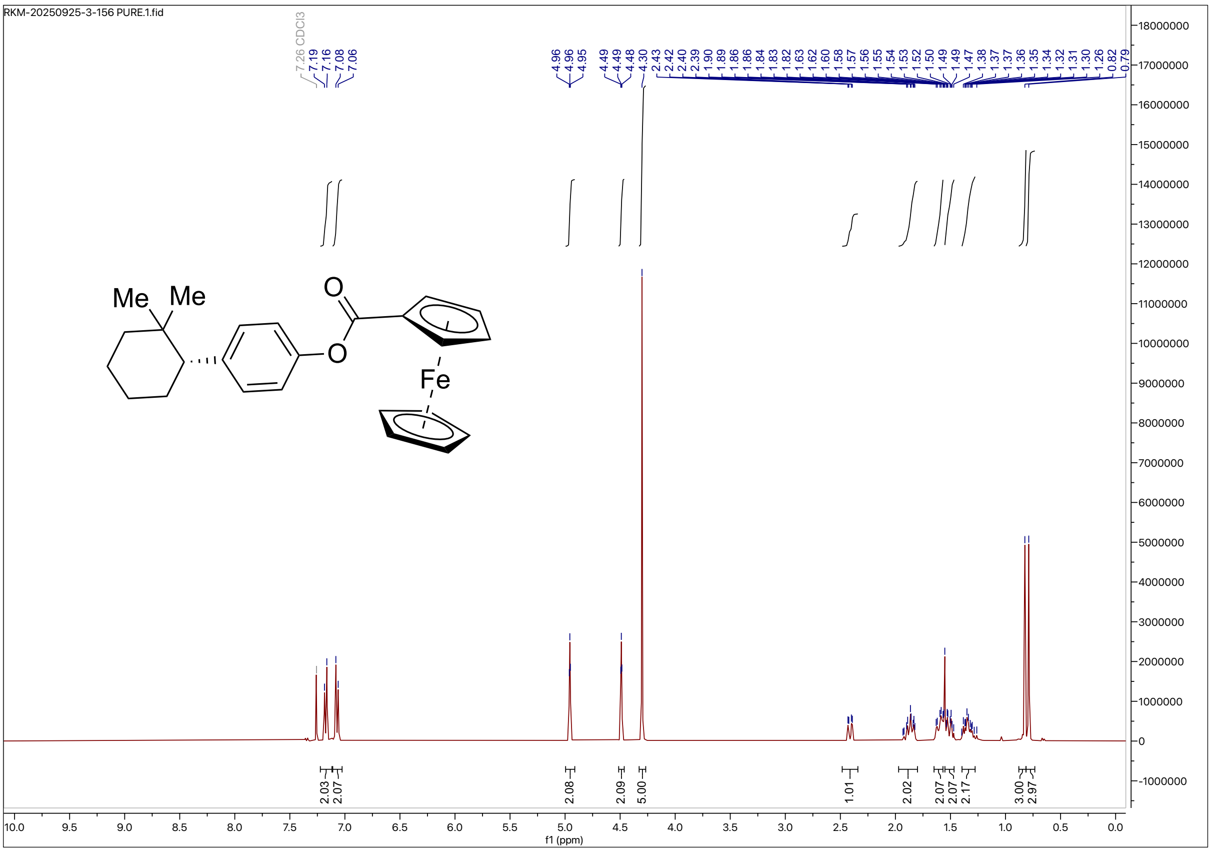
RKM-20250925-3-156 PURE.1.fid
7.26 CDCl3
7.19
7.16
7.08
7.06
4.96
4.96
4.95
4.49
4.49
4.48
4.30
Me
Me
O
O
Fe
2.03
2.07
2.08
2.09
5.00
1.01
2.02
2.07
2.07
2.17
3.00
2.97
f1 (ppm)

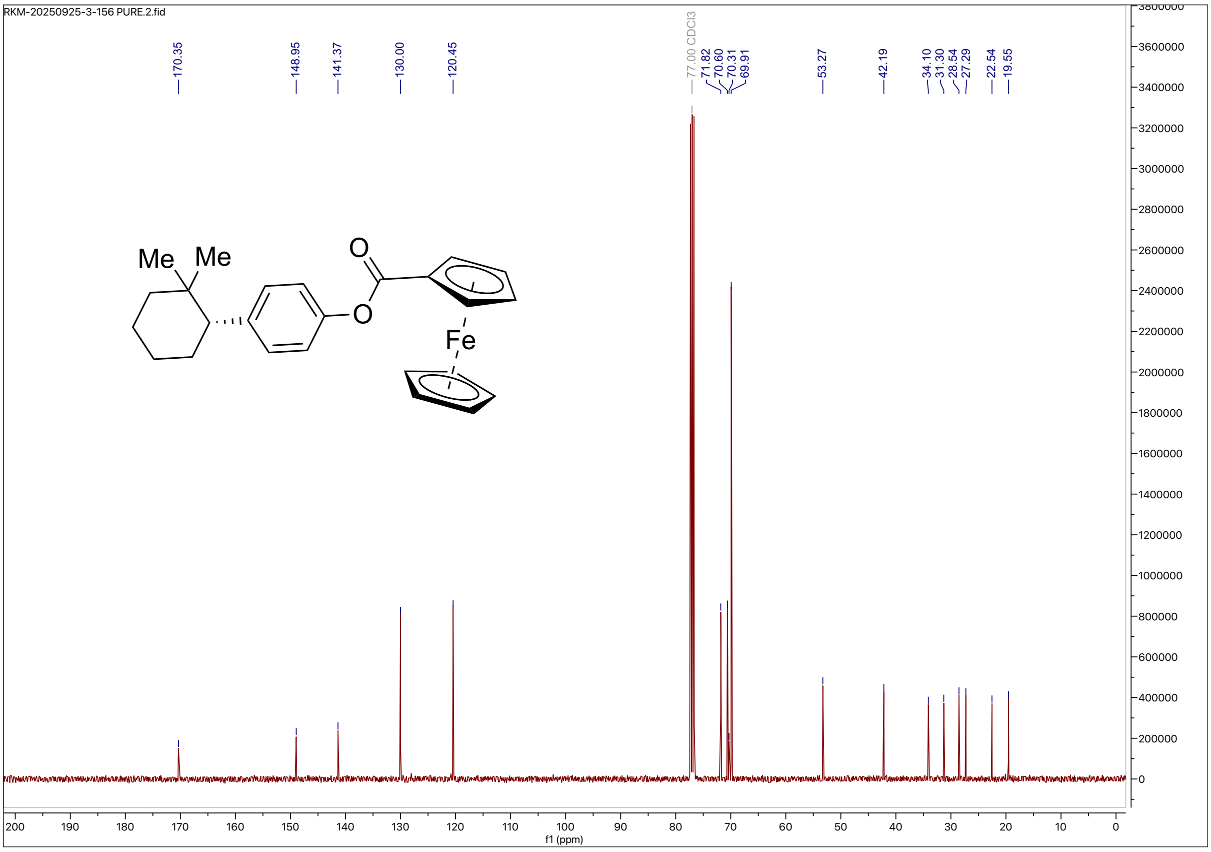
RKM-20250925-3-156 PURE.2.fid
170.35
148.95
141.37
130.00
120.45
77.00 CDCl3
71.82
70.60
70.31
69.91
53.27
42.19
34.10
31.30
28.54
27.29
22.54
19.55
Me
Me
O
O
Fe
f1 (ppm)

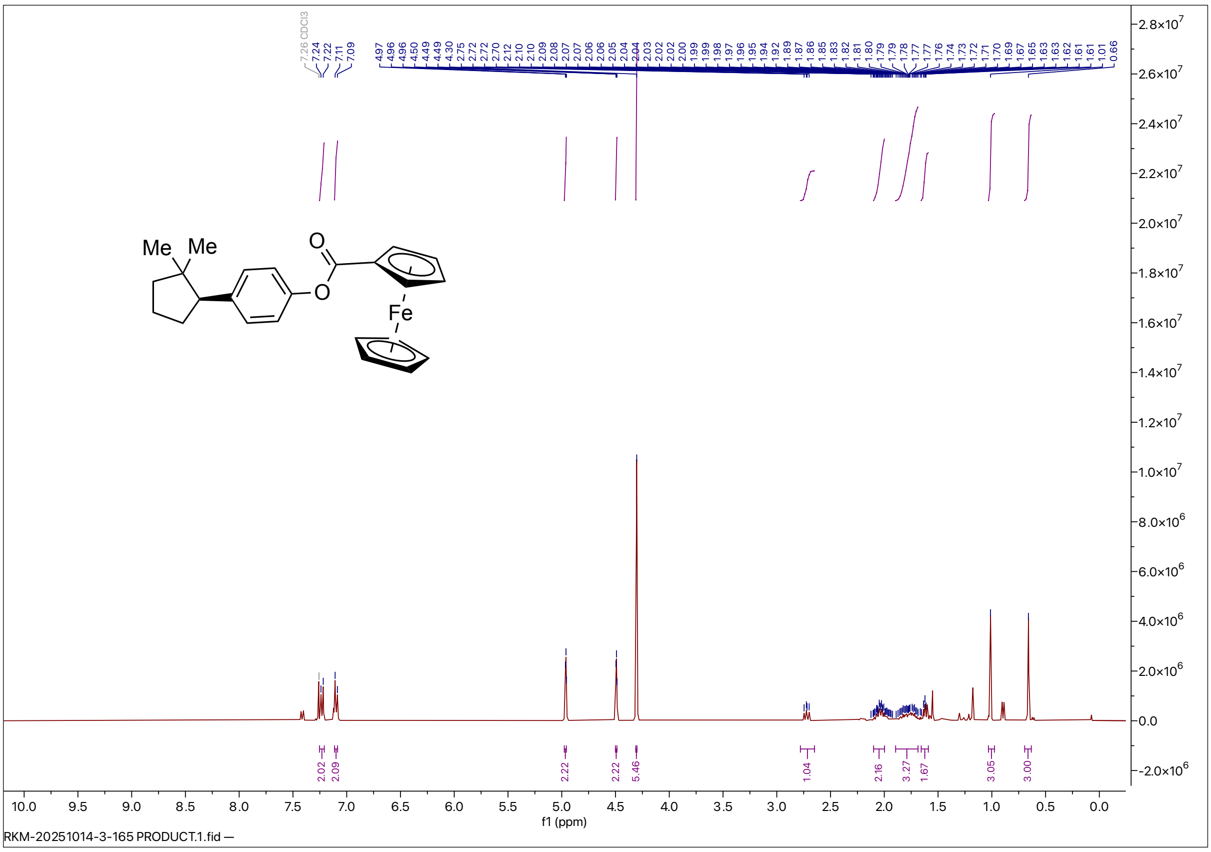
7.26 CDCl3
Me
Me
O
O
Fe
2.02
2.09
2.22
2.22
5.46
1.04
2.16
3.27
1.67
3.05
3.00
f1 (ppm)
RKM-20251014-3-165 PRODUCT.1.fid

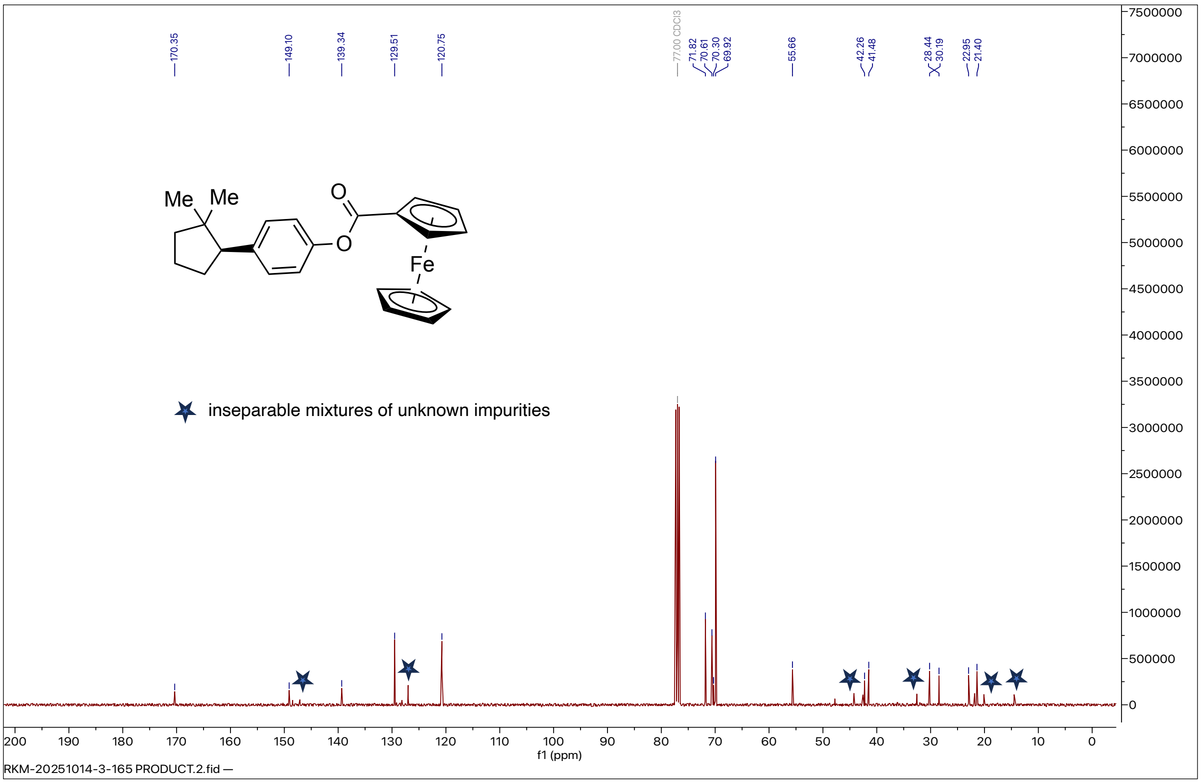

170.35
149.10
139.34
129.51
120.75
77.00 CDCl3
71.82
70.61
70.30
69.92
55.66
42.26
41.48
28.44
30.19
22.95
21.40
Me
Me
O
O
Fe
inseparable mixtures of unknown impurities
f1 (ppm)
RKM-20251014-3-165 PRODUCT.2.fid —

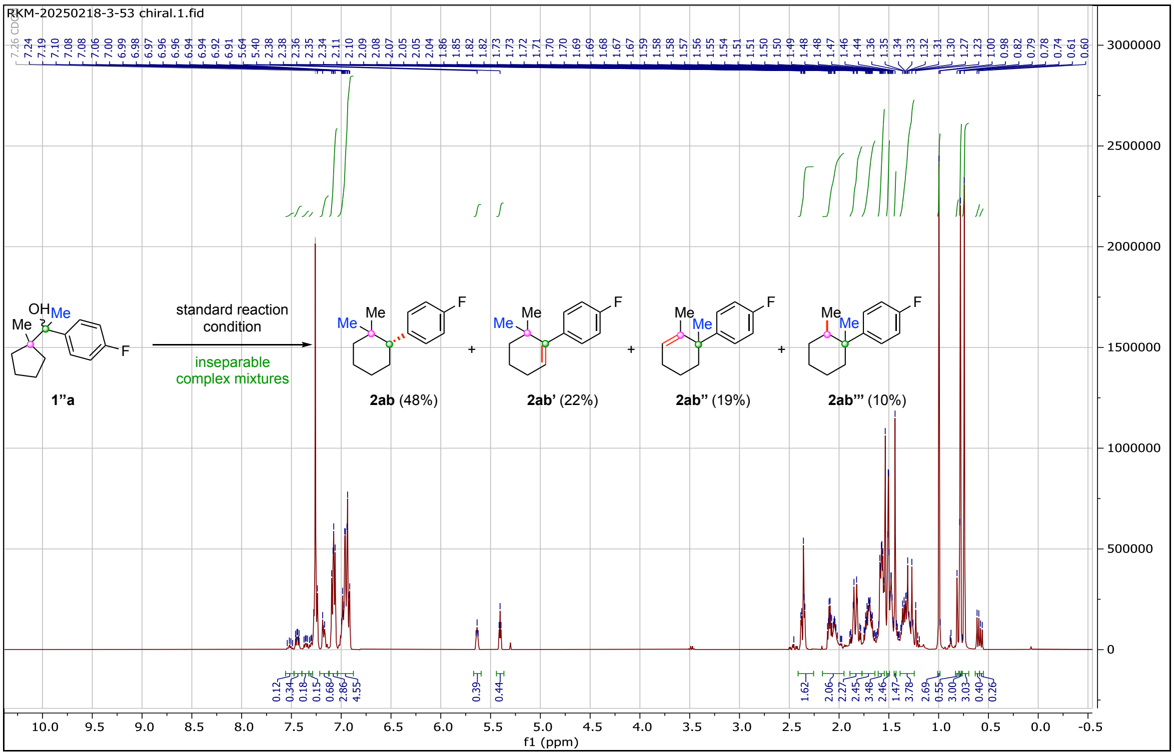
RKM-20250218-3-53 chiral.1.fid
standard reaction
condition
inseparable
complex mixtures
1''a
2ab (48%)
2ab' (22%)
2ab'' (19%)
2ab''' (10%)
f1 (ppm)

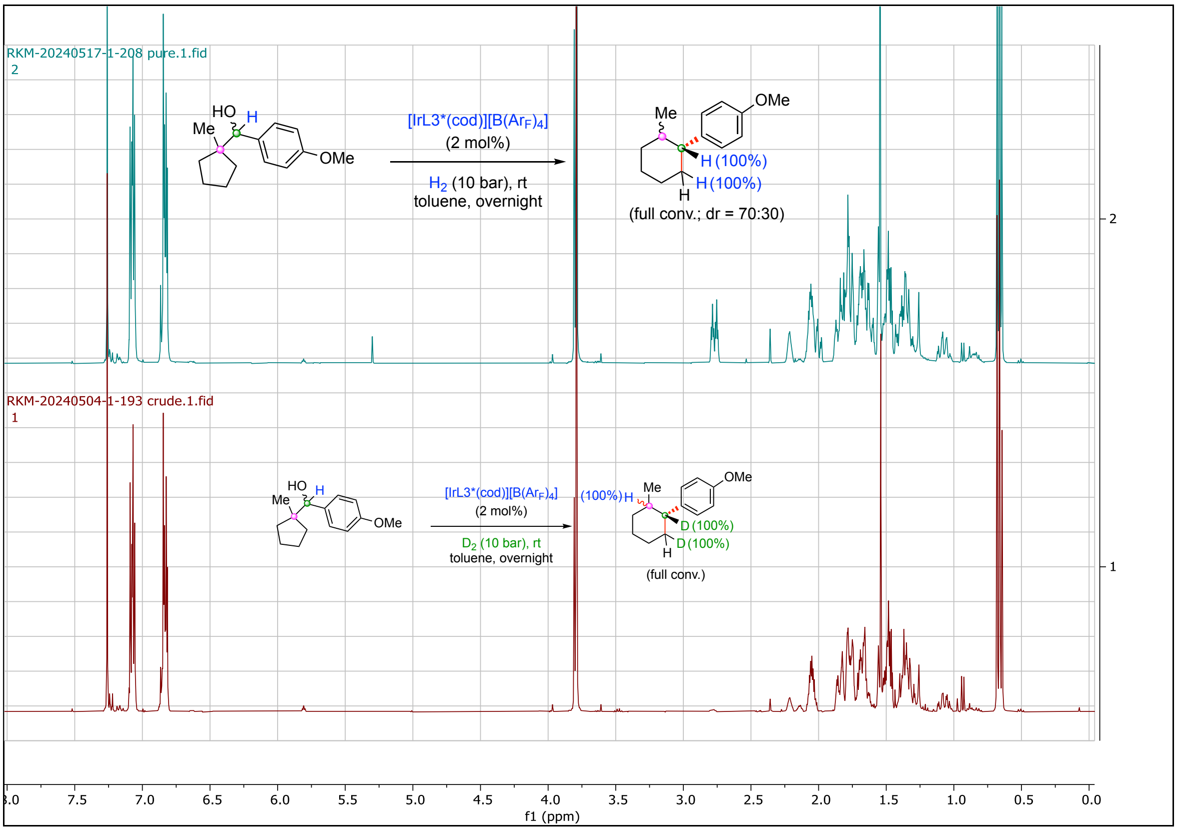
RKM-20240517-1-208 pure.1.fid
[IrL3*(cod)][B(ArF)4]
(2 mol%)
H2 (10 bar), rt
toluene, overnight
(full conv.; dr = 70:30)
RKM-20240504-1-193 crude.1.fid
[IrL3*(cod)][B(ArF)4]
(2 mol%)
D2 (10 bar), rt
toluene, overnight
(full conv.)
f1 (ppm)

SFC chromatograms

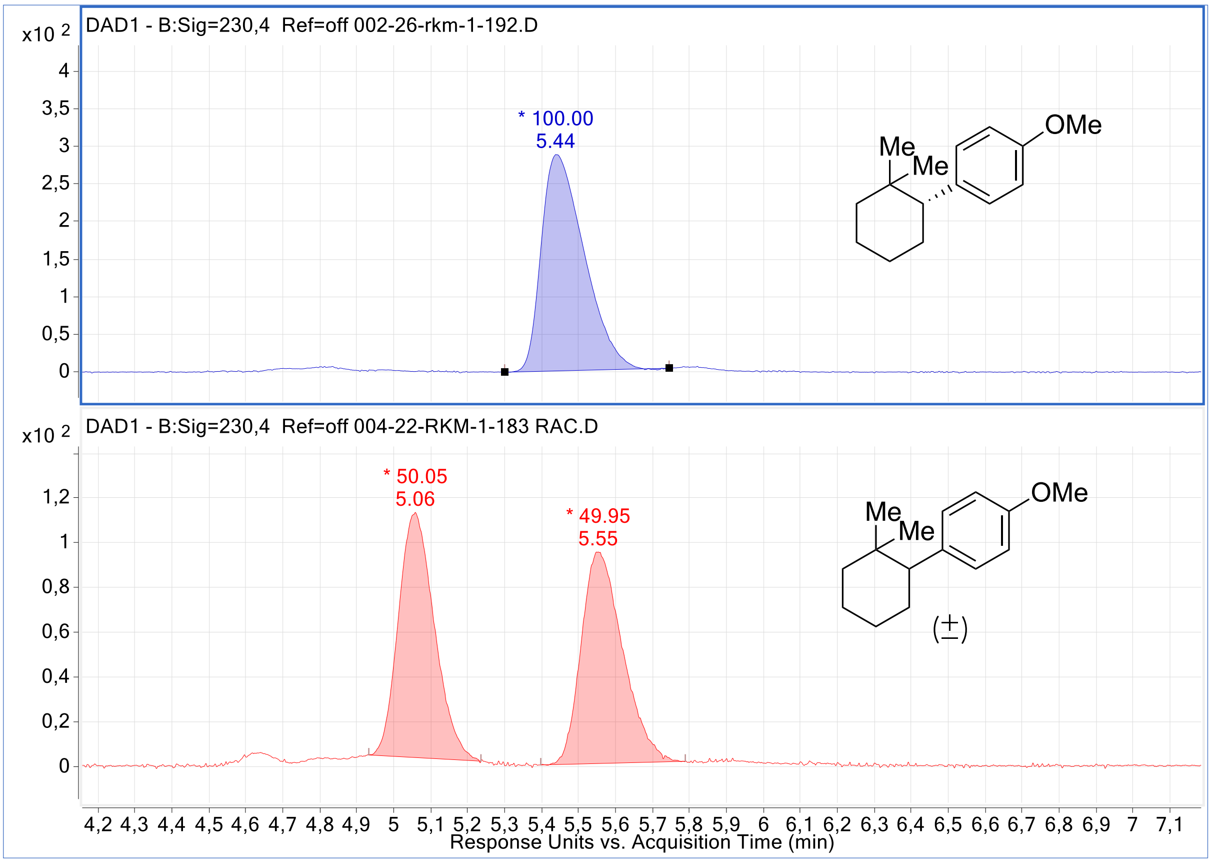

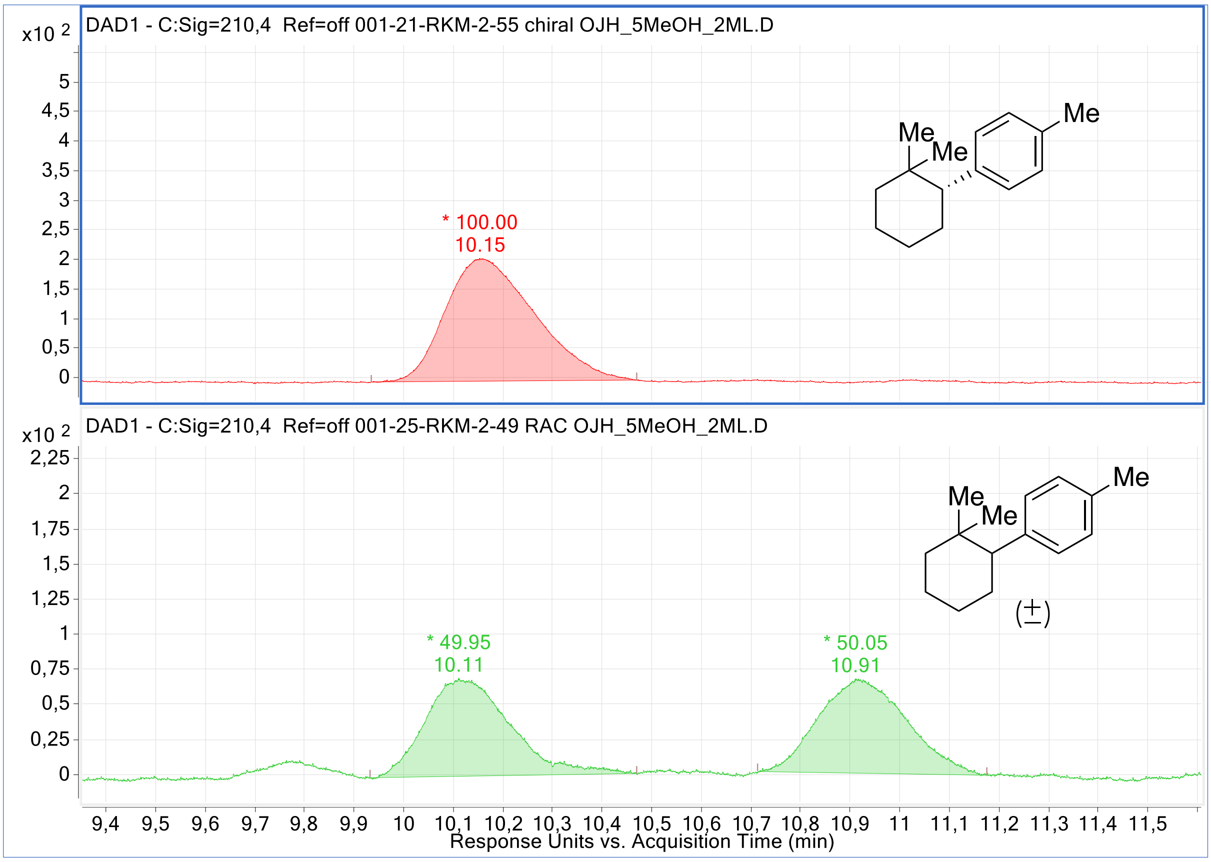
x10 2
DAD1 - C:Sig=210,4 Ref=off 001-21-RKM-2-55 chiral OJH_5MeOH_2ML.D
* 100.00
10.15
Me
Me
Me
x10 2
DAD1 - C:Sig=210,4 Ref=off 001-25-RKM-2-49 RAC OJH_5MeOH_2ML.D
* 49.95
10.11
* 50.05
10.91
Me
Me
Me
(±)
Response Units vs. Acquisition Time (min)

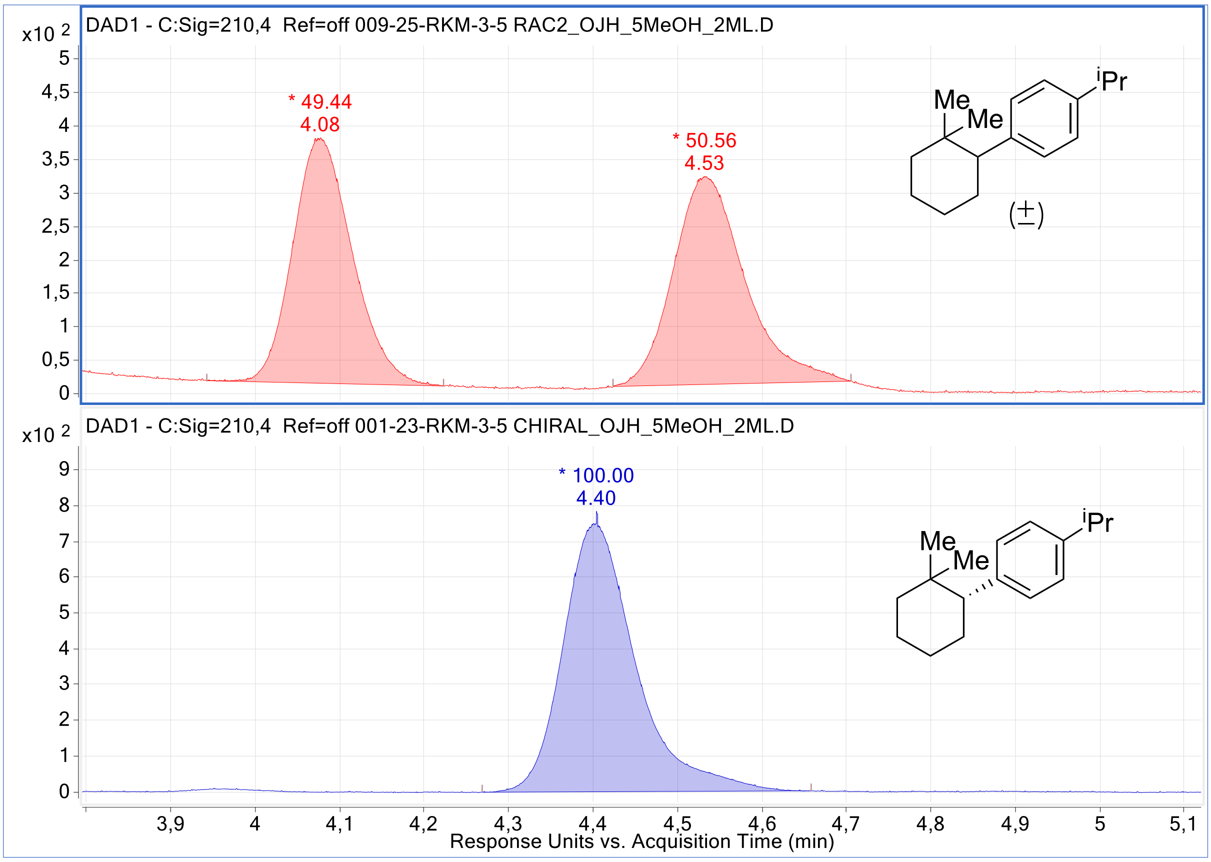
x10 2
DAD1 - C:Sig=210,4 Ref=off 009-25-RKM-3-5 RAC2_OJH_5MeOH_2ML.D
* 49.44
4.08
* 50.56
4.53
Me
Me
iPr
(±)
x10 2
DAD1 - C:Sig=210,4 Ref=off 001-23-RKM-3-5 CHIRAL_OJH_5MeOH_2ML.D
* 100.00
4.40
Me
Me
iPr
Response Units vs. Acquisition Time (min)

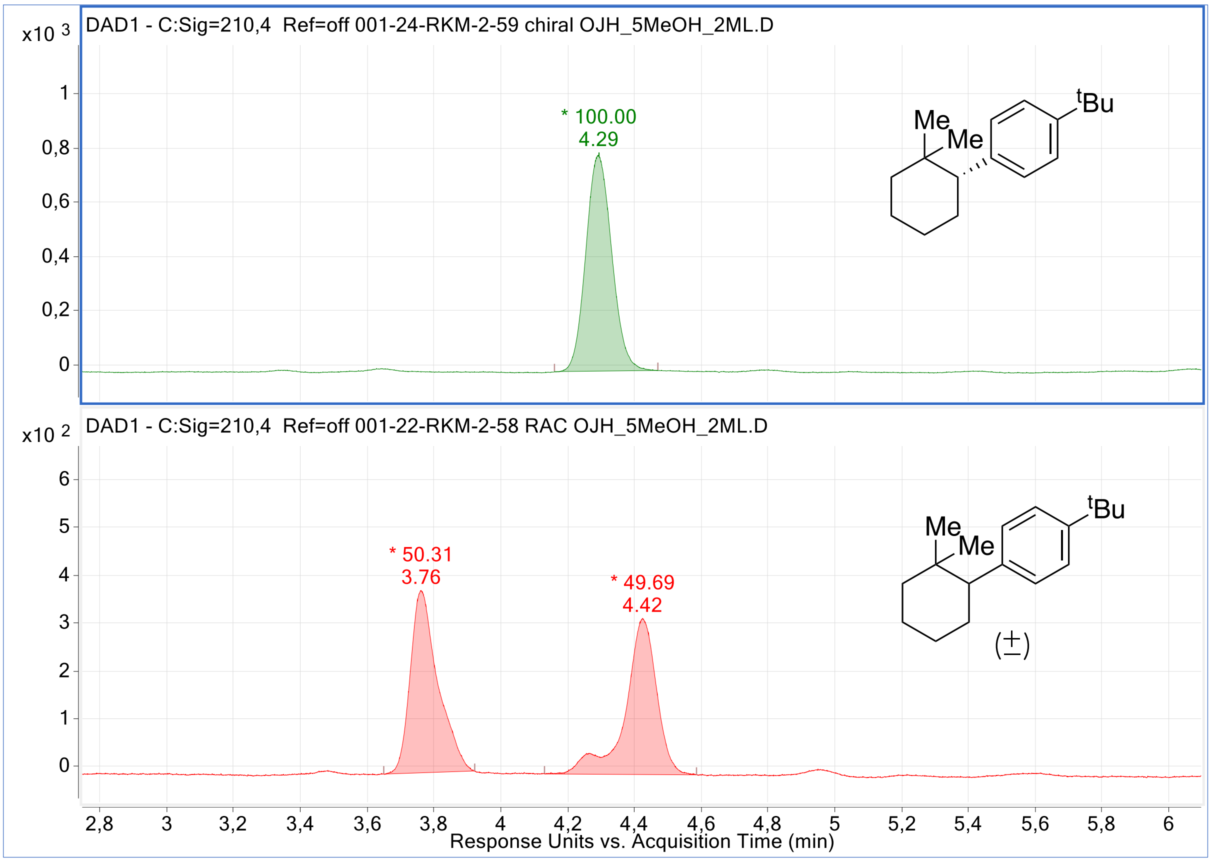

x10 3
DAD1 - C:Sig=210,4 Ref=off 001-24-RKM-2-59 chiral OJH_5MeOH_2ML.D
* 100.00
4.29
Me
Me
tBu
x10 2
DAD1 - C:Sig=210,4 Ref=off 001-22-RKM-2-58 RAC OJH_5MeOH_2ML.D
* 50.31
3.76
* 49.69
4.42
(±)
Response Units vs. Acquisition Time (min)

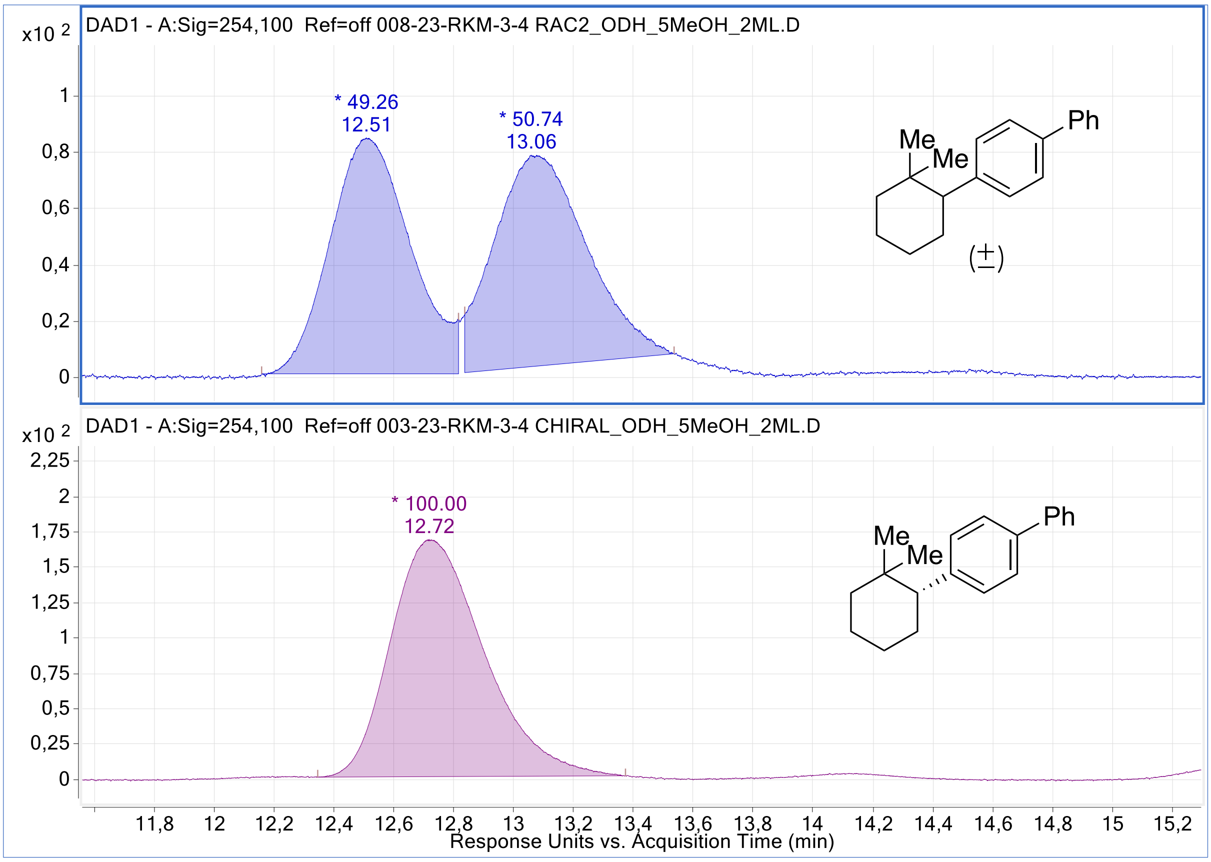

x10 2
DAD1 - A:Sig=254,100 Ref=off 008-23-RKM-3-4 RAC2_ODH_5MeOH_2ML.D
* 49.26
12.51
* 50.74
13.06
Me
Me
Ph
(±)
x10 2
DAD1 - A:Sig=254,100 Ref=off 003-23-RKM-3-4 CHIRAL_ODH_5MeOH_2ML.D
* 100.00
12.72
Response Units vs. Acquisition Time (min)

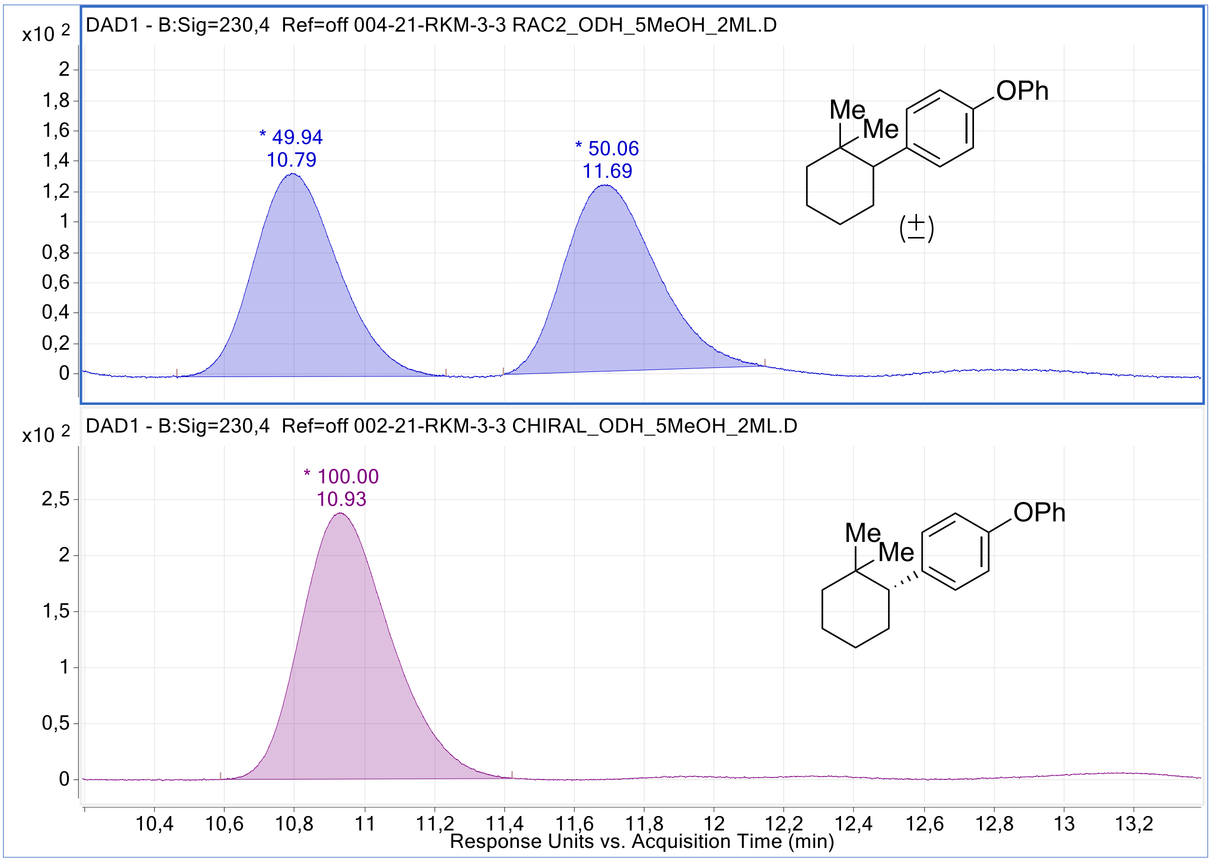
DAD1 - B:Sig=230,4 Ref=off 004-21-RKM-3-3 RAC2_ODH_5MeOH_2ML.D
* 49.94
10.79
* 50.06
11.69
OPh
Me
Me
(±)
DAD1 - B:Sig=230,4 Ref=off 002-21-RKM-3-3 CHIRAL_ODH_5MeOH_2ML.D
* 100.00
10.93
OPh
Me
Me
Response Units vs. Acquisition Time (min)

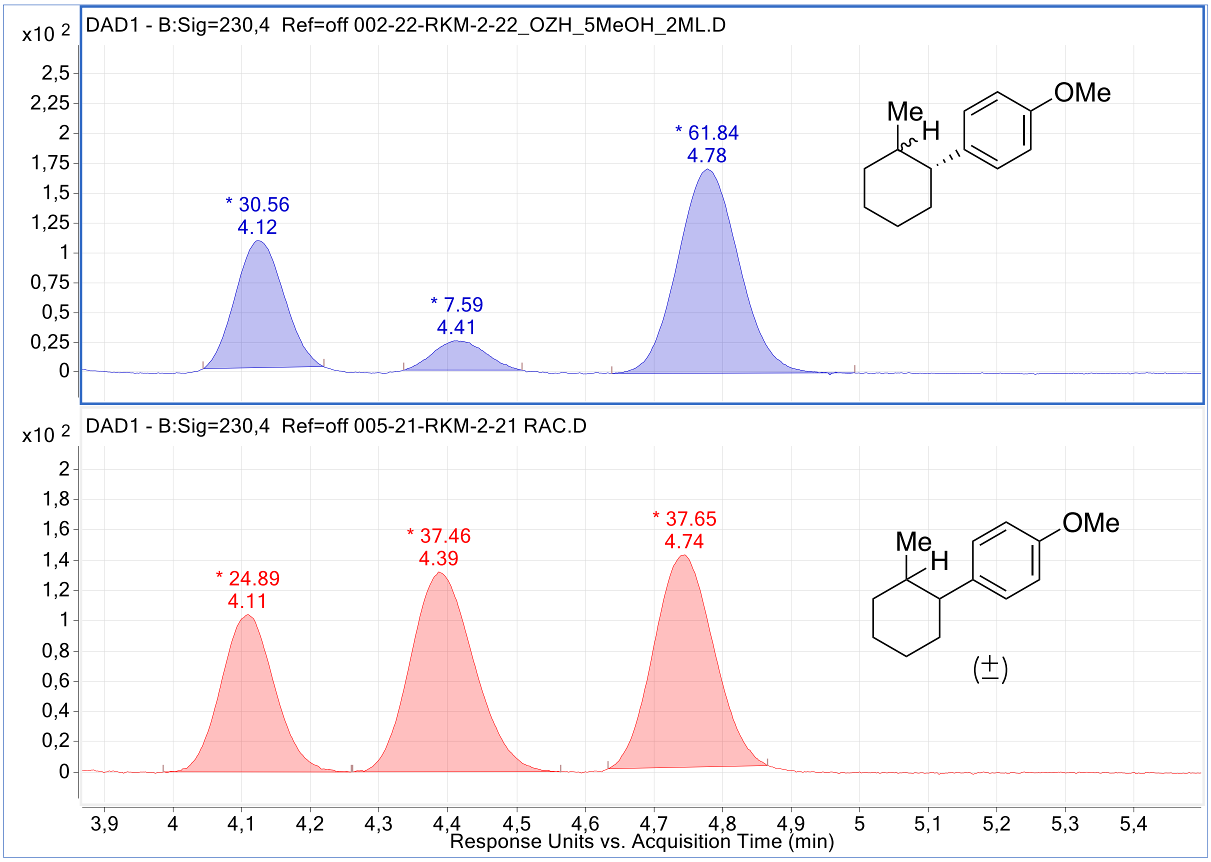
DAD1 - B:Sig=230,4 Ref=off 002-22-RKM-2-22_OZH_5MeOH_2ML.D
* 30.56
4.12
* 7.59
4.41
* 61.84
4.78
OMe
Me
H
DAD1 - B:Sig=230,4 Ref=off 005-21-RKM-2-21 RAC.D
* 24.89
4.11
* 37.46
4.39
* 37.65
4.74
OMe
Me
H
(±)
Response Units vs. Acquisition Time (min)

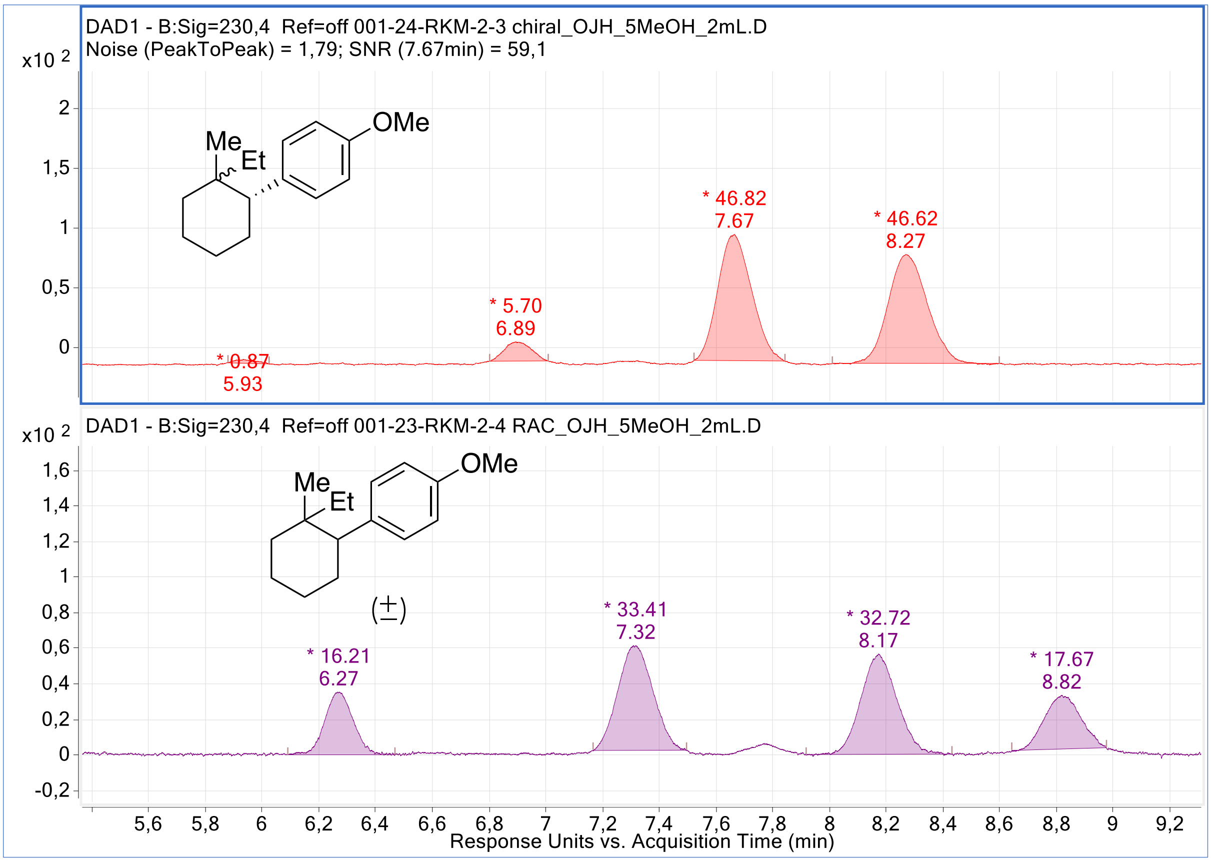

DAD1 - B:Sig=230,4 Ref=off 001-24-RKM-2-3 chiral_OJH_5MeOH_2mL.D
Noise (PeakToPeak) = 1,79; SNR (7.67min) = 59,1
x10 2
Me
Et
OMe
* 0.87
5.93
* 5.70
6.89
* 46.82
7.67
* 46.62
8.27
DAD1 - B:Sig=230,4 Ref=off 001-23-RKM-2-4 RAC_OJH_5MeOH_2mL.D
Me
Et
OMe
(±)
* 16.21
6.27
* 33.41
7.32
* 32.72
8.17
* 17.67
8.82
Response Units vs. Acquisition Time (min)

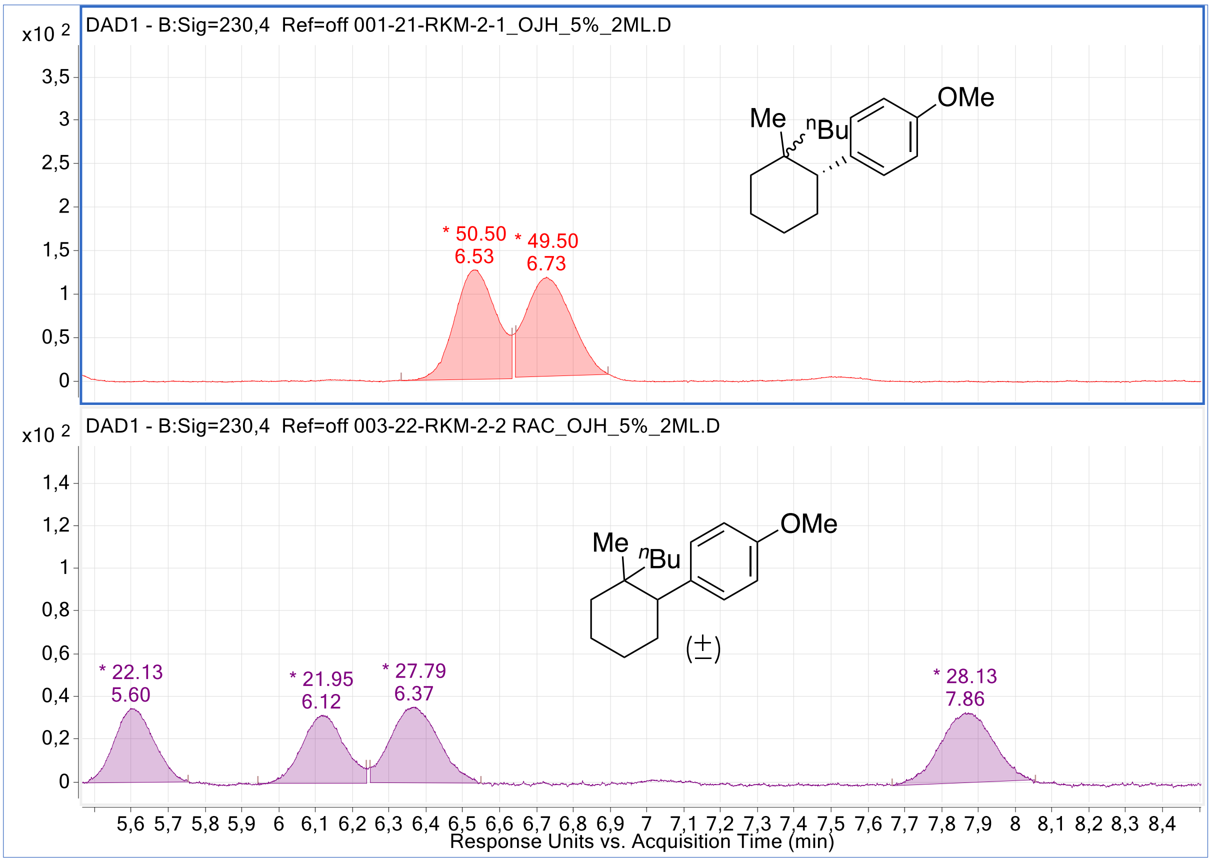

DAD1 - B:Sig=230,4 Ref=off 001-21-RKM-2-1_OJH_5%_2ML.D
x10 2
Me
nBu
OMe
* 50.50
6.53
* 49.50
6.73
DAD1 - B:Sig=230,4 Ref=off 003-22-RKM-2-2 RAC_OJH_5%_2ML.D
Me
nBu
OMe
(±)
* 22.13
5.60
* 21.95
6.12
* 27.79
6.37
* 28.13
7.86
Response Units vs. Acquisition Time (min)

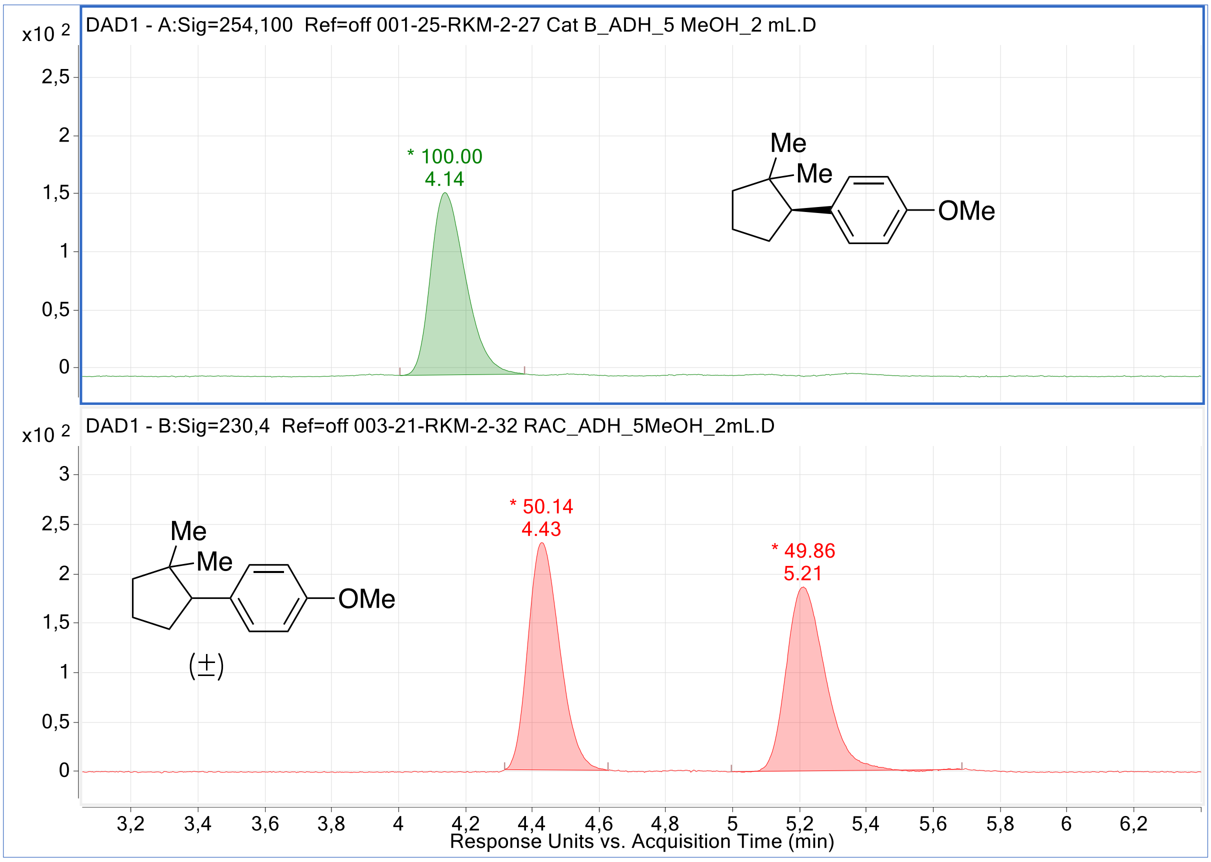
x10 2
DAD1 - A:Sig=254,100 Ref=off 001-25-RKM-2-27 Cat B_ADH_5 MeOH_2 mL.D
* 100.00
4.14
Me
Me
OMe
x10 2
DAD1 - B:Sig=230,4 Ref=off 003-21-RKM-2-32 RAC_ADH_5MeOH_2mL.D
* 50.14
4.43
* 49.86
5.21
Me
Me
OMe
(±)
Response Units vs. Acquisition Time (min)

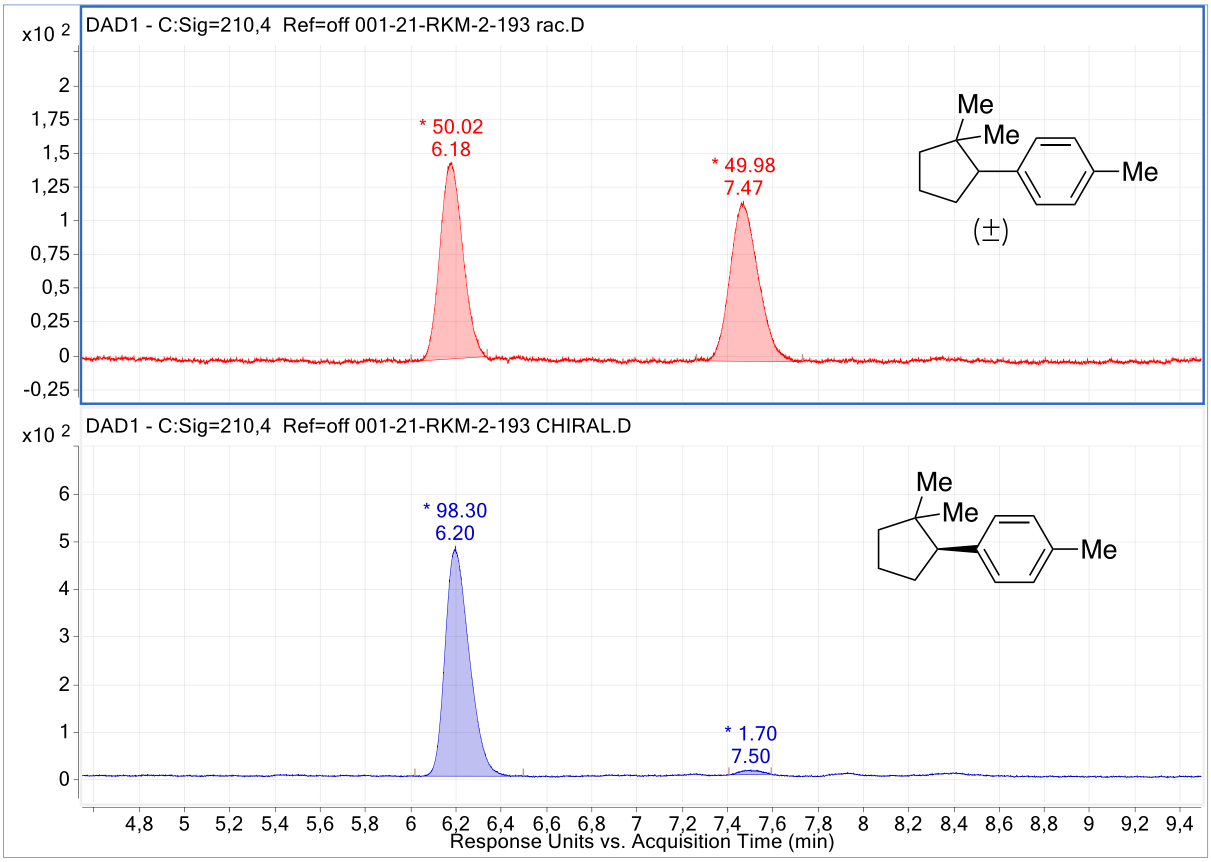
x10 2
DAD1 - C:Sig=210,4 Ref=off 001-21-RKM-2-193 rac.D
* 50.02
6.18
* 49.98
7.47
Me
Me
Me
(±)
x10 2
DAD1 - C:Sig=210,4 Ref=off 001-21-RKM-2-193 CHIRAL.D
* 98.30
6.20
* 1.70
7.50
Me
Me
Me
Response Units vs. Acquisition Time (min)

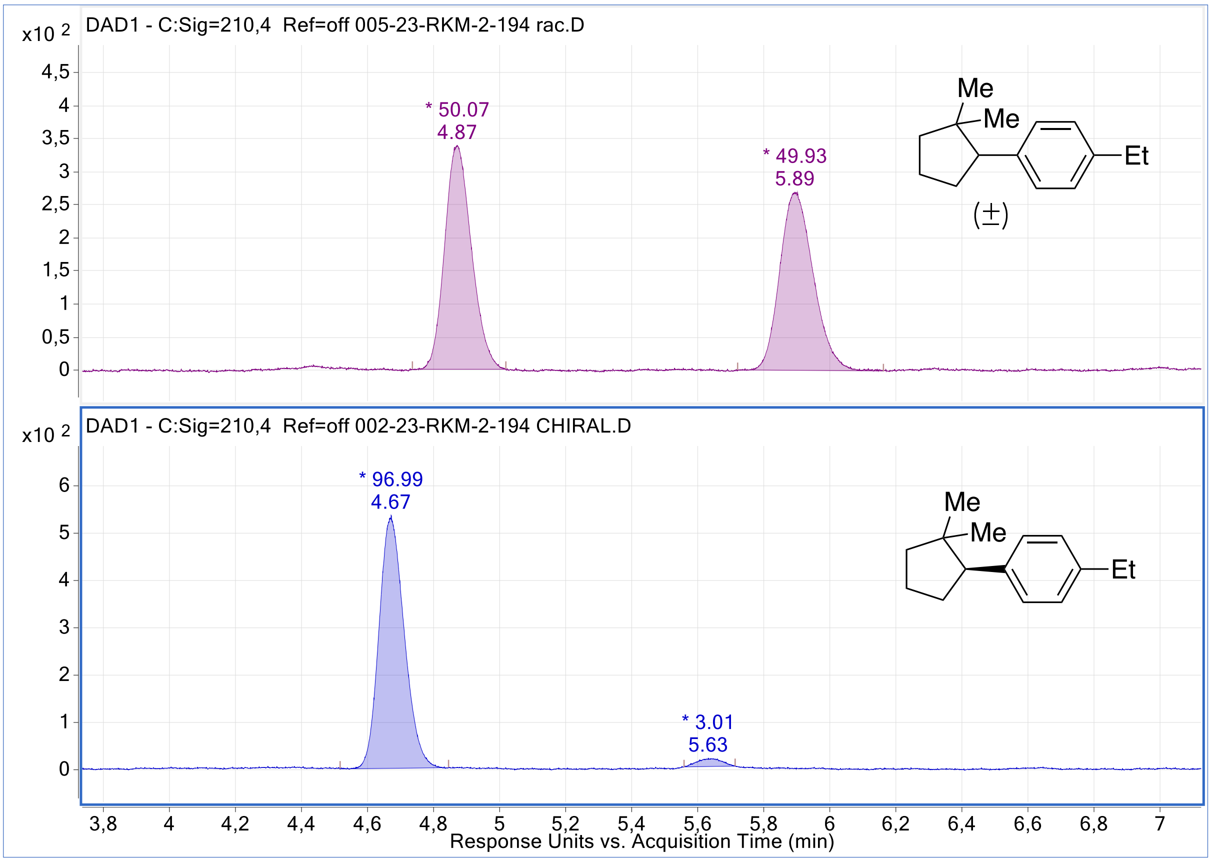
x10 2
DAD1 - C:Sig=210,4 Ref=off 005-23-RKM-2-194 rac.D
* 50.07
4.87
* 49.93
5.89
Me
Me
Et
(±)
x10 2
DAD1 - C:Sig=210,4 Ref=off 002-23-RKM-2-194 CHIRAL.D
* 96.99
4.67
* 3.01
5.63
Me
Me
Et
Response Units vs. Acquisition Time (min)

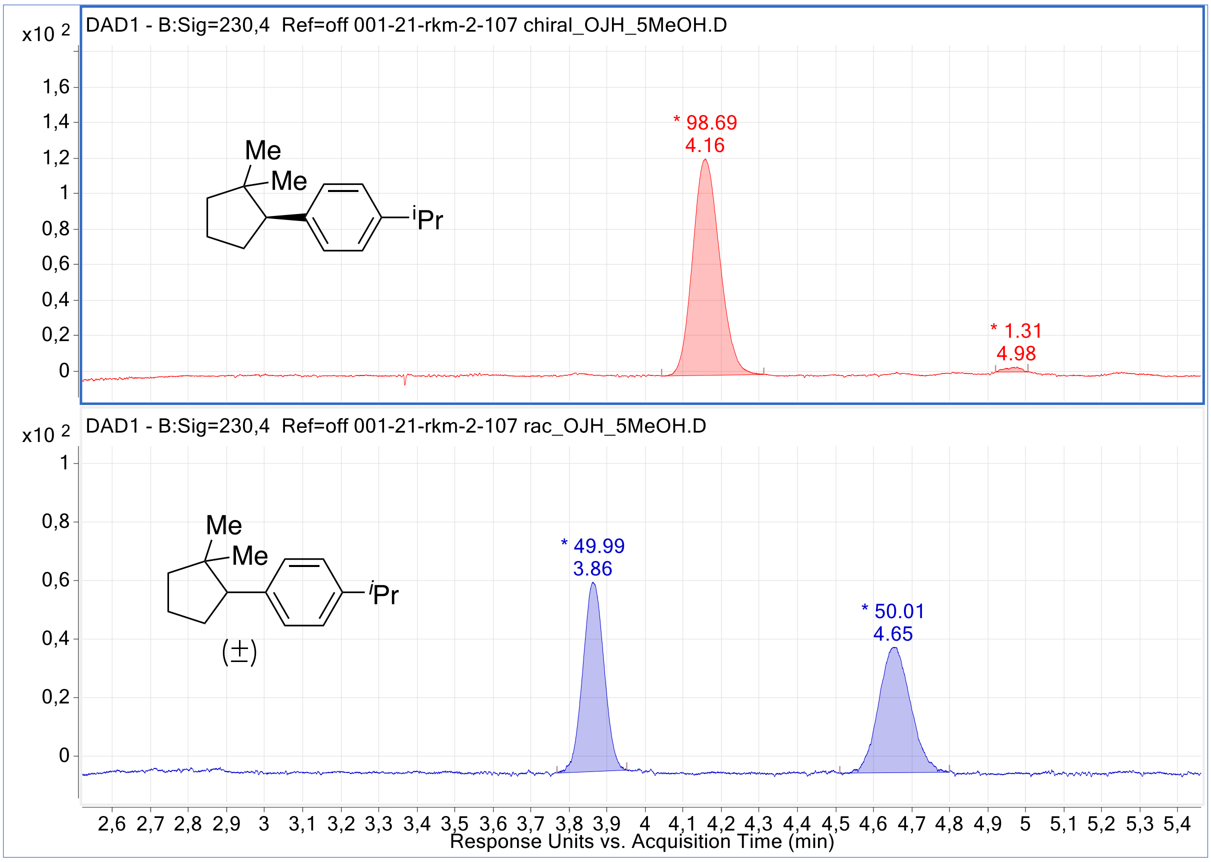
x10 2
DAD1 - B:Sig=230,4 Ref=off 001-21-rkm-2-107 chiral_OJH_5MeOH.D
Me
Me
iPr
* 98.69
4.16
* 1.31
4.98
x10 2
DAD1 - B:Sig=230,4 Ref=off 001-21-rkm-2-107 rac_OJH_5MeOH.D
Me
Me
iPr
(±)
* 49.99
3.86
* 50.01
4.65
Response Units vs. Acquisition Time (min)

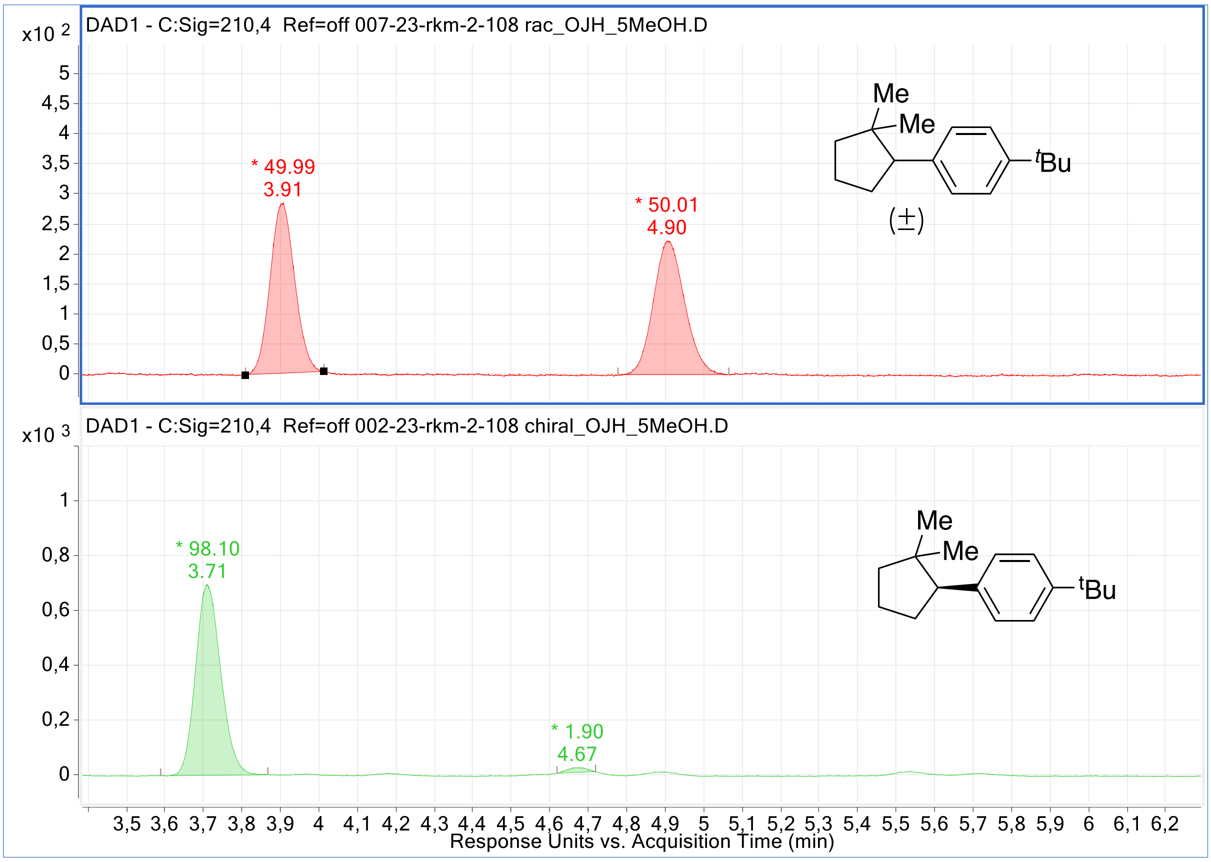
x10 2
DAD1 - C:Sig=210,4 Ref=off 007-23-rkm-2-108 rac_OJH_5MeOH.D
* 49.99
3.91
* 50.01
4.90
Me
Me
tBu
(±)
x10 3
DAD1 - C:Sig=210,4 Ref=off 002-23-rkm-2-108 chiral_OJH_5MeOH.D
* 98.10
3.71
* 1.90
4.67
Me
Me
tBu
Response Units vs. Acquisition Time (min)

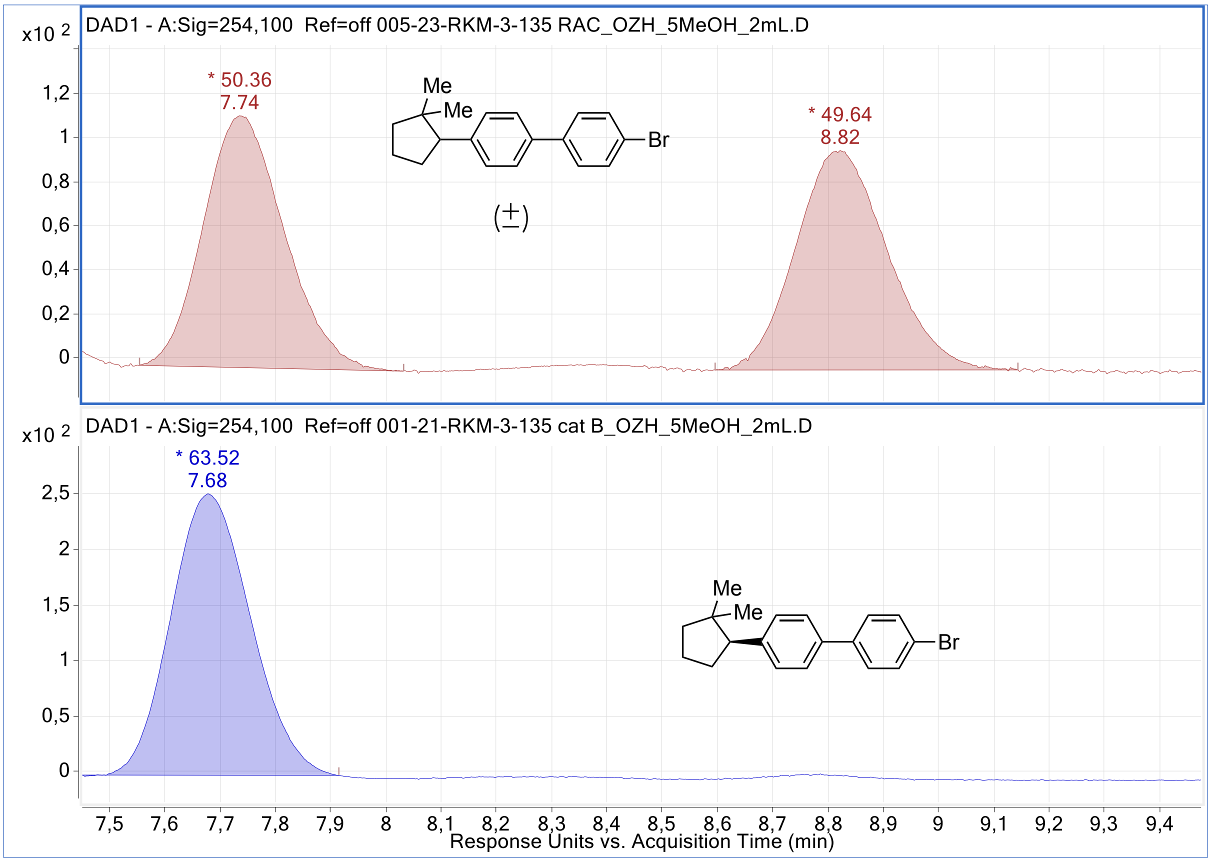
x10 2
DAD1 - A:Sig=254,100 Ref=off 005-23-RKM-3-135 RAC_OZH_5MeOH_2mL.D
* 50.36
7.74
* 49.64
8.82
Me
Me
Br
(±)
x10 2
DAD1 - A:Sig=254,100 Ref=off 001-21-RKM-3-135 cat B_OZH_5MeOH_2mL.D
* 63.52
7.68
Me
Me
Br
Response Units vs. Acquisition Time (min)

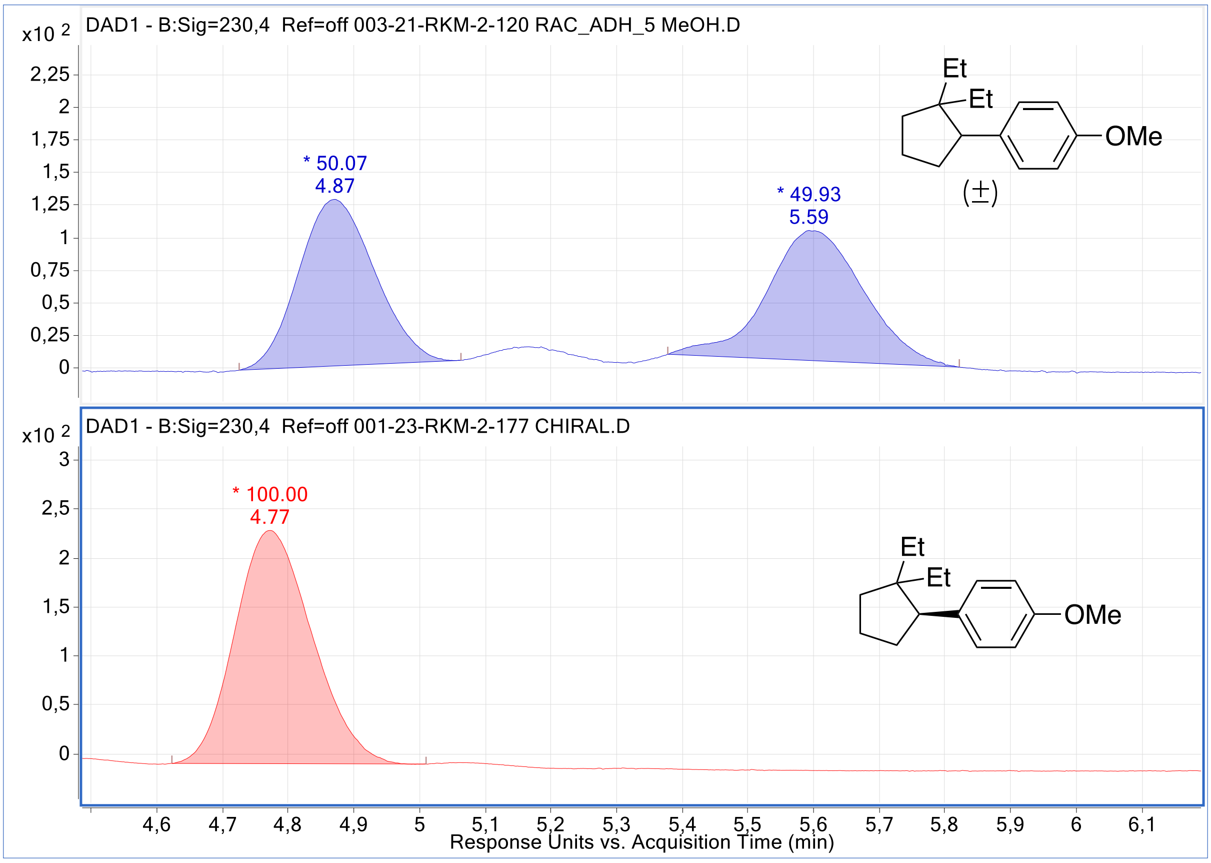

x10 2
DAD1 - B:Sig=230,4 Ref=off 003-21-RKM-2-120 RAC_ADH_5 MeOH.D
* 50.07
4.87
* 49.93
5.59
Et
Et
OMe
(±)
x10 2
DAD1 - B:Sig=230,4 Ref=off 001-23-RKM-2-177 CHIRAL.D
* 100.00
4.77
Et
Et
OMe
Response Units vs. Acquisition Time (min)

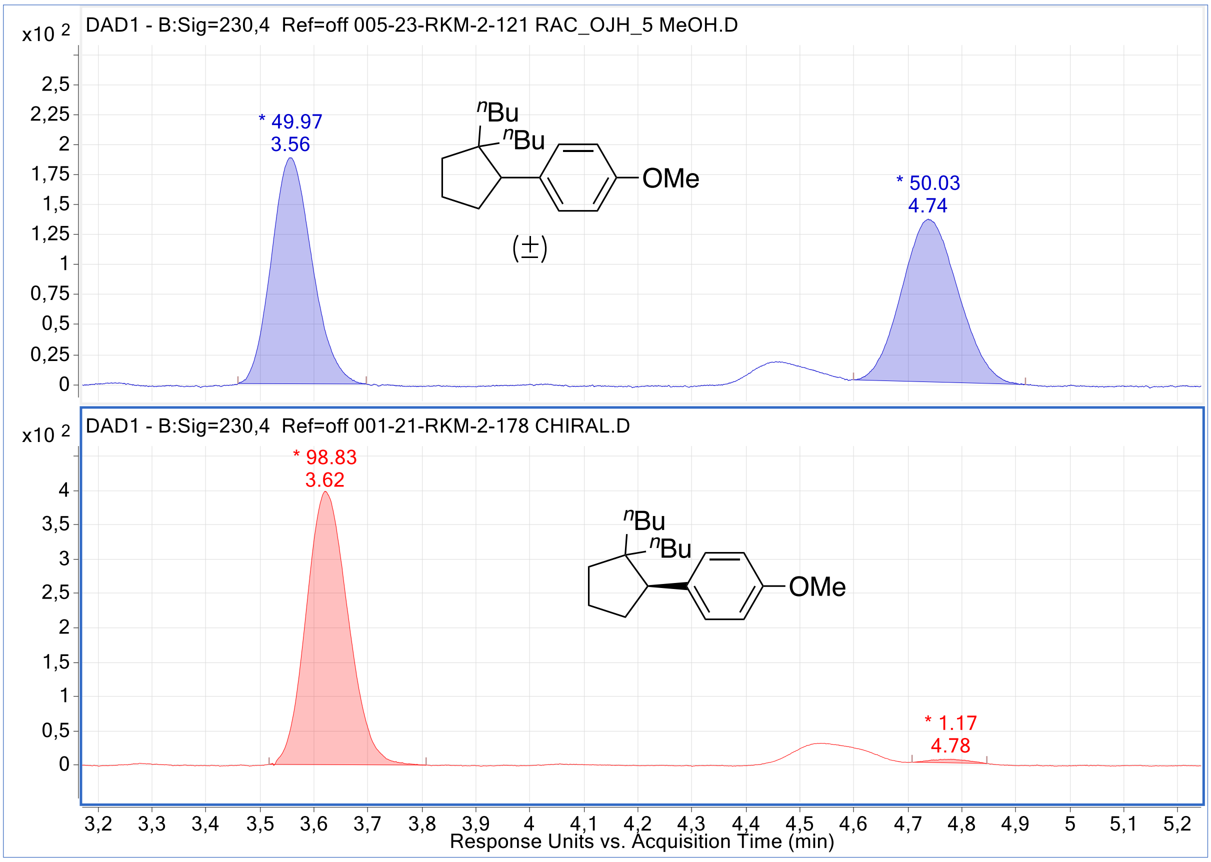

x10 2
DAD1 - B:Sig=230,4 Ref=off 005-23-RKM-2-121 RAC_OJH_5 MeOH.D
* 49.97
3.56
nBu
nBu
OMe
(±)
* 50.03
4.74
x10 2
DAD1 - B:Sig=230,4 Ref=off 001-21-RKM-2-178 CHIRAL.D
* 98.83
3.62
nBu
nBu
OMe
* 1.17
4.78
Response Units vs. Acquisition Time (min)

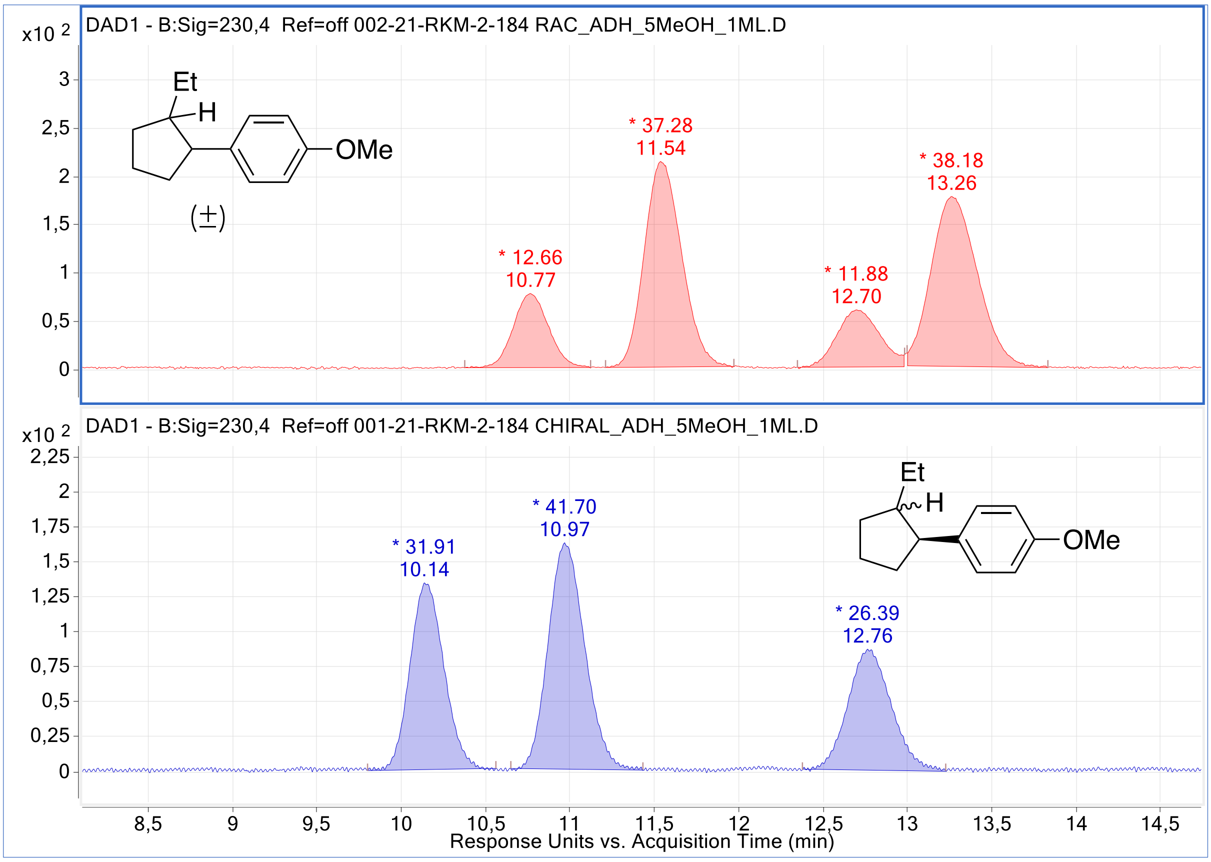
DAD1 - B:Sig=230,4 Ref=off 002-21-RKM-2-184 RAC_ADH_5MeOH_1ML.D
x10 2
Et
H
OMe
(±)
* 12.66
10.77
* 37.28
11.54
* 11.88
12.70
* 38.18
13.26
DAD1 - B:Sig=230,4 Ref=off 001-21-RKM-2-184 CHIRAL_ADH_5MeOH_1ML.D
* 31.91
10.14
* 41.70
10.97
* 26.39
12.76
Response Units vs. Acquisition Time (min)

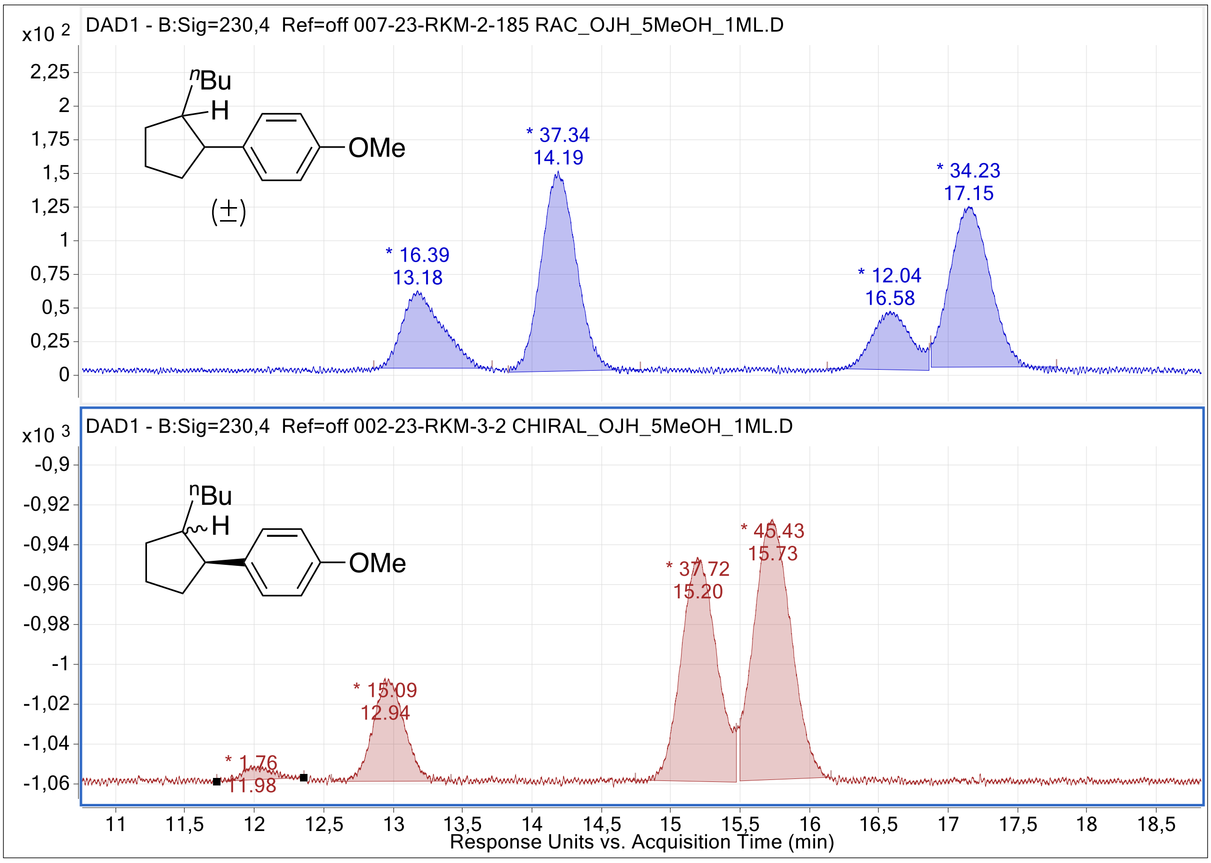
DAD1 - B:Sig=230,4 Ref=off 007-23-RKM-2-185 RAC_OJH_5MeOH_1ML.D
x10 2
ⁿBu
H
OMe
(±)
* 16.39
13.18
* 37.34
14.19
* 12.04
16.58
* 34.23
17.15
DAD1 - B:Sig=230,4 Ref=off 002-23-RKM-3-2 CHIRAL_OJH_5MeOH_1ML.D
x10 3
* 1.76
11.98
* 15.09
12.94
* 37.72
15.20
* 45.43
15.73
Response Units vs. Acquisition Time (min)

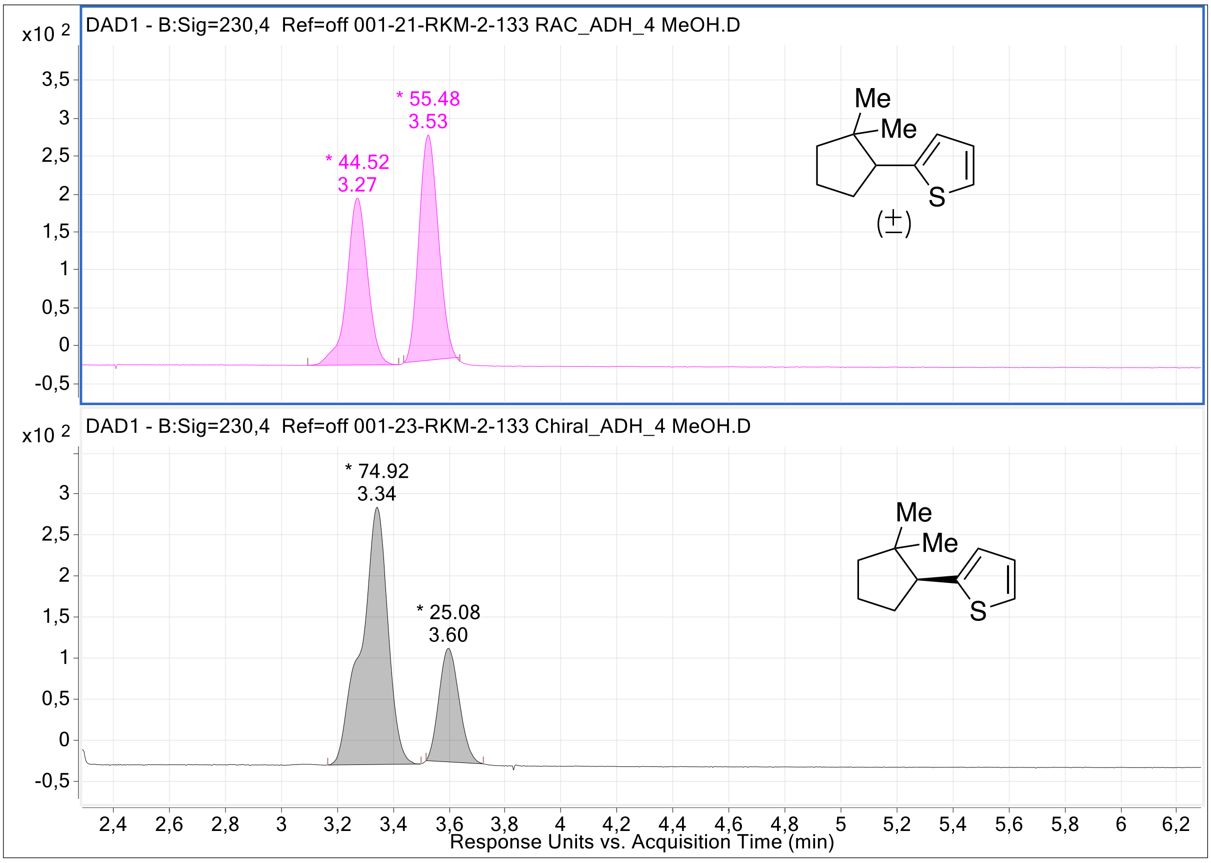
DAD1 - B:Sig=230,4 Ref=off 001-21-RKM-2-133 RAC_ADH_4 MeOH.D
* 44.52
3.27
* 55.48
3.53
(±)
DAD1 - B:Sig=230,4 Ref=off 001-23-RKM-2-133 Chiral_ADH_4 MeOH.D
* 74.92
3.34
* 25.08
3.60
Response Units vs. Acquisition Time (min)

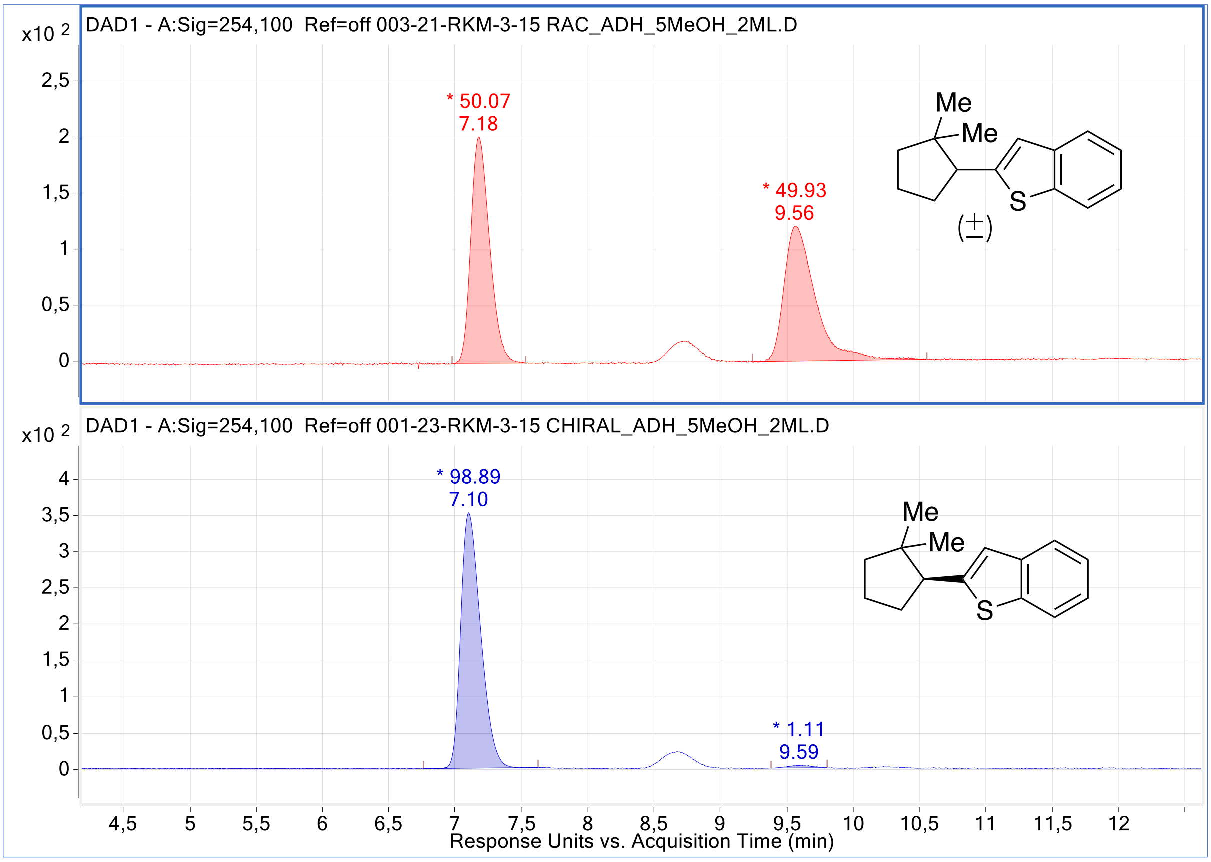
DAD1 - A:Sig=254,100 Ref=off 003-21-RKM-3-15 RAC_ADH_5MeOH_2ML.D
* 50.07
7.18
* 49.93
9.56
(±)
DAD1 - A:Sig=254,100 Ref=off 001-23-RKM-3-15 CHIRAL_ADH_5MeOH_2ML.D
* 98.89
7.10
* 1.11
9.59
Response Units vs. Acquisition Time (min)
